# Surface Proteome Analysis and Characterization of Surface Cell Antigen (Sca) or Autotransporter Family of *Rickettsia typhi*

**DOI:** 10.1371/journal.ppat.1002856

**Published:** 2012-08-09

**Authors:** Khandra T. Sears, Shane M. Ceraul, Joseph J. Gillespie, Edwin D. Allen, Vsevolod L. Popov, Nicole C. Ammerman, M. Sayeedur Rahman, Abdu F. Azad

**Affiliations:** 1 Department of Microbiology and Immunology, School of Medicine, University of Maryland Baltimore, Baltimore, Maryland, United States of America; 2 Virginia Bioinformatics Institute at Virginia Tech, Blacksburg, Virginia, United States of America; 3 Department of Biological Sciences, Towson University, Towson, Maryland, United States of America; 4 Center for Biodefense and Emerging Infectious Diseases, WHO Collaborating Center for Tropical Diseases, The University of Texas Medical Branch, Galveston, Texas, United States of America; 5 Center for Tuberculosis Research, Johns Hopkins University, Baltimore, Maryland, United States of America; Yale University School of Medicine, United States of America

## Abstract

Surface proteins of the obligate intracellular bacterium *Rickettsia typhi*, the agent of murine or endemic typhus fever, comprise an important interface for host-pathogen interactions including adherence, invasion and survival in the host cytoplasm. In this report, we present analyses of the surface exposed proteins of *R. typhi* based on a suite of predictive algorithms complemented by experimental surface-labeling with thiol-cleavable sulfo-NHS-SS-biotin and identification of labeled peptides by LC MS/MS. Further, we focus on proteins belonging to the surface cell antigen (Sca) autotransporter (AT) family which are known to be involved in rickettsial infection of mammalian cells. Each species of *Rickettsia* has a different complement of *sca* genes in various states; *R. typhi*, has genes *sca1* thru *sca5*. *In silico* analyses indicate divergence of the Sca paralogs across the four *Rickettsia* groups and concur with previous evidence of positive selection. Transcripts for each *sca* were detected during infection of L929 cells and four of the five Sca proteins were detected in the surface proteome analysis. We observed that each *R. typhi* Sca protein is expressed during *in vitro* infections and selected Sca proteins were expressed during *in vivo* infections. Using biotin-affinity pull down assays, negative staining electron microscopy, and flow cytometry, we demonstrate that the Sca proteins in *R. typhi* are localized to the surface of the bacteria. All Scas were detected during infection of L929 cells by immunogold electron microscopy. Immunofluorescence assays demonstrate that Scas 1–3 and 5 are expressed in the spleens of infected Sprague-Dawley rats and Scas 3, 4 and 5 are expressed in cat fleas (*Ctenocephalides felis*). Sca proteins may be crucial in the recognition and invasion of different host cell types. In short, continuous expression of all Scas may ensure that rickettsiae are primed i) to infect mammalian cells should the flea bite a host, ii) to remain infectious when extracellular and iii) to infect the flea midgut when ingested with a blood meal. Each Sca protein may be important for survival of *R. typhi* and the lack of host restricted expression may indicate a strategy of preparedness for infection of a new host.

## Author Summary

*Rickettsia typhi*, a member of the typhus group (TG) rickettsia, is the agent of murine or endemic typhus fever – a disease exhibiting mild to severe flu-like symptoms resulting in significant morbidity. It is maintained in a flearodent transmission cycle in urban and suburban environments. The obligate intracellular lifestyle of rickettsiae makes genetic manipulation difficult and impedes progress towards identification of virulence factors. All five Scas were detected on the surface of *R.. typhi* using a combination of a biotin-labeled affinity assay, negative stain electron microscopy and flow cytometry. Sca proteins are members of the autotransporter (AT) family or type V secretion system (TVSS). We employed detailed bioinformatic analyses and evaluated their transcript abundance in an *in vitro* infection model where *sca* transcripts are detected at varying levels over the course of a 5 day *in vitro* infection. We also observe expression of selected Sca proteins during infection of fleas and rats. Our study provides a proteomic analysis of the bacterial surface and an initial characterization of the Sca family as it exists in *R. typhi*.

## Introduction

Rickettsia (*Rickettsiales*: *Rickettsiaceae*) are Gram-negative, obligate intracellular bacteria that are maintained in enzootic cycles involving both hematophagous arthropod vectors and vertebrate hosts [1]. Rickettsiae are the causative agents of significant human diseases such as Rocky Mountain spotted fever (*R. rickettsii*) and epidemic typhus (*R. prowazekii*). Classically, the members of the genus *Rickettsia* have been divided into two groups: the tick-transmitted spotted fever group (SFG) and the insect-transmitted typhus group (TG) based on their antigenic and molecular profiles. However, these groups share some antigenic proteins such as outer membrane protein B (OmpB) and 17 kDa lipoprotein [2]. The tick-transmitted SFG currently includes over 16 species, several of which are known human pathogens (*R. rickettsii*, *R. conorii*, *and R. sibirica*). The louse and flea transmitted TG rickettsia contain the pathogenic species *R. prowazekii* and *R. typhi* (causative agent of murine typhus). Extensive phylogenetic and comparative genomic analyses have resulted in the proposal of the ancestral group (AG) and transitional group (TRG) rickettsia and these include species with mild or unknown pathogenicity as well as broad arthropod host ranges [3], [4], [5].

Despite the recent advances made in rickettsial molecular biology and genomics, their determinants of pathogenicity still remain undefined. Because of the involvement of rickettsial Omps in cell surface recognition, initial binding of bacteria to host cells, invasion processes [6], [7] as well as their immunogenicity and utility as vaccine candidates, this group of proteins has been a target of interest [8], [9], [10], [11]. Bacterial surface-exposed proteins are involved in an array of processes including sensing the environment, protection from environmental stresses, adherence to and invasion of host cells, cell growth and interaction with the immune system. For intracellular bacteria, surface exposed proteins interact with host cytoplasmic or organelle proteins [12], [13]. Characterizing the surface composition of rickettsiae allows for identification of factors required for successful colonization of mammalian and arthropod hosts.

The family of rickettsial autotransporters (ATs) are referred to as surface cell antigen (Sca) proteins [14]. A previous computation analysis identified 17 orthologs *Sca0* (*OmpA*), *Sca1-Sca4*, *Sca5* (*OmpB*), *Sca6-Sca16*) distributed throughout nine complete rickettsial genome sequences [15]. These genomes as well as those subsequently sequenced, each encode a unique repertoire of *sca* orthologs that are present in different functional states (i.e. complete, fragmented or split). Furthermore, while the AT domains are well conserved within orthologs and to a lesser degree across paralogs, much less amino acid identity is observed among the passenger domains [15]. Experimental studies have detected expression of Sca0, Sca5 and Sca4 by various methods [16], [17], [18], however, only Sca0, Sca5 and more recently Sca1 and Sca2 of *R. conorii* have been shown to function as adhesins [6], [19], [20], [21]. The internal repeat motifs within the passenger domains are proposed to render each protein an adhesin and contribute specificity to host receptors [15]. For *R. conorii* Sca5, these repeats may contribute to its specificity for the DNA protein kinase Ku70 on the surface of host cells [22]. More recently, Sca2 in *R. parkeri* has been characterized as a formin-like mediator of actin-based motility [23] indicating that some of the Scas have intracellular functions and may interact with host proteins to promote rickettsial survival.

*R. typhi*, the focal organism of this study, contains 5 *sca* orthologs in its genome – *sca1, sca2, sca3, sca4* and *sca5*. As in other species studied, Sca5 likely mediates adherence and invasion of *R. typhi* to host cells, however little is known about the expression and function of the other Sca orthologs during *R. typhi* infection in either mammalian or arthropod hosts. A better understanding of the expression and distribution of the Scas during *R. typhi* transmission and infection is crucial in order to appreciate the function of these proteins. This study is a comparative analysis that was undertaken to elucidate the transcriptional and protein expression profiles of the *R. typhi Sca* family *in vitro* (tissue culture) and *in vivo* (rat and flea infections). This study addresses our hypothesis that *R. typhi Sca* expression is time and host dependent.

## Results

### Surface proteome analysis of *R. typhi* str. Wilmington

**Figure 1:**
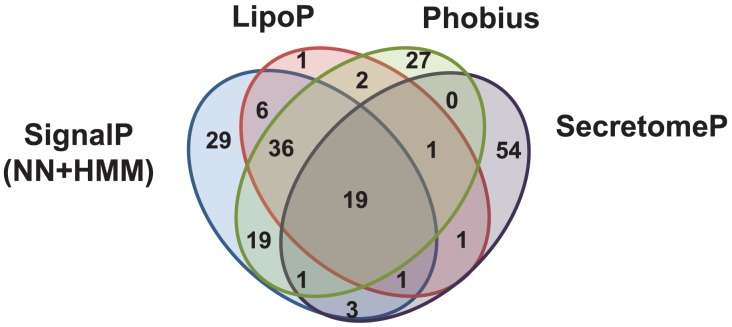
Venn diagram of the number of proteins predicted to be secreted by the indicated algorithms. The entire *R. typhi* str. Wilmington proteome was submitted to SignalP, LipoP, Phobius and SecretomeP servers as outlined in the Materials & Methods section. Results from each program were compiled and the numbers of proteins predicted by each program individually or in all possible combinations were tabulated and are indicated in the above Venn diagram.

Surface proteins of intracellular bacteria mediate interactions required for their pathogenesis and survival. Defining the surface proteome of *R. typhi* provides a better understanding of the potential interactions at the host-pathogen interface. Using CoBaltDB [24] and pSORTb v. 3.0 [25], several signal peptide and subcellular localization algorithms were employed to generate predictions of secreted and outer membrane proteins among the 838 ORFs in *R. typhi*. Of the 838 ORFs, 140 were predicted to be secreted by at least one of the four algorithms employed (Figure 1). Further, 25 proteins had no detectable secretion signals but were predicted to be localized to the membrane or extracellular to bacteria (Table S1, proteins highlighted in yellow). Only the SOSUI-GramN [26] and PSORTb v. 3.0 [27] algorithms identified proteins localized to the cytoplasmic membrane, inner membrane or outer membrane (Tables S1 and S2).

In a previous study, putative secretion signals *R. typhi* ORFs were tested for the ability to facilitate secretion to the periplasm via a sec-translocon dependent mechanism in *E. coli* [28]. We further analyzed all *R. typhi* str. Wilmington ORFs using the SecretomeP method [29] which identifies non-classically secreted proteins using a sequence-derived feature based approach and was trained on proteins experimentally identified on the bacterial surface but not predicted to be secreted by SignalP algorithms. Based on the SecretomeP method, 26 of the proteins with classical secretion signals also have signals for non-classical secretion methods (Figure 1 and Table S1, proteins highlighted in green). Fifty-four proteins were predicted to have only non-classical signals (Figure 1 and Table S2).

Only a fraction of the proteins predicted to be secreted were identified in the surface proteome analysis. A total of 68 proteins were detected (Table S3), 27 of which are predicted to be secreted (to the periplasm via the Sec translocon as determined by SignalP) or surface-exposed (in the outer membrane or extracellular to bacteria as determined by SOSUIGramN and pSORTb) by at least one of the algorithms used. Fifteen of the proteins are predicted to have secretion signal peptides as determined by SignalP NN and/or HMM algorithms. pSORTb v. 3.0 [25] predicted three proteins to be localized to the outer membrane [RT0565 and RT0699(Sca5)] or be extracellular (RT0522). SOSUIGramN localized 11 proteins to either the outer membrane (RT0521, RT0744 and RT0805) or extracellular [RT0052(Sca2), RT0699(Sca5), etc.] to bacteria (Table S3 cells shaded grey). LipoP, Phobius and SecretomeP were the only algorithms to predict signal sequences for one (RT0117), two (RT0222 and RT0584) and five (RT0138, RT0176, RT0362, RT0485 and RT0638) proteins, respectively (Tables S3). The predicted signal peptides for 11 of these 68 proteins were previously tested for Sec-translocon dependent secretion into the periplasm using an alkaline phosphatase (PhoA) gene fusion system (see footnotes Table S3) and 10 of them were found to mediate secretion [28]. Proteins predicted by the SecretomeP method may be present in the outer membrane as a result of Sec-independent secretion mechanisms. Most of the peptides identified are predicted to be cytoplasmic or have unknown localizations with few of them predicted to be localized to the surface of the bacterium as determined by pSORTb and SOSUIGramN. The expression of cytoplasmic proteins on the surface of bacteria is not uncommon. Of the proteins identified in this study, 18 have homologs that have been shown to be surface exposed in other rickettsial species [30], [31], [32], [33], other Gram-negative bacteria [34], [35], [36] and Gram-positive bacteria [37]. Additionally, 14 of the proteins are annotated as hypothetical and only 5 of the 14 have predicted signal peptides detected by at least one of the algorithms further suggesting that rickettsial proteins may have novel secretion signals.

The identification of most of the Sca proteins on the surface was largely consistent with the localization predictions (Table S1). Specifically, both pSORTb algorithms, LipoP, Phobius and SecretomeP predicted the presence of signal peptides for Sca1-3 and Sca5. SOSUI-GramN and pSORTb also localized each of these proteins as extracellular to the bacteria or to the outer membrane. SecretomeP was the only program to predict a signal sequence within Sca4 (Table S2); it is also predicted to be localized to the cytoplasm by the SOSUI-GramN and pSORTb algorithms.

### *In silico* characterization of *R. typhi* Sca proteins

**Figure 2:**
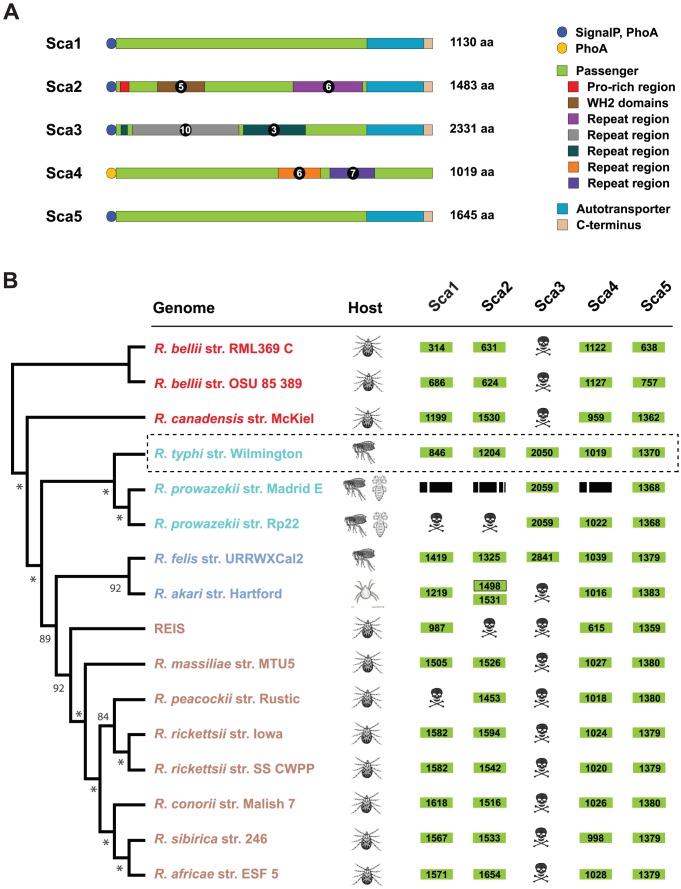
Characteristics of rickettsial surface cell antigens (Scas). A) Schema depicting the major features of *R. typhi* Sca1 (RT0015, YP_066986), Sca2 (RT0052, YP_067021), Sca3 (RT0438, YP_067397), Sca4 (RT0485, YP_067439), and Sca5 (RT0699, YP_067640). N-terminal circles depict predicted (SignalP) and/or experimentally-supported (PhoA fusion assay) sec dependent signal sequences. Divergent repeat regions [77] and other identified motifs are colored coded for Sca2, Sca3 and Sca4. B) Comparative analysis of Sca1-Sca5 across 16 rickettsia genomes. Dashed box encloses the *R. typhi* Scas. Phylogeny at left is based on whole genome analysis [82], with rickettsial groups as follows: red, ancestral group; aquamarine, typhus group; blue, transitional group, brown, spotted fever group. Principal host vectors (tick, flea, louse, mite) for each taxon are illustrated. Green boxes depict passenger domains (as illustrated in A) associated with an AT domain, with the length of each passenger given in amino acids. Skull and crossbones represent pseduogenes. Black boxes depict *sca* gene fragments encoded within the *R. prowazekii* str. Madrid E genome (all of which have a conserved downstream ORF encoding an AT domain). Accession numbers for all sequences are provided in Figure S1.

Sequence analysis of Sca1-Sca5 of *R. typhi* indicates that all five proteins are full length and contain characteristics typical of other Scas from rickettsiae (Figure 2A). Repeat regions were predicted within only three of the five proteins (Sca2, Sca3 and Sca4). Consistent with a sequence analysis of Sca2 from *R. parkeri* [23], Sca2 of *R. typhi* contains a proline-rich tract and a series of five WH2 domains; however, the position of these motifs within the passenger domain of *R. typhi* (as well as *R. bellii*, *R. prowazekii* and one of the two *R. akari* proteins) differs greatly from *R. parkeri* and the other orthologs of SFG Rickettsiae (Text S2). In general, the passenger domains from Sca1, Sca2, Sca3, and the full Sca4 protein differ in sequence length, percent amino acid identity, and number of repeat regions across the 16 analyzed *Rickettsia* taxa (Figure 2B). While the distribution of the Sca paralogs across these 16 taxa somewhat correlates with the four rickettsia groups, only the *R. felis* genome encodes a full-length ortholog for each of the five *R. typhi* Scas (Text S2 and Figure S1), which like *R. typhi*, is vectored principally by fleas.

### *Sca* gene transcription during *in vitro R. typhi* growth and *in vivo* protein expression

**Figure 3:**
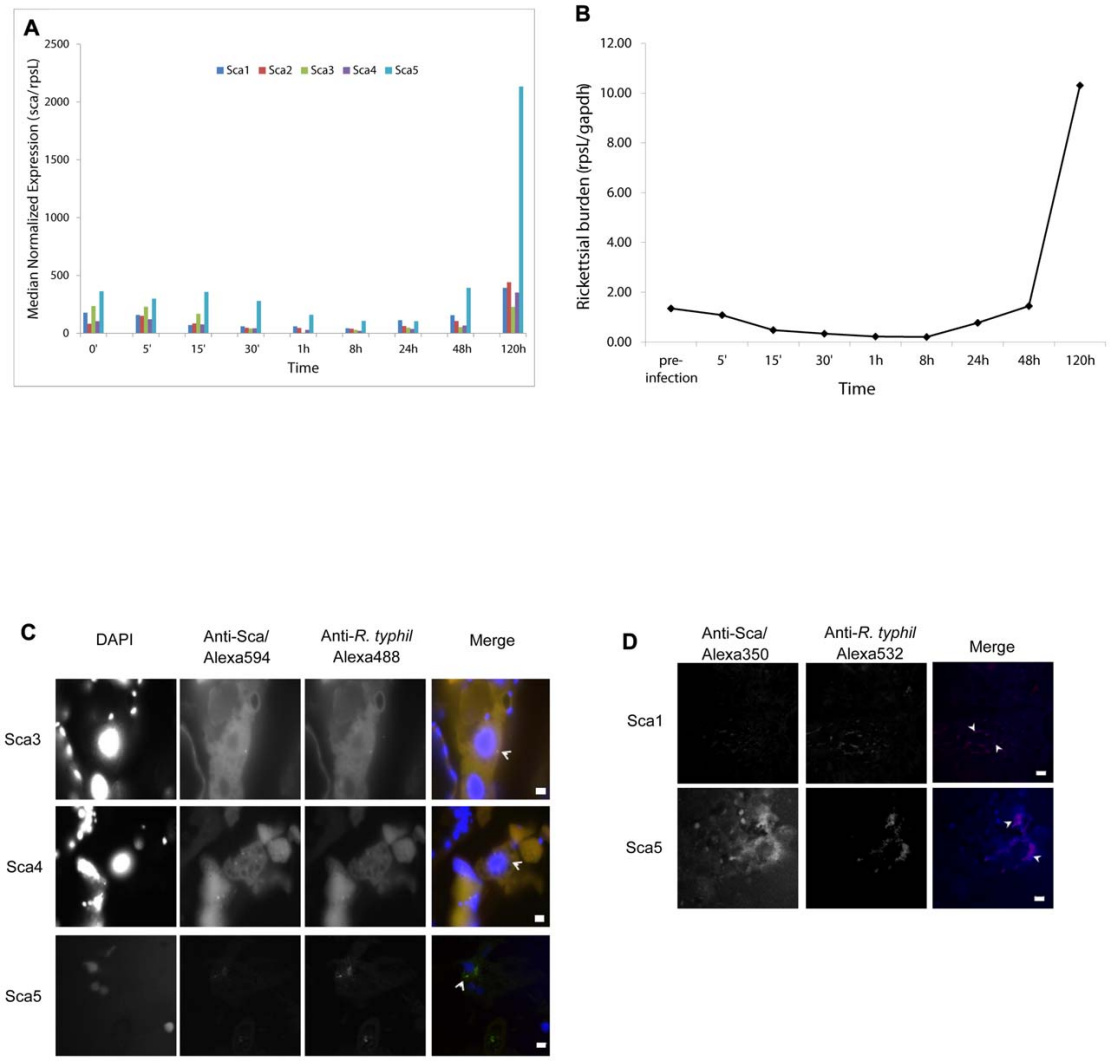
Expression analysis by real-time RT-PCR of *Sca* genes during L929 cell infection and immunofluorescence assays of Sca protein expression in *C. felis* and rat spleens. RNA was extracted from *R. typhi* infected L929 cells at different time points as described and analyzed by multiplex real-time qPCR for expression of *Sca*, *rpsL* and *GAPDH* genes. A) Transcript abundance of each *Sca* gene was normalized to *rpsL* transcript abundance and B) rickettsial burden (*rpsL*: *GAPDH*) confirmed increased infection over time. The results represent data from 3 experiments with each gene analyzed in duplicate. C) Cat fleas were housed in an artificial dog and fed uninfected or *R. typhi* infected whole sheep's blood for 48 h.; they were then fed uninfected blood for 8 days. Capsules were placed at −20°C to immobilize fleas before placing them in 3% PFA overnight. Fleas were embedded in OCT media, frozen and cryosectioned. Sections were labeled with anti-*R. typhi* rat immune serum (Alexa488-conjugated anti-rat secondary Ab – green), anti-serum to the sca protein indicated on the left (Alexa594-conjugated anti-rabbit secondary Ab – red) and mounted in VectaShield medium with DAPI (blue) to counterstain DNA. Arrowheads point to examples of positively stained rickettsiae. Bar = 5 µm. D) Spleens were harvested from 9 day infected female Sprague-Dawley rats and fixed and embedded as described. Sections (5 µm) were stained with AlexaFluor 532-labeled anti-*R. typhi* rat immune serum (red), AlexaFluor 350-labeled anti-serum to the sca protein indicated on the left (blue) and propidium iodide (green) to counterstain DNA (not shown). Sections were mounted in VectaShield medium. Arrowheads indicate examples of positively labeled rickettsiae.

We investigated possible differential transcription of *sca* genes during L929 cell infection. Of the five *sca* genes annotated in the *R. typhi* str. Wilmington genome, only *sca4* and *sca5* were known to be expressed at the transcript level before this study. All five *sca* gene transcripts were detected in total RNA extracted from L929 fibroblasts infected for 0–120 hours (Figure 3A). This complements data from a previous study in which *sca2*, *sca4* and *sca5* were identified in a genome-wide screen for temperature-shifted genes *in vitro* [38]. In general, a decrease in expression was observed within 1 h of infection and median expression levels near to or above initial infection were observed by 120 h (Figure 3A). Rickettsiae were actively growing over the course of the experiment (Figure 3B).

**Figure 4:**
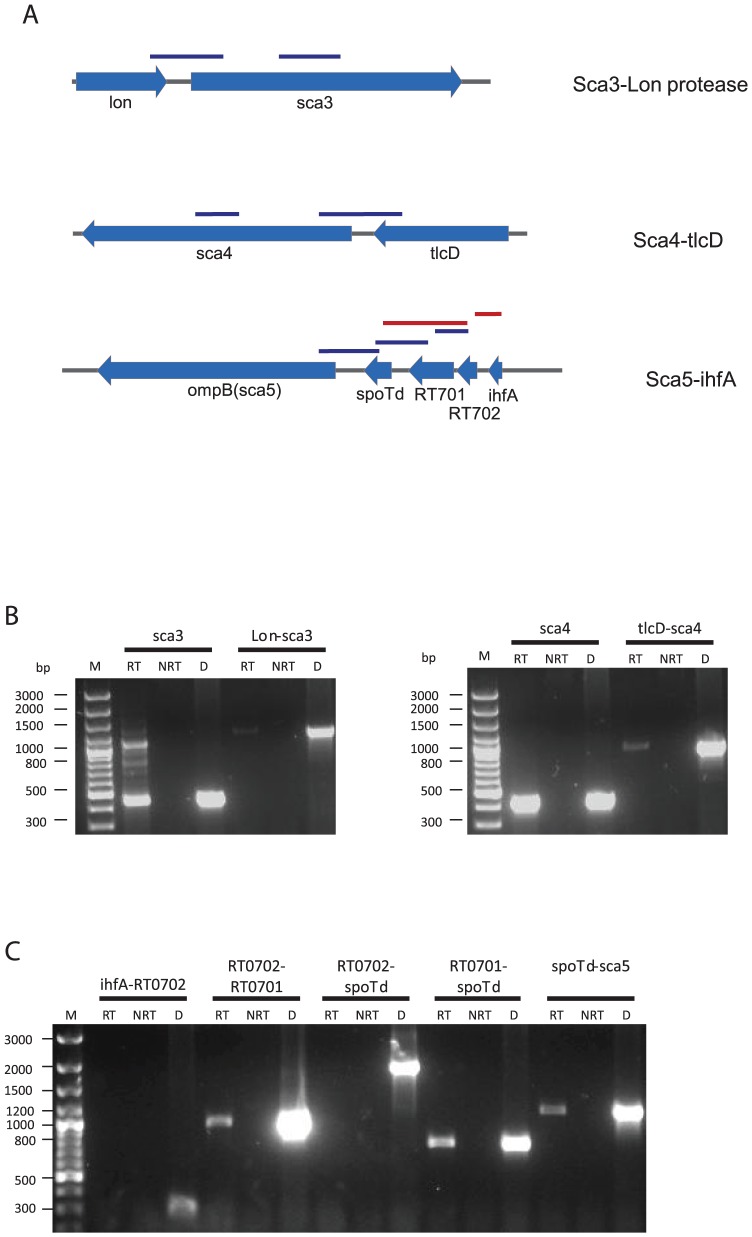
Transcriptional analysis of putative *sca* operons by RT-PCR. RNA was extracted from 72 h-infected L929 fibroblasts and RT-PCR with primers designed to amplify the regions indicated in (A). A) Schematic of putative *sca* operons. Blue bars indicate regions successfully amplified, red bars indicate regions that could not be amplified. B) RT-PCR amplification of regions between ORFs in putative *sca* operons. Left panel – lanes 2 thru 4 – RT-PCR amplification of the 400 bp region within the encoded passenger domain of *sca3* used in time course analyses ; lanes 5 thru 7 – amplification of *Lon-sca3* co-transcript. Right panel: lanes 2 thru 4 – amplification of *sca4* region used in time course analyses; lanes 5 thru 7 – amplification of *tlcD-sca4* transcript. C) Analysis of *sca5* operon; labels indicate genes spanned by primers. RT – reverse transcriptase reaction, NRT – no reverse transcriptase, D – DNA control.

Initial analysis using the OperonDB algorithm predicted that *sca3*, *sca4* and *sca5* genes had a probability of co-occurring in the same operon with a confidence of at least 73 (Table S4); however, analysis using the updated algorithm resulted in greatly reduced probabilities that any of these *sca* genes was located within an operon. However, *sca3*, *sca4*, and *sca5* are each positioned downstream of other open reading frames (Figure 4A). Therefore, we hypothesized that each gene cluster was an operon and that each *sca* was co-transcribed with the upstream genes.

We tested the above hypothesis, despite the low prediction probabilities, using RT-PCR to amplify the indicated regions (Figure 4A). Co-transcription of *Sca3* with the gene encoding the protease *Lon* and *Sca4* with one of four ATP/ADP translocases, *tlcD*, were detected (Figure 4B) indicating that both of these genes comprise a transcriptional unit with their nearby genes respectively. Co-transcription data for the putative *Sca5* operon is less conclusive. We were unable to detect a full transcript spanning the *ihfA* to *Sca5* genes (data not shown) and therefore designed primers to amplify 5 different regions that would span the entire putative operon. Transcripts of *Sca5*-*spoTd* and *spoTd-RT0701*, are detected suggesting that these 3 genes are co-transcribed. While an amplicon for *RT0701-RT0702* is detected, a transcript spanning *RT0702* and *spoTd* could not be amplified (Figure 4C). Multiple primer combinations were tested for the former combination without success.

To investigate Sca protein expression we generated a rabbit polyclonal antibody (pAb) against a peptide within the passenger domain of each Sca. Specificity of the serum directed to each Sca was confirmed by immunoblot against lysates from uninfected and infected L929 cells (Figure S2) and sections of infected and uninfected L929 cells (Figure S3 and S4). Sca5 staining is observed in the cytoplasm and the outer membrane on many rickettsiae (Figure S3). Some labeling of host cell chromatin was also observed. Although staining is weak, Sca 1 is observed in the rickettsial cytoplasm and in association with the membrane of rickettsiae. Staining is also observed on the outer membrane (Figure S3), the host cell cytoplasm and chromatin (data not shown). Sca2 and Sca3 staining is observed on the outer membrane, host chromatin or cytoplasm of very few rickettsiae (Figure S3). Sca4 staining shows a similar pattern to the other Scas with labeling of the outer membrane, periphery and rickettsial cytoplasm (Figure S3).

To investigate possible variability of Sca expression within organisms that maintain the infection in an enzootic cycle we infected rats and cat fleas. Rats serve as a primary reservoir for murine typhus in urban environments. Infection is self-limiting, however, splenic dissemination occurs a week into infection [39]. The cat flea, *C. felis*, is suspected of being one of the primary vectors of *R. typhi* transmission to small mammals and humans in the United States [40]. No staining was observed in sections from uninfected fleas or uninfected rat spleens (Figures S5 and S6). Scas3-5 are expressed in rickettsiae in various organs of 14-day infected fleas including the midgut and developing eggs (Figure 3C); staining for Scas 1 and 2 were not conclusive. Positive staining was distinctly detected for Scas1 and 5 by immunofluorescence staining of cryosections of spleens from 9-day infected Sprague Dawley rats (Figure 3D) indicating that they are expressed during mammalian infection. However, staining for Sca4 was diffuse and not clearly detected (data not shown).

### Sca protein expression on the surface of *R. typhi*

**Figure 5:**
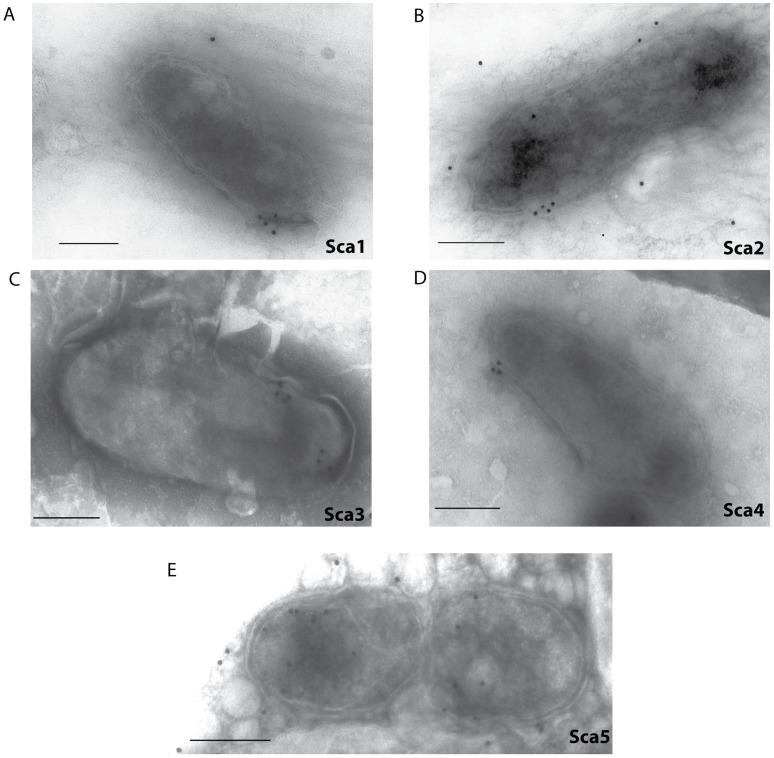
Immunogold electron microscopy of Sca expression on intact, negative stained *R. typhi*. A suspension of rickettsiae was placed on grids and allowed to dry. Samples were labeled with antibodies against a Sca protein A) Sca1, B) Sca2, C) Sca3, D) Sca4 and E) Sca5 and negatively stained for contrast. Bar = 0.25 um.

Expression of the Scas on whole rickettsiae was investigated using flow cytometry and negative stain IEM of whole rickettsiae. For flow cytometry, rickettsiae were stained with antibodies prior to fixation to avoid permeabilization so that only surface-bound Scas were detected. While the majority of rickettsiae only stained positive for the anti-*R. typhi* sera, a small proportion (1.58–3.82%) also stained positive for the respective Sca protein (Figure S7A). Non-specific staining of rickettsia and any co-purified host components was minimal (Figure S7B). To confirm surface expression of the Sca proteins, whole rickettsiae placed onto grids were labeled with anti-Sca sera followed by colloidal gold conjugated anti-rabbit IgG secondary antibody. Immunogold labeling for each Sca protein is observed on the surface of negatively stained, intact rickettsia with clusters of gold particles indicating positive staining (Figure 5).

## Discussion

Surface proteins of obligate intracellular bacteria comprise a crucial interface for pathogen-host interactions by mediating the initial attachment and infection of host cells and subsequent contact with host cytosolic proteins to promote bacterial survival and replication through the subversion of host processes. In this report, we investigated the surface proteome of *R. typhi* str. Wilmington, first by bioinformatic analyses to predict subcellular localizations and the presence of classical and non-classical secretion signals, then by selective labeling and purification of surface proteins for identification. We consequently focused on characterizing the Sca family of autotransporters.

Outer membrane proteins (OMPs) are immunodominant in rickettsial infections and immunization with these antigens has been shown to confer protection from lethal challenge in animal models [8], [9], [10], [11], [41]. Similarly, the Major Surface Proteins (MSPs) of *Anaplasma* spp. and the Outer Membrane Proteins (OMPs) of *Ehrlichia* spp. are also identified as immunogenic rickettsial proteins [31], [32], [42]. Rickettsial OMPs form the basis for antigenic relationships between and within phylogenetic groups and allow for some cross-protection from infection by multiple species. A major basis for the antigenic similarity of *Rickettsia* spp. is the presence of the complete gene for the 120 kDa outer membrane protein B (Sca5) in all species [15]. Bioinformatic analyses place Sca5 and the similar protein OmpA, which is not encoded in the genomes of TG *Rickettsia*, into the superfamily of proteins known as the type V secretion system (TVSS) or autotransporter family [43].

Many of the proteins identified on the surface of *R. typhi* (Table S3) have homologs that were similarly identified on the surface of other rickettsiae or other families of Gram-negative bacteria. These include the chaperone proteins GroEL and DnaK and enzymes pyruvate decarboxylase and fumarase (Table S3). Furthermore, proteins experimentally determined to be surface localized were identified in this study but many currently have no assigned function (*i.e.* hypothetical proteins) in the context of rickettsial infection. Homologs of proteins predicted to be cytoplasmic but experimentally localized to the surface and cytoplasm of other Gram-negative and Gram-positive bacteria [34], [36], [44] are detected on the surface of rickettsiae in this study [30], [31], [33]. Little is known about the significance of such proteins on the bacterial surface or the mechanism(s) by which they are targeted to the surface. However, it is becoming understood that such proteins have alternative functions when surface-exposed and have thus been termed moonlighting proteins [45] For example, when at the bacterial surface, the chaperone protein DnaK of *Mycobacterium tuberculosis* has been shown to bind plasminogen, stimulate chemokine synthesis in dendritic cells and compete with the human immunodeficiency virus (HIV) coreceptor chemokine receptor 5 (CCR5) [46], [47], [48]. Elongation factor-Tu (EF-Tu) and the E1 beta subunit of the pyruvate dehydrogenase (Pdh) of *M. pneumoniae* has been shown to bind fibronectin [49] and a fungal dihydrolipoamide dehydrogenase, the E3 subunit of Pdh, has been characterized as an acetyltransferase both when not in the cytoplasmic environment [50].

Few rickettsial surface-exposed proteins have been investigated; however, certain Sca proteins have been localized to the bacterial surface [23] or inferred to be located there based on antibody inhibition of infection or conference of adhesive and invasive properties to recombinant *E. coli* [19], [20], [51]. We were able to detect the recently characterized RT0522 conserved hypothetical protein, which encodes a phospholipase A_2_ homolog that was found to be secreted from the bacterium into the host cytosol [52]. Most of the algorithms used in this study detected no signal peptide in RT0522 and predicted it to be localized to the cytoplasm; however pSORTb v3.0 predicted it to be extracellular. This may be evidence that other hypothetical proteins identified on the surface but predicted by consensus to be cytoplasmic and lacking detectable signal peptides, may in fact have extracellular functions as effector proteins. It is also an indication that the algorithms, such as SubcellPredict, SLPLocal and SubLoc v.1 [53], [54], [55] (data not shown), which are trained on free-living model Gram-negative and Gram-positive bacteria, may not accurately identify the signals and motifs utilized by rickettsiae to target proteins to the surface. Discrepancies between the number of predicted outer membrane or extracellular proteins and the number actually identified may be due to low abundance of some peptides and or inaccessibility to the biotinylation reagent. It is predicted that Sca5 comprises the S-layer of rickettsiae and constitutes 15% of the total protein mass [56], [57] and might block many potential interactions. The properties of the labeling reagent must also be taken into account. The N-hydroxysulfosuccinimide (NHS) group of Sulfo-NHS-SS-biotin specifically reacts with the ε-amine of lysine residues and the reactions also proceed at a wide range of temperatures. A more comprehensive analysis of the surface proteome might be compiled by performing reactions across the range of temperatures and using similar biotinylated reagents with spacer arms of differing lengths and reactivity with different groups.

The Sca family of autotransporters drew our focus because their primary involvement in adherence and invasion is becoming more apparent but predicted functional domains within a few of them point to other roles [20], [51], [58]. Multi-functional autotransporters are not uncommon and further characterization of this family may define distinct extracellular and intracellular roles. For instance, *R. parkeri* Sca2 is observed to act as an actin-assembly mediator that mimics the eukaryotic formin proteins. Furthermore, it is the first bacterial protein to be identified to functionally and perhaps structurally mimic a domain that was previously thought to be solely eukaryotic [23]. Interestingly, this is the second instance of a rickettsial protein containing a domain with a nearly strict eukaryotic distribution. Similarly, the Sec7-domain-containing proteins (RalF) encoded within *Rickettsia* and *Legionella* species are unknown from other prokaryotes [59].

As stated above, all of the Scas in *R. typhi* were identified on the surface when considering the biotin labeling (Table S3), negative staining electron microscopy (Figure 5) and flow cytometry data collectively (Figure S7). All Sca proteins except Sca4 were predicted to have signal peptides and be localized to the outer membrane or be extracellular (Tables S1 and S2). The prediction of a signal peptide in Sca4 by SecretomeP supports evidence that the first 200 bp of *Sca4* encode sufficient information to direct secretion of a phoA fusion peptide across the inner membrane into the periplasm (unpublished data). Further, Sca4 is known to generate an antibody response to infection [60] and others have shown, as we have here, that Sca4 is under positive selection [15]. It has been posited that Sca4 and the similarly AT domain-less Sca9 may be exported through the AT domain of another Sca. However a quick analysis for possible signals based on homology among the Scas returned no supporting data for this hypothesis. Further investigation of the function and secretion mechanism of Sca4 is necessary.

**Figure 6:**
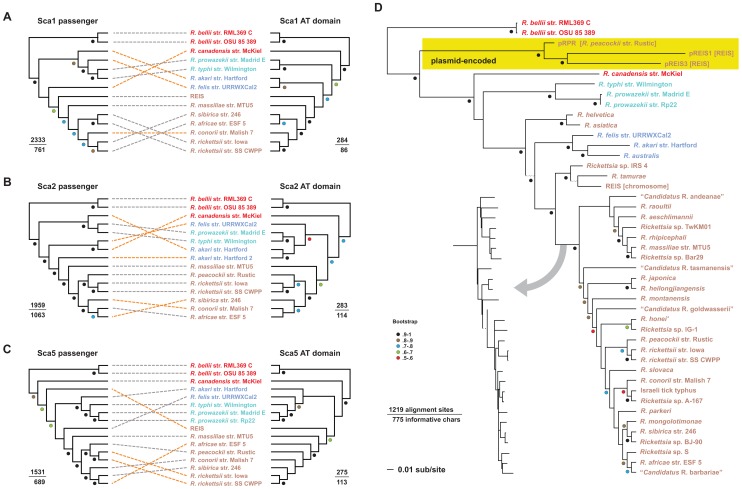
Evolution of rickettsial surface cell antigens (Scas). All datasets were aligned with MUSCLE v3.6 [74], [75] and analyzed under maximum likelihood using RAxML [76] (see text for details). Branch support was assessed with 1000 bootstrap pseudoreplications, with values in intervals of 10 (>50% only) depicted with colored circles that are explained at the bottom left. Taxon color scheme according to major rickettsia group is explained in the Figure 2 legend. (A–C) Comparison of the phylogenies estimated for the passenger (left) and AT domains (right) for (A) Sca1, (B) Sca2 and (C) Sca5. For each tree, numbers above the line depict the total number of positions for each domain alignment, with numbers under the line representing the total number of informative sites within the alignment. The phylogenies of the AT domain better corroborate the rickettsia species tree (see Figure 2), thus the position of the taxa correspond with these trees. The taxa in the trees generated from the passenger domain are connected to their corresponding partner in the AT domain trees via dashed lines, with orange illustrating the major differences between the domain-generated trees. (D) Phylogeny estimation of an expanded set of rickettsial Sca4 proteins. The yellow box encloses the only known plasmid-encoded Sca4 proteins: pREIS1 and pREIS3 from REIS, rRPR from *R. peacockii* [83]. The gray arrow illustrates the replacement of the phylogram portion of the tree with the corresponding cladogram to account for the minimal divergence across the majority of SFG Rickettsia Sca4 proteins.

Phylogeny estimation of Sca1, Sca2, Sca4, and Sca5 proteins resulted in trees that all differ from the *Rickettsia* species tree (Figure S1). This is not unexpected, as phylogenies based on single rickettsial genes and proteins rarely agree with those estimated from multiple molecules [61]. Notwithstanding, the Scas are large proteins and contain many variable sites, which should provide enough information for robust phylogeny estimation. However, the extracellular location of the passenger domains likely orchestrates selective pressures on these proteins that render them evolutionarily divergent from the species historical trajectory. Indeed, previous studies have identified positively selected sites within the Sca passenger domains [15], [62]. For Sca1, Sca2 and Sca5, we estimated separate trees based on the passenger domain and AT domain to determine conflicting phylogenetic signals within these domains (Figure 6A–C). In each case, the trees estimated for the AT domain were much more consistent with the rickettsial species tree (Figure 1B), while the trees generated from the passenger domains clearly illustrate Sca protein diversification inconsistent with rickettsial species phylogeny.

Recombination between Sca orthologs from different species has also been demonstrated to play a role in the diversification of these proteins [62]. While recombination has been observed experimentally in *Rickettsia* [63], [64], its contribution to rickettsial diversity is currently not fully understood. Notwithstanding, all rickettsia genomes encode enzymes involved in homologous recombination [65], so it is likely that active recombination occurs across species and strains. Insight into how recombination may shape Sca protein evolution is provided by a phylogeny estimation of an expanded set of Sca4 proteins (Figure 6D). Without an AT domain, contrasting phylogenetic signals within Sca4 proteins could not be determined as for the other Scas described above. However, the identification of an ancestral lineage of plasmid encoded Sca4 (Figure 6D, yellow box) proteins illustrates the inclusion of these proteins in the rickettsial mobilome (all the mobile genetic elements in the genome), allowing for the dissemination of Sca variants via lateral gene transfer (LGT). Other studies have reported the presence of Sca ORFs and fragments present on diverse rickettsial plasmids [66], [67] further supporting the role of LGT as a facilitator of mosaicism via recombination across divergent Sca orthologs (and possibly paralogs). Like the phylogenies estimated from the other Scas, the Sca4 tree does not corroborate the rickettsial species tree, and it is likely that all Scas are subject to positive selection and recombination. These selective forces, which are counter to the evolutionary history of rickettsia, are problematic for inferring species relatedness [62]. Thus, despite their usefulness as rickettsia-specific diagnostic markers, as well as their increasing accumulation from published studies, the Scas should be avoided for phylogenetic inference and classificatory purposes.

Transcriptional analysis in non-phagocytic cells demonstrates that *sca* transcription is sustained during infection (Figure 3A). Although we are unable to correlate *sca* transcription with protein levels at present, the constitutive expression of all *sca* genes points toward essential functions. Moreover, the co-transcriptional analyses (Figure 4) suggest that *scas* and the genes that are co-transcribed may function in the same processes or are part of regulatory mechanisms important for their expression. The inability to detect *ihfA-RT0702* and *RT0702-spoTd* transcripts while detecting *RT0702-RT0701* and *RT0701-spoTd* transcripts in the proposed *sca5-ihfA* operon indicates that the consecutive genes are not expressed at the same level. It is becoming understood that the concept of multiple successive genes under the control of a single promoter - that is operons - does not inherently mean that all genes are equally expressed [68]. This phenomenon, termed operon polarity, may be explained by the presence of internal transcription terminators, the activity of small RNAs (sRNAs) or by riboswitches within operons that modulate transcription in response to metabolite binding [69].

We attempted to detect all of the Sca proteins for *R. typhi* in the spleen tissue, however, in these experiments we consistently observe expression for Scas 1 and 5. This may be specific to the rat we used in this particular experiment or the time point we chose to assay (9 days). Given animal to animal variation as well as time-dependent expression of many rickettsial proteins, we do not exclude the possibility that the remaining Scas play an important role during mammalian infection. While we show that the proteins are expressed *in vivo* (Figure 3B–C), we are unable to quantitatively assess this expression and correlate it with different stages of growth or infection. However, the immunogold labeling of host cell cytoplasm and chromatin may suggest translocation of the rickettsial proteins to the cytosol. Probing infected cells with pre-immune sera or uninfected cells and tissues with Sca immune sera show no cross-reactivity with host cell components. Proteins may be expressed but not required for infection of a particular host or cell type. Rather, the bacteria will be prepared, upon exiting a cell, to infect an endothelial cell or the flea midgut epithelium. Further characterization of these proteins and identification of interacting host proteins will determine where and how these proteins function and their importance in infection of mammalian and arthropod hosts.

## Materials and Methods

### Ethics statement

This study was carried out in strict accordance with the recommendations in the Guide for the Care and Use of Laboratory Animals of the National Institutes of Health. The protocol was approved by the Institutional Animal Care and Use Committee (IACUC protocol no. 1108009) of the University of Maryland, Baltimore (assurance number A3200-01).

### Growth, maintenance and isolation of *R. typhi*

Rickettsiae were grown and maintained as previously described [38]. Low passage mouse fibroblast cells (L929, ATCC CCL1, ATCC, Manassas, VA) were grown in Dulbecco's Modified Eagle's Medium (DMEM) supplemented with 5% FBS at 37°C and 5% CO_2_ in 150 cm^2^ vented lid flasks. When the cells were 80% confluent they were infected with *R. typhi* str. Wilmington at a multiplicity of infection (MOI) of 10. To harvest rickettsiae, infected L929 cells were scraped into the media then sonicated at setting 6.5 twice for 30 seconds using a Sonic Dismembranator (Fisher Scientific, Pittsburgh, PA). The lysates were centrifuged at 1000× g for 5 min to remove large host cell material. The supernatant was centrifuged at 14,000× g for 10 min and the pellets, containing rickettsiae, resuspended in 1 mL SPG buffer (218 mM sucrose, 3.76 mM KH_2_PO_4_, 7.1 mM K_2_HPO_4_, 4.9 mM potassium glutamate). The rickettsiae were placed over a 20% OptiPrep Density Gradient medium:SPG bed (Sigma-Aldrich, St. Louis, MO) and centrifuged at 14,000× g for 10 min. The pellets were washed twice in SPG buffer and centrifuged at 14,000× g; rickettsiae were quantified using the BacLight Live/Dead assay (Molecular Probes, Eugene, OR) as per the manufacturer's instructions and stored at −80°C. For flow cytometry, infected host cells were scraped into the media, centrifuged at 14,000× g for 10 min then resuspended in 5 ml SPG buffer. Suspended host cells were ruptured by passage through a 27-gauge needle three times to disrupt host cell membranes while maintaining rickettsial membrane integrity and lysates were centrifuged at 1000× g for 5 min. Supernatants were placed over a 20% OptiPrep:SPG bed and centrifuged as above. Bacterial pellets were resuspended in 100 µl SPG.

### Biotinylation of surface proteins and NeutrAvidin affinity purification of labeled proteins

Sub-confluent monolayers of L929 fibroblasts were grown in 150 cm^2^ vented-lid flasks and infected with renografin-purified rickettsiae at an MOI of 10 for 48 h when most cells are heavily infected. Two flasks were used for each treatment. Rickettsiae were partially purified as described above, washed three times in ice-cold PBS (pH 8.0) and pelleted using centrifugation at 8,000× g for 3 min at 4°C. Pellets were resuspended in 960 µl of PBS with 80 µl of 10 mM EZ-Link Sulfo-NHS-SS-Biotin (Pierce Thermo Scientific, Rockford, IL) and incubated on ice for 30 min. As a negative control, rickettsiae were resuspended in PBS only to assess background affinity purification. As a control for background host material, a mock partial purification was performed on uninfected L929s, and the resulting pellets were labeled as described above. Free biotin was quenched by washing rickettsiae once in 50 mM Tris-HCl (pH 7.5–8.0) followed by two washes in ice-cold PBS and resuspended in RIPA buffer (25 mM Tris-HCl [pH 7.6], 150 mM NaCl, 1% NP-40, 1% sodium deoxycholate, 0.1% sodium dodecyl sulfate) supplemented with 1X Halt Protease Inhibitors (Pierce Thermo Scientific). Rickettsiae were lysed by bead beating with ≤106 µm glass beads (Sigma-Aldrich, St. Louis, MO). Beads were removed by centrifugation at 500× g for 3 min and cell debris pelleted by centrifugation at 16,000× g for 10 min. Supernatants were stored at −80°C until needed.

Labeled proteins were purified by affinity purification over columns containing NeutrAvidin Agarose resin (Pierce Thermo Scientific) as previously described [42] with modifications. Briefly, columns were equilibrated with three column volumes of wash buffer A (25 mM Tris-HCl pH 7.6, 0.15 M NaCl, 0.5% NP-40, 0.5% sodium deoxycholate, 0.05% SDS). Biotinylated samples were allowed to enter the resin bed and incubated at room temperature for 10 min. Unbound proteins were washed away with two column volumes of buffer B-1 (25 mM Tris-HCl pH 7.6, 0.65 M NaCl, 0.1% NP-40), then one volume of buffer B-2 (25 mM Tris-HCl pH 7.6, 1.15 M NaCl, 0.1% NP-40) followed by one volume of Tris-HCl buffer (25 mM Tris-HCl, 0.15 M NaCl). Washes were collected in 0.5 ml fractions and stored on ice. Captured proteins were eluted with two volumes of 5% β-mercaptoethanol-PBS. The eluates were pooled and total protein determined using a BCA assay (Pierce Thermo Scientific). Eluates were concentrated by filtration using Amicon-Ultra prior to addition of 2X SDS sample buffer (Invitrogen, Carlsbad, CA) then stored at −80°C.

### Proteomic and bioinformatic analyses

NeutraAvidin (Pierce Thermo Scientific) affinity purified proteins were separated on 4–20% Tris-glycine gels (Invitrogen) and stained for total protein visualization using PS-Blue (BridgePath Scientific, Frederick, MD). Proteins from several regions were identified using LC-MS/MS at the University of Maryland Baltimore Proteomics Core Facility as described fully in the supplementary methods. Briefly, Coomassie-stained protein bands were excised, dehydrated, digested and de-salted in preparation for LC MS/MS. MS/MS spectra were searched against a uniprot mouse database (uniprot release number 2010_05; 64,389 sequences) and a *Rickettsia typhi* database (uniprot release number 2010_05; 1,676 sequences) using Sorcerer-SEQUEST (SageN Research, Milpitas, CA). The quality of peptide and protein assignments was assessed using PeptideProphet and ProteinProphet. Proteins with probabilities ≥0.9 were accepted as true positive identifications. Proteins identified by one unique peptide were manually verified. Identified proteins were then analyzed for signal sequences and motifs that predict subcellular localization. Signal peptides were detected using the SignalP 3.0 and LipoP 1.0 servers which predict the presence and location of signal peptide cleavage sites in peptide sequences [70], [71]. SignalP predictions are based on a combination of artificial neural networks (NN) and hidden Markov models (HMM) while LipoP predictions distinguish between lipoprotein signal peptides, other signal peptides and N-terminal membrane helices. The Phobius server [72] was also utilized primarily for the prediction of signal peptides in an amino acid sequence. To predict subcellular localization predictions for peptides identified on the surface, we employed the pSORTb v3.0.2 server and the predictive program SOSUI-GramN [25], [26], [27]. Pre-computed genome results were downloaded from the pSORTb server, which uses several analytical methods for predicting a final localization including prediction of extracellular proteins. The *R. typhi* predicted proteome was submitted to the SOSUI-GramN software which uses only physicochemical factors of the total sequence and the N- and C-terminal signal sequence to predict protein localizations in Gram-negative bacteria. The SecretomeP 2.0 server [29] was utilized to generate predictions of protein secretion not initiated by signal peptides (i.e. non-classically secreted proteins). This algorithm also integrates information on post-translational and localization aspects of the protein from other feature prediction servers. CoBaltDB [24], a comprehensive database that compiles prediction outputs from multiple sources regarding complete prokaryotic proteomes, was also utilized for comparison of subcellular localization predictions.

### Comparative analysis of rickettsial Sca proteins

Orthologs of the five Scas encoded with the *R. typhi* genome were extracted from the PATRIC web site [73]. Initially, only protein sequences from 16 completely sequenced rickettsia genomes were included: *R. bellii* str. RML369-C; *R. bellii* str. OSU 85 389; *R. canadensis* str. McKiel; *R. typhi* str. Wilmington; *R. prowazekii* str. Madrid E; *R. prowazekii* str. Rp22; *R. felis* str. URRWXCal2; *R. akari* str. Hartford; *Rickettsia* endosymbiont of *Ixodes scapularis* (REIS); *R. massilae* str. MTU5; *R. peacockii* str. Rustic; *R. rickettsii* str. Sheila Smith; *R. rickettsii* str. Iowa; *R. conorii* str. Malish 7; *R. sibirica* str. 246; *R. africae* str. ESF-5. Except for split ORFs in *R. prowazekii* genomes, pseudogenes (as detected using tblastn searches across all genomes) were not included in the analyses. Sequences for each Sca orthologous group were aligned using MUSCLE v3.6 [74], [75] with default parameters (full annotated alignments are available in Supplement 1). Phylogenies were estimated for Sca1, Sca2, Sca4, and Sca5 under maximum likelihood using RAxML [76] (Figure S2). A gamma model of rate heterogeneity was used with estimation of the proportion of invariable sites. Branch support was evaluated from 1000 bootstrap pseudoreplications. For Sca1, Sca2, Sca3, and Sca5, the alignments were divided into the passenger domain and AT domain. Phylogenies of each domain from Sca1, Sca2 and Sca5 were estimated. For Sca4, sequences from additional rickettsial species (and plasmids) were included in a larger analysis, with alignment and phylogeny estimation as described above. Repeat regions within Sca4 and the passenger domains of Sca1, Sca2, Sca3, and Sca5 were predicted using HHrepID [77].

### L929 cell infection time course, RNA extraction and cDNA synthesis

Sub-confluent monolayers of L929 cells in 6-well plates were infected with an MOI of 10 *R. typhi* str. Wilmington for 0, 5, 15 or 30 min and 1, 8, 24, 48 and 120 h. Rickettsiae-infected cells were washed briefly with cold PBS and immediately disrupted with the lysis buffer, Buffer RLT, the first step in the total RNA extraction procedure using the AllPrep DNA/RNA kit (Qiagen, Valencia, CA). Total RNA was DNase-treated with the RNase-Free DNase Set (Qiagen) then cleaned up and concentrated with the RNeasy MinElute Cleanup kit (Qiagen). DNA removal was confirmed using the SuperScriptIII One-step RT-PCR with Platinum *Taq* kit (Invitrogen, Carlsbad, CA) and cDNA was made using the SuperScriptIII First-Strand Synthesis SuperMix for qRT-PCR kit (Invitrogen).

### Real-time quantitative PCR

Expression of the *Sca* genes was analyzed as previously described [78] with some modifications. Briefly, gene expression was detected using LUX primers (Invitrogen) in a multiplex format. Primers for *rpsL*, *GAPDH* and one of the *Sca* genes were included in each PCR reaction for amplification using the Platinum Quantitative PCR SuperMix-UDG kit (Invitrogen). Reactions were performed in duplicate on a MX3005P Stratagene real-time thermal cycler. Primer pairs were designed using the D-LUX Designer (Invitrogen) and chosen based on a melting temperature of 55°C and optimal amplicon size of 90–200 bp. Primer pairs used in this study are listed in Table 1. Cycling conditions were as follows: one cycle of 50°C for 1 h; one cycle of 95°C for 10 min; and 40 cycles of 95°C for 30 s, 60°C for 1 min, and 72°C for 30 s followed by a disassociation cycle of 95°C for 1 min, a 30-s hold at 55°C, and a ramp up at 0.1°C/s to 95°C for a 30-s hold. Data were imported and analyzed as described in Ceraul *et al* 2007. Cycle thresholds above 45 were excluded from analysis and the results from three experiments were combined and the median normalized expression values were calculated.

**Table 1:** Primers designed for real-time quantitative PCR analysis of *sca* genes, *rpsL* and mouse *GAPDH* expression during *R. typhi* infection of mouse fibroblast cells and co-transcription of genes in putative *sca* operons.

	Target/primer name	Sequence	Direction	Label
*Real-time qRT-PCR analysis of infection time course in L929 cells*				
	rpsL/AZ5250	AGGTTCCTGACCTTCCTGGTGT	FWD	None
	RT rpsL_340RL†	CGTTCGTGGCACCATAACGTGAAcG	REV	JOE
	mGAPDH/GAPDH-mouse_204FL†	CGTGACCAGTATGACTCCACTCAcG	FWD	FAM
	mGAPDH/AZ5987	CTGGAAGATGGTGATGGGCTTC	REV	None
	sca1/AZ5915	AGGTATGGGAAGTCACGAGATGG	FWD	None
	sca1/R. typhi sca1_3078RL*	cggaaGACTTTAGTACCTAAAATTCcG	REV	Alexa594
	sca2/R. typhi sca2_1827FL*	cggtgGGCAGAATTATTTATCACcG	FWD	Alexa594
	sca2/AZ5918	GAGCGGAAGTGCCCAGATTT	REV	None
	sca3/AZ5920	TAGGACAAATAGGAACGCCACAAA	FWD	None
	sca3/R. typhi sca3_3123RL* †	cggaaTAATACCACCTCCAGTTCcG	REV	Alexa594
	sca4/R. typhi sca4_703FL* †	cggcCCTATAAAACTTGATAAAGCcG	FWD	Alexa594
	sca4/AZ5922	GTGGTTTACCATTTGGCCCTTC	REV	None
	sca5/R. typhi sca5_751FL* †	cggcTAATACTACTCCTGATGCcG	FWD	Alexa594
	sca5/AZ5924	AATTTACCAGTACCGTCTCTTCCA	REV	None
*Co-transcription RT-PCR analyses*				
	Lon-sca3 R3/AZ6423	TGGCTGTGCTTTGTTTACTACTATT	FWD	N/A
	Lon-sca3 R3/AZ6424	TTTGACTTACTTCAACACCACCAAA	REV	N/A
	sca4-tlcD/AZ6502	GCGATAACAATAGGTGCTATTCAA	FWD	N/A
	sca4-tlcD/AZ6503	ATCTTGATTACTCAAAAAAGCACGG	REV	N/A
	Sca5-spoTd/AZ6504	GCTTTGGATGTAGTTAAGAATTCTC	FWD	N/A
	Sca5-spoTd/AZ6505	AGCAGTAATAACCGAATTTGTAGC	REV	N/A
	spoTd-RT0701/AZ6506	TCCTGCGACTTTATCCAAAA	FWD	N/A
	spoTd-RT0701/AZ6507	AACTATAATCCTACATGCGATCAAG	REV	N/A
	RT0702-spoTd/AZ7162	ACATAGATCTTTAGATTTAACTATAATC	FWD	N/A
	RT0702-spoTd/AZ7163	ATGACAAACAATAAGCTCACAAA	REV	N/A
	RT0702-RT0701/AZ7164	AGTTACTTTCTCTTCTTGTAACC	FWD	N/A
	RT0702-RT0701/AZ7165	ATGACAAACAATAAGCTCAC	REV	N/A
	RT0701-ihfA/AZ6510	ATGACTATTACAAAAAACAAAA	FWD	N/A

* The lowercase letters on the 5′ end are included for hairpin formation.

† The 3′ lowercase letter is the labeled base.

### Multiple antigen peptide synthesis, antibody production, purification and immunoblotting

Polyclonal antibodies were raised against predicted immunogenic peptides from each of the Sca proteins. Complete protein sequences for Sca proteins 1–4 (accession numbers YP_066986, YP_067021, YP_067397 and YP_067439 respectively) were submitted to Custom Peptide/Antibody Services (Invitrogen) for peptide design based on antigenicity, residue accessibility and hydrophilicity predictions. The following peptide sequences were chosen for use in the custom antibody PolyQuik rabbit protocol (Invitrogen): Sca1 - _753_ NYNKGEKNYDSDFK_767_ (Sca1_753–767_), Sca2 – _496_ LNNQNVQDENNKEW_509_ (Sca2_496–509_), Sca3 – _314_ IKGINNEEERLNLK_327_ (Sca3_314–327_), Sca4 – _263_ HYEEGPNGKPQLKE_276_ (Sca4_263–276_). All peptides were within the predicted passenger domains of the proteins and were conjugated to form multiple antigen peptides (MAPs) for enhanced immunogenicity. For Sca5, the peptide sequence _651_ NDGSVHLTHNTYLI_665_ (Sca5_651–665_) was chosen based on the immunogenicity of the *R. prowazekii* and *R. conorii* Sca5 homologs [79], [80]. The Sca5 peptide was also conjugated to form a MAP and used in the Premium Rabbit Protocol (Invitrogen). For all downstream applications, polyclonal rabbit and *R. typhi*-immune rat sera were purified using the Melon Gel IgG Spin Purification Kit (Pierce Thermo Scientific). The purified sera were eluted at a 10-fold dilution and subsequent dilutions are given with respect to undiluted sera.

For immunoblots to determine antibody specificity, pellets of uninfected or *R. typhi* infected L929 cells were solubilized in 2X LDS loading buffer (Invitrogen) with a reducing agent and boiled for 10 min at 100°C. Samples were run on NuPAGE 4–12% Bis-Tris gels in MOPS buffer or, when blotting for Sca3, 3–8% Tris-Acetate gels in Tris-Acetate buffer (Invitrogen) then transferred to PVDF membranes which were processed following a standard immunoblotting protocol. Membranes were probed with anti-Sca sera diluted 1∶250 and developed with SuperSignal West Pico Chemiluminescent Substrate (Pierce Thermo Scientific).

### Rat infections

Infections of 6-week old female laboratory white rats, *Rattus norvegicus* Sprague-Dawley (Charles River Laboratories Inc., Wilmington, MA) were carried out under BSL3 conditions. Rats received intradermal injections of 0.5 mL DMEM supplemented with 15% FBS containing 1×10^3^ *R. typhi* at the base of the tail. Control rats were injected with media only. Rats were euthanized on day 9 and organs were harvested, cut into pieces (≈0.5 cm^3^) and placed in fixation buffer [0.05 M phosphate buffer, 0.1 M lysine, 2 mg/mL sodium periodate, 1% paraformaldehyde (PFA)] overnight. The fixation buffer was replaced with a solution of 10% sucrose in phosphate buffer and incubated for 30 minutes at 4°C with occasional shaking; this step was repeated with 20 and 30% sucrose solutions. Tissue samples were embedded in Tissue-Tek Optimal Cutting Temperature (OCT) Compound (Sakura Finetek USA, Inc., Torrance, CA), frozen in a liquid nitrogen/isopentane bath and stored at −80°C. Sections (2–3 µm) were prepared using a Leica CM1900 Cryostat (Leica Microsystems Inc., Bannockburn, IL) and stained as described below.

### Flea infections

Infections of membrane-adapted *C. felis* (HESKA Corp., Loveland, CO) fleas were performed in a feeding apparatus under BSL3 conditions. Fifty fleas were placed into membrane feeding capsules and provided either 4 mL of uninfected or infected whole sheep's blood in a feeding reservoir. For an infection, renografin-purified *R. typhi* was added to blood for a concentration of 2.5×10^5^ rickettsiae per mL. On day 3, uninfected blood was added to the existing blood in all of the feeding reservoirs. Fleas were harvested at days 3, 5, 10 and 14 post-infection and placed in either 4% PFA or 4F1G fixative (4% PFA, 1% glutaraldehyde, 0.1 M PIPES, 0.1 M sucrose, 2 mM CaCl_2_) for electron microscopy overnight at 4°C. Fleas fixed in 4% PFA were embedded in Tissue-Tek OCT Compound and frozen at −80°C prior to sectioning; 3–5 µm sections were prepared using a Leica CM1900 Cryostat.

### Flow cytometry analysis of Sca surface expression

OptiPrep-purified rickettsiae were incubated with mixtures of anti-*R. typhi* rat immune serum (1∶250) and anti-Sca rabbit serum (1∶50) with end over end mixing for 1 hour. Controls containing no primary antibodies, anti-*R. typhi* serum only or pre-immune rabbit serum only were also prepared. Bacteria were washed twice with 200 µl PBS then resuspended in the appropriate secondary antibody mixtures (Alexa Fluor 488-conjugated donkey anti-rat 1∶500, Alexa Fluor 647-conjugated donkey anti-rabbit 1∶500, Alexa Fluor 647-conjugated donkey anti-rat 1∶500) and incubated for 30 min with mixing. Bacteria were washed and resuspended in 100 ul of 4% paraformaldehyde in PBS and incubated for 20 min with mixing then washed again prior to being resuspended in 500 µl PBS for flow cytometry analysis. Samples were analyzed on a BD FACSCanto II instrument (BD Biosciences, San Jose, CA) using the 488 nm (to detect A488-conjugated anti-R. typhi immune serum staining) and 633 nm (to detect A647-conjugated anti-Sca antibody staining) lasers. Rickettsiae stained with anti-*R. typhi* serum and either A488-conjugated anti-rat or A647-conjugated anti-rat secondary antibodies served as positively stained controls. Flow cytometry analyses were performed at the University of Maryland Greenbaum Cancer Center Shared Flow Cytometry Facility.

### Immuno-electron microscopy of L929 cells and purified rickettsiae

For electron microscopy, 48 h infected L929 cells were washed three times with PBS then fixed for at least one hour in PFGPA.1 fixative (2.5% formaldehyde, 0.1% glutaraldehyde, 0.03% picric acid (trinitrophenol), 0.03% CaCl_2_, 0.05 M cacodylate buffer pH 7.3–7.4). After washing in 0.1 M cacodylate buffer cells were scraped off the plastic, pelleted and processed as previously described [81]. Briefly, the pellets were stained *en bloc* with 2% aqueous uranyl acetate, dehydrated in 50% then 75% ethanol and embedded in LR White resin medium grade (Structure Probe, West Chester, PA). Ultrathin sections were cut on a Leica Reichert Ultracut S ultramicrotome and collected onto Formvar-carbon coated nickel grids (Electron Microscoy Sciences [EMS], Hatfield, PA). The grids were incubated in a wet chamber sequentially on drops of blocking buffer (0.1% BSA and 0.01 M glycine in 0.05 M Tris-buffered saline [TBS]), then on primary antibody with appropriate dilution in 1% BSA in 0.05 M TBS (diluting buffer) for 1 hr at room temperature and then overnight at 4°C. Primary antibodies were used at a 1∶50 dilution. After washing in blocking buffer, grids were incubated with a goat anti-rabbit IgG secondary antibody conjugated to 15 nm colloidal gold particles (Aurion, EMS), diluted 1∶20 in diluting buffer for 1 hr at room temperature. After washing in TBS and distilled water grids were fixed in 2% aqueous glutaraldehyde, washed, stained with uranyl acetate and lead citrate and examined in a Philips 201 or Philips CM-100 Electron microscope at 60 kV.

Surface labeling of whole rickettsiae for imaging by immuno-electron microscopy was performed as follows. 10^7^ purified rickettsiae were washed in 1X PBS and suspended in 4F1G fixative for 15 min. Rickettsiae were washed and resuspended in 10 mM HEPES; 20 µl drops were placed on Formvar-carbon coated nickel grids. Samples were blocked with 5% BSA, 0.1% CWFS (cold water fish skin) gelatin in HEPES for 15 min at room temperature. Antiserum to each Sca was diluted in 1% BSA, 0.1% CWFS gelatin diluting buffer for 1 hour at room temperature and then 4°C overnight. Grids were washed three times with 1X HEPES followed by incubation with goat anti-rabbit IgG secondary antibody conjugated to 15 nm colloidal gold particles (Aurion), diluted 1∶20 in diluting buffer for 1 hr at room temperature. Samples were fixed with 1% paraformaldehyde for 5 min at room temperature then washed three times with ddH_2_O for 5 min per wash. Finally, rickettsiae were negatively stained by incubation with 1% ammonium molybdate for 15 min at room temperature. Grids were viewed as noted above.

### Immunofluorescence assays of rat spleen and *C. felis*

Staining was performed at biosafety level 2. For staining of rat tissues, antisera were directly labeled with either an Alexa Fluor 350 dye (Sca antisera) or Alexa Fluor 532 dye (anti-*R. typhi* serum) (Molecular Probes). Prior to staining, rat sections were treated with a 100 µg/ml solution of DNase-free RNase (Roche, Indianapolis, IN) in 2X SSC (0.3 M NaCl, 0.03 M sodium citrate, pH 7.0) for 20 minutes at 37°C. Sections were washed briefly three times with 2X SSC, blocked with 5% BSA-2X SSC for 15 min then incubated with Alexa Fluor 350-conjugated rabbit anti-Sca antibody and Alexa Fluor 532-conjugated rat immune serum to whole *R. typhi* diluted 1∶100 and 1∶250 respectively in 2X SSC. Slides were placed in a humidifying chamber for 30 min at 37°C. Slides were mounted with VectaShield fluorescent mounting medium (Vector Laboratories, Burlingame, CA) for observation. For flea sections, the samples were blocked with 5% BSA-PBS then sequentially incubated with an anti-Sca serum followed by rat immune serum to whole *R. typhi*. Positive staining was assessed using Alexa Fluor 594 donkey anti-rabbit IgG and Alexa Fluor 488 donkey anti-rat IgG secondary antibodies (Molecular Probes) each diluted 1∶500 in 1% BSA-PBS for 30 min at 37°C. Slides were mounted with VectaShield fluorescent mounting medium with DAPI (Vector Laboratories).

### Accession numbers not listed in the respective figure legends are as follows

YP_067439: Rickettsia typhi str. Wilmington; ADE30028: Rickettsia prowazekii Rp22, AF163010_1: Rickettsia sp. IRS 4; AF155056_1: Rickettsia sp. Bar29; AAZ83584: Rickettsia asiatica; YP_001499380: Rickettsia massiliae MTU5; AAZ95593: Rickettsia tamurae; Q9AJ81: Rickettsia rhipicephali; Q9AJ79: Rickettsia japonica YH; YP_002916099: Rickettsia peacockii str. Rustic; AEK74699: Rickettsia heilongjiangensis 054; ABQ02467: Rickettsia sp. IG-1; AF163004_1: Rickettsia honei; ABD34821: Rickettsia raoultii; ACT33310: Candidatus Rickettsia tasmanensis; AF163009_1: Rickettsia Helvetica; ADH15759: Rickettsia aeschlimannii; NP_360304: Rickettsia conorii str. Malish 7; YP_002845259: Rickettsia africae ESF-5; ABQ02470: Rickettsia sp. TwKM01; ZP_00141907: Rickettsia sibirica 246; Q9AJ80: Rickettsia slovaca;AF163007_1: Rickettsia sp. A-167; YP_001650046: Rickettsia rickettsii str. Iowa; YP_001494783: Rickettsia rickettsii str. ‘Sheila Smith’; AF155058_1: Israeli tick typhus rickettsia; Q9AJ75: Rickettsia parkeri; AF163002_1: Rickettsia montanensis; AAZ78251: Rickettsia mongolotimonae; AF163001_1: Rickettsia sp. S; AAP92486: Rickettsia sp. BJ-90; YP_246741: Rickettsia felis URRWXCal2; YP_001493505: Rickettsia akari str. Hartford; Q9AJ64: Rickettsia australis; ACF20370: Candidatus Rickettsia barbariae; ADD12071: Candidatus Rickettsia andeanae; YP_001492306: Rickettsia canadensis str. McKiel; ADV19198: Candidatus Rickettsia goldwasserii; ZP_04699447: Rickettsia endosymbiont of Ixodes scapularis; YP_537939: Rickettsia bellii RML369-C; YP_001496362: Rickettsia bellii OSU 85–389; NP_220875, NP_220874: Rickettsia prowazekii str. Madrid E; ZP_04698207: Rickettsia endosymbiont of Ixodes scapularis; YP_002922015: Rickettsia peacockii str. Rustic; ZP_04698322: Rickettsia endosymbiont of Ixodes scapularis.

The content is solely the responsibility of the authors and does not necessarily represent the official views of the National Institute of Allergy and Infectious Diseases or the National Institutes of Health.

## Supporting Information

Figure S1 **Phylogenetic analysis of rickettsial surface cell antigens (Scas).** For each analysis, sequences were aligned using MUSCLE v3.6 [1], [2] (default parameters). Phylogenetic trees were estimated in PAUP* v4.0b10 (Altivec) under parsimony [7], implementing 500 random sequence additions with 100 trees saved per replication. Branch support was assessed via 1000 pseudoreplications. GenBank accession numbers are provided for each sequence. (A–D) Phylogenies estimated for Sca sequences encoded within 16 *Rickettsia* genomes. (A) Sca1. (B) Sca2. (C) Sca4. (D) Sca5. (E–H). Phylogenies estimated for an expanded set of Sca sequences containing the majority of the passenger and AT domain. (E) Sca1. (F) Sca2. (G) Sca4. (H) Sca5.
(EPS)

Figure S2 **Immunoblotting of Sca proteins in uninfected and infected L929 cell fractions.** L929 cells were infected with *R. typhi* for 4 days as described or left uninfected. Cells were washed and harvested for partial purification of rickettsiae and the resulting fractions separated by SDS-PAGE and processed for immunoblotting. Antiserum to each Sca (indicated above each blot) was used for probing. M – Marker, L – Uninfected L929 cells, RT – partially purified *R. typhi*. Arrows indicate bands corresponding to the predicted size range of the Sca protein blotted for.
(EPS)

Figure S3 **Immunogold post-embedding electron microscopy of Sca expression in** ***Rickettsia typhi*** **grown in L929 cells.** Bar = 0.5 um. A) Sca5/ompB is localized in the host cell cytoplasm and in the outer membrane of rickettsiae (arrows); B) Sca1 displays weak labeling in the host cell cytoplasm and in the outer membrane of rickettsiae (arrows); C) Sca2 labeling at the outer membrane (arrow); D) Sca3 labeling mostly at the rickettsial outer membrane (arrows); E) Sca4 labeling in the rickettsial cytoplasm, at its periphery and on the outer membrane (arrows).
(TIF)

Figure S4 **Immunogold post-embedding electron microscopy of Sca expression in uninfected L929 cells.** Bar = 0.25 um. Uninfected L929 cells were left uninfected and processed for immunogold electron microscopy as described in the methods. Sections were labeled with antibodies against the Sca protein indicated in the black box within each image.
(EPS)

Figure S5 **Immunofluorescence assays of Sca expression in uninfected** ***C. felis*** **.** Cat fleas were housed in an artificial feeding unit and fed uninfected whole sheep's blood for 14 days. Capsules were placed at −20°C to immobilize fleas before placing them in 3% PFA overnight. Fleas were embedded in OCT medium, frozen and cryosectioned. Sections were labeled with anti-*R. typhi* rat immune serum (Alexa488-labeled anti-rat secondary Ab – green), anti-serum to the Sca protein indicated on the left (Alexa594-labeled anti-rabbit secondary Ab – red) and mounted in VectaShield medium with DAPI (blue) to counterstain DNA.
(TIF)

Figure S6 **Immunofluorescence assays of Sca expression in uninfected rats.** Spleens were harvested from 9 day uninfected female Sprague-Dawley rats and fixed and embedded as described. Sections were labeled with anti-*R. typhi* rat immune serum (Alexa532-conjugated - red), anti-serum to the Sca protein indicated on the left (Alexa350-conjugated - blue) and stained with propidium iodide (green) to counterstain DNA then mounted in VectaShield medium.
(TIF)

Figure S7 **Flow cytometry assays.** A) Rickettsiae were gently purified and stained with anti-*R. typhi* immune sera and either anti-rat AlexaFluor 488 or AlexaFluor 647-conjugated anti-rat secondary Ab to determine the parameters for positive staining. B) Rickettsiae were gently purified and stained with anti-*R. typhi* immune serum (A488-conjugated anti-rat secondary Ab) and anti-Sca sera (A647-conjugated anti-rabbit secondary Ab) and analyzed by flow cytometry. Rickettsiae stained with both labels appear in the upper right quadrant. Each treatment was analyzed in triplicate and the data shown is representative of three experiments. C) Uninfected cells were taken through the purification process and the resulting pellet stained as indicated to assess the background contribution of host components to the analyses.
(TIF)

Table S1 **Signal peptide and subcellular localization predictions for classically secreted, membrane and surface-exposed proteins.** Each ORF in the *R. typhi* predicted proteome was analyzed for signal peptides using SignalP 3.0, LipoP 1.0 and Phobius servers. Subcellular localization predictions were identified using pre-compute genome results from the pSORTb v3.0.2 server and the predictive program SOSUI-GramN.
(XLS)

Table S2 **Proteins predicted to be secreted by only the SecretomeP method.** Predictions for proteins secreted by non-classical methods were made using the SecretomeP 2.0 server.
(XLS)

Table S3 **Surface-exposed proteins of** ***R. typhi*** **str. Wilmington identified by LC/MS/MS.** Proteins were run on SDS-PAGE gels and analyzed as described in the methods and section and Text S1. The presence of signal peptides and predicted subcellular localizations are included.
(XLS)

Table S4 **Prediction of co-transcription of** ***sca*** **genes.** The OperonDB algorithm (http://operondb.cbcb.umd.edu/cgi-bin/operondb/operons.cgi) was utilized to determine the probability of each *sca* gene being transcribed with genes immediately upstream or downstream from them.
(DOC)

Text S1 **Supporting information – methods and references.** Details on the processing of Coomassie-stained protein bands for LC-MS/MS analysis and the software used for protein identifications are outlined in file Text S1. References for supporting figures are also included in this Supporting Information file.
(DOC)

Text S2 **Multiple sequence alignment of rickettsial surface cell antigens (Scas).** For each analysis, sequences were aligned using MUSCLE v3.6 [1], [2] (default parameters). Alignments were not manually adjusted. Sca1-5 alignments are noted above each alignment. All *R. typhi* sequences are bolded. Each alignment contains two rows above the sequences. Top row, alignment coordinates (at left) and sequence features, which are described further in Figure 2 of the text. Repeat regions are depicted with an ‘R’, with each individual repeat underlined for *R. typhi* as predicted with HHrepID [3]. Bottom row, vertical bars noting every tenth position within the alignments. Putative signal sequences, as predicted with SignalP v.3.0 [4], are colored green. AT domains, as provided in the *R. typhi* genome annotation [5], are colored dark blue. For the Sca2 alignment further coloring in the N-terminal region depicts conserved features previously described (see Figure S4 of Haglund *et al.* [6]). Additionally, all Pro residues are colored red, with the Pro-rich tract of *R. typhi* distinguished from the different Pro-rich tract of SFG and SFG-like Sca2 sequences. GenBank accession numbers are provided for each sequence in the trees shown in Figure S2.
(DOC)
