# Supplementary figures and images for: Surface Proteome Analysis and Characterization of Surface Cell Antigen (Sca) or Autotransporter Family of Rickettsia typhi
Source: PLoS Pathog. 2012 Aug 9;8(8):e1002856. doi: 10.1371/journal.ppat.1002856 (PMC3415449; doi:10.1371/journal.ppat.1002856)

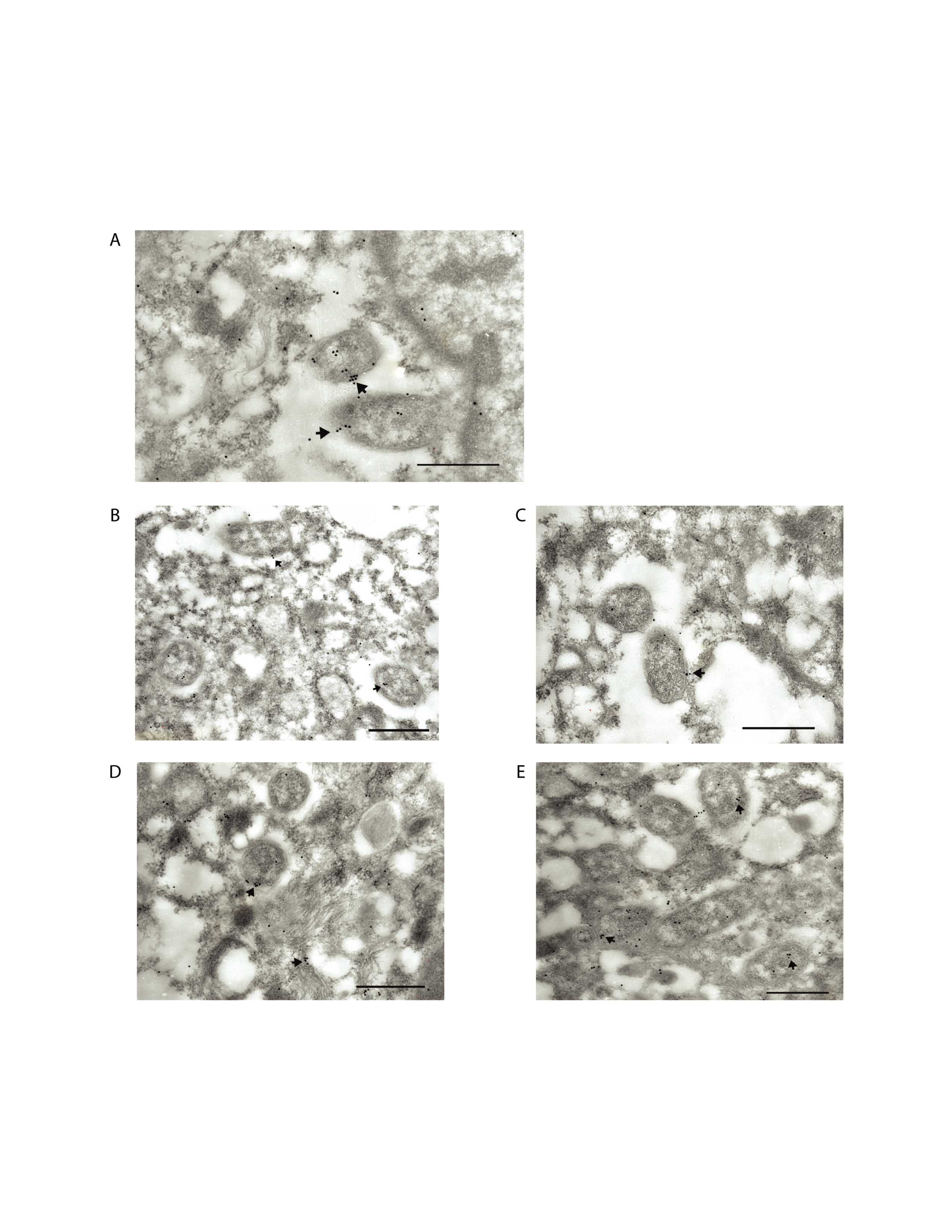

Supplement: Figure S3 — Immunogold post-embedding electron microscopy of Sca expression in Rickettsia typhi grown in L929 cells. Bar = 0.5 um. A) Sca5/ompB is localized in the host cell cytoplasm and in the outer membrane of rickettsiae (arrows); B) Sca1 displays weak labeling in the host cell cytoplasm and in the outer membrane of rickettsiae (arrows); C) Sca2 labeling at the outer membrane (arrow); D) Sca3 labeling mostly at the rickettsial outer membrane (arrows); E) Sca4 labeling in the rickettsial cytoplasm, at its periphery and on the outer membrane (arrows). (TIF) [file ppat.1002856.s003.tif]

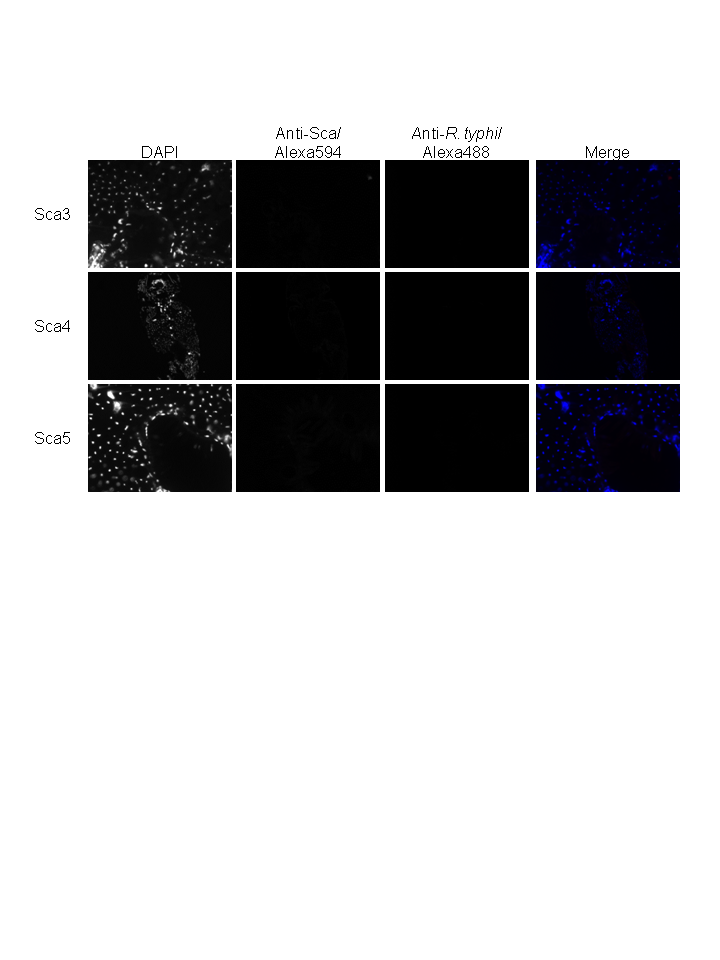

Supplement: Figure S5 — Immunofluorescence assays of Sca expression in uninfected C. felis . Cat fleas were housed in an artificial feeding unit and fed uninfected whole sheep's blood for 14 days. Capsules were placed at −20°C to immobilize fleas before placing them in 3% PFA overnight. Fleas were embedded in OCT medium, frozen and cryosectioned. Sections were labeled with anti-R. typhi rat immune serum (Alexa488-labeled anti-rat secondary Ab – green), anti-serum to the Sca protein indicated on the left (Alexa594-labeled anti-rabbit secondary Ab – red) and mounted in VectaShield medium with DAPI (blue) to counterstain DNA. (TIF) [file ppat.1002856.s005.tif]

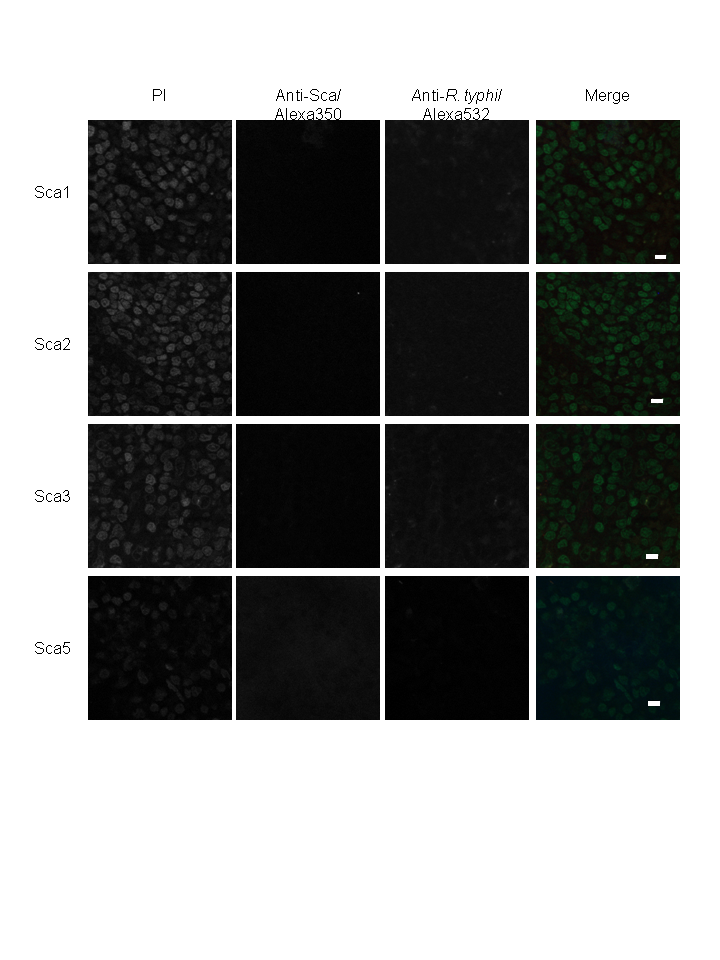

Supplement: Figure S6 — Immunofluorescence assays of Sca expression in uninfected rats. Spleens were harvested from 9 day uninfected female Sprague-Dawley rats and fixed and embedded as described. Sections were labeled with anti-R. typhi rat immune serum (Alexa532-conjugated - red), anti-serum to the Sca protein indicated on the left (Alexa350-conjugated - blue) and stained with propidium iodide (green) to counterstain DNA then mounted in VectaShield medium. (TIF) [file ppat.1002856.s006.tif]

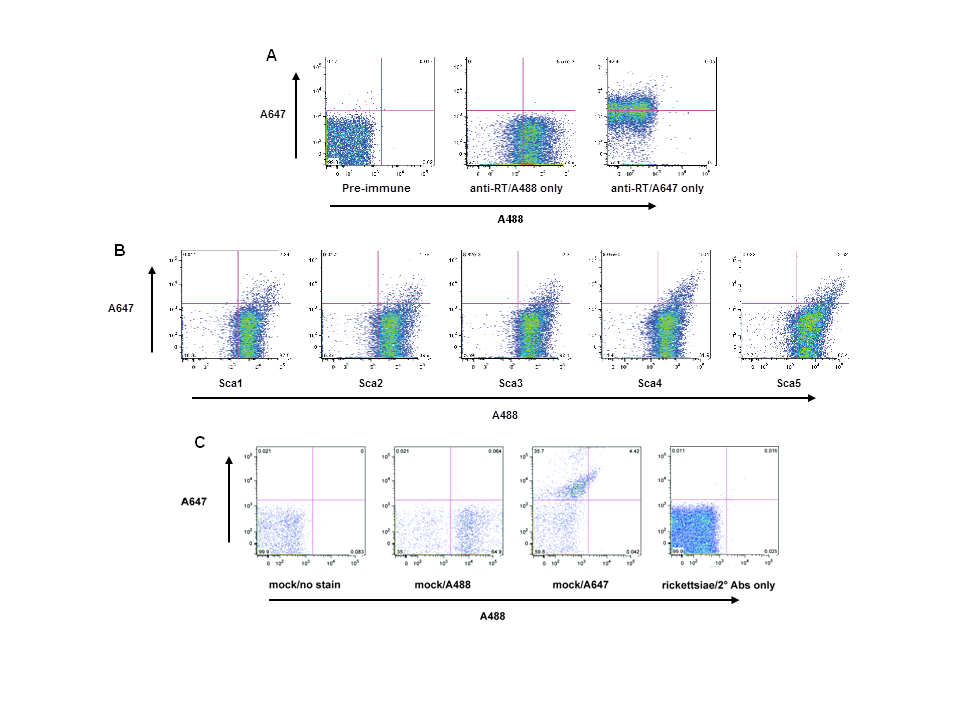

Supplement: Figure S7 — Flow cytometry assays. A) Rickettsiae were gently purified and stained with anti-R. typhi immune sera and either anti-rat AlexaFluor 488 or AlexaFluor 647-conjugated anti-rat secondary Ab to determine the parameters for positive staining. B) Rickettsiae were gently purified and stained with anti-R. typhi immune serum (A488-conjugated anti-rat secondary Ab) and anti-Sca sera (A647-conjugated anti-rabbit secondary Ab) and analyzed by flow cytometry. Rickettsiae stained with both labels appear in the upper right quadrant. Each treatment was analyzed in triplicate and the data shown is representative of three experiments. C) Uninfected cells were taken through the purification process and the resulting pellet stained as indicated to assess the background contribution of host components to the analyses. (TIF) [file ppat.1002856.s007.tif]
