# Supplementary material for: Surface Proteome Analysis and Characterization of Surface Cell Antigen (Sca) or Autotransporter Family of Rickettsia typhi
Source: PLoS Pathog. 2012 Aug 9;8(8):e1002856. doi: 10.1371/journal.ppat.1002856 (PMC3415449; doi:10.1371/journal.ppat.1002856)
Supplement: Table S4 — Prediction of co-transcription of sca genes. The OperonDB algorithm (http://operondb.cbcb.umd.edu/cgi-bin/operondb/operons.cgi) was utilized to determine the probability of each sca gene being transcribed with genes immediately upstream or downstream from them. (DOC) [file ppat.1002856.s011.doc]

**Supplementary table S4. Prediction of co-transcription of *sca* genes.**

| **Gene** | **ORFS (gene pair)** | | **Confidence** |
| --- | --- | --- | --- |
| Sca3 | YP_067396 ATP-dependent protease La. | YP_067397 cell surface antigen Sca3 | confidence=75 n=2 |
| Sca4 | YP_067439 cell surface antigen | YP_067440 ADP/ATP carrier protein 4 | confidence=82 n=10 |
|  | YP_067437 hypothetical protein | YP_067439 cell surface antigen | confidence=76 n=7 |
| Sca5 | YP_067640 rickettsial outer membrane protein B | YP_067641 guanosine-3,5-bis(diphosphate) 3-pyrophosphohydrolase SpoTd | confidence=75 n=6 |
|  | YP_067640 rickettsial outer membrane protein B | YP_067642 beta-glucosidase | confidence=76 n=8 |
|  | YP_067641 guanosine-3,5-bis(diphosphate) 3-pyrophosphohydrolase SpoTd | YP_067642 beta-glucosidase | confidence=82 n=8 |
|  | YP_067641 guanosine-3,5-bis(diphosphate) 3-pyrophosphohydrolase SpoTd | YP_067643 hypothetical protein | confidence=73 n=1 |
|  | YP_067642 beta-glucosidase | YP_067644 integration host factor alpha subunit | confidence=82 n=9 |

OperonDB (http://operondb.cbcb.umd.edu ) was searched for *R. typhi* str. Wilmington *sca* genes and the pre-calculated confidence values with which they are expected to be co-transcribed with genes immediately upstream or downstream of them. Confidence values are based on the number of times the gene pair co-occurs in various genomes, n=the number of genomes in which the pair is found. NB – OperonDB currently lists lower confidence values based on co-occurrence and experimental data.
