# Supplementary material for: Surface Proteome Analysis and Characterization of Surface Cell Antigen (Sca) or Autotransporter Family of Rickettsia typhi
Source: PLoS Pathog. 2012 Aug 9;8(8):e1002856. doi: 10.1371/journal.ppat.1002856 (PMC3415449; doi:10.1371/journal.ppat.1002856)
Supplement: Text S1 — Supporting information – methods and references. Details on the processing of Coomassie-stained protein bands for LC-MS/MS analysis and the software used for protein identifications are outlined in file Text S1. References for supporting figures are also included in this Supporting Information file. (DOC) [file ppat.1002856.s012.doc]

Supplement

Briefly, Coomassie-stained protein bands were excised, cut into approximately 1 × 1 mm pieces and dehydrated with methanol for 5 min. The gel pieces were then washed as follows:  1 x 5 min with 30% methanol/70% water, 2 x 10 min with water, and 3 x 10 min with 100 mM ammonium bicarbonate (NH_4_HCO_3_)/30% acetonitrile.  Gel pieces were dried in a SpeedVac.  Protein disulfide bonds were reduced with 10 mM tris(hydroxypropyl)phosphine (TCEP) in 100 mM NH_4_HCO_3_ for 60 min at 56 °C, followed by alkylation with 55 mM iodoacetamide in 100 mM NH_4_HCO_3_ for 45 min at room temperature in the dark. The gel pieces were washed with 100 mM NH_4_HCO_3_ for 15 min and dehydrated with acetonitrile followed by complete drying in a SpeedVac. Gel pieces were rehydrated in trypsin solution (15 ng/µL trypsin in 50 mM NH_4_HCO_3_) on ice for 45 min. Excess trypsin solution was discarded, replaced with 50 mM NH_4_HCO_3_ and incubated overnight at 37 °C. Digestion buffer was collected and saved.  Peptides were extracted once with 50 mM NH_4_HCO_3_, once with acetonitrile and twice with 5% formic acid in 50% acetonitrile; each extraction was performed by incubating at 37 °C for 15 min with vortexing. All supernatants were combined, dried in a SpeedVac and de-salted using PepClean C-18 Spin columns (Pierce).  De-salted peptides were stored at -20 °C prior to LC-MS/MS analysis.

Chromatographic separation of peptides was performed using an Xtreme Simple nano LC system (Microtech Scientific, Orange, CA) equipped with a 150 mm x 75 μm C-18 reversed-phase column (5 μm particles with 300 Å pores).  Mobile phase compositions were as follows: A) 2 % acetonitrile, 0.1 % formic acid; B) 95 % acetonitrile, 0.1 % formic acid.  Samples were injected in 0.1 % formic acid using a Surveyor Autosampler (Thermo Fisher Scientific, Waltham, MA).  A 20 min LC gradient method from 5 – 40 % solvent B at a flow rate of 1.0 µL/min flow was used to elute the peptides into the mass spectrometer.  MS analysis was performed using an LTQ-Orbitrap (Thermo Fisher Scientific) mass spectrometer equipped with a nanospray ionization source containing an uncoated 10 μm i.d. SilicaTip^TM^ PicoTip^TM^ nanospray emitter (New Objective, Woburn, MA). The spray voltage was 1.8 kV and the heated capillary temperature was 200 °C.  MS spectra were acquired in the profile mode at 60,000 resolution in the Orbitrap mass analyzer.  MS/MS spectra were acquired in the linear ion trap using a top 5 data-dependent acquisition method with dynamic exclusion enabled (repeat count = 1, 180 sec exclusion duration).  Other mass spectrometric data generation parameters were as follows: collision energy 35 %, full scan MS mass range 400-1800 m/z, minimum signal 1000 counts, isolation width 3.0 m/z.  MS/MS spectra were searched against a uniprot mouse database (uniprot release number 2010_05; 64,389 sequences) and a *Rickettsia typhi* database (uniprot release number 2010_05; 1,676 sequences) using Sorcerer-SEQUEST (SageN Research, Milpitas, CA).  The quality of peptide and protein assignments was assessed using PeptideProphet (<http://peptideprophet.sourceforge.net/>) and ProteinProphet (<http://proteinprophet.sourceforge.net/>).  Proteins with probabilities ≥ 0.9 were accepted as true positive identifications.  Proteins identified by one unique peptide were manually verified.
