# Supplementary material for: Surface Proteome Analysis and Characterization of Surface Cell Antigen (Sca) or Autotransporter Family of Rickettsia typhi
Source: PLoS Pathog. 2012 Aug 9;8(8):e1002856. doi: 10.1371/journal.ppat.1002856 (PMC3415449; doi:10.1371/journal.ppat.1002856)
Supplement: Text S2 — Multiple sequence alignment of rickettsial surface cell antigens (Scas). For each analysis, sequences were aligned using MUSCLE v3.6 [1], [2] (default parameters). Alignments were not manually adjusted. Sca1-5 alignments are noted above each alignment. All R. typhi sequences are bolded. Each alignment contains two rows above the sequences. Top row, alignment coordinates (at left) and sequence features, which are described further in Figure 2 of the text. Repeat regions are depicted with an ‘R’, with each individual repeat underlined for R. typhi as predicted with HHrepID [3]. Bottom row, vertical bars noting every tenth position within the alignments. Putative signal sequences, as predicted with SignalP v.3.0 [4], are colored green. AT domains, as provided in the R. typhi genome annotation [5], are colored dark blue. For the Sca2 alignment further coloring in the N-terminal region depicts conserved features previously described (see Figure S4 of Haglund et al. [6]). Additionally, all Pro residues are colored red, with the Pro-rich tract of R. typhi distinguished from the different Pro-rich tract of SFG and SFG-like Sca2 sequences. GenBank accession numbers are provided for each sequence in the trees shown in Figure S2. (DOC) [file ppat.1002856.s013.doc]

**[1-100 ]**

**[ | | | | | | | | | | ]**

R. bellii RML369-C -----M----------------------------------------------------------------------------------------------

R. bellii OSU 85-389 ----MLQPQKEDQSKQHPTF-------------------------------TQADLNVRMLPLV----------------ETYMEVGGLIKNLQKTPSFS

R. prowazekii Madrid E -----**MNKLTAQNLLKKSRFLKYSL-LTSISVGAVMA**IPVERIAMGMDQEAFCAELSKKLSLEFSQS--YEDTI------STTQE---------------

**R. typhi Wilmington -----MNKLTEQNLLKKSRFLKYSL-LASISVGAVMAMPVEGIAMGIDKKAFCTELNTKLLLKFSQSSVNKDTI------STTQE--------------T**

R. akari Hartford -----**MNKLTEQNLLKKSRFLKYSM-LASISVGAVMA**MPIEGMAMSKEA--FRTELSRRLSL-------NKDTT------STSPQIGTVIVPEPEVNTYT

R. australis -----**MNKLTEQNLLKKSRFIKILLCVASISVGA**VMVMPVEGMAMSKEA--FRTELSRKLSL-------NKDTI------STSPQIGTVIVPESEVNTYT

R. felis URRWXCal2 -----**MNKLTEQNLLKKSRFLKYSL-LASISVGA**IIAIPFEGMASSKQKALFQNALEKKLSLRLSQSNGNRDTT------NTPQQIGTVIVPEPERNTYT

R. canadensis McKiel -----**MSTLIEQSQLQKSRFLKYSL-LASISVGTIITLPFEGMA**SSEPI--------------------NLDII--------------------------

R. helvetica -----**MNKLTEQNLLKKSRFLKYFCTASNFMYGAIIA**IPFEGMAMSKEV--FRIDLSNKLLNNLSQLNGNKDTT------NTPQQIGTVIVRGPEINTYT

REIS -----**MNKLTEQNLLKKSRFLKYSL-LASISVGA**IITIPFESMAMSKEA--FRIDLGNKLLNNLSQLNGNKDTT------NTPQQIGTVIVPESEINTYI

R. massiliae MTU5 **MEIFIMNKLTEQHLLKKSRFLKYSL-LASISVGA**IIAIPFEGMAMSKEA--FRIDLSNKLLNNLSQLNGNKDTT------NTPQQIGTVIVPEPEINTYT

Candidatus R. amblyommii ------------------------------------------MATSKEA--LRIDLSNKLESR-AQLNGHKERQ------Y-PQQIGTVIVSEPEINTYT

R. rhipicephali -------**VLTEQHLLKKSRFLKYSL-LASISVGA**IIAIPFEGMAMSKEA--FRIDLSNKLLNNLSQLNGNKDTT------NTPQQIGTVIVPEPEINTYT

R. aeschlimannii -----**MNKLTEQHLLKKSRFLKYSL-LTSISVGA**IIAIPFEGMAMSKEA--FRTDLSNKLLNNLSQLNGSKDTT------STSQQIGTVIVPEPEINTYT

R. montanensis -----**MNKLTEQHLLKKSRFLKYSL-LASISVGA**IIAIPFEGMAMSKEA--FCIDLSSKLLNSLSQLNGNKDTTNTPDTTNTPQQIGTVIVPEPEINAYI

R. slovaca 13-B ------------------------L-LC------------------------------------------------------------------------

R. rickettsii Iowa -----**MNKLTEQHLLKKSRFLKYSL-LASIAVGA**--AIPFEGMAMSKEA--FRIDLSNKLLNHVSQLKGHKDTT------NTPQQIGTVIVPEPEINTYT

R. rickettsii Sheila Smith -----**MNKLTEQHLLKKSRFLKYSL-LASIAVGA**--AIPFEGMAMSKEA--FRIDLSNKLLNHVSQLKGHKDTT------NTPQQIGTVIVPEPEINTYT

R. japonica -----**MNKLTEQHLLKKSRFLKYSL-FASISVGA**IIAIPFEGMAMSKEA--FRIDLSNKLLNHVSQLNGHKDKT------NTPQQIGTVIVPEPEINTYT

R. honei ---------------------------------------------------------------------------------TPQQIGTVIVPEPEINTYT

R. conorii str. Malish 7 -----**MNKLTEQHLLKKSRFLKYSL-LASISVGA**--AIPFEGMAMSKEA--FRIDLSNKLLNHVSQLNGYKDTTN-----NTPQQIGKVIVSEPKINTYT

R. sibirica 246 -----**MNKLTEQHLLKKSRFLKYSL-LASISVGA**--AIPFEGMAMSKEA--FRIDLSNKLLNHVSQLNRHKDTT------NTPQQIGTVIVSEPEINTYT

R. africae ESF-5 -----**MNKLTEQHLLKKSRFLKYSL-LASISVGA**--VIPFEGMAMSKEA--FRIDLSNKLLNHVSQLNGHKDTT------STSQQIGTVIVSEPEINTYT

R. parkeri -----**MNKLTEQHLLKKSRFLKYSL-LASISVGA**--AIPFEGMAMSKEA--FRIDLSSKLLNHVSRLNGNKDTQ------IPRKQIGTVIVSEPEINTYT

R. heilongjiangensis ----------------------------------------------------------------------------------PQQIGTVIVPEPEINTYT

Israeli tick typhus ---------**TEQHLLKKSRFLKYSL-LASISVGA**--AIPFEGMAMSKEA--FRIDLSNKLLNHVSQLNGHKDTT------NTPQQIGTVIVSEPEINTYT

Rickettsia sp. A-167 -----**MNKLTEQHLLKKSRFLKYSL-LASISVGA**--AIPFEGMAMSKEA--FRIDLSNKLLNHVSQLNGHKDTT------NTPQQIGTVIVSEPEINTYT

**[101-200 ]**

**[ | | | | | | | | | | ]**

R. bellii RML369-C ----------------------------------------------------------------------------------------------------

R. bellii OSU 85-389 QQKLIDTSLASIKKEQKNLENIIQELNILRLKLVNKPQNETSYNRRIE-----EKLAEAKQIESRINGYQDKIKTN---------------------VGF

R. prowazekii Madrid E KNNLSNNGPSNKSDMAEELANVTTK---SLYK-VRKMQAPEFKISENKFLNNLDS--QTISKELDPNTYTTES---ISQKPEIILTASSTTVSTDS-NSF

**R. typhi Wilmington NNNLSNNVQSNKSDMTEEVANVTTE---SLCK-VSKMQAPEFKASENKFLNTLD-FQTLSQ-ELDRNSYTTKP---IPQKPEIMLTTSSTTTSMGS-NSF**

R. akari Hartford ASEIREMAISKQSKTFNPLKDIPID---DHYRVVARSKSDVGKARKARPISRRKTLGDAEKVVQNQDDYTHALTEQTPKKPEMMITASSPTVSPAS-NDF

R. australis TSAIREMEISKQPKAFNPLKDIPIE---DHYKVVARSKSDVGKARNARPITRSKTLVGAEKVEQNQNNYTPEPTEQTPQKPEMMITASSPTVSPAS-NGF

R. felis URRWXCal2 SSEIREMEISKQSKASNPLKDIPIE---DHYKKVARSKSDVGKARKVRPPTRSKTFVDTEKVEQNQNNYTHEPTEQTPQKPEIIITASSPTVSPAS-NGF

R. canadensis McKiel ------------ERGDNGLLQPVIN---NKFE-RAKSFATAGNKRK-RPPTRRKILVGNETTEQNQNIDTSEPIEQTAQKPEIIITVSSPTVSPTS-GSF

R. helvetica PSEIREMEISSQSKPSNPLKDIPIE---DHYKKIAQSRSEVGKARKVQPPTRSKTIVGGEKTEQSQNTYIPGPTEQTPQKPEIIITASSPTVSLAS-SSL

REIS PSEIREMEISNKPKASNPLKDVPIE---DHYKKIARSKSDVGKARKVRPPNRSKTFVDSEKTERSQNTYTLEPTEQTPQKPEIIITASSPTVSPAS-NSF

R. massiliae MTU5 PSEIREMKISNKPKASNPLKDVPVE---DHYKVVARSKSDVGKARKVRPITRRKTFVGTEKTEQIQNTYTPEPTEQMLQKPEIIITASSPTVSPASNNSF

Candidatus R. amblyommii PSEIREMKISNEPKAYNPLKDIPIE---DHYKVVARSKSDVGKARKVRPITRCKTFAGIEKTEQSQNTYTPESTEQMPQKPEIIITASSPTVSPAS-NSF

R. rhipicephali PSEIREMKISNKPKASNPLKDVPVE---DHYKVVARSKSDVGKARKVRPITRRKTFVGTEKTEQSQNTYTPEPTEQMPQKPEIIITTSSPTVSPAS-NSF

R. aeschlimannii PSEIREMKISNKPKASNPLKDVPVE---DHYKVVAYSKSDVGKARKVRPITRRKTFVGTEKTEQSQNTYTPELTEQMPQKPEIIITASSSTVSPAS-NSF

R. montanensis PSEIREMKISNKPKASNPLKDVPVE---DHYKVVARSKSEVGKARKVRPITRRKTFVGTEK------------TEQMPQKPEIIITASSPTVSPAS-NSF

R. slovaca 13-B --------------------------------------------------------------------------------------------SPAS-NSF

R. rickettsii Iowa PSEIREMKISNKPKASNPLKDVPIE---DHYKVVARSKSDVGKARKVRPITRRKTFAGIEKTKQSQNTYTPESTEQMPQKPEIIITASSPTVSPAS-NSF

R. rickettsii Sheila Smith PSEIREMKISNKPKASNPLKDVPIE---DHYKVVARSKSDVGKARKVRPITRRKTFAGIEKTKQSQNTYTPESTEQMPQKPEIIITASSPTVSPAS-NSF

R. japonica PSEIREMKISNKPKTSNPLKDVPIE---DHYKVVARSKSDVGKARKVRPITRRKTFAGTEKTAQSQSTYTPESIEQMPQKSEIIITESSPTFSPAS-NGF

R. honei PSEIREMKISNKPKASNPLKDVPIE---DHYKVVARSKSDVGKARKVRPITRRKTFAGIEKTEQS----TPESTEQMLQKSEIIITASSPTVSPAS-NSF

R. conorii str. Malish 7 PSEIREMKISNKPKASNPLKDVPIE---DHYKVVARSKSDVGTARKVRPITRCKTFAGIEKTEQSQNTYTPESTEQMPQKPEIIITASSPTVSPAS-NSF

R. sibirica 246 PSEIREMKISNKPKAYNPLKDVPIE---DHYKVVARSKSDVRKSRKVRPITRCKTFAGIEKTEQSQNTYTPESTEQMPQKPEIIITASSPTVSPAS-NSF

R. africae ESF-5 PSEIREMKISNEPKVYNPLKDIPIE---DHYKVVARSKSDVGKARKVRPITRCKTFAGIEKTEQSQNTYTPESTEQMPQKPEIIITASSPTVSPAS-NSF

R. parkeri PSEIREMKISNKPKAYNPLKDVPIE---DHYKVVARSKSDVGKARKVRPITRCKTFAGIEKTEQSQNTYTPESTEQMPQKPEIIITASSPTVSPAS-NSF

R. heilongjiangensis PSEIREMKISNKPKASNPLKDVPIE---DHYKVVARSKSDVGKARKVRPITRRKTFAGTEKTAQSQSTYTPESIEQMPQKSEIIITESSPTFSPDS-NGF

Israeli tick typhus PSEIREMKISNKPKASNPLKDVPIE---DHYKVVARSKSDVGKARKVRPITRCKTFAGIEKTEQSQNTYTPESTEQMPQKPEIIITASSPTVSPAS-NSF

Rickettsia sp. A-167 RSEIREMKISNKPKASNPLKDVPIE---DHYKVVARSKSDVGKARKVRPITRCKTFAGIEKTEQSQNTYTPESTEQMPQKPEIIITASSPTVSPAS-NSF

**[201-300 ]**

**[ | | | | | | | | | | ]**

R. bellii RML369-C ----------------------------------------------------------------------------------------------------

R. bellii OSU 85-389 ITAN------------------------------------------------------KEDTHIVPKNNLALLGHQ------------------------

R. prowazekii Madrid E VTVSTA-----------------SSLKPVTSY--------------------------QPTPDFKPNYSLGFNTPI--NTNLKIVRKLSFSSEQPQ--IV

**R. typhi Wilmington VNISSA-----------------SSLKPVTPQ--------------------------QSAPDFKPNYSLGSNTPI-------NIKELSFSSE--QQQIA**

R. akari Hartford VTAPNTPNTSLASPEHYTTALSTPSSTPTTPQPTSDSLASPEHYTTELSTPSSTPTTPQPTSDSKPNDNLES-TPP--NTNSKAVRRLSFSSSESQQ-TV

R. australis VTAPNTPNTTLASPEHYTMALGTPSSTPATPY--------------------------QPTQDAQPNDHLGAKTPP--NTNSKAVRRLSF-SSEPQQ-TV

R. felis URRWXCal2 VTAPNTPNTTLTSPEHYTTAPGTP-STPVTPY--------------------------QPTPDSKPNDNLEAKTPP--NTNSKAVRRLSF-SSEPQQTVV

R. canadensis McKiel VTAPNTLNTTPIFSTPSTSPEHIYTTAPGTPYD-------------------------EPIPHATPNNQYA------------VARRLSFSEPQQIEQPT

R. helvetica VTAPNTPSTTLTSPEHYTTAPGTP-STLATPY--------------------------QPTPDSKPNDSVGANTPP--NTNSKVVQKLSFSSE--PQQTV

REIS VTAPNTPSTTLTSPEYYTTAPSTP-STPATPY--------------------------QPTPDSKPNDSVGVNTPP--NTSSKVVQKLSFSSE--PQQIV

R. massiliae MTU5 VTAPNTPNTTLISPEHYTTAPGTPSSTPVTPY--------------------------QSTSDSKPNDSLGANTPP--NTNSKAARRLSFSSSDPQQQTV

Candidatus R. amblyommii ITAPNTPNTTLTSPEHYNTAPGTSSSTPATSY--------------------------QSTSDSKPNDSLGANSPPNINTNSKATRRLSFSSSNPQQQAV

R. rhipicephali VTAPNTPNTTLISPEHYTTAPGTPSSTPATPY--------------------------QPTSDSKPNDSLGANTLPNINTNSKAVRRLSFSNSDPQQQTV

R. aeschlimannii VTAPNTPNTILTSPEHYTTAPGTPSSTPATPY--------------------------QPTPDSKPNDSLGANIPPNINTNSKAARRLSFSSSDPQQQTV

R. montanensis VTAPNTPNTTLTSPEHYATAPGTPSSTPVTPY--------------------------QPTPDSKPNYSLGANTPPNINTNSKAARRLSFSSSDPQQQTV

R. slovaca 13-B ITAPNTPNTNLTSPEHYTTAPGTPSSTPATPY--------------------------QPTSDSKPNDSLGANTPPNINTNSKAARRLSFSSSDPQQQAV

R. rickettsii Iowa ITAPNTPNTTLISPEHYTTAPGTPSSTPVTPY--------------------------QPTSDSKPNDSLGANTPP--NTNSKAARRLSFSSSDPQQQAV

R. rickettsii Sheila Smith ITAPNTPNTTLISPEHYTTAPGTPSSTPVTPY--------------------------QPTSDSKPNDSLGANTPP--NTNSKAARRLSFSSSDPQQQAV

R. japonica ITAPNTPNTTLTSPEHYTTAPGTPSSTPATPY--------------------------QSTSDSKPNDSLGANTPPNINTNSKAVRRLSFSSSGPQQQAV

R. honei ITAPNTPNTTLTSPEHYTTAPGTPSSIPATLY--------------------------QPTSDSKPNDSLGANTPPNINTNSKAARRLSFSSSDPQQQAV

R. conorii str. Malish 7 ITAPNTPNTTLTSPEHYTTAPGTPSSTPATPY--------------------------QSTSDSKPNDSLGANTPPNINTNSKAVRRLSFSSSGPQQQAV

R. sibirica 246 ITAPNTPNTTLTSPEHYTTAPGTPSSTPATPY--------------------------QSTSDSKPNDSLGANTPPNINTNSKAARRLLFSSSDPQQQAV

R. africae ESF-5 ITAPNTPNTTLTSPEHYSTAPGTPSSTPATSY--------------------------QSTSDSKPNDSLGANTPPNINTNSKASRRLSFSSSDPQQQAV

R. parkeri ITAPNTPNTTLTSPEHYTTAPGTSSSTPATPY--------------------------QSTSDSKPNDSLGANPPPNINTNSKAARRLSFSSSDPQQQAV

R. heilongjiangensis VTAPNTPNTTLTSPEHYTTAPGTPSSTPATPY--------------------------QSTSDSKPNDSLGANTPPNINTNSKAVRRLSFSSSGPQQQAV

Israeli tick typhus ITAPNTPNTTLTSPEHYTTAPGTPSSTPATPY--------------------------QSTSDSKPNDSLGANTPPNINTNSKAVRRLSFSSSDPQQQAV

Rickettsia sp. A-167 ITAPNTPNTTLTSPEHYTTAPGTPSSTPATPY--------------------------QSTSDSKPNDSLGANTPPNINTNSKAVRRLSFSSSDPQQQAV

**[301-400 ]**

**[ | | | | | | | | | | ]**

R. bellii RML369-C ----------------------------------------------------------------------------------------------------

R. bellii OSU 85-389 ---------------------------------NIEYSLHRINSANSNKRPVLFDRLLKDLNKKKDEA---KAV--------------WEQFVIYKENKE

R. prowazekii Madrid E Q----HSKSMIPLMPTAPVVLP--KSSIEIEP-EMVSNIPRVDDTISIKSSEVREDI----KGTKDQKTLVDQF--------------NSSIGIWSKKTG

**R. typhi Wilmington Q----HSNSTIPLMPTTSLVLPVKKSSVEIAP-EMVNNISRVDDTVSIKSSEVTEDI-KGTKGKKNKKILANQF--------------NKSIGSWSKKTG**

R. akari Hartford Q----PSKSTVSPIP-TAHPLPITKSSTEIAA-GMVSNILRVNEILGIKLAEVQSTI-KNTKGKKNKKPL-QRL--------------QKQIFLSQKKTK

R. australis Q----PSKSIVPPIPTTPIPLPINKSSTEIAA-GMVSNILRVNKMLGIKLAEVESTI-KNTQGKKNKKPL-QQL--------------QKQIVLSQKKTK

R. felis URRWXCal2 Q----PSTQAKPPVP----PKPLLLSSAGVVAKKVVSNILRVNEMLGIKLAEVESTI-KNTQGKENKKPL-QQL--------------KKQIASSQKTTE

R. canadensis McKiel T----LIKPTMSPKP----------TTLEITA-GIVNRISTHNENAKRKLGMITAAI-EQTSGKKAKKSAQKKAPVAEVSTAGPFLNQQRQLDVLTQKQK

R. helvetica Q----LSTPTVPPKPITSVPLPIKKSSIEVTT-GMVSNISRVNETIGIKLAEVESTI-KNTQGKKNKKPW-QRL--------------HKQLTSSQKTTE

REIS Q----LSKPTLPPRPITPVPLPIKKSSTEIAA-GMVSNISRVNETIGIKLAEVTQAI-KDTTGKKNKKPL-QKL--------------HTQLTSSQKTAK

R. massiliae MTU5 Q-SSSQVKPEVPPKS-TFVPLPIKKSSTEIAA-GMVSNISRVNEMIGIKLAEVTQAIQKNTTDTKNTKSL-QKL--------------YMQLTSAQKTIE

Candidatus R. amblyommii Q-SSSQVKSEVPPKP-TFVPLPIKKSSTEIVA-GMVSNISRVHEMIGIKLAEVTQAI-KDTTDKKDKKRL-QKL--------------YTQLTSTQKTTE

R. rhipicephali Q-SSSQVKPEVPPKS-TFVPLPIKKSSTEIAA-GMVSNISRVNEMIGIKLAEVTQAIQKNTTDTKNTKPL-QKL--------------YTQLTSAQKTIE

R. aeschlimannii Q-SSSQVKPEVHLKS-TF-PLPMKKSSTEIAA-GMVSNISRVNAMIGIKLAEVTQAI-KDTTGKKNKKPL-QKL--------------YTQLTSAQKTIE

R. montanensis Q-SSSQVKPEVLPES-TFVPLPTKKSSTEIAA-GVVSNISRVNEMIGIKLAEVTQAI-KDTTGKKNKKPL-QKL--------------YTQLTSAQKTTE

R. slovaca 13-B Q-SSSQVKSEVPPKP-TFVPLPIKKSSTEIVA-GMVSNISRVNEMIGIKLAEVTQAI-KDTTGKKDKKQL-QKL--------------YTQLTSTQKTTE

R. rickettsii Iowa Q-SSSQVKSEVPPKP-TFVPLPIKKGSTEIVA-GMVSNISRVNEMIGIKLAEVTQAI-KDTTGKKDKERL-QKL--------------YRQLTSTQKTTE

R. rickettsii Sheila Smith Q-SSSQVKSEVPPKP-TFVPLPIKKGSTEIVA-GMVSNISRVNEMIGIKLAEVTQAI-KDTTGKKDKERL-QKL--------------YRQLTSTQKTTE

R. japonica Q-SSSQVKSEVPLKP-TFVPLPTKKSSTEIAA-GMVSNISRVNEIIGIKLAEVTQAI-KDTTGKKNKKPL-QKL--------------YTQLTSAQKTTE

R. honei Q-SSSQVKPEVPPKP-TFVPLPIKKSSTEIVA-GMVSNISRVNEMIGIKLAEVTQAI-KDTTDKKDKERL-QKL--------------YTKLTSTQKTTE

R. conorii str. Malish 7 Q-SSSQVKSEVPPKP-TFVPLPIKKSSTEIVA-GMVSNISRVNEMIGIKLAEVTQAI--DTTDKKDKERL-QKL--------------YTQLTSTQKTTE

R. sibirica 246 Q-SSSQVKSEVPPKP-TFVPLPIKKSSTEIVA-GMVSNISRVNEMIGIKLAEVTQAI-KDTTDKKDKKRL-QKL--------------YTQLTSTQKTTE

R. africae ESF-5 Q-SSSQVKSEVRPKP-TFVPLPIKKSSTEIVA-GMVSNISRVHEMIGIKLAEVTQAI-KDTTDKKDKKRL-QKL--------------YTQLTSTQKTTE

R. parkeri Q-SSSQVKSEVPPKP-TFVPLPIKKRSTEIVA-GMVSNISRVNEMIGIKLAEVTQAI-KDTTDKKDKKRL-QKL--------------YTQLTSTQKTTE

R. heilongjiangensis QCSSSQVKSEVPPKP-TFVPLPTKKSSTEIAA-GMVSNISRINEIIGIKLAEVTQAI-KDTTGKKNKKPL-QKL--------------YKQLTSAQKITE

Israeli tick typhus Q-SSSQVKSEVPPKP-AFVPLPIKKSSTEIVA-GMVSNISRVNEMIGIKLAEVTQAI-KDTTDKKDKERL-QKL--------------YTQLTSTQTTTE

Rickettsia sp. A-167 Q-SSSQVKSEVPPKP-TFVPLPIKKSSTEIVA-GMVSNISRVNEMIGIKLAEVTQAI-KDTTDKKDKERL-QKL--------------YTQLTSTQKTTE

**[401-500 ]**

**[ | | | | | | | | | | ]**

R. bellii RML369-C ----------------------------------------------------------------------------------------------------

R. bellii OSU 85-389 RK------------------------------------------DLNGDENRQIKAAEALLKQYDNDIK--LVEEAFENYKEDSRKSNDIQSPA------

R. prowazekii Madrid E KNKY----------------------------------------DLNKDSSQKMAKFKKDLTTKNKDTD--RLSE---EIDIPE----------------

**R. typhi Wilmington KNKY----------------------------------------EL-NKDSSKIEKFRKDLTTKNKDTD--RLSA---EIDISE----------------**

R. akari Hartford ALKLEAAAIGIKIKELDEENKSLASWNKKKGGVKDKYELDKDSAKI-KENNKKIDDLQQKLTNKNDEAN--SLSREVGGV-------VLTKLQGKATVTE

R. australis ALKLEAAAIGIKIKELDEENRSLASWNKKTGGVKNKRELDKDSAKV-KENNKKIDALQQKLTNKNEEAN--SLSREIGGI-------VLTKPQGKAPVTE

R. felis URRWXCal2 KLKSEAAKIGIKIKELDEENKSL-----KTGRVKNKRELDKDNAKL-KENNKKIDKLQQKLTNKNNEAN--RLSE---EIDISVKKVVLIKPQVKAPVTE

R. canadensis McKiel VNAVRQAAVNKKKRREN---------------------------EV-KRNSTDVEKLITQMNQVQLEQNQRRIAAELQRKAETSTKHVTIKNKNKSKADK

R. helvetica ALKSKAEEIEKKIKILSDQNKELDNLSK---GARLKSTQKQDKIKI-GKNKDKIKKLEKELISKNNEVD--RLSK---KIDISVNKVVLTKPQGKAPVTE

REIS KLKSEAERIGQKIIALDEANTSLSDWNKKGGGIKNKHESDKDTTKV-GKNNKEIEKLKKELVNRNSEAD--TLFQKIEKIDIPV-NKVSIKSQGTVPVTT

R. massiliae MTU5 TLKSKAEKIETQI-------------------------------KVGGEDKNKIKKLEKKLTSKNNKAD--RLFQKIEKIDIPA-NKISIESQETVPVTT

Candidatus R. amblyommii TLKSRAEEIETKI-------------------------------KI-GENKDKIKKLKKELTSKNNKAD--RLFRKIEKIDIPA-NKVSIKSQETVPVTT

R. rhipicephali ALKSKAEKIETQI-------------------------------KV-GENKNKIKKLEKELTSKNNKAD--RLFQKIEKIDIPA-NKISIESQETVPVTT

R. aeschlimannii ALKSRAEEIETKI-------------------------------KI-GENKNKIKKLEKELTSKNNEAN--RLFQKIEKIDIPA-NKVSIKSQETVPVTT

R. montanensis ALKSRVEEIETRI-------------------------------KI-GENKNKIKKLEKELTSKNNEAD--RLFQKIEKIDIPT-NKVSIKSQETVPVTT

R. slovaca 13-B ALKSRAEEIETKI-------------------------------KI-GENKDKIKKLEKELTSKNNKAD--SYFRKIEKIDIPT-NKVSIKSQETVPVTT

R. rickettsii Iowa VLKSRAEEIETKI-------------------------------KI-GKNKDKIKKLEKELTSKNNKAD--RLFQKIEKIDIPA-NKVSIKSQETVPVTT

R. rickettsii Sheila Smith VLKSRAEEIETKI-------------------------------KI-GKNKDKIKKLEKELTSKNNKAD--RLFQKIEKIDIPA-NKVSIKSQETVPVTT

R. japonica ELKSRAEEIETKI-------------------------------KI-GKNKDKIKKLEKELTSKNNKAD--RLFQKIEKIDIPA-NKVSIKSQETVPVTT

R. honei ALKSRAEEIETKI-------------------------------KI-GENKDKIKKLEKELTSKNNKAD--RLFQKIEKIDIPA-NKVSIKSQETVPVTT

R. conorii str. Malish 7 KLKSRAEEIETKI-------------------------------KI-GENKDKIKKLEKELTSKNNKAD--RLFQKIEKIDIPA-NKVSIKSQETVPVTT

R. sibirica 246 TLKSRAEEIETKI-------------------------------KI-GENKGKIKKLEKELTSKNNKAD--RLFQKIEKIDIPA-NKVSIKSQETVPVTT

R. africae ESF-5 TLKSRAEEIETKI-------------------------------KI-GENKDKIKKLKKELTSKNNKAD--RLFRKIEKIDIPA-NKVSIKSQETVPVTT

R. parkeri TLKSRAEEIETKI-------------------------------KI-GKNKDKIKKLEKELTSKNNKAD--RLFQKIEKIDIPA-NKVSIKSQETVPVTT

R. heilongjiangensis ELKSRAEEIETKI-------------------------------KI-GKNKDKIKKLEKELTSKNNKAD--RLFQKIEKIDIPA-NKVSIKSQETVPVTT

Israeli tick typhus TLKSRAEEIETKI-------------------------------KI-GENKDKIKKLEKELTSKNNKAD--RLFQKIEKIDILA-NKVSIKSQETVPVTT

Rickettsia sp. A-167 TLKSRAEEIETKI-------------------------------KI-GENKDKIKKLEKELTSKNNKAD--RLFQKIEKIDILA-NKVSIKSQETVPVTT

**[501-600 ]**

**[ | | | | | | | | | | ]**

R. bellii RML369-C ----------------------------------------------------------------------------------------------------

R. bellii OSU 85-389 ----------SSNLEYLALEEKFASLRKHDE---LDIETKYHELRR------------------------------------------------------

R. prowazekii Madrid E ----------TKVNAFQAQQARIDEAQRDLL---ES----------------------------------------------------------------

**R. typhi Wilmington ----------TKVSAFQAQQARIDEAHRDLL---NP----------------------------------------------------------------**

R. akari Hartford MPLAGPFLNQQQQLELLTQQQKVNAARQAVV---DKRRAEANEVKRNSAEYKDPVSEREEVQLERNQRHIAVEKTKKQQQVVDKQRTEREKKRKEENYQK

R. australis MPLVGPFLNQQQQLEVLTQQQKVNAARQAVV---NKRRMEVNEVKRNSAEYKDLISERGGVQLEQNQQHIAEAKTKKQQQKDNKQSTEREKKRKEENYQK

R. felis URRWXCal2 MPPAGSFLNQQQQLEVLTQQQKVNAARQAVV---DKKRAEANGVKRNSAEYKDLISEREEIQLEQNQRHIAVEKTKKQQQA-------------------

R. canadensis McKiel V---------RQEKTFLQKIHLTKTEQEKKIAEDKNARAAAEKVRKEKNKQAQKAEEISNIKLKVSEQLIQMLDYKAKLQEKLQE---------------

R. helvetica IPPAGVFLNQQQQLDVLTQQQKVTAARQAVV---DKKRAEANEVKRNSAEYKDLKDK-------------------------------------------

REIS AS--------TKVSAFQAQQARINEAQQDVLGQ-NQQHIAAGKTKKQRQAEDKQRTERE-------------------------------KKRKEENHQK

R. massiliae MTU5 AS--------TQVSAFQAQQARINEAQQGVLVQKNQQHIAAEKTKKQRQAEDKQRTERE-----------------------------------------

Candidatus R. amblyommii AS--------TEVSAFQAQQARINEARQGVF---NKNKSSGDNARK-----SSAGTKRE-----------------------------------------

R. rhipicephali VS--------TQVSAFQAQQARINEAQQGILVQKNQQHIAAKKTKKQQQAEDKERTERE-----------------------------------------

R. aeschlimannii AS--------TKVSAFQVQQARINEAQQGVLVQ-NQQHIAAEKTKKQRQAKDKQRTERE-----------------------------------------

R. montanensis AS--------TKVSAFQAQQARINEAQQVVQ---NQQYIAAKKTKKQRQAEDKQRAERE-----------------------------------------

R. slovaca 13-B AS--------TEVSAFQAQQARINEARQGVF---NKNKSSGDNAGK-----SSAGTKRE-----------------------------------------

R. rickettsii Iowa AS--------TEVSAFQAQQARINEARQSIF---NKNKSSGDNARK-----SNAGTKRE-----------------------------------------

R. rickettsii Sheila Smith AS--------TEVSAFQAQQARINEARQSIF---NKNKSSGDNARK-----SNAGTKRE-----------------------------------------

R. japonica AS--------TKVSAFQAQQARINEARQGIF---NKNKSSGDNARK-----SSARTERE-----------------------------------------

R. honei AS--------TEVSAFQAQQARINEARQGVF---NRNKSSGDNARK-----SSAGTKRE-----------------------------------------

R. conorii str. Malish 7 AS--------TEVSAFQAQQARINEARQGVF---NKNKSSGGNARK-----SSAGTKRE-----------------------------------------

R. sibirica 246 AS--------TKVSAFQAQQARINEARQGVF---NKNKLSGDNVRK-----SSAGTKRE-----------------------------------------

R. africae ESF-5 AS--------TEVSAFQAQQARINEARQGVF---NKNKSSGDNARK-----SSAGTKRE-----------------------------------------

R. parkeri AS--------TEVSAFQAQQARINEARQGVF---NKNKSSGDNVRK-----SSAGIKRE-----------------------------------------

R. heilongjiangensis AS--------TKVSAFQAQQARINEARQGVF---NKNKSSGDNARK-----SSARTERE-----------------------------------------

Israeli tick typhus VS--------TEVSAFQAQQARINEARQGVF---NKNKSSGGNARK-----SSAGTKRE-----------------------------------------

Rickettsia sp. A-167 AS--------TEVSAFQAQQARINEARQGVF---NKNKSSGGNARK-----SSAGTKRE-----------------------------------------

**[601-700 ]**

**[ | | | | | | | | | | ]**

R. bellii RML369-C ----------------------------------------------------------------------------------------------------

R. bellii OSU 85-389 ---------------------------------------------KRLDHEKSQLLQDLAQESQLRQHIEDLGSTPSSSNSSDSDVEIKAQGYSATPLKK

R. prowazekii Madrid E ---------------------------------------------NQQKSARKRFEENKTKEK--------VKKIFNNAKKKITRTKLKKLQDQVGNHII

**R. typhi Wilmington ---------------------------------------------NQQKAPGKRLEENKTKGKVIKAAVDGVQKIFNNAKKKISRTKLKKLQDQVGNHVI**

R. akari Hartford EKARDIVEIREDIAKKMQQISNANTT--SNRLQELELTVGAIEDKNQKKEAQKQLSKIKKQEKAIKAAADEAQKILNDAKKAASRTKLKKLQEQIGNHVA

R. australis EKARDILGLREDIANKMQQISNANTT--SNMLQELELKVGAIEDKKQRQEAQKQLSKIKKQEKAIKVAADGAQKILNDAKKEVSRTKLKKLQEQMGNYVA

R. felis URRWXCal2 ------------------------------------------EDKEQRQEAQKQLSKIKKQEKAIKAASDEAQKILNDAKKEASRTKLKKLQEQMDNHVA

R. canadensis McKiel ---------------------------------------------VERIEAKHEVSVIKKELKNIEKLVSKAAKVVNNAETETSRTTLQVMQDKMNGYSE

R. helvetica ---------------------------------------------KQRQAAQKQLSEIKKQEKAMKTVVDRAVDI-------------------------

REIS EKARDIVEIREDIAKKMQSTMSGYSEILPKKLEELEIRVGNIKDKKKKQEAQKQLSEIKKQEKAIKTAADKAKEVAASAKKETSRTALRAMQDKMNGYSE

R. massiliae MTU5 ---------------------------------------------KKKQEAQKQLSEIKKQEQAIKTAADKAKEVAASAKKETSRTALRTMQDKMNGYSE

Candidatus R. amblyommii ---------------------------------------------KKKQEAQKQLLEIKKQEKAIKTASDKAKEVAASAKKEISRTALRAMQDKMNGDSE

R. rhipicephali ---------------------------------------------KK----QKQLSEIKKQEKAIKTAADKAKEVAASVKKETSRTALRTMQDKTNGYSE

R. aeschlimannii ---------------------------------------------KKKQEEQKQLSEIKKQEKAIKTAADKAKEVAASAKKETSRTALRAMQDKMNGYSE

R. montanensis ---------------------------------------------KKKQEAQKQLSEIKKQEKAIKTAADKAK---------------------------

R. slovaca 13-B ---------------------------------------------KKKQEAQKQLSEIKKQEKAIKTASDKAKEVAASAKKETSRTALRAMQDKMNGDSE

R. rickettsii Iowa ---------------------------------------------KKKQEAQKQLSEIKKQEKAVKTASDKAKEVAASAKEETSRTALRAMQDKMNGDSE

R. rickettsii Sheila Smith ---------------------------------------------KKKQEAQKQLSEIKKQEKAVKTASDKAKEVAASAKEETSRTALRAMQDKMNGDSE

R. japonica ---------------------------------------------KKKQEAQKQLSEIKKQEKAIKTASDKAKEVAASVKKETSRTALRAMQDKMNGDSD

R. honei ---------------------------------------------KKKQEAQKQLSEIKKQEK--------AKEVAASAKKETSRTALRVMQDKMNGDSE

R. conorii str. Malish 7 ---------------------------------------------KKKQEAQKQLSEIKKQEKAIKTASDKAKEVAASAKKETSRTALRAMQDKMNGDSE

R. sibirica 246 ---------------------------------------------KKKQEAQKQLLEIKKQEKSIKTASDKAKEVAASAKKETSRTALRAMQDKMNGDSE

R. africae ESF-5 ---------------------------------------------KKKQEAQKQLLEIKKQEKAIKTASDKAKEVAASAKKEISRTALRAMQDKMNGDSE

R. parkeri ---------------------------------------------KKKQEAQKQLLEIKKQEKAIKTASDKAKEVAASAKKETSRTALRAMQDKMN----

R. heilongjiangensis ---------------------------------------------KKKQEAQKQLSEIKKQEKAIKTASDKAKEVAASVKKETSRTALRAMQDKMNGDFD

Israeli tick typhus ---------------------------------------------KKKQEAQKQLSEIKKQEKAIKTASDKVKEVAASAKKETSRTALRAMQDKMNGDSE

Rickettsia sp. A-167 ---------------------------------------------KKKQEAQKQLSEIKKQEKAIKTASDKAKEVAASAKKETSRTALRAMQDKMNGDSE

**[701-800 ]**

**[ | | | | | | | | | | ]**

R. bellii RML369-C --------------------------------------------------------------DGF-----------------------------------

R. bellii OSU 85-389 YASVHSISDLVGSKEDLSVEFEKLVEAESLNNESKTDRENIDPKLKTEFKEIM---------DGF-----------------------------------

R. prowazekii Madrid E MGT-----------------------LPTIPNTLPTSKPTMQ--LSNDINRTV-----SEDEDGYLVPCKAQPQIYTVILPSQEIENH------------

**R. typhi Wilmington IGT------------------------STIPNPLLISKPTMQ--FSNKLNGTL-----LEDEDGYLVPCEAQPQIYTVILPGQEIENH------------**

R. akari Hartford MVTNDKIEESIKKLEGLVLTSSTTSSTATISKQLLTAKSTMQ--LSHELQRTL-----PEDGYGYLMPIKVQQQ--------------------------

R. australis MVTNDKIEGSIKKLEGLVLTPSTTASIATISKQLLTAKSTMP--LSHELQRTL-----PEDEDGYLEPIKVQQQ--------------------------

R. felis URRWXCal2 MVTNDKIEENIKKLEGLALTPSTTSSAATISKQSLTAKSTMQ--LSHELQRTL-----PEDEDGYLEPIKVQQQPYTVMLSNQEVADP------------

R. canadensis McKiel QLD--KIEENLKLLTPIVYSSN-----DSTYKQPPKAKPIMP--LTSGVQGILGKQLQLEEKYGYLVPNQTQPQTYTTVLPNQEVADP------------

R. helvetica -----------EKLEVLVSTQSTTSSTATIPKQPSKAQPIIP--LSHGVQKMLDE--QLDEYGGYLVPIKAQPQTY---MLNQEVADP------------

REIS QLN--KIEENLKLLTPVVYNSST----GSTYKQPPKAKPTMP--LSRGVQRILGE--QLENEDGYLVSTKVQQQPYTVMLSSQEIEDP------------

R. massiliae MTU5 QLN--EM-ANLKLLTPAVYNSST----GSTYKQPPKAKPTMP--LSHGVQRILGE--QPEDEEEYLVPIKVQQQPYIVILPSQEIENPKSMDSGLGSLSS

Candidatus R. amblyommii QLN--KIEENLKLLTPVVYNSST----GPTYKQSPKAKPTIP--LNYGVQRILGE--QPEDEEGYLVPIKVQQQPY------QEIEDP------------

R. rhipicephali QLN--EIEANLKLLTPAVYNSST----GSTYKQPPKAKPTIP--LSHGVQRILGE--QLEDEEGYLVPIKVQQQPYIVTLPSQEIEDPRSMDSGLGSLSS

R. aeschlimannii Q-------------TPAVYNSST----GSTYKQPPKAKPTIP--LSHGVQRILGE--QPEDEEGYLVPIKVQQQPYTVILPSQEIEDP------------

R. montanensis ---------------------------------------------------------------GYLVPMKVQQQPYTVMLPSQEIEDPKSMGSGLGSLSS

R. slovaca 13-B QLN--KIEKNLKLLTPVVYNSST----GPTYKQSPKAKPTIP--LSHGVKRILGE--QPEDEEGYLVPIKVQQQPYTVILPNREIEDP------------

R. rickettsii Iowa QLN--KIEENLKLLTPVVYNSST----GPTYKQSPKAKPTIP--LSHGVQRILGE--QPEDEEGYLVPIKVQQQPYTVMLPNQEIEDP------------

R. rickettsii Sheila Smith QLN--KIEENLKLLTPVVYNSST----GPTYKQSPKAKPTIP--LSHGVQRILGE--QPEDEEGYLVPIKVQQQPYTVMLPNQEIEDP------------

R. japonica ---------------------------GPTYKQPPKAKPTIP--LSHGVQRILCE--QPEDEEGYLVPRKVHQQPYTVMLPNQEIEDP------------

R. honei QLN--KIEENLKLVTPVVYNSST----GPTYKQSPKAKPIIP--LSHGLQRILGE--QPEDEEGYLVPIKVQQQPY-------TIEDP------------

R. conorii str. Malish 7 QLN--KIEENLKLLTPVVYNSST----GPTYKQSPKATPTIP--LSHGVQRILGE--QPEDEEGYLVPIKVQQQPY------QEIEDP------------

R. sibirica 246 QLN--KIEENLKLLTPVVYNSST----GPTYKQSPKAKPTIP--LSHGVQRILGE--QPEDEEGYLVAIKVQQQPY------QEIEDP------------

R. africae ESF-5 QLN--KIEENLKLLTPVVYNSST----GPTYKQSPKAKPTIP--LNYGVQRILGE--QPEDEEGYLVPIKVQQQPY------QEIEDP------------

R. parkeri ------IEENLKLLTPVVYNSST----GPTYKQSPKAKPTIP--LSHGVQRILGE--QPEDEEGYLVPIKVQQQPY------QKIEDP------------

R. heilongjiangensis ---------------------------GPTYKAGAKKQSPTQYHLSHGVQRILGE--QPEDEEGYLVPRKVQQQPYSVMLPNQEIEDP------------

Israeli tick typhus QLN--KIEENLKLLTPDVYNSSI----GPTYKQSPKAKPTIP--LSHGVQRILGE--QPEDEQGYLVPIKVQQQPY------QEIEDP------------

Rickettsia sp. A-167 QLN--KIEENLKLLTPDVYNSST----GPTYKQSPKAKPTIP--LSHGVQRILGE--QPEDEEGYLVPIKVQQQPY------QEIEDP------------

**[801-900 ]**

**[ | | | | | | | | | | ]**

R. bellii RML369-C ----------------------------------------------------------------------------------------------------

R. bellii OSU 85-389 ----------------------------------------------------------------------------------------------------

R. prowazekii Madrid E ---------------------------------------------------------------------------------KSMDSDL------------

**R. typhi Wilmington ---------------------------------------------------------------------------------KSMDSDL------------**

R. akari Hartford -----------------------------------------------------------------------------IQANRSNEG--------------

R. australis -----------------------------------------------------------------------------IQENRSKEG--------------

R. felis URRWXCal2 ---------------------------------------------------------------------------------KSMDSGLGSLPSGIET---

R. canadensis McKiel ----------------------------------------------------------------------------------------------------

R. helvetica ----------------------------------------------------------------------------------------------------

REIS ---------------------------------------------------------------------------------KSMDSGLGSLSNGTLG---

R. massiliae MTU5 GTLGK--------------------------------------------------ETDHSKDIYEAKASQYINYLNSIQPNQSNQAQIDGVMDSLATEMR

Candidatus R. amblyommii -------------------------------------------------------IPSHSKDIYEAKVSQYINYLTSIQPNQSNQAQIDSVIDSLATEMR

R. rhipicephali GTLGKETGEDVGFYSGWGNMPYTQAANVNSSL----------------------QLSSHSKDIYEAKASQYINYLNSIQPNQSNQAQIDGVIDSLVTEMR

R. aeschlimannii ---------------------------------------------------------------------------------KSMDSGLGNLSSGTLGKEI

R. montanensis GTLGKETDEDVGFYSGWGNMPYTQAANVNSSLEGNLVLGMPQQQIQENRSEVSGQLPSHSKDIYEAKASLHINYLNSIQPNQSNQAQIDGVIDSLAAAMR

R. slovaca 13-B -------------------------------------------------------IPSHSKDIYEAKVSQYINYLNSIQPNQSNQAQIDSVIDSLATEMR

R. rickettsii Iowa -------------------------------------------------------IPSHSKDIYEAKVSQYINYLNRIQPNQSNQAQIDSVIDSLATEMR

R. rickettsii Sheila Smith -------------------------------------------------------IPSHSKDIYEAKVSQYINYLNRIQPNQSNQAQIDSVIDSLATEMR

R. japonica -------------------------------------------------------IPSHSKDIYESKVSQYINYLNSIQPNQSNQAQIDSVIDSLATEMR

R. honei -------------------------------------------------------IPSHSKDIYEAKASQYINYLNSIQPNQSNQAQIDSVIDSLATEMK

R. conorii str. Malish 7 -------------------------------------------------------IPSHSKDIYEAKVSQYINYLNSIQPNQSNQAQIDSVIDGLATEMR

R. sibirica 246 -------------------------------------------------------IPSHSKDIYEAKVSQYINYLNSIQPNQSNQAQIDSVIDSLATEMR

R. africae ESF-5 -------------------------------------------------------IPSHSKDIYEAKVSQYINYLNSIQPNQSNQAQIDSVIDSLATEMR

R. parkeri -------------------------------------------------------IPSHSKDIYEAKVSQYINYLNSIQPNQSNQAQIDSVIDSLATEMR

R. heilongjiangensis -------------------------------------------------------IPSHSQDIYESKVSQYINYLNSIQPNQLNQAQIDSVIDGLATEMR

Israeli tick typhus -------------------------------------------------------IPSHSKDIYEAKVSQYINYLNSIQPNQSNQAQIDSVIDGLATEMR

Rickettsia sp. A-167 -------------------------------------------------------IPSHSKDIYEAKVSQYINYLNSIQPNQSNQAQIDSVIDGLATEMR

**[901-1000 ]**

**[ | | | | | | | | | | ]**

R. bellii RML369-C ----------------------------------------------------------------------------------------------------

R. bellii OSU 85-389 ----------------------------------------------------------------------------------------------------

R. prowazekii Madrid E ----------------------------------------------------------------------------------------------------

**R. typhi Wilmington ----------------------------------------------------------------------------------------------------**

R. akari Hartford ----------------------------------------------------------------------------------------------------

R. australis ----------------------------------------------------------------------------------------------------

R. felis URRWXCal2 ----------------------------------------------------------------------------------------------------

R. canadensis McKiel ----------------------------------------------------------------------------------------------------

R. helvetica ----------------------------------------------------------------------------------------------------

REIS ----------------------------------------------------------------------------------------------------

R. massiliae MTU5 KLSADQFNQKLGEIAHLASIKAYEGLFSKLYDIQQARVPETQKVYEQVEIFQSYAEYEEKSRKSATPILSRSSS--------------------------

Candidatus R. amblyommii KFSADQFSQKLGEIAHLASIKAYAGLFEKLYEIQQARILETQKVYEQAEISQSYAEYEENSRKSSTPVLSRSSSAKSV---NFEEKSALLQTTTTDESLR

R. rhipicephali KLSADQFNQKLGEIAHLASIKAYEGLFLKLYDVQQARVPETQKVYEQAEISQSYAEYEEKSRKSATPILSRSSS--------------------------

R. aeschlimannii DEDVGFYSG-----------------------------WGNMPYTQAANVNGSFAEHEEKSRKSGTPVLSRSSS--------------------------

R. montanensis KLSADQFNQKLGDVAHLASIKAYEGLYSKFMKFSKLEFGLKKRI-EQAEIFSLMQSMKRNSRKSGTPVLSRSPSAKSVISSNFEEKSALLQTTTTDESLR

R. slovaca 13-B KFSADQFSQKLGEIAHLASIKAYEGLFEKLYEIQQARIPETQKVYEQAEISQSYAEYEENSRKSSTPVLSRLSSAKSVISSNFEEKSALLQTTTTDESLR

R. rickettsii Iowa KFSADQFSQKLDEIAHLAGIKAYEGLFEKLYEIQQARIPETQKVYEQAEISQSYAEYEENSRKSSTPVLSPSSSAKSVISSNFEEKSALLQTTTTDESLR

R. rickettsii Sheila Smith KFSADQFSQKLDEIAHLAGIKAYEGLFEKLYEIQQARIPETQKVYEQAEISQSYAEYEENSRKSSTPVLSPSSSAKSVISSNFEEKSALLQTTTTDESLR

R. japonica KFSADQFSQKLGEIAHLASIEAYNDLFEKLYEIQQARIPETQKVYKQAEIFQSYAEYEENLRKSSTPVLSRSSSAKSVISSNFEEKSALLQTTTTDESLR

R. honei KFSADQFSQKLGEVAHLASIKAYEGLFEQLYEIQQARIPETQKVYEQAEISQSYAEYEENSRKSSTPVLSRSSSAKSVISSNFEEKSALLQTTTTDESLR

R. conorii str. Malish 7 KFSADQFSQKLGEIAHLASIKAYEGLFEKLYEIQQARILETQKVYEQAEISQSYAEYEENSRKSSIPVLSRSSSAKSVISSNFEEKSALLQTTTTDESLR

R. sibirica 246 KFSADQFSQKLGEIAHLASIKAYEGLFDKLYEIQQARILETQKVYEQAEISQSYAEYEQNSRKSSTPVLSRSSSAKSV---NFEEKSALLQTTTTDESLR

R. africae ESF-5 KFSADQFSQKLGEIAHLASIKAYAGLFEKLYEIQQARILETQKVYEQVEISQSYAEYEENSRKSSTPVLSRSSSAKSV---NFEEKSALLQTTTTDESLR

R. parkeri KFSADQFSKKLGEIAHLFSIKEYEGLFEKLYEIQQARILETQKVYEQAEISQPYAEYEENSRKSSTPVLSRSSSAKSV---NFEEKSAFLQTTTTDESLR

R. heilongjiangensis KFSADQFSQKLGEIAHLASIKAYEGLFEKLYEIQQARIPKTQKVYEQEEIFQSYAEYEENLRKSSTPVLSRSSSAKSVISSNFEEKSALLQTTTTDESLR

Israeli tick typhus KFSADQFSQKLGEIAHLASIKAYEGLFEKLYEIQQARILETQKVYEQAKISQSYAEYEENSRKSSTPVLSRSSSAKSVISSNFEEKSALLQTTTTDESLR

Rickettsia sp. A-167 KFSADQFSQKLGEIAHLASIKAYEGLFEKLYEVQQARILETQKVYEQAKISQSYAEYEENSRKSSTPVLSRSSSAKSVISSNFEEKSALLQTTTTDESLR

**[1001-1100 ]**

**[ | | | | | | | | | | ]**

R. bellii RML369-C ----------------------------------------------------------------------------------------------------

R. bellii OSU 85-389 ----------------------------------------------------------------------------------------------------

R. prowazekii Madrid E ----------------------------------------------------------------------------------------------------

**R. typhi Wilmington ----------------------------------------------------------------------------------------------------**

R. akari Hartford ----------------------------------------------------------------------------------------------------

R. australis ----------------------------------------------------------------------------------------------------

R. felis URRWXCal2 ----------------------------------------------------------------------------------------------------

R. canadensis McKiel ----------------------------------------------------------------------------------------------------

R. helvetica ----------------------------------------------------------------------------------------------------

REIS ----------------------------------------------------------------------------------------------------

R. massiliae MTU5 ----------------------------------------------------------AKSDSSRKNNVSGSIPEIQQLQSEKMRTETLGVQNDLGLDLH

Candidatus R. amblyommii SDNNWKNSAPYSSSPKLDKRGLEYLDLAGDAFVQNLKQPDTLTIETLGLITPTQNTTVAKSDSSRKNNVSGSISGIQQLQSEKMSTETLGVQDDLGLDLH

R. rhipicephali ----------------------------------------------------------AKSDSSRKNNVSGSIPEIQQLQSEKMRTETLGVQNDLGLDLH

R. aeschlimannii ----------------------------------------------------------AKSDSSRKNNVSGSIPEIQQLQSEKMRTETLGVQNDLGLDLH

R. montanensis SDKNWKNPAPYSSSPKLDK-----------------------------IVTPTQNTTVAKSDSSRKNNASGSIPEIQQLQSEKMRTETLGMQNDLGLDLH

R. slovaca 13-B SDNNWKNSAPYSSSPKLDKRGLEYLDLAGDAFVQNLKQPDTLTIETLGLIMPTQNTTVAKSDSSRKNNVSGSISEIQQLQSEKMRTETLGVQDDLGLDLH

R. rickettsii Iowa SDNNWKNSAPYSSSPTLNKRGLEYLDLAGDAFVQNLKQSDTLTIETLDLITPTQNTTVAKSDSSRKNNVSGSIPEIQQLQSEKMRTETLGVQDDLGLDLH

R. rickettsii Sheila Smith SDNNWKNSAPYSSSPTLNKRGLEYLDLAGDAFVQNLKQSDTLTIETLDLITPTQNTTVAKSDSSRKNNVSGSIPEIQQLQSEKMRTETLGVQDDLGLDLH

R. japonica SDNNWKNPAPYSSSPKLDKRGLEYLDLAGDAFVQNLKQPDTLTIEALSLITPTQNTTVAKSDSSRKNNVSGSIPEIQQLQSEKMRTETLGVQDDLELYLH

R. honei SDNNWKNSAPYSSSPKLDKKGLEYLDLAGDAFVQNLKQPDTLTIETLGLITPAPNTTVAKSDSSRKNNVSGSIPEIQQLQSEKMRTETLGVQDDLGLDLH

R. conorii str. Malish 7 SDNNWKNSAPYSSSPKLDKRGLEYLDLAGDAFVQNLKQPDTLTIETLGLITPTQNTTVAKSDSSRKNNVSGSISEIQQLQSEKMRTETLGVQDDLGLDLH

R. sibirica 246 SDNNWKNSAPYSSSPKLDKRGLEYLDLAGDAFVQNLKQPDTLTIETLGLITPTQNTTVAKSDSSRKNNVSGSISEIQQLQSEKMRTETLGVQDDLGLDLY

R. africae ESF-5 SDNNWKNSAPYSSSPKLDKRGLEYLDLAGDAFVQNLKQPDTLTIETLGLITPTQNTTVAKSDSSRKNNVSGSISEIQQLQSEKMSTETLGVQDDLGLDLH

R. parkeri SDNNWKNSALYSSSPKLDKRGLEYLDLAGDAFVQNLKQPDTLTIETLGLITPTQNTTVAKSDSSRKNNVSGSISEIQQLQSEKMRTETLGVQDDLGLDLH

R. heilongjiangensis SDNNWKNSAPYSSSPKLDKRGLEYLDLAGDAFVQNLKQPDTLTIEALSLITPTQNTTVAKSDSSRKNNVSGSIPEIQQLQSEKMRTETLGVQDDLELYLH

Israeli tick typhus SDNNWKNSAPYSSSPKLDKRGLEYLDLAGDAFVQNLKQPDTLTIETLSLITPTQNTTVAKSDSSRKNNVSGSISEMQQLQSEKMRTETLGVQDDLGLDLH

Rickettsia sp. A-167 SDNNWKNSAPYSSSPKLDKRGLEYLDLAGDAFVQNLKQPDTLTIETLGLITPTQNTTVAKSDSSRKNNVSGSISEMQQLQSEKMRTETLGVQDDL-----

**[1101-1200 ]**

**[ | | | | | | | | | | ]**

R. bellii RML369-C ----------------------------------------------------------------------------------------------------

R. bellii OSU 85-389 ----------------------------------------------------------------------------------------------------

R. prowazekii Madrid E ----------------------------------------------------------------------------------------------------

**R. typhi Wilmington ----------------------------------------------------------------------------------------------------**

R. akari Hartford ----------------------------------------------------------------------------------------------------

R. australis ----------------------------------------------------------------------------------------------------

R. felis URRWXCal2 ---------------------------------------------------------------------------------------------------D

R. canadensis McKiel -------------------------------------------------------------------------------------KRMDSGFVSLDKETD

R. helvetica ----------------------------------------------------------------------------------------------------

REIS ------------------------------------------------------------------------------------------------KETD

R. massiliae MTU5 SQEERFDSGFRSLESQNLSSAYDFDQLSASWDAAYNQRMNTNVPQETLTEESTYSGSSKKKQGNIIKRAVSKVGSILQTNDAENRKRKRDGETSKQRTVD

Candidatus R. amblyommii -----------------------------------------YAPQETLTEESTYLVSSKKKQGNIIKRALSKVGSILQTNYAENRRRKRDGETSKQRTVD

R. rhipicephali SQEERFDSGFRSLESQNLSSAYDFDQLSASWDAAYNQRMNTNAPQETLTEESTYSGSSKKKQGNIIKRAVSKVGSILQTNDAENRKKKGDGETSKQRTVD

R. aeschlimannii SQEERFDSAFRSLESQNLSSAYDFDQLSASWDAAYNQRMNTNAPQETLTEESTYLVSSKKKQGNIIKRAVSKVGSILQTNDAENRKSKRDGETSKQRTVD

R. montanensis SQEERFDSGFRSLESQNLSSAYDFDQLSASLDAAYNQRMNTNAPQETLTEESTYSVSSKKKQGNIIKRAVSKAGSILQTNGAENRKRKRDGETSKQRTVD

R. slovaca 13-B -----------------------------------------YAPQETFTEESTYLVSSKKKQGNIIKRAVSKVGSILQTNYAENRKRKRDGETSKQRTVD

R. rickettsii Iowa -----------------------------------------YAPQETLTEESTYLVSSKKKQGNIIKRAVSKVESILHTNYSENRKRKRNGETSKQRTVD

R. rickettsii Sheila Smith -----------------------------------------YAPQETLTEESTYLVSSKKKQGNIIKRAVSKVESILHTNYSENRKRKRDGETSKQRTVD

R. japonica -----------------------------------------YTPQETLTEESTYLVSSKNKQGNIIKRAVSKIGSILQTNYAENRKRKRDGETSKQRTVD

R. honei -----------------------------------------YVPQETLTEESTYLVSSKKKQGNIIKRAVSKVGSILQINYAENRKRKRDGETSKQRTVD

R. conorii str. Malish 7 -----------------------------------------YTPQETLTEKSTYLVSSKKKQGNIIKRAVSKVGSILQTNYAENRRRKRDGETSKQRTVD

R. sibirica 246 -----------------------------------------YAPQDTLTEESTYLVSSKKKQGNIIKRALSKVDSILQTNYAENRRRKRDGETSKQRTVD

R. africae ESF-5 -----------------------------------------YAPQETLTEESTYLVSSKKKQGNIIKRALSKVGSILQTNYAENRRRKRDGETSKQRTVD

R. parkeri -----------------------------------------YAPQETLTEESTYLVSSKKKQGNIIKKALSKVGSILQTNYAENRRRKRDGETSKQRTVY

R. heilongjiangensis -----------------------------------------YTPQETLTEESTYLVSSKNKQGNIIKRAVSKVGSILQTNYAENRKRKRDGETSKQRTVD

Israeli tick typhus -----------------------------------------YTPQETLTEESTYLVSSKKKQGNIIKRAVSKVGSILQTNYAENRRRKRDGETSKQRTVD

Rickettsia sp. A-167 ----------------------------------------------------------------------------------------------------

**[1201-1300 ]**

**[ | | | | | | | | | | ]**

R. bellii RML369-C ----------------------------------------------------------------------------------------------------

R. bellii OSU 85-389 ----------------------------------------------------------------------------------------------------

R. prowazekii Madrid E ----------------------------------------------------------------------------------------------------

**R. typhi Wilmington ----------------------------------------------------------------------------------------------------**

R. akari Hartford -------------------------------------------------SNPNSTTYLDLYPEE------------------------------------

R. australis -------------------------------------------------SNPNFTTYLDLYPEE------------------------------------

R. felis URRWXCal2 EDTEFNLGWGNGHHKELILSVDSKLIEKAAQLISGLDVKRSEILQT---TSPSQQKSLNLALQGIENDYQEAIEVYQGLQQALIHKSEDIKAYNAEAEKR

R. canadensis McKiel ADVGFNTSCGKGTD-CTQVGQELSFELPTLKVLGYQDNINQTLDTIARANSIERPILFQNLLKDLERRKQEAKMVEEQAIMHVINNPND-----------

R. helvetica ----------------------------------------------------------------------------------------------------

REIS EDAGFYSGWGNKHHKESSLSVDSGLTKKAAQLISLLDAKRTEILQT---TSPSQQKSLNLALQE------------------------------------

R. massiliae MTU5 QEGGFGHAWGNENHKESSLSVVSGFTKKATQLVSLLDAKRTTILQT---TSPSQKKSLNLVLQEIENDYHETIKISQELQQVLIHKPEDIKAYNAKAEKK

Candidatus R. amblyommii QEREFGHVWGNENHKESSLSVVSGCIKKATQLISLLDAKRTAILQT---TSPSQRRSVSLVLQEIKNDYQEAVKISQKLQQVLIRKPEDIKAYNAKAEKK

R. rhipicephali QEGGFGHAWGNENHKESSLSVVSGFTKKATQLISLLDAKRTAILQT---TSPSQKKSLNLVLQEIENDYQEAIKISQELQQVLIRKPEDIKAYNAKAEKK

R. aeschlimannii QEGGFGHAWRNENHKESSLSVVSGFTKKATQLVSLLDAKRTAILQT---TSPSQKKSLDLALQEIENGYQETIKISQELQQVLIRKPEDIKAYNAKAEKK

R. montanensis QEGGFGHAWGNENHKESSLSVVSGFTKKATQLISLLDAKRTAILQT---TSPSQKKSLNLALQEIENDYQEAIKISQELQQVLIRKPEDIKAYNAKAEKK

R. slovaca 13-B QEGEFGHAWGNENHKESSLSVVSGFIKKATQLISLLDAKRTAILQT---TSSSQSKSVSLVLQEIENDYREAIKISQELQQVLIRKPEDIKAYNAKAEKK

R. rickettsii Iowa QEGEFGHAWGNENHKESSLSVVSGFTKKATQLISLLDAKRTAILQT---TSPSQRKSLSLVLQEIENDYREAIKISQELQQVLIRKPEDIKAYNAKAEKK

R. rickettsii Sheila Smith QEGEFGHAWGNENHKESSLSVVSGFTKKATQLISLLDAKRTAILQT---TSPSQRKSLSLVLQEIENDYREAIKISQELQQVLIRKPEDIKAYNAKAEKK

R. japonica QEGEFGHAWGNENH-----NVVSGFTKKATQLISLLDAKRTAILQT---TSPSQRKSLSLVLQEIENDYREAIKISQELQQVLIRKPEDIKAYNSKAEKK

R. honei QEGKFGHAWGNENHKESSLSVVIGFTKKATQLISLLDAKRTRILQTTAATSPSQRKSLSLVLQEIENDYQETIKISQELQQVLIRKPKDIKAYNAKVEKK

R. conorii str. Malish 7 QEGEFGHAWGNENHKESSLSVVSGCIKKATQLISLLDAKRTAILQT---TSPSQRRSVSLVLQEIENDYREAIKISQKLQQVLIRKPEDIKAYNAKAEKK

R. sibirica 246 QEGEFGHAWGNENHKESNLSVVSGCIEKATQLISLLDAKRTAILQT---TSPSQRRSVSLVLQEIENDYREAIKVSQKLQQVLIRKPEDIKAYNAKAEKK

R. africae ESF-5 QEREFGHVWGNENHKESSLSVVSGCIKKATQLISLLDAKRTAILQT---TSPSQRRSVSLVLQEIENDYQEAVKISQKLQQVLIRKPEDIKAYNAKAEKK

R. parkeri QEGEFGHVWGNENHKESSLSVVSGCIKKATQLISLLDAKRTAILQT---TSPSQRRSVSLVLQEIENDYREAIKISQKLQQVLIRKPEDIKAYNAKAEKK

R. heilongjiangensis QEGEFGHAWGNENH-----NVVSGFTKKATQLISLLDAKRTAILQT---TSPSQRKSLSLVLQEIENDYREAIKISQELQQVLIRKPEDIKAYNSKAEKK

Israeli tick typhus QEGEFGHAWGNENHKESSLSVVSGCMKKATQLISLLDATRTAILQT---TSPSQRRSVSLVLQEIENDYREAIKISQKLQQVLIRKPENIKAYNAKAEKK

Rickettsia sp. A-167 ----------------------------------------------------------------------------------------------------

**[1301-1400 ]**

**[ | | | | | | | | | | ]**

R. bellii RML369-C ----------------------------------------------------------------------------------------------------

R. bellii OSU 85-389 ----------------------------------------------------------------------------------------------------

R. prowazekii Madrid E ----------------------------------------------------------------------------------------------------

**R. typhi Wilmington ----------------------------------------------------------------------------------------------------**

R. akari Hartford ----------------------------------------------------------------------------------------------------

R. australis ----------------------------------------------------------------------------------------------------

R. felis URRWXCal2 LDAIKSRADKYFNNIETDADVGFNQDWSNVHTQTAQSDSVLKMQQALLEK--------------------------------------------------

R. canadensis McKiel ----------------------------------------------------------------------------------------------------

R. helvetica ----------------------------------------------------------------------------------------------------

REIS ----------------------------------------------------------------------------------------------------

R. massiliae MTU5 IDAIKSRADKHFNNIKTDADVGFNPNWGNSHSMPTANMNSLP----------------------------------------------------------

Candidatus R. amblyommii LDAIKSRADKHFNNIETDVDVGFNPNGGNSHSMPTANMDSLPKNLAVTPPINVGSLYNSLQAQQFREDH----------------------KNIIIN---

R. rhipicephali LDAIKSRADKHFNNIKTDADVGFNPNWGNSHSIPTANMNSLP----------------------------------------------------------

R. aeschlimannii LDAIKSRANKHL----------------------------------------------------------------------------------------

R. montanensis LDAIKSRADKHFNNIETDADVGFNPNWGNSHSMPTANMNNLPKNLAI----NVGSLYNSPQAQQFREDH----------------------KNIIINAIS

R. slovaca 13-B LDAIKSRADKYFNNIETDVDVGFNPNGGNSHSMPTANMDSLPKNLAVTPPINVGSLYNSPQAQQFREDH----------------------KNIIINDRS

R. rickettsii Iowa LDAIKSRADKHFDNIETDVDIGFNPNGGNSHSMPTANMDSLPKNLAVTPPINVGSLYNSPQAQQFREDH----------------------KNIIIN---

R. rickettsii Sheila Smith LDAIKSRADKHFDNIETDVDIGFNPNGGNSHSMPTANMDSLPKNLAVTPPINVGSLYNSPQAQQFREDH----------------------KNIIIN---

R. japonica LDAIKSRADKYFNNIETDADVGFNPNGGNNHSMPTANMDSLPKNLAVTPPINVGSLYNSPQAQQFREDHIVRELTDVDASIYNTAPPEVVKEAEALLDRS

R. honei LDAIKSRADKHFNNIETDVDVGFNPNGGNSHSMVTTNMDSLPKNLAVTPPINVGSLYNSPQAQQFREDH----------------------KNIIINDRS

R. conorii str. Malish 7 LDAIKSRADKHFNNIETDVDVGFNPNGGNSHSMPTANMDILPKNLAVTPPTNVGSLYNSPQAQQFQEDH----------------------KNIIIN---

R. sibirica 246 LDAIKSRADKHFNNIETDVDVGFNPNGGNSHSMPTANMDSLPKNLAVTPPINVGSLYNSPQAQQFREDH----------------------KNI------

R. africae ESF-5 LDAIKSRADKHFNNIETDVDVGFNPNGGNSHSMPTANMDSLPENLAVTPPINVGSLYNSLQAQQFREDH----------------------KNIIIN---

R. parkeri LDAIKSRADKHFNNIEIDVDVGFNPNGGNSHSMPTANMDSLPKNLAVTPPINVGSLYNSPQAQQFREDH----------------------KNIIIN---

R. heilongjiangensis LDAIKSRADKYFNNIETDADVGFNPNGGNSHSMPPANMDSLPKNLAVTPTINVGSLYNSPQAQQFREDH----------------------KNIIIN---

Israeli tick typhus LDAIKSXADKHFNNIETDVDVGFNPNGGNSHSMPTANMDSLPKNLAVTPPINVGSLYNSPQAQQFREDH----------------------KNIIIN---

Rickettsia sp. A-167 ----------------------------------------------------------------------------------------------------

**[1401-1500 ]**

**[ | | | | | | | | | | ]**

R. bellii RML369-C -----------------------------------------------------------------------------------------------DRVAA

R. bellii OSU 85-389 -----------------------------------------------------------------------------------------------DRVAA

R. prowazekii Madrid E -------------------------------------------------------------------------------------------WTLSSKTLG

**R. typhi Wilmington -------------------------------------------------------------------------------------------LSLSTKTLG**

R. akari Hartford -------------------------------------------------------------------------------EGLKSIDLALGILPTSASVIP

R. australis -------------------------------------------------------------------------------EGLKSIDLGLGSLPIGASVIP

R. felis URRWXCal2 -----------------------------------------------------------RSKEGSNPNFTTYLDPQPGEEGLKSIDSGLGSLPIDASVIP

R. canadensis McKiel -------------------------------------------------------------------------------------DQAKEQVALVDKLLE

R. helvetica -------------------------------------------------------------------------------------------KSMDSGFVS

REIS ----------------------------------------------------------------------------------------------------

R. massiliae MTU5 --------------------------------------------------------------------------------------------NLVDSTIK

Candidatus R. amblyommii -------------------------------------------------------------------------------------DRSQGRLNLVDSTIK

R. rhipicephali --------------------------------------------------------------------------------------------NLVDSTIK

R. aeschlimannii ---------------------------------------------------------------------YNTAPPEVLKEAEALLDRSQGRLNLVDSTIK

R. montanensis SISSSGVPKESIDSTITKSLPYLDNDQYQEVVLRVEKVRDINTRGYLLNKLKQLGASEVRELTDVDASIYNIAPPEVLKEAEALLDRSQGRLNLVDNTIK

R. slovaca 13-B QGRLNLVDSTIVRELTDVDASIYNTAPPEVLKEAEALLDRSQGR------LNLVDSTIVRELTDVDASIYNTAPPEVLKEAEALLDRSQGRLNLVDSTIK

R. rickettsii Iowa -------------------------------------------------------------------------------------DRSQGRLNLVDSTIT

R. rickettsii Sheila Smith -------------------------------------------------------------------------------------DRSQGRLNLVDSTIT

R. japonica QGRLNLVDSTIVRELTDVDASIYNTAPPEVVKEAEALLDRSQDR------LNLVDSTIVRELTDVDASIYNIAPPEVVKEAEALLDRSQDRLNLVDSTIK

R. honei QGRLNLVDSTIVKELTDVDASIYNTAPPEVLKEAEALLDRSQGR------LNLVDSTIVKELTDVDASIYNTAPPEVLKEAEALLDRSQGRLNLVDSTIK

R. conorii str. Malish 7 --------------------------------------DRSQGR------LNLVDSTIVRELTDVDASIYNTAPPEVLKEAEALLDRSQGRLNLVDNTIK

R. sibirica 246 -----------------------------------------------------------------------------------INDRSQGRLNLVDSTIK

R. africae ESF-5 -------------------------------------------------------------------------------------DRSQGRLNLVDSTIK

R. parkeri -------------------------------------------------------------------------------------DRSQGRLNLVDSTIK

R. heilongjiangensis --------------------------------------DRSQGR------LNLVDSTMVRELTDVDASIYNTAPPEVVKEAEALLDRSQGRLNLVDSTIK

Israeli tick typhus --------------------------------------DRSQGR------LNLVDSTIVRELTDVDAS-YNTAPPEVLKEAEALLDRSQGRLNLVDSTI-

Rickettsia sp. A-167 ---------------------------------------------------------------DVDAS-YNTAPPEVLKEAEALLDRSQGRLNLVDSTIK

**[1501-1600 ]**

**[ | | | | | | | | | | ]**

R. bellii RML369-C ----------------------------------------------------------------------------------------------------

R. bellii OSU 85-389 ----------------------------------------------------------------------------------------------------

R. prowazekii Madrid E KEIDEVH-----------------------------------------------PNIVLLSEK-------------------------------------

**R. typhi Wilmington KEIDEVH-----------------------------------------------PNIGLSSEK-------------------------------------**

R. akari Hartford QYVEELNNLASFHSLNQIQQEKSLIVTNLLLSEASALSDDEFRAVANEVHPKIRPILTYNRRK-------------------------------------

R. australis QYVEELNNLPSFPSLSQIQQGKSLIITNMLLSETSALSDDEFKAVANEIHPKIQPILTYNRKK-------------------------------------

R. felis URRWXCal2 QYVEELNNLASFSSLNQIQQKKPLIITNMLLSETSALSDDEFRAVANEVHPKIRPILTYNRQK-------------------------------------

R. canadensis McKiel KYNTDIKLV-------------------------RKTLEDNKENNSRNNVKKPLERLVFEAKL-------------------------------------

R. helvetica RTSD----------------------------------------------------VGFGSNQ-------------------------------------

REIS ----------------------------------------------------------------------------------------------------

R. massiliae MTU5 KGTQPL------STIYESVSDENLASETIY-----KTEQPKPSISYKNTSKRKLPIPLFRSAE-------------------------------------

Candidatus R. amblyommii KGTQPLSNL---STIYESVSYEDLASETIY-----KTEQPKPSISYTNTSKRKLP---FRSAE-------------------------------------

R. rhipicephali KGTQPL------STIYESVSYENLASETIYKTEQPKTEQPKPSVSYKNTSKRKLPIPLFRSAE-------------------------------------

R. aeschlimannii KGTQSLSNL---STIYESVSYENLASETI--------EQPNPSISYKNTSKRKLPIPLFRSAQ-------------------------------------

R. montanensis KGTQPLSNL---STIYESVSYENLASETIY-----KTEQPKPSISYKNTSKRKLPIPLLRSAE-------------------------------------

R. slovaca 13-B KGTQPLSNL---STIYESVSYENLASETIY-----KTEQPKPSISYTNTSKRKLPIPLFRSAE-------------------------------------

R. rickettsii Iowa KGTQPLSNL---STIYESVSDENLASETIY-----KTEQPKPSISYTNTSKRKLPIPLFRSAE-------------------------------------

R. rickettsii Sheila Smith KGTQPLSNL---STIYESVSDENLASETIY-----KTEQPKPSISYTNTSKRKLPIPLFRSAE-------------------------------------

R. japonica KGTQPL------STIYESVSYENLASETIY-----KTEQPKPSISYTNTSKRKLPIPLFRSAD-------------------------------------

R. honei KGTQPLSNL---STIYESVSYENLASETIY-----KTEQPKPSISYTNTSKRKLPIPLFKSAE-------------------------------------

R. conorii str. Malish 7 KGTQPLSNL---STIYESVSYENLASETIY-----KTEQPKPSISYTNTSKRKLPIPLFRSAE-------------------------------------

R. sibirica 246 KGTQPLSNL---STIYESVSYENLASETIY-----KTEQPKPSISYTNTSKRKLPIPLFRSAE-------------------------------------

R. africae ESF-5 KGTQPLSNL---STIYESVSYEDLASETIY-----KTEQPKPSISYTNTSKRKLP---FRSAK-------------------------------------

R. parkeri KGTQPLSNL---STIYESVSYENLASETIY-----KTEQPKPSISYTNTSKRKLPIPLFRSAELDKKLEYLDLEDKLLEVEEARIVKEKEAIAKLNQYQD

R. heilongjiangensis KGTQPLSNL---STIYESVSYENLASETIY-----KTEQPKPSISYTNTSKRELPIPLFRSAD-------------------------------------

Israeli tick typhus --VRELTDV---DASYNTA-----------------------------------PPEVLKEAE-------------------------------------

Rickettsia sp. A-167 KGTQPLSNL---STIYESVSYENLASETIY-----KTEQPKPSISYTNTSKRKLPIPLFRSAE-------------------------------------

**[1601-1700 ]**

**[ | | | | | | | | | | ]**

R. bellii RML369-C ---------------------------------------DINSELETLTAAKGLNDQPNANIGTKTEDA----SDQLLL---------------------

R. bellii OSU 85-389 ---------------------------------------DINSELETLTAAKGLNDQPNANIGTKTEDA----SDQLLL---------------------

R. prowazekii Madrid E ---------------------------------------ETEAKIVED-----------VKISNTTNSRGVFENTSKSFGQHNN-FKLTQDLYGKDRKQY

**R. typhi Wilmington ---------------------------------------EIEAKLVEDETTYSQDVQNAINILNTTNDK----NTARLFGQHNN--KLTLDLHDNDRKKY**

R. akari Hartford ---------------------------------------EKEAKLVEAETAHNQVIQKAIKTPNSTKAKEAVENAARLFGQHNNSFKLMQDIRDEHRRKY

R. australis ---------------------------------------ETEAKLVKAETAHNQAVQNAIKTPNSTQAKEAVENAANLFGQHNNSFKLTQNLRDEHRRKY

R. felis URRWXCal2 ---------------------------------------EKEAKLVEAEAAHSQVVQNAIKTPNSTQAKEAVENAAKLFGQYNNSFKLTQALRDEHRKKY

R. canadensis McKiel -------------------------------------PPIREYDALCEESQKVIASKRATEELRNQREDDPELEKLAIIEYMEVQKKQSQL---EQDIKA

R. helvetica -------------------------------------------------------------------------NSNRAYEDLPLIEPENQQ---QHDMLT

REIS ----------------------------------------------------------------------------------------------------

R. massiliae MTU5 -------------------------------LDKKLDYLDLEDKLLKVEEARIVKEKQAIAKLNQYRDPENLEFKRLAVEARDLSSKESQL---KEKRKA

Candidatus R. amblyommii -------------------------------LDKKLEYLDLEDKLLEVEEARIVKEKQDIAKLNQYQDPENLEFKRLAMEALDLSSKESQL---KQKRKA

R. rhipicephali -------------------------------LDKKLDYLDLEDKLLKVEEARIVKEKQAIAKLNQYQDPENLEFKRLAVETRDLSSKESQL---KEKRKA

R. aeschlimannii -------------------------------LDKKLDYLDLEDKLLKVEEARIVKEKQAIAKLNQYQDPENLEFKRLAIEARDLSSKESQL---KEKRKT

R. montanensis -------------------------------LDKKLDYLDLEDKLLKVEEARIVKEKQAIAKLNQYQDPENLEFKRLAIEARDLSSKESQL---KEKRKA

R. slovaca 13-B -------------------------------LDKKLEYLDLENKLLEVEEARIVKEKEVIAKLNQYQDPENLEFKRLAMEALDLSSKKSQL---KQKRKA

R. rickettsii Iowa -------------------------------LDKKLEYLDLEDKLLEVEEARIVKEKQAIAKLNQYQDPENLEFKRLAMEALDLSLKESQL---KAKRKA

R. rickettsii Sheila Smith -------------------------------LDKKLEYLDLEDKLLEVEEARIVKEKQAIAKLNQYQDPENLEFKRLAMEALDLSLKESQL---KAKRKA

R. japonica -------------------------------LDKKLEYLDLEDKLLEVEEARIVKEKQAIATLDQYQDPENPEFKRLAMEALDLSSKESQL---KAKRKT

R. honei -------------------------------LDKKLEYLDLEDKLLEVEEARIVKEKQAIAKLNQYQDPENLEFKRLAMEALDLSSKESQL---KQKRKA

R. conorii str. Malish 7 -------------------------------LDKKLEYLDLEDKLLEVEEARIVKEKEAIAKLNQYQDPENLEFKRLAMEALDLSSKESQL---KQKRKA

R. sibirica 246 -------------------------------LEKKLEYLDLEDKLLEVEEARIVKEKQAIAKLNQYQDPENLEFKRLAMEALDLSSKESQL---KQKRKA

R. africae ESF-5 -------------------------------LDKKLEYLDLEDKLLEVEEARIVKEKQDIAKLNQYQDPENLEFKRLAMEALDLSSKESQL---KQKRKA

R. parkeri PENLEFKRLAMEALDLSSKESQLKQKRKAIELDKKLEYLDLEDKLLEVEEARIVKEKQAIAKLNQYQDPENLEFKRLAMEALDLSSKESQL---KQKRKA

R. heilongjiangensis -------------------------------LDKKLEYLDLEDKLLEVEEARIVKEKQAIATLDQYQDPENPEFKRLAMEALDLSSKESQL---KAKRKT

Israeli tick typhus -----------------------------ALLDRSQGRLNLVD--------RIVKEKEAIAKLNQYQDPENLEFKRLAMEALDLSSKESQL---KQKRKA

Rickettsia sp. A-167 -------------------------------LDKKLEYLDLEDKLLEVEEARIVKEKEAIAKLNQYQDPENLEFKRLAMEALDLSSKESQL---KQKRKA

**[1701-1800 ]**

**[ | | | | | | | | | | ]**

R. bellii RML369-C ---------STNEHSSLGVS-----------------------------------------------------------------NGRED----------

R. bellii OSU 85-389 ---------STNEHSSLGVS-----------------------------------------------------------------NGRED----------

R. prowazekii Madrid E YKQEIN------SINSLDSAVLVESQQFK--KTKSLEDIED-GSLSSQLKQTKYKSVLSPSSYVNDMFSNEHHQNSSIELPKLLHSSLSDTNSSLNDCIN

**R. typhi Wilmington YETHKQ---LSNSI-------------------------------------------RSPKS--------------------------------------**

R. akari Hartford YKTPKK---VTDSINSLDLVVLEESQQLKEPRSNSLEALDLAGSVSS-----------------------------------------------------

R. australis YKTPKK---VTDSINSLDLAVLAESQQLKEPRSKSLEALDLAGSVSSQLKQAEYKSVLSPSI-----------------------NGLEDLSGSNVMKLG

R. felis URRWXCal2 YETPKK---VTDSINSLDLAVPAESQQLKEPRSKSLEALDLAGSVSSQLKQAEYKSVLSPS------------------------SSVNSLLSNEDEQNH

R. canadensis McKiel IDPLFQAAIYSDSELSRELS-----------RSYSV------GDI---LSNTESNLSHSSSV-----------------------SSLNNLSSSNVMKLE

R. helvetica IETLDL---ITPTQNTT-----------------------------------------------------------------------------------

REIS ----------------------------------------------------------------------------------------------------

R. massiliae MTU5 IEAAFS---LNEEYSSTDVSIS---------RSYSI------GDISSLLSDAESNLSRSPSV-----------------------SGLEDLNNSNVMKLG

Candidatus R. amblyommii IEAEFS---LNEKSSSTDVSIL---------RSYSI------DDISGVLSDAESNLSRSPSV-----------------------SGLEDLNNSNVMQLG

R. rhipicephali IEAAFS---LNEEYSSTDVSIS---------RSYSI------GDISSLLSDAESNLSRSPSV-----------------------SGLEDLNNSNVMKLG

R. aeschlimannii REATFS---LNKEYSSTDVSIS---------RSYSI------GDISSLLSDAESNLYCSPSV-----------------------SGLEDLNNSNVMKLG

R. montanensis MEAAFS---LNKEYSSTDVSMS---------RSYSI------GDISSVPSDAESNLSRSPSV-----------------------SGLEDLNNSNVMKLG

R. slovaca 13-B IEAEFS---LNEKSSSTDVSI----------RSYSI------DDISGVLSDAESNLSRSSSV-----------------------SGLEDLNNSNVMQLG

R. rickettsii Iowa IGAEFS---LNEKSSSTDVSI----------RSYSI------DDISSVLSGAESNLSRSPSV-----------------------SGLEDLNN---MQLG

R. rickettsii Sheila Smith IGAEFS---LNEKSSSTDVSI----------RSYSI------DDISSVLSGAESNLSRSPSV-----------------------SGLEDLNN---MQLG

R. japonica IAAEFS---LNEKSSSTDVSIL---------RSYSI------DDISSALSDAESNLSRSPSV-----------------------SGLEDLNNSNVMKLG

R. honei IEAEFS---LNEKSSSTDVSIL---------RSYSI------DDISNVLSDAESNLSHSPSV-----------------------SG-------------

R. conorii str. Malish 7 IEAEFS---LNEKSSSTDVSIL---------RSYSI------DDISGVLSDAESNLSRSPSV-----------------------SGLEDLNNSNVMQLE

R. sibirica 246 IEAEFS---LNEKSSSTDVSIL---------RSYSI------DDISGVLSDAESNLSRSPSV-----------------------SGLEDLNNSNVMQLG

R. africae ESF-5 IEAEFS---LNEKSSSTDVSIL---------RSYSI------DDISGVLSDAESNLSRSPSV-----------------------SGLEDLNNSNVMQLG

R. parkeri IEAEFS---LNEKSSSTDVSI----------RSYSI------DDISGVLSDAESNLSHSPSV-----------------------SGLEDLNNSNVMQLG

R. heilongjiangensis IAAEFS---LNEKSSSTDVSIL---------RSYSI------DDISSVISDAESNLSRSPSV-----------------------SGLEDLNNSNVMKLG

Israeli tick typhus IEAEFS---LNEKSSSTDVSIL---------RSYSI------DDISGVLSDAESNLSRSPSV-----------------------SGLEDLNNSNVMQLG

Rickettsia sp. A-167 IEAEFS---LNEKSSSTDVSIL---------RSYSI------DDISGVLSDAESNLSRSPSV-----------------------SGLEDLNNSNVMQLG

**[1801-1900 ]**

**[ | | | | | | | | | | ]**

R. bellii RML369-C ----------------------------------------------------------------------------------------------------

R. bellii OSU 85-389 ----------------------------------------------------------------------------------------------------

R. prowazekii Madrid E QLRCANTSDVYNLSYTLSNKTKQLSIDELKNTLEQMQTSPNINIVLPMLIRVQQDYVNEVAEIYQQTIEQRKQNPSEQAKNQEEVVAAYFTQEYDKLKII

**R. typhi Wilmington ----------------------------------------------------------------------------------------------------**

R. akari Hartford -----------PDY-----KAKELELDTLN------HDDPKFKKLMLEVFEMSKEKILLEGEIKH-----------------------------------

R. australis KLKSEH-EKITNDY-----KAKELELDTLN------HDDPKFKKLMLEVFEMSKEKIWLEGEIKYLDTESKPKV--TESK--------------------

R. felis URRWXCal2 EFEIP--KSLSFSFGSLNSDAESQQLKEPR------S--------------KSLEALDLAGSVSS-------QLKQAEYK--------------------

R. canadensis McKiel ELKSKY-EKISNDY-----KAKELELDALN------QNDPKFKELMLEVFAMSKDKIWLEGEIKHLETQFEPNIHSAEGK--------------------

R. helvetica ----------------------------------------------------------------------------------------------------

REIS ----------------------------------------------------------------------------------------------------

R. massiliae MTU5 ELESKHDEKIANDY--------------------------------------------------------KAKV--TESK--------------------

Candidatus R. amblyommii ELKSKH-EKIANDY-----N-KELELDTLN------K-----------------EKILLEGKIKDLDTESKPKV--TESK--------------------

R. rhipicephali ELESKHDEKIANDC--------------------------------------------------------KAKV--TESK--------------------

R. aeschlimannii ELKSKH-EKIANDY-----KAKELELDTLN------HDDPEFKKLRLEVFEISKEKIWLEGEIKHLDTESKPKV--NESK--------------------

R. montanensis ELKSKH-EKIANDY-----KAKELELDTLN------HDDPKFKKLKLEVFEILKEKIWLEGEIKHLDTESKPKV--TESK--------------------

R. slovaca 13-B ELKSKH-EKIANDY-----N-KELELDTLN------K-----------------EKILLEGKIKHLDTESKPKV--TESK--------------------

R. rickettsii Iowa ELKSK----------------KELELDTLN------K-----------------EKIWLD-----LDTESKPKV--TESK--------------------

R. rickettsii Sheila Smith ELKSK----------------KELELDTLN------K-----------------EKIWLD-----LDTESKPKV--TESK--------------------

R. japonica ELKSKH-KKIANDY-----KAKELELDTLN------H--------------DDPEKIWLEGEIKHLDTESKPKV--NESK--------------------

R. honei -------------------------LDNLN------K-----------------EKIWLEGKIKHLDTESKPKV--TESK--------------------

R. conorii str. Malish 7 ELKSKH-EKIANDY-----N-KELELDTLN------K-----------------EKIWLEGEIKHLDTEFKPKV--TESK--------------------

R. sibirica 246 ELKSKH-EKIANDY-----N-KELELDTLN------K-----------------EKILLEGKIKHLDTESKPEV--TESK--------------------

R. africae ESF-5 ELKSKH-EKIANDY-----N-KELELDTLN------K-----------------EKILLEGKIKHLDTESKPKV--TESK--------------------

R. parkeri ELKSKH-EKIANEY-----N-KELELDTLN------K-----------------EKILLEGKIKHLDTESKPKV--TESK--------------------

R. heilongjiangensis ELKSKH-KKIANDY-----KAKELELDTLN------H-----------DDP---EKIWLEGEIKHLDTESKPKV--TESK--------------------

Israeli tick typhus ELKSKH-EKIANDY-----N-KELELDTLN------K-----------------EKIWLEGEIKHLDTESKPKV--TESK--------------------

Rickettsia sp. A-167 ELKSKH-EKIANDY-----N-KELELDTLN------K-----------------EKIWLEGEIKHLDTESKPKV--TESK--------------------

**[1901-2000 ]**

**[ | | | | | | | | | | ]**

R. bellii RML369-C ------------KPSSSGLIDGGKSEHRL------------------------------LGVSDGKEEEDRFLGLSDG----------------------

R. bellii OSU 85-389 ------------KPSSSGLIDGGKSEHRL------------------------------LGVSDGKEEEDRFLGLSDG----------------------

R. prowazekii Madrid E NSFRSLKTDSITNTSLASSVNIFGSNEDLS-------------------------WDNTGDIGVGYRYGPEHSCPTPD----------------------

**R. typhi Wilmington --------DLTTNSSLASSLNIFGSNEDLS-------------------------WDNAGDIGVGYRYGDEYSCPTPD----------------------**

R. akari Hartford -----------------------------------------------------------LDTESNKGSVYTYIKVLNN----------------------

R. australis --------PVFLRSSSASSLNSLYNGHDLS-------------------SRDVVPPVDTLTIETNKGSVDTYIRVLSN----------------------

R. felis URRWXCal2 --------SVLSPSSSVNSL--FSNEDEQNHEFEILKSLSFSFGSLNSDAESNLSWDNTGDIGVGHRYGAEDSCPTPDQSSSKIANSSDKNSSETKLALL

R. canadensis McKiel --------PVFSPD----SINSVFSGVDY------------------------------INTNDPTKLIENKIAALRI----------------------

R. helvetica --------------------------------------------------------------------------VAKN----------------------

REIS -------------------------------------------------------------IENDKDYVDTYIRVLSN----------------------

R. massiliae MTU5 --------PVFSCSSSVGSINSLSNGDDLS-------------------SRDVVTPVDTLTIEIDKDYVDTYIRVLSN----------------------

Candidatus R. amblyommii --------PVFSCSSSVGSIHSFSNDDDLS-------------------SRDVVTPVDTLNIEINKDYVDTYIRVLSN----------------------

R. rhipicephali --------PVFSCSSSVGSINSLSNGDDLS-------------------SRDVVTPVDTLTIEIDKDYVDTYIRVLSN----------------------

R. aeschlimannii --------PVFSCSSSVGGINSLSNGDDLS-------------------SRDVVTPVDTLTIEIDKDYVDTYIRVLSS----------------------

R. montanensis --------PVFSCSSSVGSINSLSNGDDLS-------------------SRDV-------TIEIDKDYVDTYIRVLSN----------------------

R. slovaca 13-B --------PVFSCSSSVGSINSFSN-DDLS-------------------SRDVVTPVDTLNIEINKDYVDTYIRVLSN----------------------

R. rickettsii Iowa --------PVFSCSSSVGSINSFSN-DDLS-------------------SRDVVAPVDTLNIEINKDYVDTYIRVLSN----------------------

R. rickettsii Sheila Smith --------PVFSCSSSVGSINSFSN-DDLS-------------------SRDVVAPVDTLNIEINKDYVDTYIRVLSN----------------------

R. japonica --------PVFSCSSSVGSINSFSNGDNLS-------------------SRDVVTSVDTLTREIDKDYVDTYIRVLSN----------------------

R. honei --------PVFSCSSSVGSINSFSNDDDLS-------------------SRDVVTPVDTLNIEINKDYVDTYIRVLSN----------------------

R. conorii str. Malish 7 --------PVFSCSSSVGSINSFSNDDDLS-------------------SRDVVTPVDTLNIEINKDYVDTYIRVLSN----------------------

R. sibirica 246 --------PVFSCSSSVGSINSFSNDDDLS-------------------SRDVVTPVDTLNIEINKDYVDTYIRVLSN----------------------

R. africae ESF-5 --------PVFSCSSSVGSIHSFSNDDDLS-------------------SRDVVTPVDTLNIEINKDYVDTYIRVLSN----------------------

R. parkeri --------PVFSCSSSVGSINSFSNDDNLN-------------------SRDVVTPVDTLNIEINKDYVDTYIRVLSN----------------------

R. heilongjiangensis --------PVFSCSSSVGSINSFSNGDDLS-------------------SRDVVTSVDTLTREIDKDYVDTYIRVLSN----------------------

Israeli tick typhus --------PVFSCSSSVGSINSFSNDDDLS-------------------SRDVVTPVDTLNIEINKDYVDTYIRVLSN----------------------

Rickettsia sp. A-167 --------PVFSCSSSVGSINSFSNDDDLS-------------------SRDVVTPVDTLNIEINKDYVDTYIRVLSN----------------------

**[2001-2100 ]**

**[ | | | | | | | | | | ]**

R. bellii RML369-C ----------REDKPSSSGSSDGGK-------------------------------SEYSLLGVSDGKEEEDRFLGLS----------DGREDKPSSLRL

R. bellii OSU 85-389 ----------REDKPSSSGSSDGGK-------------------------------SEYSLLGVSDGKEEEDRFLG------------------------

R. prowazekii Madrid E ----------DRLSSKLIDMSNKNT------KNVKGLEEGVTIYDSIDSVADPLKSNDVSFEEINEALKQLSAAISETVINSSESKVQEDPEDILDGSIL

**R. typhi Wilmington ----------DKSSSKLIDISDKNT------TKVKGLEEGITMYDSIDLVADSLKSNAVSFKERNEALKQLSTAISETVISLSESNVQEDAEDILDGSIL**

R. akari Hartford ---K------IQTMEELLGSSSRSE-------ELTHIKEAM-AYIS-NSIQDVEELDERMLLAINARLQEHDKKISSLLLEKYTS---EEAEDIFAQSLL

R. australis ---K------IQKMEELLGSSSSSE-------ELTHIKEAI-AYIS-NSIHDVNELDARMLLAINAQLQEHDEKISSLLLEQYTS---EEAEDIFAQSLL

R. felis URRWXCal2 RTYQMAVIVKRQELEQLPSTSSGADQNTQKIKELKEVEVTMHDAIN-ACIADSSKLNDVSFEEINEALKKLGSEIPESAISDFESKAQKDPEAILDGSIL

R. canadensis McKiel --YQMAVIVKRQELEQLPSTSSGADQTAQQIKKLQEKEVIMHDAIN-ACIADPSKLNNVSFEEIKEALKKLSAGIPESVINVLESKAQEDPEDILNGLTL

R. helvetica ---D------SSRKSNVSG-------------SIPEIQQPQ---------------SEKMLLAINARLQEYDEKISSLLLEKYTS---EEAEDILAQLLL

REIS ---K------IQKIEELPGSSSRSE-------ELNHIKEAM-AYIS-DRIQDVEELDKNMLLAINALLQEHDEKISSLLLEKYTS---EEAEDILAQSLL

R. massiliae MTU5 ---K------IQKIEELPGSSSRSE-------ELNHIKEAM-AYIS-NRIQDVEELDEKMLLAMNARLQEHDEKISSLLLEKYTG---EEAADILAQLLL

Candidatus R. amblyommii ---KIQKIEEIQKIEELSGSSSRSE-------ELNHIKEAM-AYIS-NRIQDVEELDEKMLLAINAQLQEHDEKISSXL-----R---XXATDILAQLLL

R. rhipicephali ---K------IQKIEELPGSSSRSE-------ELNHIKEAM-AYIS-NRIQDVEELDEKMLLAINARLQEHDEKISSLLLEKYTG---EEAADILAQLLL

R. aeschlimannii ---K------IQKIEELPGSSSVSE-------ELNHIKETM-AYIS-NRIQDVEELDEKMLLAINARLQEHDEKIYSLLLEKYTG---EEAADILAQSLL

R. montanensis ---K------IQKIEELPGSSSRSE-------ELNHIKEAM-AYIS-NRIQDVEELDEKMLLAINARLQEHDEKISSLLLEKYKG---EEAADILAQLLL

R. slovaca 13-B ---K------IQKIEELSGSSSRSE-------ELNHIKEAM-TYIS-NRIQDVEELDEKMLLAINAQLQEHDEKISSLL-----R---EEATDILAQLLL

R. rickettsii Iowa ---K------IQEIEELSGSSSRSE-------ELNHIKEAM-AYIS-NRIQDVEELDEKMLLVINSQLQEHDEKISSLL-----R---EEATDILAQLLL

R. rickettsii Sheila Smith ---K------IQKIEELSGSSSRSE-------ELNHIKEAM-AYIS-NRIQDVEELDEKMLLVINSQLQEHDEKISSLL-----R---EEATDILAQLLL

R. japonica ---K------IQKIEELPGSSSRSE-------ELNHIKEAM-AYIS-NRIQDVEELDEKMLLAINARLQEHDEKISSLLLEKYTG---EEAADILAQSLL

R. honei ---K------IQEIEELSGSSSR-E-------ELNHIKEAM-AYIS-NRIQDVEELDEKVLLAINAQLQEHDKKISSLL-----R---EEATDILAQLLL

R. conorii str. Malish 7 ---KIQKIEEIQKIEELSGSSSRSE-------ELNHIKEAM-AYIS-NRIQDVEELDEKMLLAINAQLQEHDEKISSLL-----R---EEATDILAQLLL

R. sibirica 246 ---K------IQKIEELSGSSSRSE-------ELNHIKEAM-AYIS-NRIQDVEELDEKMLLAINAQLQEHDEKISSLL-----R---EEATDILAQLLL

R. africae ESF-5 ---KIQKIEEIQKIEELSGSSSRSE-------ELNHIKEAM-AYIS-NRIQDVEELDEKMLLAINAQLQEHDEKISSLL-----R---EEATDILAQLLL

R. parkeri ---K------IQKIEELSGSSSRSE-------ELNHIKEAM-VYIS-NRIQDVEELDEKMLLAINAQLQEHDEKISSLL-----R---EEATDILAQLLL

R. heilongjiangensis ---K------IQGIEELPGSSSRSE-------ELNHIKEAM-AYIS-NRIQDVEELDEKMLLAINARLQEHDEKISSLLLEKYTG---EEAADILAQSLL

Israeli tick typhus ---KIQKIEEIQKIEELSGSSSRSE-------ELNHIKEAM-AYIS-NRIQDVEELDEKMLLAINAQLQEHDEKISSLL-----R---EEATDILAQLLL

Rickettsia sp. A-167 ---KIQKIEEIQKIEELSGSSSRSE-------ELNHIKEAM-AYIS-NRIQDVEELDEKMLLAINAQLQEHDEKISSLL-----R---EEATDILAQLLL

**[2101-2200 ]**

**[ | | | | | | | | | | ]**

R. bellii RML369-C SD---------------------------------GGKSEHSLLGVSDGK--EEEDRFLGLSDGSDDKHGLLALSDGRENKNDSLKLKDGDDKRSFLRLK

R. bellii OSU 85-389 ---LSD-----------------------------GGKSEHSLLGVSDGK--EEEDRFLGLSDGSDDKHGLLALSDGRENKNDSLKLKDGDDKRSFLRLK

R. prowazekii Madrid E QAMLNSN----------------------------NDQSIESLSLGSEEEQGQEETEA---SIQNAGDKKLLPVPA--SSNESTLLLSD-CKKMNI----

**R. typhi Wilmington QAMLNSN----------------------------NDKSIESLSLGSKE---QEETEA---GIRKAGDKQLLPVPVL---------LSD-CKENEYLSLD**

R. akari Hartford QEMLVS-----------------------------DCESESTLPAGHEE---KEDTEVTESGIQKAGDKELLALHV--SSNECALALDD-DREKESLELG

R. australis QEMLVS-----------------------------DGKSESTLPVGHEE---LEDTEVTELGIQKAGDTGLLALHV--SSNECALALSD-DREKECLELG

R. felis URRWXCal2 QEMLSS---------------------------------KDSLPSGDEE---QEDTEVTESGIQKAGDKGLLALPV--SSNECALALSD-GREKECLALG

R. canadensis McKiel QEMLSSN----------------------------NDTTIDSLPSGKEQS--SEDEEVTKAGIQEVEDKQLLALPV--SSNEYALALSD-DSEKECLVLS

R. helvetica QEMLVS-----------------------------DGKSESTLPAGDEE---QEDTEV------TDVSRELSLLPALASSNESALALSD-SREKECLALG

REIS QEMLVS-----------------------------DGKSESTLPTGDEE---QEDTEV------TDVSGKLSLLPV--SSNEYALALSD-GSE-------

R. massiliae MTU5 QDMLVS-----------------------------DGKSESTLPAGDEE---QEDTEV------TEVSRQLSSLPALASSNECALALSD-DREKECLALG

Candidatus R. amblyommii QEMLVS-----------------------------DGKSESTLPAGDEE---QEDTEV---------SRQLSSLPALASSNESALALSD-DREKECLALG

R. rhipicephali QDMLVS-----------------------------DGKSESTLPAGDEE---QEDTEV------TEVSRQLSSLPALASSNESALALSD-DREKECLALG

R. aeschlimannii QEMLVS-----------------------------DGKSESTLPAGDEE---QEDTEV---------SRQLSSLPALASSNECALALSD-DREKECLALG

R. montanensis QDMLAS-----------------------------DGKSESTLPAGDKE---QEDTEDTEDTEVKEVSRQLSSLPALASSNECALALSD-DREKECLALG

R. slovaca 13-B QEMLVS-----------------------------DGKSESTLPAGDEE---QEDTEV---------SRQLSSLPALASSNESALALSD-DREKECLALG

R. rickettsii Iowa QEMLVSDGKSESTLPAGDEEQEDTEVSRQLSSLPADGKSESTLPAGDEE---QEDTEV---------SRQLSSLPALASSNESALALSD-DREKECLALG

R. rickettsii Sheila Smith QEMLVSDGKSESTLPAGDEEQEDTEVSRQLSSLPADGKSESTLPAGDEE---QEDTEV---------SRQLSSLPALASSNESALALSD-DREKECLALG

R. japonica QEMLVS-----------------------------DGKSESILPAGDEE---QEDTEV---------SRQLSSLPALASSNECALALSD-DREKECLALG

R. honei QEMLVS-----------------------------DGKSESTLPAGDEE---QEDTEV---------SRQLSSLPALASSNESALALSD-DREKECLALG

R. conorii str. Malish 7 QEMLVS-----------------------------DGKSESTLPAGDEE---QEDTEV---------SRQLSSLPALASSNESALALSD-DREKECLALG

R. sibirica 246 QEMLVS-----------------------------DGKSESTLPAGDEE---QEDTEV---------SRQLSSLPALASSNESALALSD-DREKECLALG

R. africae ESF-5 QEMLVS-----------------------------DGKSESTLPAGDEE---QEDTEV---------SRQLSSLPALASSNESALALSD-DREKECLALG

R. parkeri QEMLVS-----------------------------DGKSESTLPAGDEE---QEDTEV---------SRQLSSLPALASSNESALALSD-DREKECLALG

R. heilongjiangensis QEMLVS-----------------------------DGKSESILPAGDEE---QEDTEV---------NRQLSSLPALASSNESALALSD-DREKECLALG

Israeli tick typhus QEMLVS-----------------------------DGKSESTLPAGDEE---QEDTEV---------SRQLSSLPALASSYESALALSD-DREKECLALG

Rickettsia sp. A-167 QEMLVS-----------------------------DGKSESTLPAGDEE---QEDTEV---------SRQLSSLPALASSNESALALSD-DREKECLALG

**[2201-2300 ]**

**[ | | | | | | | | | | ]**

R. bellii RML369-C SGEKDEDSFLKLI------------------DSDDDYDSGIEEDLE----------YSKPLKKIDAITTIPLPQ-AVEEVKKSVAAIAPTTNQQIQTVQK

R. bellii OSU 85-389 SGEKDEDSFLKLI------------------DSDDDYDSGIEEDLE----------YSKPLKKIDAITTIPLPQ-AVEEVKKSVAAIAPTTNQQIQTVQK

R. prowazekii Madrid E ------------------------------------------------------------YHMNKVDTTILLEQEAKQEMQTQISENAPTLNQ-VKVINT

**R. typhi Wilmington NYNKGEKN----------------------------YDSDFKEEEE--TIEQLSNSD--WYKMTKADTAILLEQEAKQEMQTQISENAPTLNQ-AKVINT**

R. akari Hartford GGSEDEER----------------------------YDSGFEEEEE--IIEQLSDSDGGNIKITEVDMAIPLEQKAKQEMQTQISENAPTLNQ-AKVVNT

R. australis DGSEDEEG----------------------------YDSGFEEEAE--IIEQLSDYDGDNLKITEVDTAIPLEQKAKKEMQTQISENAPTLNQ-AKVVNT

R. felis URRWXCal2 DGSEEKE--------------------------------------E--TIEQLSDSVEGNLKI---------EQEEKKEMQTQISKNAPTLNQ-AKVVNT

R. canadensis McKiel DGSEDEDD---------------------------DNDSGIDSESEEGVVDQLLDSREDNLKTTEVNTVISLKQEVIEETKKSIAAIAPTLNQGVQVVKN

R. helvetica DGSGDEES----------------------------YDSGFEAEEE--TIEQLSDSDGGNLKMTEVDTAIPLEQEAKQEMQMQISENAPTLNQ-AKVVNT

REIS -YEEDEES----------------------------YDSGFEEEKE--TIEQLSDSDGGNLKITEVDTAVPLEQKAKKEMQTQILENAPTLNQ-AKVVNT

R. massiliae MTU5 DGSEDKES----------------------------YDSGFEEEEE--TIGQLSDSDGGNLKITEVDTAIPLEQEAKKEMQTQISENAPTLNQ-VKVVNT

Candidatus R. amblyommii DSSEDEES----------------------------YDSEFEEEEE--TIGQLSDSDGGNLKITEVDTVIPLEQEAKKEMQTQISENAPTLNQ-AKVVNT

R. rhipicephali DGSEDEES----------------------------YDSGVEEEEE--TIGQLSDSDGGNLKITEVDTAIPLEQEAKKEMQTQISENAPTLNQ-VKVVNT

R. aeschlimannii DGSEDEES----------------------------YDSGFEEEEE--TIGQLSDSDGGNLKIMEVDTAIPLEQEAKKEMQTQISESAPTLNQ-AKVVNT

R. montanensis DGSEDEES----------------------------YDSGFEEEEE--TIGQLSDSDGGNLKITEVDTAIPLEQEAKKEMQTQISENAPTLNQ-AKVVNT

R. slovaca 13-B DGSEDEES----------------------------YDSGFEEEEE--TIGQLSDSDGGNLKITEVDTVIPLEQEAKKEMQTQISENAPTLNQ-AKVVNT

R. rickettsii Iowa DGSEDEES----------------------------YDSGFEEEEE--TIGQLSDSDGGNLKITEVDKVIPLEQEAKKEMQMQISENAPTLNQ-AKVVNT

R. rickettsii Sheila Smith DGSEDEES----------------------------YDSGFEEEEE--TIGQLSDSDGGNLKITEVDKVIPLEQEAKKEMQMQISENAPTLNQ-AKVVNT

R. japonica DGSEDEESSNECALALSDDREKECLALGDGSEDEESYDSGFEEEEE--TIGQLSDSDGGNLKITEVDTAIPLEQEAKKEMQTQISENAPTLNQ-AKVVNT

R. honei DGSEDEES----------------------------YDSGFEEEEE--TIGQLSDSDGGNLKITEVDTVIPLEQEAKKEMQTQISENAPTLNQ-AKVVNT

R. conorii str. Malish 7 DSSEDEES----------------------------YDSGFEEEEE--TIGQLSDSDGDNLKITEVDTVIPLEQEAKKEMQTQISENAPTLNQ-AKVVNT

R. sibirica 246 DSSEDEES----------------------------YDSGFEEEEE--TIGQLSDSDGGNLKITEVDTVIPLEQEAKKEMQTQISENAPTLNQ-AKVVNT

R. africae ESF-5 DSSEDEES----------------------------YDSEFEEEEE--TIGQLSDSDGGNLKITEVDTVIPLEQEAKKEMQTQISENAPTLNQ-AKVVNT

R. parkeri DSSEDEES----------------------------YDSGFEEEEE--TIGQLSDSDGGNLKITEVDTVIPLEQEAKKEMQTQISENAPTLNQ-AKVVNT

R. heilongjiangensis DGSEDEES----------------------------YDSGFEEEEE--TIGQLSDSDGGNLKITEVDTAIPLEQEAKKEMQTQISENAPTLNQ-AKVVNT

Israeli tick typhus DSSEDEES----------------------------YDSGFEEEEE--TIGQLSDSDGGNLKITEVDTVIPLEQEAKKEMQTQISENAPTLNQ-AKVVNT

Rickettsia sp. A-167 DSSEDEES----------------------------YDSGFEEEEE--TIGQLSDSDGGNLKITEVDTVIPLEQEAKKEMQTQISENAPTLNQ-AKVVNT

**[2301-2400 AT Domain ]**

**[ | | | | | | | | | | ]**

R. bellii RML369-C VTKDSIFTRLDSIAVVKINEDNNVAIAAGDEE-**SPVKRGLWMRTMYGVNNQGRVNNINGYRGINKGGTVGFDVEIDNNIIGIAYSNVHSVFKFKNNNNND**

R. bellii OSU 85-389 VTKDSIFTRLDSIAVVKINEDNNAAIAAGDEE-**SPVKRGLWMRTMYGVNNQGRVNNINGYRGINKGGTVGFDVEIDNNIIGIAYSNVHSVFKFKNNNNND**

R. prowazekii Madrid E IVNNMIRNRLDTSI-------NMVVVGASAEEE**SNIKRGLWMRGMYGTNNHGRVDNMTGYRGINKGATVGFDAEINNNIIGIAYSNVHSVFKFKNGKNND**

**R. typhi Wilmington IVNNMIRNRLDTFI-------NMVVVGAGDEEESNIKRGLWMRAMYGTNNHGRVNNMTGYRGINKGATVGFDAKINNNIIGIAYSNVHSVFKFKNSKNND**

R. akari Hartford IVNNMIRNRLDTSMNI---ANNIVAVGAGDEEE**SRITRGLWMRGMYGTNNHGRVENMNGYRGTNKGATIGFDVEIDNNILGIAYSNVHSVFKFKNSKDND**

R. australis IVNNMIRNRLDASMNM---ANNIVAVGAGDEEE**SCIKRGLWMRGMYGTNNHGRVENMNGYRGTNKGATIGFDVEIDNNILGIAYSNVHSVFKFKNSKNND**

R. felis URRWXCal2 IVNNMIRNRLDASMNM---SNNMVAVGAGDEEE**SRIKRGLWMRGMYGTNNHGRVENMTGYRGTNKGATIGFDVEIDNNILGIAYSNVHSVFKFKNSKNND**

R. canadensis McKiel ISKSILGARLDSSE-------ALTAVAAGDEEE**SRIKRGLWMRGMYGTNNHGRVNNMTGYRGINKGTTIGFDFEIDNNIVGIAYSNVYSVFKFKNSKNND**

R. helvetica IVNNMIRNRLDASMNM---SNNMVAVGAGDEED**-RIKRGLWMRGMYGTNNHGRVDNMTGYRGTNKGATIGFDVEIDNNIVGIAYSNVHSVFKFKNSKNND**

REIS IVNNMIRNRLDASM-------NMVAVGAGDEEE**SRIKRGLWMRGMYGVNNHGRVDNMTGYRGTNKGATIGFDVEIDNNILGIAYSNVHSVFKFKNSKNND**

R. massiliae MTU5 IVNNMIRNRLDASMNM---SNNMVAVGAGDEEE**SHIKRGLWMRGMYGTNNHGRVENMTGYRGTNKGATIGFDAEIDNNIVGIAYSNVHSVFKFKNSKNND**

Candidatus R. amblyommii IVNNMIRNRLDASMNM---SNNMVTVGAGDEEE**SNIKRGLWMRGMYGTNNHGRVENMTGYRGTNKGATIGFDAEIDNNIVGIAYSNVHSVFKFKNSKNND**

R. rhipicephali IVNNMIRNRLDASMNM---SNNMVAVGAGDEEE**SHIKRGLWMRGMYGTNNHGRVDNMTGYRGTNKGATIGFDAEIDNNIVGIAYSNVHSVFKFKNSKNND**

R. aeschlimannii IVNNMIRNRLDTSMNM---SNNMVAVGAGDEEE**SHIKRGLWMRGMYGTNNHGRVDNMTGYRGTNKGATIGFDAEIDNNIVGIAYSNVHSVFKFTNSKNND**

R. montanensis IVNNMIRNRLDASMNM---SNNMVAVGAGDEEE**SHIKRGLWMRGMYGTNNHGRVDNMTGYRGTNKGATIGFDAEIDNNIVGIAYSNVHSVFKFKNSKNND**

R. slovaca 13-B IVNNMIRNRLDASMNM---SNNMVAVGAGDEEE**SHIKRGLWMRGMYGTNNHGRVENMTGYRGTNKGATIGFDAEIDNNIVGIAYSNVHSVFKFKNSKNND**

R. rickettsii Iowa IVNNMIRNRLD--VNM---SNNM-AVGAGDEEE**SHIKRGLWMRGMYGTNNHGRVENMTGYRGTNKGATIGFDAEIDNNIVGIAYSNVHSVFKFKNSKNND**

R. rickettsii Sheila Smith IVNNMIRNRLD--VNM---SNNM-AVGAGDEEE**SHIKRGLWMRGMYGTNNHGRVENMTGYRGTNKGATIGFDAEIDNNIVGIAYSNVHSVFKFKNSKNND**

R. japonica IVNNMIRNRLDASMNM---SNNMVAVGAGDEEE**SHIKRGLWMRGMYGTNNHGRVENMTGYRGTNKGATIGFDAEIDNNIVGIAYSNVHSVFKFKNSKNND**

R. honei IVNNMIRNRLDASMNI---SNNMVAVGAGDEEE**SHIKRGLWMRGIYGTNNHGRVENMTGYRGTNKGATIGFDAEIDNNIVGIAYSNVHSVFKFKNSKNND**

R. conorii str. Malish 7 IVNNMIRNRLDASMNM---SNNMVAVGAGDEEE**SHIKRGLWMRGMYGTNNHGRVENMTGYRGTNKGATIGFDAEIDNNIVGIAYSNVHSVFKFKNSKNND**

R. sibirica 246 IVNNMIRNRLDASMNM---SNNMVAVGAGDEEE**SHIKRGLWMRGMYGTNNHGRVENMTGYRGTNKGATIGFDAEIDNNIVGIAYSNVHSVFKFKNSKNND**

R. africae ESF-5 IVNNMIRNRLDASMNM---SNNMVTVGAGDEEE**SNIKRGLWMRGMYGTNNHGRVENMTGYRGTNKGATIGFDAEIDNNIVGIAYSNVHSVFKFKNSKNND**

R. parkeri IVNNMIRNRLDASMNM---SNNMVAVGAGDEEE**SHIKRGLWMRGMYGTNNHGRVENMTGYRGTNKGATIGFDAEIDNNIVGIAYSNVHSVFKFKNSKNND**

R. heilongjiangensis IVNNMIRNRLDASMNM---SNNMVAVGAGDEEE**SHIKRGLWMRGMYGTNNHGRVENMTGYRGTNKGATIGFDAEIDNNIVGIA-----------------**

Israeli tick typhus IVNNMIRNRLDASMNM---SNNMVAVGAGDEEE**SHIKRGLWMRGMYGTNNHGRVENMTGYRGTNKGATIGFDAEIDNNIVGIAYSNVHSVFKFKNSKNND**

Rickettsia sp. A-167 IVNNMIRNRLDSSMNM---SNNMVAVGAGDEER**SHIKRGLWMRGMYGTNNHGRVENMTGYRGTNKGATIGFDAEIDNNIVGIAYSNVHSVFKFKNSKNND**

**[2401-2500 ]**

**[ | | | | | | | | | | ]**

R. bellii RML369-C **KEIIKSHIVSIYGQKELPKNFVLQGLVSASKNFIKNKTTYLFNNTKFKSNVKHRNHSYNAEALLNYNYLATNNIVITPNIGLRYGKSRDGVYNETGISIQ**

R. bellii OSU 85-389 **KEIIKSHIVSIYGQKELPKNFVLQGLVSASKNFIKNKTTYLFNNTKFKSNVKHRNHSYNAEALLNYNYLATNNIVITPNIGLRYGKSRDGVYNETGISIQ**

R. prowazekii Madrid E **KELIGSKVISIYGQKELQQNFTLQLLVSASKNFIKDKINYSYGDTQIKSHVKHHNHSYNAEALLNYNYLVKNSIIITPNIGLKYGKSRDGVYNETGINVQ**

**R. typhi Wilmington KELIGSKVISIYGQKELQQNFTLQVLVSASKNFIKDKINYSYGDTKIRSNVKHHNHSYNAEALLNYNYLVKNSIIVTPNIGLRYGKSRDGIYNETGINVQ**

R. akari Hartford **KELIDSHVISIYGQKELPRNFALQALVSASKNFIKDKTTYSYGDTKIGSNVRHRNHSYNAEALLNYNYLLQNKCVITPNIGLRYGKSRDGVYNETGINVQ**

R. australis **KELIDSHVISIYGQKELPRNFALQALVSASKNFIKDKTTYSYGDTKIGSNVKHRNHSYNAEALLNYNYLLQNKFVITPNIGLRYGKSRDGVYNETGINVQ**

R. felis URRWXCal2 **KELIDSHVVSIYGQKELPRNFALQALVSASKNFIKDKTTYSYGDTKIRSNVKHSNHSYNAEALLNYNYLLQNKLVITPNIGLRYGKSRDGVYNETGINVQ**

R. canadensis McKiel **KEFINSHVVSIYGQKELPKNFVAQALVSASKNFIKDKTTYSYGDTNIRSNVKHRNHSYNAEALLNYNYLVKNNLVITPNIGLRYGKSRDGVYNEIGINVQ**

R. helvetica **KELINSHVVSIYGQKELPKNFALQALVSASKNFIKDKTTYSYGDTKIRSNVKHRNHSYNAEALLNYNYLLQNKLVIIPNVGLRYGKSRDGVYNETGINIQ**

REIS **KELINSHVVSIYGQKELPRNFALQALVSASKNFIKDKTTYSYGDTKIRSNVKHRNHSYNAEALLNYNYLLQNKLVIIPNIGLRYGKSRDGVYNETGINVQ**

R. massiliae MTU5 **KELINSHVVSIYGQKELPKNFALQALVSASKNFIKDKTTYSYGDTKIRSNVKHRNHSYNAEALLHYNYLLQSKLIITPNIGLRYGKSRDGVYHETGINVQ**

Candidatus R. amblyommii **KELINSHVVSIYGQKELPKNFALQALVSASKNFIKDKTTYSYGDTKIKSNVKHRNHSYNVEALLHYNYLLQSKFIITPNIGLRYGKSRDGVYNETGVNVQ**

R. rhipicephali **KELINSHVVSIYGQKELPKNFALQALVSASKNFIKDKTPYSYGDTKIRSNVKHRNHSYNAEALLHYNYLLQSKLIITPNIGLRYGKSRDGVYHETGINVQ**

R. aeschlimannii **KELINSHVVSIYGQKELPKNFALQALVSASKNFIKDKTTYSYGDTKIRSNVKHRNHSYNAEALLNYNYLLQSKLVITPNIGLRYGKSRDGVYHETGINVQ**

R. montanensis **KELINSHVVSIYGQKELPKNFALQALVSASKNFIKDKTTYSYGDTKIRSNVKHRNHSYNVEALLNYNYLLQSKLLITPNIGLRYGKSRDGVYNETGINVQ**

R. slovaca 13-B **KELINSHVVSIYGQKELPKNFALQALVSASKNFIKDKTTYSYGDTKIKSNVKHRNHSYNAEALLHYNYLLQSKLVITPNIGLRYGKSRDGVYNETGVNVQ**

R. rickettsii Iowa **KELINSHVVSIYGQKELPKNFALQALVSASKNFIKDKTTYSYGDTKIKSNVKHRNHSYNAEALLHYNYLLQGKLVITPNIGLRYGKSRDGVYNETGVNVQ**

R. rickettsii Sheila Smith **KELINSHVVSIYGQKELPKNFALQALVSASKNFIKDKTTYSYGDTKIKSNVKHRNHSYNAEALLHYNYLLQGKLVITPNIGLRYGKSRDGVYNETGVNVQ**

R. japonica **KELINSHVVSIYGQKELPKNFALQALVSASKNFIKDKTTYSYGDTKIRSNVKHRNNSYNAEALLQYNYLLQSKFVITPNIGLRYVKSRDGVYNEIGVNVQ**

R. honei **KELI-IVMWFLFTDKKNYRRICIQALVSASKNFIKDKTTYSYGDTKIRSNVKHRNHSYNAEALLHYNYLLQSKLVITPNIGLRYGKSRDGVYNETGVNVQ**

R. conorii str. Malish 7 **KELINSHVVSIYGQKELPKNFALQALVSASKNFIKDKTTYSYGDTKIKSNVKHRNHSYNAEALLHYNYLLQSKLVITPNIGLRYGKSRDGVYNETGVNVQ**

R. sibirica 246 **KELINSHVVSIYGQKELPKNFALQALVSASKNFIKDKTTYSYGDTKIKSNVKHRNHSYNAEALLHYNYLLQSKFVITPNIGLRYGKSRDGVYNETGVNVQ**

R. africae ESF-5 **KELINSHVVSIYGQKELPKNFALQALVSASKNFIKDKTTYSYGDTKIKSNVKHRNHSYNVEALLHYNYLLQSKFVITPNIGLRYGKSRDGVYNETGVNVQ**

R. parkeri **KELINSHVVSIYGQKELPKNFALQALISASKNFIKDKTTYSYGDTKIKSNVKHRNHSYNVEALLHYNYLLQSKFVITPNIGLRYGKSRDGVYNETGVNVQ**

R. heilongjiangensis **----------------------------------------------------------------------------------------------------**

Israeli tick typhus **KELINSHVVSIYGQKELPKNFALQALVSASKNFIKDKTTYSYGDTKIKSNVKHRNHSYNAEALLHYNYLLQSKLVITPNIGLRYGKSRDGVYNETGVNVQ**

Rickettsia sp. A-167 **KELINSHVVSIYGQKELPKNFALQALVSASKNFIKDKTTYSYGDTKIKSNVKHRNHSYNAEALLHYNYLLQSKLVITPNIGLRYGKSRDGVYNETGVNVQ**

**[2501-2600 ]**

**[ | | | | | | | | | | ]**

R. bellii RML369-C **EIALATKENNILSGIIGAKTKIPLKN----NNLGLTLHSSIEHNFNEKTQRVNRTIQIQGN**KFTQNHIIPKQAKTAYNLGGGIIGSVKNTIISLDYNYYL

R. bellii OSU 85-389 **EIALATKENNILSGIIGAKTKIPLKN----NNLGLTLHSSIEHNFNEKTQRVNRTIQIQGN**KFTQNHIIPKQAKTAYNLGGGIIGSVKNTIISLDYNYYL

R. prowazekii Madrid E **EIALTMKENNILSGILGAKVKVSLIDVLKFNNLGLTFQTAVEHNFKEKTQRINRVIKIFDN**IFKQDYLIPKQPKTSYNLGTGIICGINNTNIAVDYNYYL

**R. typhi Wilmington KIALTMKENNILSGILGTKVQVSLRDVLKFNNLGLTFQAAVEHNFEEKTQRINRVVKIFDNIFKQDYLIPKQPKTSYNLGTGIIFGINNTNISVDYNYYL**

R. akari Hartford **EIALTMKENNILSGIVGTKVKVPLKDVLKFNNLGITFQGAVEHTFKEKTQRINRVVKIFDH**KFKQDYLIPKQPKTSYNLGTGIIGSIKNTTISLDYNYYL

R. australis **EIALTMKENNILSGIVGTKVKVPLKDALKFNNLGLTFQGAVEHTFKEKTQRINRVVKIFDN**TFKQDYLIPKQPKTSYNLGTGIIGSIKNTTISLDYNYYL

R. felis URRWXCal2 **EIALTMKENNILSGIVGTKVKVPLKDVLKFNNLGLTFQGAVEHNFKEKTQRINRVVKIFDT**EFKQNYAIPKQPKTSYNLGTGIIGSIKNTTISLDYNYYL

R. canadensis McKiel **EIALTMKENNILSGIIGTKVKVPLKDVLKFNNLGLIFQGAVEHNFKEKTQRINRVIKILDN**TFKQYYLIPKQPKISYNLGVGIMGSIKNTTISLDYNYYL

R. helvetica **EIALTMKENNILSGVIGTKVTVPLKDVLKFNNLGLIFQGAVEHNFKEKTQRINRVVKIFDT**EFKQNYVIPKQPKTSYNLGTGIIGSIKNTTIALDYIYYL

REIS **EIALTMKENNILSGIVGTKVKVPLKDALKFNNLGLTFQGAVEHNFKEKTQRINRVVKIFDN**TFKQDYLIPKQPKTSYNLGTGIIGSIKNTTISLDYNYYL

R. massiliae MTU5 **EIALTMKENNILSGIVGTKVTVPLKDALKFNNLGLTFQGAVEHNFKEKTQRINRVVKIFDN**TFKHNYLIPKQPKTSYNLGTGIIGSIKNTTISLDYNYYL

Candidatus R. amblyommii **EIALTMKENNILSGIVGTKVTVPLKDALKFNNLGLTFQGAVEHNFKEKTQRINRVVKIFDN**TFKHHYLIPKQPKTSYNLGTGIIGSIKNTTISLDYNYYL

R. rhipicephali **EIALTMKENNILSGIVGTKVTLPLKDALKFNNLGLTFQGAVEHNFKEKTQRINRVVKIFDD**TFKHDYLIPKQPKTSYNLGTGIIGSIKNTTISLDYNYYL

R. aeschlimannii **EIALTMKENNILSGIVGTKVTVPLKDALKFNNLGLTFQGAVEHNFKEKTQRINRVVKIFDD**TFKHDYLIPKQPKTSYNLGTGIIGSIKNTTILLDYNYYL

R. montanensis **EIALTMKENNILSGIVGTKVTVPLKDALKFNNLGLTFQGAVEHNFKEKTQRINRVVKIFDN**TFKHNYLIPKQPKTSYNLGTGIIGSIKNTTISLDYNYYL

R. slovaca 13-B **EIALTMKENNILSGIVGTKVTVPLKDALKFNNLGLTFQGAVEHNFKEKTQRINRVVKIFDN**TFKHNYLIPKQPKTSYNLGTGIIGSIKNTTISLDYNYYL

R. rickettsii Iowa **EIALTMKENNILSGIVGTKVTVPLKDALKFNNLGLTFQGAVEHNFKEKTQRINRVVKIFDN**TFKHNYLIPKQPKTSYNLGTGIIGSIKNTTISLDYNYYL

R. rickettsii Sheila Smith **EIALTMKENNILSGIVGTKVTVPLKDALKFNNLGLTFQGAVEHNFKEKTQRINRVVKIFDN**TFKHNYLIPKQPKTSYNLGTGIIGSIKNTTISLDYNYYL

R. japonica **EIALTMKENNILSGIVGTKVTVPLKDALKFNNLSLTFQGAVEHNFKEKTQRINRVVKIFDN**TFKHNYLIPKQPKTSYNLGTGIIGSIKNTTISLDYNYYL

R. honei **EIALTMKENNILSGIVGTKVTVPLKDALKFNNLGLTFQGAVEHNFKEKTQRINRIVKIFDN**TFKHNYLIPKQPKTSYNLGTGIIGSIKNTTISLDYNYYL

R. conorii str. Malish 7 **EIALTMKENNILSGIVGTKVTVPLKDALKFNNLGLIFQGAVEHNFKEKTQRINRVVKIFDN**TFKHNYLIPKQPKTSYNLGTGIIGSIKNTTISLDYNYYL

R. sibirica 246 **EIALTMKENNILSGIVGTKVTVPLKDALKFNNLGLTFQGAVEHNFKEKTQRINRVVKIFDN**TFKHNYLIPKQPKTSYNLGTGIIGSIKNTTISLDYNYYL

R. africae ESF-5 **EIALTMKENNILSGIVGTKVTVPLKDALKFNNLGLTFQGAVEHNFKEKTQRINRVVKIFDN**TFKHHYLIPKQPKTSYNLGTGIIGSIKNTTISLDYNYYL

R. parkeri **EIALTMKENNILSGIVGTKVTVPLKDALKFNNLGLTFQGAVEHNFKEKTQRINRVVKIFDN**TFKHNYLIPKQPKTSYNLGTGIIGSIKNTTISLDYNYYL

R. heilongjiangensis **-------------------------------------------------------------**---------------------------------------

Israeli tick typhus **EIALTMKENNILSGIVGTKVTVPLKDALKFNNLGLTFQGAVEHNFKEKTQRINRVVKIFDN**TFKHNYLIPKQPKTSYNLGTGIIGSIKNTTISLDYNYYL

Rickettsia sp. A-167 **EIALTMKENNILSGIVGTKVTVPLKDALKFNNLGLTFQGAVEHNFKEKTQRINRVVKIFDN**TFKHNYLIPKQPKTSYNLGTGIIGSIKNTTISLDYNYYL

**[2601-2621 ]**

**[ | | | ]**

R. bellii RML369-C NKRYHSHQGSIKLKVNL----

R. bellii OSU 85-389 NKRYHSHQGSIKLKVNL----

R. prowazekii Madrid E NKHYRSHQGSLKIKVNL----

**R. typhi Wilmington NKHYRSHQGSVKIKVNL----**

R. akari Hartford NKHYRSHQGSVKLKVNL----

R. australis NKHYRSHQGSVKLKVNL----

R. felis URRWXCal2 NKHYRSHQGSVKLKVNL----

R. canadensis McKiel NKHYRSHQGSVKLKVNL----

R. helvetica NKHYRSHQGSVKLKVNL----

REIS NKHYRSHQGSVKLKVNL----

R. massiliae MTU5 NKHYRSHQGSVKLKVNL----

Candidatus R. amblyommii NKHYRSHQGSVKLKVNL----

R. rhipicephali NKHYRSHQGSVKLKVNL----

R. aeschlimannii NKHYRSHQGSVKLKV------

R. montanensis NKHYRSHQGSVKLKVNL----

R. slovaca 13-B NKHYRSHQGSVKLKVNL----

R. rickettsii Iowa NKHYRSHQGSVKLKVNL----

R. rickettsii Sheila Smith NKHYRSHQGSVKLKVNL----

R. japonica NKHYRSHQGSVKLKVNL----

R. honei NKHYRSHQGSVKLKVNHER--

R. conorii str. Malish 7 NKHYRSHQGSVKLKVNL----

R. sibirica 246 NKHYRSHQGSVKLKVNL----

R. africae ESF-5 NKHYRSHQGSVKLKVNL----

R. parkeri NKHYRSHQGSVNA--------

R. heilongjiangensis ---------------------

Israeli tick typhus NKHYRSHQGSVKLKVNNE---

Rickettsia sp. A-167 NKHYRSHQGNVKLKVNMKGAA

**[1-100 Pro-rich tract ]**

**[ | | | | | | | | | | ]**

R. bellii RML369-C ----------MSLGYKLKSTLLKYSFVVAISINLLAINSGILLLTLNNKAEAAPPPPPPPPP-------PPPPPPPPPPPPPTPPPPP------------

R. bellii OSU 85-389 ----------MSLGYKLKSTLLKYSFVVAISINLLAINSGILLLTLNNKAEAAPPPPPPPP-------SPPPPSP--------PPPPSP-----------

R. prowazekii Madrid E ----------MTITGKLKSVLLTYSFIISINLLT-------TMSE--ASAPSGNPPPPPPP--------PPPPIEGLNKFKSLNSPPNHQLDNSAKVEQA

**R. typhi Wilmington ----------MTITGKLKSVLLTYSFIILINLLT-------AMSE--SLASSWNPPPPP----------PPPPIEGLNNFKSLNPPSKHSLGNSSKVEQS**

R. akari Hartford ----------MSITGKLKSVLLTYSFIISISINL--------FTT--GSEAAGNPPPPPPP--------PPLPPPGWTYQ------AASSNNTGSGGQTT

R. akari Hartford 2 ----------------MSTCLFTSSFLSTSTRAA-------SFKDLVSKTPTWQKHNAKQQQNIWKDFTPNEKIKKWQEANLIPSFTQAQDDLGIQYKET

R. australis MDLQNSHSKKYVLTFFMSICLLTSSFLSTSARAA-------SFKDLVSKTPTWQKHNSKQQQNIWKDFTLNEKIKKWQEANLIPSLTQAQDDLGIKYKET

R. felis URRWXCal2 MSLQNSHSKKYVLTFFMSTCLLTSSFLSTSARAA-------SFTQLANQIPTLSGLSEVQRKQKWNSYTLQEKQEAWRRAKLTPDFVQAMIDMQTGFTES

R. canadensis McKiel MNAQSFNSKKYALAFFMSTYLLAISFLSTSARAA-------SFTQLANKTPALSHLSEAQRKNQWNQYSLVTKQAAWKAAKLVPDLNQAILDVQNGFDPK

REIS ----------MILLIRLPDTL-------------------------------------------------------------------------------

R. massiliae MTU5 MNLQNSYSKKYVLTFFMSICLLTSSFLSTSARAA-------NFKDLVSKTPTWEKYNSTQQQNIWKNLTPNEKIKKWQEADLVPSFTQAQDDLGIKYKET

R. rhipicephali MNLQNSHSKKYVLTFFMSICLLTSSFLSTSARAA-------NFKDLVSKTPTWEKYNLKQQQNIWDNLTPNEKIKKWQEADLVPSFTQAQDDLGINYKET

R. aeschlimannii ----------------------------------------------------------------------------WQEAALVPSFTQAQDDLGIKYKET

R. montanensis ---QNSHSKKYVLTFFMSTCLLTSSFLSTSARAA-------SFKDLVSKNPAWEKHNEIQQQNIWKDLTPNEKIKKWQAADLVPSFTQAQDDLGIKYKET

R. slovaca 13-B -----EFTKSHSKNMFTFFCTCCDSSFSTSARAA-------SFKDLVSKTPAWEKHNSTQQQKIWKNLTLNEKIKKWQEAALVPSFTQAQNDLGIKYKET

R. peacockii Rustic ---------------------MTSSFLSTSARAA-------SFKDLVSKTPAWEKHNATQQQNIWNDLTQNEKIKKWQEAALVPSFTQAHNDLGIKYKET

R. rickettsii Iowa MNLQNSHSKKYVLTFFMSTCLLTSSFLSTSARAA-------SFKDLVSKTPAWAKHNSTQQQNIWKDLTPTEKIKKWQEAALVPSFTQAQNDLGIKYKET

R. rickettsii Sheila Smith ---------------------MTSSFLSTSARAA-------SFKDLVSKTPAWAKHNSTQQQNIWKDLTPTEKIKKWQEAALVPSFTQAQNDLGIKYKET

R. japonica --LQNSHSKKYVLTFFMSTCLLTSSFLSTSARAA-------SFKDLVSKIPAWEKHNSTQQQNIWKDLTPNEKIKKWQEADLVPSFTQAQDDLGIKYKET

R. honei -----------------------------------------------------------------------------AEAALVPSFTQAQHDLGIKYKET

R. conorii Malish 7 MNLQNSHSKKYVLTFFMSTCLLTSSFLSTSARAA-------SFKDLVSKTPAWEKHNSTQQQNIWKDLTPNEKIKKWQEAALVPSFTQAQNDLGIKYKET

R. sibirica 246 ----------------MSTCLLTSSFLSTSARAA-------SFKDLVSKTPAWEKHNSTQQQNIWKDLTPNEKIKKWQEAALVPSFTQAQNDLGIKYKET

R. africae ESF-5 MNLQNSHSKKYVLTFFMSTCLLTSSFLSTSARAA-------SFKDLVSKTPAWEKHNSTQQQNIWKDLTPNEKIKKWQEAALVPSFTQAQNDLGIKYKET

R. parkeri MNLQNSHSKKYVLTFFMSTCLLTSSFLSTSARAA-------SFKDLVSKTPAWEKHNSTQQQNIWKDLTPNEKIKKWQEAALVPSFTQAQNDLGIKYKET

**^^ ^ ^ ^^**

**[101-200 ]**

**[ | | | | | | | | | | ]**

R. bellii RML369-C -------------------------LPKTPPVDPKSA----------AKDINAELGPTFNPYDTLKKNRKNIKNKKDNSDLEAFLNLN------------

R. bellii OSU 85-389 -------------------------PPKTPPVDPKSA----------AKDINAELGPTFNPYDTLKKNRKNIKNKKGNSDLGAFLNLN------------

R. prowazekii Madrid E QHTN--KNYTTSNIKTQLSQSLEV-PTPTGEFGKKYSKLIN------PKTSKQNLKIEF-PNKIISLVRDELTTKYFAADIEKILTLF------------

**R. typhi Wilmington QHTN--KNCTTSNIQMQFSQSLEV-PTPTGEFGKKYSKLIN------PKTSKQNLKIEF-PKKIISLVRDELTLKYFTADIERILTLFSKKDDLILSEAK**

R. akari Hartford QPES--SKSTGSSKKSSSSTKIPEVHPPTTELGKRYAKLID------IHDRKQNLIIQF-PKVIAYLIRQELTLQNLDTSVHNIITLFNKREDSILDNAK

R. akari Hartford 2 DLSSFLDNTRHKARQARAEILLYIQRIKQQDFDTKKQEYIKQGIVPTYMEAATILGISYDPSKIYDNVEKDQNVRRAEKDKKALIDLY-----ISSIKRD

R. australis DLSSFLDKTRHKARQARAEILLYIERIKQQYFDTKKQEYIKQGIVPTDMEAATNLGISYDPSKIDNNVEKDQKVRRAEKDKQALIDLY-----ISSINRD

R. felis URRWXCal2 DLSSRNNKTRHKAREKKSELDLYIAGTK-QGFKEKVDGYISQGKIPTPEEAAQNLEIDYDAKKTDNKLEKNQNVRRVEKDKKALLDLY-----IHSITTS

R. canadensis McKiel LLSDSNTNIRHKAREKNADITLYIAQIAQPDYDKKFNEYINRGVIPTPEEVGENLGIVYDPRKTNSTIETDQKVRRAEKDKKAIIDRY-----IDLVKIA

REIS ------------KKRAGPKVIIGIYQIKVQIIQKHLRGFL-----------------------------------RIEKRNNHILLIL------------

R. massiliae MTU5 DLSSFVDNTRNKARHARAKILLYIERVKQQDFDTKKQEYINQGVVPTDIEAATNLGISYDPSKIDNNVENDQKVRRAAKDKKAVIELY-----ISSINRD

R. rhipicephali DLSSFVDKTRHKARQARAKILLYIERIKQQDFDTKKQEYINQGVVPTYIEAATNLGISYDPSKIDNNVENDQKVRRTEKDKKAVIELY-----ISLINRD

R. aeschlimannii ALSSFVDNTRHTARQARAKILLYIERVKQQDFDTKKQEYINYGVLPTDIEAATNLGISYDPSKIYNNVETDQKVRRAEKDKKAVIDLY-----ISSINRD

R. montanensis DLSSFLDNTRHKARQARAEILLYIERIKQQDFDTKKQEYINQGVVPTDIEAATNLGISYDPSKIDNNVENDQKVRRAEKDKKAVIGLY-----VSSINRG

R. slovaca 13-B DLSSFLDNTRHKARQARAEILLYIARVKQQDFDTKKQEYINQGVVPTDIEAETNLGISYDPSKIDNNVEHDQKVRRAEKDKKAVIELY-----VSSINRD

R. peacockii Rustic DLSSFLDNTRHKARQARAKILLYIERVKQQDFDTKKQEYINQGVVPTDIEAATNLGISYDPSKIDNNVEHDQKVRRAEKDKKAVIELY-----ISSINRD

R. rickettsii Iowa DLSSFLDNTRHKARQARAEILLYIERIKQQDFDTKKQAYINQGVVPTDIEAATNLGISYDPSKIDHNVEHDQKVRRAEKDKKAVIELY-----ISSINRD

R. rickettsii Sheila Smith DLSSFLDNTRHKARQARAEILLYIERIKQQDFDTKKQAYINQGVVPTDIEAATNLGISYDPSKIDHNVEHDQKVRRAEKDKKAVIELY-----ISSINRD

R. japonica DLSSFLYNTRHKARQARAEILLYIERVKQQDFDTKKQEYINQGVVPTDIEAATNLGISYDPNKIDNNVEHNQKVRRAEKDKKAIIELY-----VSSINRA

R. honei DLSSFLDNTRHKARRARAEILLYIERVKQQDFDTKKQEYINQGVVPTDIEAATNLGISYDPSKIDNNVEHDQKVRRAEKDKQAVIELY-----VSSINRG

R. conorii Malish 7 DLSSFLDNTRHKARQARAEILLYIERVKQQDFDTKKQAYINQGVVPTDIEAATNLGISYDPSKIDNNVEHDQKVRRAEKDKKAVIELY-----VSSINRG

R. sibirica 246 DLSSFLDNTRHKARQARAEILLYIERVKQQDFDTKKQEYINQGVVPTDIEAATNLGISYDPSKIDNNVEHDQKVRRAEKDKKAVIELY-----ISSINRD

R. africae ESF-5 DLSSFLDNNRHKARQARAEILLYIERVKQQDFDTKKQEYINQGVVPTDIEAATTLGISYDPSKIDNNVEHDQKVRRAEKDKKAVIELY-----ISSINRG

R. parkeri DLSSFLDNTRHKARQARAEILLYIERVKQQDFDTKKQEYINQGVVPTDIEAATNLGISYDPSKIDNKVEHDQKVRRAEKDKKAVIELY-----ISSINRG

**^ ^^ ^^^ ^ ^ ^ ^**

**[201-300 RRRRRRRRRRRRRRRRRRRRRRRRRRRRRRRRRRRRRRRRRRRRR]**

**[ | | | | | | | | | | ]**

R. bellii RML369-C ----------GIAQ----------------MSKLFSIVLTQHTDNDELRARFFKD**IIRNN**K--LSPTEKFNLANQIAANNGIYEKDDK------------

R. bellii OSU 85-389 ----------GIAQ----------------MSKLFSIVLTQHTDNDELRTRFFKD**IVRNN**K--LSPTEKFNLANQIAANNGIYEKDDK------------

R. prowazekii Madrid E -----SKKNDVI------------------LSEAKE--------------KMKKE**ILSKQ**GNILEQLKFINANTEILT----------------------

**R. typhi Wilmington EKYKEVIQDPYIAQFTNKNQNTGSFLENKLFEETREEAILRLLSLEQLDAQMKKEILSKQGDILEQLKFINANTEILGSYGQTASTDILKKIPKELPKIL**

R. akari Hartford KIYQEFINDPYIAQFTNKNKQTGDFLDNKLFEESHEEAILRLLSLEQLDAQRKQD**ILSQQ**GNILEQLKFIKENSEILGSYGKTESTNISKKLPEELPKIL

R. akari Hartford 2 IKYRHYVNNHIIPE----------------IKEVKTALNMDKDDAESFVLSIRTE**IMENA**KEQYIADRHIPTEKELKNRFGISRDDNRDAYIKSIRLKVM

R. australis IKYKHYIDNNIIPE----------------IKEVKTALNMDQDDAESFVVSIRTE**IMENA**KGQYIADSHIPTEKELKNRFGISRDDNRDGYIKSIRLKVM

R. felis URRWXCal2 VKEKEYITTGQVPE----------------LGELEKALNISKEEAKHRRTIIRDQ**VMANE**RPKLVRSGTVLTKKELHKRFGKDTTTDDTKYIDDITTEVM

R. canadensis McKiel LVEKDYTTTGMVPE----------------LEELTKKLNIDKAKAKLRRTAIRDQ**VMKNK**KAEIITIGKIPTQETLHKLFGKDRESNDTKYINDIRMHVM

REIS ---------------------------------------MNKDDAESFVVSIRTE**IMENA**KGQYIADSHIPTEKELKNRFGISRADNRDGYIKSIRLKVM

R. massiliae MTU5 IKYKHYVDNDIIPE----------------MKEVRTALNMNKDDAESFVASIRTE**IMENA**KGQYIADSHIPTEKELKNRFGISRDDNRDGYIKSIRLKVM

R. rhipicephali IKYKHYVDNDIIPE----------------MKEVRTALNMNKDDAESFVASIRTE**IMENA**KGQYIADSHIPTKKELKNRFGISRGDNRDDYIKSIRLKVM

R. aeschlimannii IKYKYYVDKDIIPK----------------MQEVMTTLNMNKDDAESFVASIRTE**IMENA**KEQYIANSHVPTKEELTNRFGTSRYDNKDGYIKSIRLKVM

R. montanensis IKYKHYVDNDIIPA----------------MKEVRTALNMNKDDAESFVASIRTE**IMENA**KRQYIADSHIPTEQEFKNRFGISRDDNRDGYIKSIRLKVM

R. slovaca 13-B IKYKHYVDNDIIPE----------------MQEVRTALNMNKDDAQSFVASIRTE**IMENA**KGQYIAGSHIPTEKEL------------------------

R. peacockii Rustic IKYKHYVDNDIIPE----------------LQEVRTALNMNKDDAQSFVASIRTE**IMENA**KGQYIADSHIPTEKELKKKFGISRDDNRDGYIKSIRLKVM

R. rickettsii Iowa IKYKHYVDNDIIPE----------------MQEVRTALNMNKDDAQSFVASIRTE**IMENA**KGQYIADSHIPTEKELKKKFGISRDDNRDGYIKSIRLKVM

R. rickettsii Sheila Smith IKYKHYVDNDIIPE----------------MQEVRTALNMNKDDAQSFVASIRTE**IMENA**KGQYIADSHIPTEKELKKKFGISRDDNRDGYIKSIRLKVM

R. japonica IKYKHYVDNDIIPE----------------MQEVRTALNMNKDDAESFVASIRTE**IMENA**KGQYIADSHIPTEKELKKRFGIFRDDNRDGYIKSIRLKVM

R. honei IKYKHYVDNDIIPE----------------MQEVRTALNMNKDDAQSFVASIRTE**IMENA**KGQYIADSHIPTEKELKKKFGISRDDNRDGYIKSIRLKVM

R. conorii Malish 7 IKYKHYVDNDIIPE----------------IQEVRTALNMNKDDAQSFVASIRTE**IMENA**KGQYIADSHIPTEKELKKKFGISRDDNRDGYIKSIRLKVM

R. sibirica 246 IKYKHYVDNDIIPE----------------MQEVRTALNMNKDEAQSFVASIRTE**IMENA**KGQYIADSHIPTEKELKKKFGISRDDNRDGYIKSIRLKVM

R. africae ESF-5 IKYKHYVDNDIIPE----------------IQEVRTALNMNKDDAQSFVASIRTE**IMENV**KGQYIADSHIPTEKELKKKFGISRDDNRDGYIKSIRLKVM

R. parkeri IKYKHYVDNDIIPE----------------MQEVRTALNMNKDDAQSFVASIRTE**IMENA**KGQYIADSHIPTEKELKKKFGISRDDNRDGYIKSIRLKVM

**GNYMN ^ ^^ ^**

**[301-400 RRRRRRRRRRRRRRRRRRRRR ]**

**[ | | | | | | | | | | ]**

R. bellii RML369-C ------------------------------**-**--------------------------------------------------------------GNIIIDN

R. bellii OSU 85-389 ------------------------------**-**--------------------------------------------------------------GNIIIDN

R. prowazekii Madrid E ------------------------------**-**------------------------------------EHSKAILKDKLKELSKQLDEISSNKLVGFILDE

**R. typhi Wilmington SKQGDILEQLKFINANTEILN----------------------------------------------EHSKAILKDKLKELSKQLDEISSNQLVGFILDE**

R. akari Hartford SQQGDILGQLRFIKENSEILGAYG--KTVS**T**DISKKLPKELPKILSQQGDILGQLKFINDNSEILLCGHSKAILKDKLKELSKQLDTISSNQLVGFILDE

R. akari Hartford 2 DKEKPQYIAANSIPTEKELEQKFGCDKGEA**T**NYIASIATQK---------MLNKKAYYIDNNIIPAVEELKQEFRIGKIKANSYIQQITDGINANQLLNN

R. australis DKEKPQYIAANSIPTEKELEQKFGANKGEA**T**NYIASIATQM---------MLSKKSYYIDNNIIPNADELMNEFKIGPVKATSYINQIRAGIEANQFLNN

R. felis URRWXCal2 YTKKQGYVNTDFLPKISEIMNEFKVDKGRA**N**---------------------------------------------------LYLNQIKAGIEAKLLADN

R. canadensis McKiel HNKKQHYVDTNHIPNTHEIMKEFNIDKGHA**K**---------------------------------------------------LYLKQIKAETEVKHQADN

REIS NKEKPQYIADNSIPTEKELKQKFGADKGEA**T**NYIALIATQM---------MLNKKAYYIDNNIIPIVKELKEEFKYRDNK-------------SKFLYTA

R. massiliae MTU5 DKEKPQYIADNSIPTEKELEKKFGADKDEA**T**NYIASIATQM---------MLGKKSYYIDNNIIPNADELMNEFKIGKIKANSYIDQIRAGIDANQLLNN

R. rhipicephali DKEKPQYIADNSIPTEKELEKKFGADKDEA**T**NYIASIATQM---------MLGKKSYYIDNNIIPNADELMNEFKIEDIKANSYIHQIRAGIDAKQLLNN

R. aeschlimannii NKEKPQYIADSSIPTEEELEKKFGANKDEA**T**NYIASIATQM---------MFDIKPYYIDN-ILPLTLKIMHKFKIGKIKHFLYRSN-KSRNKGKTVLNN

R. montanensis DKEKPQYIAGNSIPTEKELEQKFGADKGEA**T**NYIASIATQM---------MLGKKSYYIDNTIIPNADELMNEFKIGKIKANSYIDQIRAGMKANQFLNN

R. slovaca 13-B -------------------EQKFGTDKGEA**T**NYIASIATQM---------MLGKKSYYIDNNIIPNANELMNEFKIGPVKATSYINQIRAGIEANQFLNN

R. peacockii Rustic DKEKPQYIADNHIPTEKELEQKFGADKGEA**T**NYIAS------------------------------------------------INQIRAGIEANQFLNN

R. rickettsii Iowa ENAKGQYIADSHIPTEKELEQKFGADKGEA**T**NYIASIATQM---------MLGKKSYYIDNNIIPNTDELMNEFNIGPVKATSYINQIRAGIEAKQFLNN

R. rickettsii Sheila Smith ENAKGQYIADSHIPTEKELEQKFGADKGEA**T**NYIASIATQM---------MLGKKSYYIDNNIIPNTDELMNEFNIGPVKATSYINQIRAGIEAKQFLNN

R. japonica DKEKPQYIADSHIPTEKELEQQFGADKGEA**T**NYIASIATQM---------MLGKKSYYIDNNIIPNADELMNEFKIGIVKANSYIDQIRAGIAANQLLNN

R. honei ENAKGQYIADSHIPTEKELEQKFGADKVRH**K**LY-RTIATQM---------MLGKKSYYIDNNIIPNADELMNEFKIGPVKATVYINQIRAGIEANQLLNN

R. conorii Malish 7 DKEKPQYIADSHIPTEKELEQKFGADKGEA**T**NYIASIATQM---------MLDKKSYYIDNNIIPNADELMNEFKIGPVKATSYINQIRAGIEANQFLNN

R. sibirica 246 DKEKPQYIADSHIPTEKELEQKFGADKGEA**T**NYIASIATQM---------MLGKKSYYIDNNIIPNADELMNEFKIGPVKTASYINQIRAGIEANQFLNN

R. africae ESF-5 DKEKPQYIADSHIPTEKELEQKFGADKGEA**T**NYIASIATQM---------MLGKKSYYIDNNIIPNADELMNEFKIGPVKAMSYINQIRAGIEANQFLNN

R. parkeri DKEKPQYIADSHIPTEKELEQKFGVDKGEA**T**NYIASIATQM---------MLGKKSYYIDNNIIPNADELMNEFKIGPVKATSYINQIKAGIEANQFLNN

**^**

**[401-500 ]**

**[ | | | | | | | | | | ]**

R. bellii RML369-C TN-----------------------------LNNNIIGS--------------TSYISNFNPNYKYIISYSTP-------------TEILEKDLTSEII-

R. bellii OSU 85-389 TN-----------------------------LNNNIIGS--------------TSYISKFNPNYKYIISYSTP-------------TEILEKDLTSEII-

R. prowazekii Madrid E NK-----------------------------INTNFKNV-------PFSEKKVREQVNNLNNKILEKIFLKDDG-----TITEQDLTKILQKHKETVLIK

**R. typhi Wilmington NK-----------------------------INTNLKNV-------HFSEKKVRETVSNLNNKILEKIFLKDDG-----TITEQDLTKILQKHQETVLIK**

R. akari Hartford NK-----------------------------INTNLKNV-------PFSAKKVREQVNNLNNEILEKIFLHDDG-----TITEQDLTRILQKYKETDLIK

R. akari Hartford 2 NN-TTKPSTVGSTKKIETKSDNWYMSNQG--INTTETSS-------VVTTGRKEKQSYYFDPISTFKAHFNNKENNGNLTQPQHNINRIIQQEENIEEFK

R. australis ND-TTKPSTGGSKKKSETKSDNWYMSNQG--ANTTGTSS-------GVPTGRKEKQSYFFDPISTFKAHFNNKESKGNLTQSQHNINRIIQQEENIGAFE

R. felis URRWXCal2 NQTTTKPFTKHSRTT----------------TNTAGISSGV-----PFDTGRTKPETKSFDFKRSMYSLLNRKQED-QLSKTEQHLKQQIKLEENKEEFK

R. canadensis McKiel KQNTTKPFTKHSRTTTD--------------TEEKFSQF-------PFDTLKKEKQPYSFDPRSTLERLLKGKGNN-NFTQSQQNIYKIIQQEANIEEFK

REIS NN------------------------------------------------NRNRCKPQMYNKIS------------------------------------

R. massiliae MTU5 NDTTTKQSAGHSQKKSGSKHN-WYMSNQG--THNTETSSGVSRTSSGISTGRKEKQPYFFDPISTFKTHFNHKKNKGNLTQSQHNINRIIQQEENIEAFN

R. rhipicephali NDTTTKQSAGHSQKKSGSKHN-WYMSNQG--THNTETSSGVSRTSSGIFTGRKEKQPYFFDPISTFKTHFNHKKNKGNLTQSQHNINRIIQQEENIEEFK

R. aeschlimannii ND-TTKQPAKFSQKKSVSKDDWYTWSNQGELNKDKGESS-------IISIGRKEKKPYFFNPISTFRTYLNNKENNDQLTLSQQNIKRIIQQEEXIKDFK

R. montanensis ND-TTKPSTGRSQKRAEAKTTIGICQIKVQIIQEHLQ---------GFPPGRKKKQPYFFDPISTFKTHFNNKESKGNLTQSQRNINRIIQQEENIEEFK

R. slovaca 13-B ND-TTKPSTGRSQKKSGSKNDHWYMSNQS--INNTGTSS-------RIFTGREKKQRYFFGPISTFKTHFNTKASKGNLTQSQHNINRIIQQEENIEEFK

R. peacockii Rustic ND-TTKPSTGRSQKKSGSKNDHWYMSNQS--INDTRTSS-------RIFTGRAKKQRYFFDPISTFKTHFNTKANKGNLTQSQHNINRIIQQEENIEEFK

R. rickettsii Iowa ND-TTKPSTGHSQKKSGSKNDHWYMSNQS--INDTGTSS-------RIFTGREKKQRYFFDPISTFKTHFNTKASKGNLTQSQHTIKRIIQQEENIAEFK

R. rickettsii Sheila Smith ND-TTKPSTGHSQKKSGSKNDHWYMSNQS--INDTGTSS-------RIFTGREKKQRYFFDPISTFKTHFNTKASKGNLTQSQHTIKRIIQQEENIAEFK

R. japonica ND-TTNPSAGRSQKKSGSQNDHKYRPNQD--INNTGTSS-------GIFTGRKKKQQYFFDPISTFKKYFNTKASKGNLTQSQHNLNRIIQQEENIEDFK

R. honei ND-TTKPSTGRSQKKSGNKNDHWYMSNQG--INNTGTSS-------RIFTGREKKQRYFFDPISTFKTYFNTKASKGNLTQSQHNINRIIQQEENIEEFK

R. conorii Malish 7 ND-TTKPSTGRSQKKSGSKNDHWYMSNQS--INNTGTSA-------RIVTGREKKQRYFFDPISTFKTYFNTKASKGNLTQSQHNINRIIQQEENIEEFK

R. sibirica 246 ND-TTKPSTGRSQKKSGSKNDHWYMSNQS--IHNTGTSS-------RIFTGREKKQRYFFDPISTFKTHFNTKANKGNLTQSQHSINRIIQQEENIEEFK

R. africae ESF-5 ND-TTKPSTGRSQKKSGSKNDHWYMSNQ---SHNTGTSS-------RIFTGRAKKQRYFFDPISTFKTYFNTKANKGNLTQSQHNINRIIQQEENIEEFK

R. parkeri ND-TTKPSTGRSQQKSGSKNNPWYMSNQS--IHNTETSS-------QISTGRDKKQRYFFDSISTFKTQFNAKANKGNLTLSQQNINKLIEQEENIEQFK

**[501-600 ]**

**[ | | | | | | | | | | ]**

R. bellii RML369-C -----------------DITKGFEKDSKGNSYRKFDETKLKQI--------------------------------EQERVY--------------GLIKS

R. bellii OSU 85-389 -----------------DITKGFEKDSKGNSYRKFDETKLKQI--------------------------------EQERVY--------------GLIKS

R. prowazekii Madrid E NLTK-----AIVY-----IDGNKNNETV--------NKTLEKCLEETTPEQQGMILDVLTNNTRIRKALITKIEREQRQEHNQKLNK--------NIAGD

**R. typhi Wilmington NLTK-----AIVY-----IDGNKNNATV--------NKTLEKCLEQTTPEQQELILDVLTHNTRIRTVLITKIEREQRQNHNKKLNK--------NIAGD**

R. akari Hartford NLTK-----AIIY-----IDGNESNETV--------SKTLAKGLENTTPEQRGLIVDVLTHNTRIRKALITKIDRERRQERNQKLNK--------NTEGD

R. akari Hartford 2 NLIKTDPIAALNL----TVDSSYKKEAVTSILSDFNDDTIQRVLFSDDMGQLDFKTNIDVKNRPILKALLENSSSEEKTKFAERIQDYATR----NISNS

R. australis NLIKTDPIAALNL----KVDSSYKKEAVTTILSDFNDDTIQRVLFSNDRGQLDFKTNIDVTNRPILKELLENSSSEEKTKFAERIKDYATR----NISNS

R. felis URRWXCal2 EILTKNPIDALLFAEQSNLGNSFKQEAISNI--DLSKD-ISRILFTVD----------DKGNRTILNTIL-TTTPEHKDELIKQAQHHAIQTLPTSISDK

R. canadensis McKiel HLIKTEPIAALNL----EVDSSYKKEAVNAILSDFNNDTIQKILFSDDKGKLNFNTNIDVEHKPILKELFKNSSEEQRKKIIENISKYAKE----NIIPL

REIS ---------ALAY---------------------------------------------------------------------------------------

R. massiliae MTU5 NLIKTNPIAALNL----EVDSSYTQEAVTTILSDFNDDTIQRVLFSNDTGQLDFNTNIDVKNRPILKKLLENSSSEEKTKFTERIQDYATR----NISNS

R. rhipicephali NLIKTNPIAALNL----EVDSSYKQEAVTTILSDFNDDTIQRVLFSNNTGQLDFNTNIDVKNRPILKKL-ENSSSEEKTKFTERIQDYATR----NISNS

R. aeschlimannii DLIKTDPIAALNL----EVDSSYKQEAVKTILSDFNDDTIQRVLFSDDTERLDFNTNIDVKNRPIFKELLENSSSEEKTKFVERIQDYAIR----NISNS

R. montanensis NLIKTDPIAALNL----EVGSSYKQEAVTTILSDFNDDTIQRVLFSNDKEQLDFNTNIDVKNRPILKELLENSSSEEKTKFAERIQDYATR----NIFNS

R. slovaca 13-B NLIKTDPIAALTL----QVDSSYKQKAVTTILSDFNDDTIQRVLFSNDKGPLDFNTNIDVKNRPILQELLENSSSEEKTKFAERIQDYATR----NISNS

R. peacockii Rustic NLIKTDPIAALTL----QVDSSYKQEAVTTILSDFNDDTIQRVLFSNDKGQLDFKTNIDVKNRPILQELLENSSSEEKTKFVERIQDYATR----NISNS

R. rickettsii Iowa NLIKTDPIAALTL----QVGSSYKQEAVTTILSDFNDNTIQRVLFSNDKGQLDFKTNIDVKNRPILQELLENSSSEEKTKFAERIQDYATR----NISHS

R. rickettsii Sheila Smith NLIKTDPIAALTL----QVGSSYKQEAVTTILSDFNDNTIQRVLFSNDKGQLDFKTNIDVKNRPILQELLENSSSEEKTKFAERIQDYATR----NISHS

R. japonica NLIKTDPIAALTL----QVDSIYKQEAVTTILSDFNDDTIQRVLFSNDKGQLDFNTNIDVKNRPILKELLENSSSEEKTKFAERIQDYATR----NISNS

R. honei NLIKTDPIAALTL----QVDSSYKQEAVTTILSDFNDDTIQRVLFSNDKGQLDFSTNIDVKNRPILQELLENSYSEEKTKFAERIQDYATR----NISNS

R. conorii Malish 7 NLIKTDPIAALTL----QVDSSYKQEAVTTILSDFNDDTIQRVLFSNDKGKLDFNTNIDVKNRPILQELLENSSSEEKTKFAERIQDYATR----NISNS

R. sibirica 246 NLIKTDPIIALTL----QVDSSYKQEAVTTILSDFNDDTIQRVLFSNDKGQLDFNTNIDVKNRPILQELLENSSSEEKTKFAERIQDYATR----NISNS

R. africae ESF-5 NLIKTDPIAALTL----QVDSSYKQEAVTTILSDFNDDTIQRVLFSNDKGQLDFNTNIDVKNRPILQELLENSSSEEKTKFAERIQDYATR----NISNS

R. parkeri DLIKTNPIAALLL----QVDSSYKNQAVKIILKDRNDNTIQRLLFTNDTGQLDFNTNIKVKNRPILQTFLNNSTSKDKTKFAEIIQDYATR----NISNS

**[601-700 ]**

**[ | | | | | | | | | | ]**

R. bellii RML369-C KVE---------SLIELAYGANIKYVDYYNSN-------------INDNSY-------------------------------------------------

R. bellii OSU 85-389 KVE---------SLIELAYGANIKYVDYYNSN-------------INDNSY-------------------------------------------------

R. prowazekii Madrid E TFVDALKK----ALVH-RTSNPETIQKSLERRKKETPKNLNVWDRISQNIPNLNNQNDNQNGQDENNKEWEESNQNADYLNNTNIYRITKAKQDLEKAVK

**R. typhi Wilmington SFVDALKK----ALVH-RTSNSETILKVVEQRKQETPKNLNVWDRISQNIPNLNNQNVQ----DENNKEWDESNKNADDLNNTNIYMIT--KHDLERAVN**

R. akari Hartford PFVDALKK----ALVQ-RKSDPETIQKELERRKHETPRDLNAWDRVRQNMHNPNNQNDNPNTHDENNKGWKEDNQNAQDANNNDVDRITKAKNELEQAVN

R. akari Hartford 2 QFEEKARL----DLIKLAASKDRSLVEKFLALQLELKNKMQSH--IVKSEYILTPKIVAEINIELKNQGLIIDSLTKD-------NMIKLAKEVNKQALN

R. australis QFEEKARL----DLIKLAASKDKSLVEKFLALQLELKNKMQSY--IVKSEYILTPKIVAAINIELKNQGLIIDSLTKD-------DMIKLAKSVNKQALN

R. felis URRWXCal2 DVSDKKKL----TLATLAATEDKKVLEEAL----------DNW--LSTNGYKRKPEV------------------------------------------E

R. canadensis McKiel VFKGEVRHDKLSDLLKLAAITDKSIVKKVLMLQLELKNNVHSY---TAQGNGISPEGIKVINAILRKYHVTEKDLSSS-------EMIKLANSFHSQGFE

REIS ----------------------------------------------------------------------------------------------------

R. massiliae MTU5 QFKEKARL----DLIKLAASKDKSSVENFLALQLELKNKMQPY--IVKSTYILTPEIVKAINIELKNKGLIIDSLTKD-------DMIKLVKALNKQTLN

R. rhipicephali QFEEKARL----DLIKLAASKDKSSVENFLALQLELKNKMQPH--IVKSTYILTPEIVKEINIELKNKGLIIDSLTKD-------DMIKLAKALNKQTLN

R. aeschlimannii KFEEKARL----DLVKLAASKDKSSVENFLALQLELKNKMHQY--IAKSVYILTPEIVNEINIELKNKGLITDSLTQD-------YIIKLTKAVNKQTLN

R. montanensis QFEEKARL----DLIKLAASKDKSSVENFLALQLELKNRMQPY--IVKSVYILTPEIVKEINIELKNKGLIIDSLTKD-------DMIKLAKAVNKQTLN

R. slovaca 13-B QFKEKARL----DLIKLAASKDKSSVENFLTLQLELKNRMQPY--IVNSVYILTPEIVKEINIEPKKKGLIRDSLTKD-------YMIKLAKEVNNHTLN

R. peacockii Rustic QFEEKARL----DLIKLAASKDKSSVENFLTLQLELKNRIQPH--IANSVYVLTLEMVKEINIELKNKGLIKDSLTKD-------YMIKLAKEINNHTLN

R. rickettsii Iowa QFEEKARL----DLIKLAASKDKRSVENFLTLQLELKNRMQPY--IVNSAYILTPEIVKEINIELKNKGLIRDSLTKD-------YMIKLAKEVNNHTLN

R. rickettsii Sheila Smith QFEEKARL----DLIKLAASKDKRSVENFLTLQLELKNRMQPY--IVNSAYILTPEIVKEINIELKNKGLIRDSLTKD-------YMIKLAKEVNNHTLN

R. japonica QFEEKARL----DLIKLAASKDKSSVENFLTLQLELKNRMQPY--IVKSVYILTPEIVKEINIELKNKGLIRDSLTKD-------DMIKLAKEVNNYTLN

R. honei KFEEKARL----DLIKLAASKDKSSVENFLTLQLELKNRMQPY--IVNSVYILTPEIVEEINIELKNKGLIRDSLTKD-------YMIKLAKEVNNHTLN

R. conorii Malish 7 QFEEKARL----DLIKLAASKDKSSVENFLTLQLELKNRMQPY--VVNSVYILTPEIVKEINIELKNKGLIRDSLTKD-------YMIKLAKEVNNHTLN

R. sibirica 246 QFEEKARL----DLIKLAASKDKSSVENFLTLQLELKNIMQPY--IVNSVYILTPEIVKEINIELKNKGLIRDSLTKD-------YMIKLAKEVSNHTLN

R. africae ESF-5 QFEEKARL----DLIKLAASKDKSSVENFLTLQLELKNRMQPY--IVNSVYILTPEIVKEINIELKNKGLIRDSLTKD-------YMIKLAKEVNNHTLN

R. parkeri QFEEKARL----DLIKIAASKDKSSVENFLTLQLELKNRMQPY--IVNSVYILTPEIVKEINIELKNKGLIRDSLTKD-------YMIKLAKEVNNHTLN

**[701-800 ]**

**[ | | | | | | | | | | ]**

R. bellii RML369-C ---------EKYFTEYEKEVITKALGYKR-AYEEKTGL-------------------------------------------------------------Y

R. bellii OSU 85-389 ---------EKYFTEYEKEVITKALGYKR-AYEEKTGL-------------------------------------------------------------Y

R. prowazekii Madrid E ETINKFSAMSTLIKDNTIKNTMAYQKYLK-GAEDQLALAKEKGKEL--IENSVQTFKIIPKKY--------------------------QDDMNENWQNY

**R. typhi Wilmington ETITKFSAMSTLLKDK--KNAGAYQRYLK-EAEDQLALAQEKGKEL--IKNSAQTFKIIPKKY--------------------------QDDINENWQNY**

R. akari Hartford AATRKFSEMSSLLKDNTIKNATACQGYLK-GAEDQLALAKEKGQEL--IENSVQAFKIIPKKS--------------------------QDNMDDNWQNY

R. akari Hartford 2 SAIKVILSDNNTLSNETNKILGLAVGNNA-NNLAQTQSGMPNPPPL-PLSGGIPNPPPLPLSGGIPNPPPLPLSGGIPNPPPLPLNGSMPPPPHLNSQGF

R. australis SVIKVILSDNNALTNETNKILGLAVGNNA-NNLEQTQSGMPNPPPL-PLNGGIPNPPPLPLNGSMQ-----------------------PPPPPLNSQGF

R. felis URRWXCal2 SLISILLSDETTLKAGIDKIFELPVENNVNNNSNKGQNGTPILPPTPPLNGSMPPSPPPPLLNGT------------------------PTSTAFNN---

R. canadensis McKiel DSIKIMSFSEQELNTQIYNLFGLSIQNKT--------HGNGNMPPL------LPPPP--------------------------------PPLPPLFGNPY

REIS ------LALYLILSKKLDKSVSLYFI---------------------------------------------------------------PRSPCAMTSSF

R. massiliae MTU5 SAIKVILSDSNILSNETNKILGLAVGNNA-NNLEQTQSGIPNPPPL-PLNGGIPNPPPLPLNGSMP-----------------------PPPPPLNSQDF

R. rhipicephali SAIKVILSDSNILSNETNKILGLAVGNNA-NNLEQTQSGIPNPPPL-PLNGGIPNPPPLPLNGGIPNPPPLPLNGSMP-----------PPPPPLNSQDF

R. aeschlimannii SAIKVILSDSSILNNEINKILGLAVGNNA-NNLEQAQSGIPNPPPL-PLNMSIPNPPPLPLNGSMP-----------------------PPPPPLNSQDF

R. montanensis SVIKVILSDSNMLSNETNKILGLAAGNNA-NNLEQTQSGIPNPPPL-PLNGGIPNPPPLPLNGSMP-----------------------PPPPPLNSQGF

R. slovaca 13-B SVIKVILSDSNILSNETNKILGLAVGNNA-NNLEQTQSGIPNPPPL-PLNGGIPNPPPLPLNG--------------------------SIPPPLHSQGF

R. peacockii Rustic SVIKVILSDSNILSNETNKILGLAVGNNA-NNLEQTQSGIPNPPPL-PLNGDIPNPPPLPLNGSI-------------------------PSPPLHSQGF

R. rickettsii Iowa SVIKVILSDSNILSNETNKILGLAVGNNA-NNLEQTQSGIPNPPPL-PLNGDIPNPPPLPLNGDIPNPPPLPLNGDIPNPPPLPLNGSMPPPPPLHSQGF

R. rickettsii Sheila Smith SVIKVILSDSNILSNETNKILGLAVGNNA-NNLEQTQSGIPNPPPL-PLNGDIPNPPPLPLNGSM------------------------PPPPPLHSQGF

R. japonica SVIKVILSDSTILSNETNKILGLAVGNNA-NNLEQTQSGIPNPPPL-PLNGSIPNPPPLPLNGSIPNPPPLPLNGSMP-----------PLPPPFNSQGF

R. honei SVIKVILSDSNILSNETNKILGLAVGNNA-NNLEQTQSGIPNPPHSNRSLGVFQSASSTAQRSM-------------------------PPPPPLILHRL

R. conorii Malish 7 SVIKVILSDSKILSNETNKILGLAVSNNA-NNLEQTQSGIPNPPPL-PLNGGIPNPPPLPLNGSM-------------------------PPPPLHSQGF

R. sibirica 246 SVIKVILSDSNILSNETNKILGLAVGNNA-NNLEQTQSGIPNPPPL-PLNGGIPNPPPLPLNGSM------------------------PPPPPLHSQGF

R. africae ESF-5 SVIKVILSDSNILSNETNKILGLAVGNNA-NNLEQTQSGIPNPPPL-PLNGGIPNPPPLPLNGNM------------------------PPPPPLHSQGF

R. parkeri SVIKVILSDSNILSNEANKILGLAVGNNA-NNLEQTQSDIPNPPPL-PLNGGIPNPPPLPLNGSI-------------------------PPPPLHSQGF

**[801-900 ]**

**[ | | | | | | | | | | ]**

R. bellii RML369-C KSEVDKLDRSILSEE-------------EYIKKCEEILEKIGESSWDTYQVSKPGSSSKISYEEYE-----------------KLVTNEAKKRAVIARFL

R. bellii OSU 85-389 KSEVDKLDRSILSEE-------------EYIKKCEEILEKIGESSWDTYQVSKPGSSSKISYEEYE-----------------KLVTNEAKKRAVIARFL

R. prowazekii Madrid E LSPEEIIELTALNEH------------TNTLTSNKNKSGYFTSTA-EALQCKTKQQEYYTLLSKLKKIGIEKQQKKLVKDYVDEMITNAKQAVKKIERTL

**R. typhi Wilmington LSPAEMIELTALNEH------------TNTLKSNKNKSGHFRSSE-EALQYKAKQHEYYTLLAELKKIGIAKQQEKLVKDYVDEMITNAKQAVEKFERTS**

R. akari Hartford LSPKEIIALNALSDE------------TNTLKSKKNKAGKFTSTA-DASQFKAKQQKYHTLLSELQKIGIAKQQEKLVKDYVDEMLTNAKKAVGKIETTL

R. akari Hartford 2 ISNSNNLDLNKLQAEYSHIHSLYTQFILNTTVQPKVLPQPTASSA-TSTERSEPETAYAKLYVEYR----TETGGKKAYDLQDQLIKRQADLTNVIRQIL

R. australis ISNSNNFDLNKLQAEYSHIHSLYTQFTRNTTVQPKVLLQPTASSA-TSTERSDPETIYAKLYAEYR----TETGGKKADDLQDQLITRQADLTNVIRQIL

R. felis URRWXCal2 SNPNHKFDLKNFEATYPRLYKSYNEFIQNTTSASQSQA--------TTTSNNIPDT---------K----AKMG--ESLELLKQKVAKQNEVIGLIHNEV

R. canadensis McKiel --------LDKLKST-PKLYDLYNQFIQNNKSKNKKTTENITKS--NTPKRSEPKTEYAKLYKQYR----AETGGKKADDLQDQLIKKQSDITNVIRQIL

REIS LSTS------------------------------------------------------------------------------------------------

R. massiliae MTU5 SSNSNNFDLNKLQAEYPHIHSLYIQFTRNTTVQPKVPLQPTTASA-TSTDRSTPETAYAKLYAAYR----TETGGKKADDLQDQLIKRQADLTNVIRQIL

R. rhipicephali SSNSNNFDLNKLQAEYPHIHSLYIQFTRNTTVQPKVPLQPTTPSATTSTDRSTPETAYAKLYAAYR----TETGGKKAYDLQDQLIKRQADLTNVIRQIL

R. aeschlimannii SSNSNDFDLNKLQAEYPKIYTSYIQFTRNTTVQPKALLQLTTSSA----HRKMPKTAYAKLYAAYR----TETGGKKADDLQDQLIKRQADLTNVIRQIL

R. montanensis SSNSNNFDLNKLQAEYPHIHSLYIQFTRNT-VQPKVPSQPTTSSA-TSTDRSTPETAYAKLYTEYR----TETGGKKADDLQDQLIKRQADLDKCLRQIL

R. slovaca 13-B SSNSKHFDLNQLQAEYPHIHSLYIQFTHNTTVQSKAPLQPTASSA-TSTGRSTPETAYAKLYAEYR----TETGGTKANDLQDQLIKRQADLTNVIRQIL

R. peacockii Rustic SSNSKHFDLNQLQAEYPHIHSLYIQFTHNTTVQSKAPLQPTASSA-TSTGRSTPKTAYAKLYAEYR----TETGGTKANDLQDQLIKRQADLTNVIRQIL

R. rickettsii Iowa SSNSKHFDLNQLQTEYPHIHSLYIQFTHNTTVQSKAPLQPTASSA-TSTGRSTPETAYAKLYAEYR----TETGGTKANDLQDQLIKRQADLTNVIRQIL

R. rickettsii Sheila Smith SSNSKHFDLNQLQTEYPHIHSLYIQFTHNTTVQSKAPLQPTASSA-TSTGRSTPETAYAKLYAEYR----TETGGTKANDLQDQLIKRQADLTNVIRQIL

R. japonica SSNSNHFDLNQLQAEYPHIHSLYIQFTSNTTVQSKAPLQPTASSA-TSTDRSTPETAYAKLYAEYR----TETGGTKANDLQDQLIKRQADLTNVIRQIL

R. honei YA-SKHFDLNQLQAEYPHIHSLYIQFTHNTTVQSKAPLQPTASSA-TSTGRSTPETAYAKLYAEYR----TETGGTKANDLQDQLIKRQADLTNVIRQIL

R. conorii Malish 7 SSNSKHFDLNQLQTEYPHIHSLYVQFTHNTTVQSKAPLQPTASSA-TSTGRSTPETAYAKLYAEYR----TETGGTKANDLQDQLIKRQADLTNVIRQIL

R. sibirica 246 SSNSKHFDLNQLQTEYPHIHSLYIQFTHNTTVQSKAPLQPTASSA-TSTVRSTPETAYAKLYAEYR----TETGGTKANDLQDQLIKRQADLTNVIRQIL

R. africae ESF-5 SSNSKHFDLNQLQTEYPHIHSLYIQFTHNTTVQSKAPLQPTASSA-TSTVRSTPETAYAKLYAEYR----TETGGTKANDLQDQLIKRQADLTNVIRQIL

R. parkeri ISNSQHFDLNQLQTEYPHIHSLYIQFTHNTTVQSKAPLQPTASST-TSTVRSTPETAYAKLYAEYR----TETGGTKANDLQDQLIKRQADLTNVIRQIL

**[901-1000 ]**

**[ | | | | | | | | | | ]**

R. bellii RML369-C ME------------------------------------------DNNKINGKRLKIVKEDGSEEYIDNTE----------------------FNLIRAEN

R. bellii OSU 85-389 ME------------------------------------------DNNKINGKRLKIVKEDGSEEYIDNTE----------------------FNLIRAEN

R. prowazekii Madrid E -EKVNQKKENKYEFSESSALISKEILDAQARLENAKQKIEFIKLKQIISDKRQVNSSDEDSDDDSKKKCNQTKSRTMKIQMMILKKNAIKLKVELENAQ-

**R. typhi Wilmington LEHINQKKENKQ--------ISKEILDAQERLENAKQKIEFIKFKYIISNKRQVNSSDEDSDDDADKNA-------------------IKQKTELENAQ-**

R. akari Hartford -EYLNKKKENKNDNAESSPLISKEIFEAREQLKKAEQKLERIKFKNTIAKQRKADSSDEDSDDEWDDDYDK-------KATKQKEELANKQKQELEDAQ-

R. akari Hartford 2 TTSYANQGADEKTLVNVFSISTPEIEAKAKDVFNKLVQDPYI--QYITVNGKKATTSEDIIKNLFNEDTDDAVKRILLSSCKISEELKKPIKHELNQLKL

R. australis TESYANQGADEKTLVNLFSISTPEIAEKAKEVFNKLVQDPYI--QDITVNGKKATTSEDIIQNLFNEDTDDAVKRILLSSCKISEELKKPIKHELNQLKL

R. felis URRWXCal2 TKLY---NFSPKTFVNLFNTENEEIIKKIEQ----IAKREDI--QKILQDNDIKITS-TFVSKIFNESLEQTKQRLRSSNIINAKQYKR-IEQYANKQEC

R. canadensis McKiel TASYAEQGINTEDLITLFGNPNPEVTEKAKEVFVILVQDPYI--QDITVNGKKTITSEAILQNLFNEDADDAVERILLSSCKISEALKKPIEHELNKSKL

REIS -----------------------------------------------------------------------------------------------ARAAS

R. massiliae MTU5 TESYANQGADAKTLINLFSISTPEIAEKAKEAFNTLVQDPYI--QDITVNGKKTTTSEEIIKNLFNEDTDDAVKRILLSSCKISEELKRPIKLEFNKSEL

R. rhipicephali TESYANQGADAKTLVNLFSISTPEIAEKAKEAFNTLVQDPYI--QDITVNGKKTTTSEEIIKNLFNEDTDDAVKRILLSSCKISGELKRPIKLEFNKSEL

R. aeschlimannii TESYANQGADTKTLVNLFSISTPEIAEKAKEAFNTLIQDPYI--QDIAVNGKKTITSEDIIKNLFNEDTDDAVKRILLSSCKMPEELKIPIQLELNKSEL

R. montanensis TESYANQGADEKTLVNLFSISTPEIAEKAKEAFNTLVQDPYI--QDITVNGKKTITSEEIIKNLFNEDNDDAVKRILLSSCKISEELKKPIKLEFNKSEL

R. slovaca 13-B TESYANQGADAKTLVNLFSISTPEIAEKAKEAFNTLAQDQYI--KDITVNGKKTITSEEIIKNLFNEDTDDAVKRILLSSCKISEELKRPIKLEFNKSEL

R. peacockii Rustic TESYANQGADEKTLVNLFSISTPEIAEKAKEAFNILAQDQYI--KDITVNGKKTITSEEIIKNLFNEDTDDAVKRILLSSCKISEELKRPIKLEFNKSEL

R. rickettsii Iowa TESYANQGADEKTLVNLFSISTPEIAEKAKEAFNTLAQDQYI--KDITVNGKKTITSEEIIKNLFNEDTDDAVKRILLSSCKISEELKRPIKLEFNKSAL

R. rickettsii Sheila Smith TESYANQGADEKTLVNLFSISTPEIAEKAKEAFNTLAQDQYI--KDITVNGKKTITSEEIIKNLFNEDTDDAVKRILLSSCKISEELKRPIKLEFNKSAL

R. japonica TESYANQGADEKTLVNLFSISTPEIAEKAKEAFNTLVQDQYI--KDITVNGKKTITSEEIIKNLFNEDTDDAEKRFLLSSCKISEELKRPIKLEFNKSEL

R. honei TESYANQGADEKTLVNLFSISTPEIAEKAKEAFNTLVQDQYI--KDITVNGKKTITSEEIIKNLFNEDTDDAVKRILLSSCKISEELKRPIKLEFNKSEL

R. conorii Malish 7 TESYANQGADEKTLLNLFSISTPEIAEKAKEAFNTLAQDQYI--KDITVNGKKTITSEEIIKNLFNEDTDDAIKRILLSSCKISEELKRPIKLEFNKSEL

R. sibirica 246 TESYANQGADEKTLLNLFSISTPEIAEKAKEAFNTLAQDQYI--KDITVNGKKTITSEEIIKNLFNEDTDDAVKRILLSSCKISEELKRPIKLQFNQSEL

R. africae ESF-5 TESYANQGADEKTLLNLFSISTPEITEKAKEAFNTLAQDQYI--KDITVNGKKTITSAEIINNLFNEDTDDAVKRILLSSCKISEELKRPIKLQFKQSEL

R. parkeri TESYANQGADEKTLLNLFSISTPEIAEKAKEAFNTLAQDQYI--KDITVNGKKTITSEEIIKNLFNEDTDDAVKRILLSSCKISEELKRPIKLKFNQSEL

**[1001-1100 RRRRRRRRRRRRR]**

**[ | | | | | | | | | | ]**

R. bellii RML369-C NWDLTPANIPLSKVMKDTNN--------------------------------LSENEKMKKLL---------------------ARIEFLENNTNNLDII

R. bellii OSU 85-389 NWDLTPANIPLSKVMKDTNN--------------------------------LSENEKMKKLL---------------------ARIEFLENNTNNLDII

R. prowazekii Madrid E -KDINQAKKNLENAEAKNEA--------------------------------------------------------------------------------

**R. typhi Wilmington -KDINQAKKNLEDAKAKYAALQ-----------------------------------------------------------------ITLNYSPNGMDSK**

R. akari Hartford -EEIKQAKKNLENAEVRDAERKQQEAAAKRQEELKRKQDEVNHKIVEDEVERKAENAQAERLEDMDAMFKNAKDEAEHKEKANRQRQEELKRKQDEVNRK

R. akari Hartford 2 MREFESKTTLFEQLEFAYANAKN-----------------LDQDIFGNKVEELINNPKTLTTA---------------------QQATFLITEDTNLRKT

R. australis IRELESKPTLFEQLEFAYANAKN-----------------FDQDIFGNRVEELINNPKILTIL---------------------QQATFLITEDTNLRKT

R. felis URRWXCal2 VTEFLRITNPLEQLKFANKYINI-----------------LGQSTFNGKLNELIENPNKLTFS---------------------QKINFVLQGYQELTRE

R. canadensis McKiel MRELESKQNPFEQLKFAYEKAAI-----------------LGQ-TFDTKLAELIKNPAILTTQ---------------------EQINFLTQENNKLKKV

REIS FKDLVSKTPAWE----------------------------------------------------------------------------------------

R. massiliae MTU5 IRELQSKQNPFEQLEFAYTNAQY-----------------FDQDIFGNRVEELINNPHILTIA---------------------QQATFLITEDTHLRKT

R. rhipicephali IRELQSKQNPFKQLEFAYTNAQY-----------------FDQDIFGNRVEELINNPHILTTA---------------------QQATFLITEDTHLRKT

R. aeschlimannii IRELQNKKNPFAQLEFAYTNAQH-----------------FDQDIFGDRVKELINNPNILTIE---------------------QQATFLTKTDTHLRKT

R. montanensis IRELQGKQNPFEQLEFAYTNAKN-----------------FDQDIFGNRVKELINNPNILTIG---------------------QQATFLITEDTNLRKT

R. slovaca 13-B IRELQGKQNPFEQLEFAYINAKN-----------------FDQDIFGNRVDELINNPNILTLV---------------------QQATFLITEDTNLRKT

R. peacockii Rustic IRELQGKQNPFKQLEFAYINAKN-----------------FDQDIFSNRVDELINNPNILTIV---------------------QQATFLITEDTNLRKT

R. rickettsii Iowa IRELQGKQNPFEQLEFAYINAKN-----------------FDQDIFSNRVDELINNPNILTIV---------------------QQANFLITEDTNLRKT

R. rickettsii Sheila Smith IRELQGKQNPFEQLEFAYINAKN-----------------FDQDIFSNRVDELINNPNILTIV---------------------QQANFLITEDTNLRKT

R. japonica LRELQSKQNLFEQLAFAYTNEKH-----------------FNQDVFGTRVDELITNPNILTIV---------------------QQANFLITEDTNLRKI

R. honei IRELQGKQNPFEQLEFAYINAKN-----------------FDQDIFGNRVEELINNPNILTIV---------------------QQATFLITEDTNLRKT

R. conorii Malish 7 IRELQGKQNPFKQLEFAYINTKN-----------------FDQDIFGNRIDELINNPNILTIV---------------------QQATFLTKEDTNLRKT

R. sibirica 246 IRELQGKQNPFEQLEFAYINAKN-----------------FDQDIFGNRVDELINNPNILTIV---------------------QQATFLITEDTNLRKT

R. africae ESF-5 IRELQGKQNPFEQLKFAYINAKN-----------------FDQDIFGNRVDELINNPNILTIV---------------------QQATFLITEDTNLRKT

R. parkeri IRKLQGKQNPFEQLEFAYINAKN-----------------FDQDIFGNRVDELINNPNILTIV---------------------QQATFLITEDTNLRKT

**[1101-1200 RRRRRRRRRRRRRRRRRRRRRRRRRRRRRRRRRRRRRRRRRRRRRRRRRRRRRRRRRRRRRRRRRRRRRRRRRRRRRRRRRRRRRRRRRRRRRRRRRRRR]**

**[ | | | | | | | | | | ]**

R. bellii RML369-C NELEDAKRQLDELKS-------------------------------------------------------------------------------------

R. bellii OSU 85-389 NELEDAKRQLDELKS-------------------------------------------------------------------------------------

R. prowazekii Madrid E ----------------------------------------------------------------------------------------------------

**R. typhi Wilmington TTEDQEQFKTDNIAAESLEDMAVMFKDSEEAAERKEEVTL-------NYSPN---------------GMDSKTTEDQEQFKTDNIAAESLE---------**

R. akari Hartford IVEDEVKRKAENVQAERLEDMDAVFKNAKDEAEHKEKANRQRQEELKRKQDEVNRKIVEDEVE----RKAENAQAERLEDMDAMFKNAKDEAEYKEKANR

R. akari Hartford 2 IDTDQAQAKLDDLRKAILSTIKLEELIKA-NLPHNEFIAI-----VKEKEPELLKEFLKANTI----KLEGNNNLDQLRLVLPSFTCMSDE-QLRVLASK

R. australis INADQAQAKLDDLRTAILSTIKFEELITV-NLPKNEFIAI-----VKEKEPELLKEFLKATTI----KLEGNNNLDQLRLVLPSFTGMSNE-QVRILASK

R. felis URRWXCal2 IPT--AKANLNKLKQNILEKIEIQQLIANKDISRKDLLDI-----LNNKNPELLKSLLEAKVILEENKLNNSANEVDLKEIIPSLNYLTSE-QLTSLINR

R. canadensis McKiel IHSDQAQAKLDNLRTAILSTIKFEELTTA-NLSQNDFIAI-----VKEKDPELLKEFLKATII----KHESNNNLDQLRLALPSFTGMSDQ-QVRILSSK

REIS ----------------------------------------------------------------------------------------------------

R. massiliae MTU5 INSDQAQAKLDDLRTAILSTIKFEELIKA-NLPQHEFIAI-----VKEKEPELLQEFLNATTI----KLTGNNNLDQLRLALPSFTGMSNE-QVRILTSK

R. rhipicephali INSDQAQAKLDDLRTAILSTIKFEELITA-NLPQHAFIAI-----VKEKKPELLQEFLNATTI----KLTGNNNLDQLRLALPSFTGMSNE-QIRILTRK

R. aeschlimannii INSDQAQAKLDDLRTAILSTIKFEELITV-DLSQHEFIAI-----VKEKKPALLQELLQAITI----KLT-NNNSDQLRLALPSFTGMSDE-QLRILTSK

R. montanensis INSDQAQAKLDDLRTAILSTIKFEELITA-NLPQHEFIAI-----VKEKKPELLKEFLKATTI----KLTGNNNLDQLRLALPSFTDMSNE-QVRILASK

R. slovaca 13-B INSDQAQAKLDDLRTAILSTIKFEELITA-NLPQHDFIAI-----VKEKDPELLKEFLKATTL----KLTGNNNLDQLRLALPSFTDMSNE-QIRILSNK

R. peacockii Rustic INSDQAQAKLDDLRTAILSTIKFEELITA-KLPQHDFIAI-----VKEKDPELLKEFLKATTL----KLTGNNNLDQLRLALPSFTDMSNE-QIRILANK

R. rickettsii Iowa INSDQAQAKLDDLRTAILNTIKFEELITT-NLPQHDFIAI-----VKEKDPALLQEFLKATTL----KLTGNNNLDQLRLALPSFADMSNE-QIRILANK

R. rickettsii Sheila Smith INSDQAQAKLDDLRTAILNTIKFEELITT-NLPQHDFIAI-----VKEKDPALLQEFLKATTL----KLTGNNNLDQLRLALPSFADMSNE-QIRILANK

R. japonica INSDQAQDKLDKLRTAILNTIKFEELITA-NLPHNEFIAI-----VKEKDPELLKELLKASTI----KLTGNNNLDQLRSALPSFTDMSNE-QISILASK

R. honei INSDQAQAKLDDLRTAILSTIKFEELITA-NLPQHDFIAI-----VKEKDPELLKEFLKATTI----KLTDNNNLDQLRLALPSFTGMSNE-QIRILTSK

R. conorii Malish 7 INSDQAQAKLDDLRTAILSTIKIEELITA-NLPQHDFIAI-----VKEKDPELLKEFLKATTL----TVTGNNNLDQLRLALPSFTGMSNE-QIRILSNK

R. sibirica 246 INSDQAQAKLDDLRTAILSTIKFEELITA-NLPQHDFIAI-----VKEKDPELLKEFLKATTL----KVTGNNNLDQLRLALPSFKGMSNE-QIRILSNK

R. africae ESF-5 INSDQAQAKLDDLRTAILSTIKFEELITT-NLSQHDFIAI-----VKEKDPELLKEFLKATTL----KVTGNNNLDQLRLALPSFTGMSNE-QIRILSNK

R. parkeri INSDQAQAKLDDLRTAILSTIKFEELITA-NLPQHDFIAI-----VKEKDPELLKEFLKATTL----KVTGNNNLDQLRLALPSFTGMSNE-QIRILSNK

**[1201-1300 RRRRRRRRRRRRRRRRRRRRRRRRRRRRRRRRRRRRRRRRRRRRRRRRRRRRRRRRRRRRRRRRRRRRRRRRRRRRRRRRRRRRRRRRRRRRRRRRRRRR]**

**[ | | | | | | | | | | ]**

R. bellii RML369-C -------------------------------------------------------------------------------------KKITGLFALNNN---

R. bellii OSU 85-389 -------------------------------------------------------------------------------------KKITELFALNNN---

R. prowazekii Madrid E ----------------------------------------------------------------------------------------------------

**R. typhi Wilmington** -**DMAVM**------**FKDSEEAAERKEEVTLNYS**------------------**PNGMDSKTTEDQEQFKTD**-----------**NIAAESLEDMAVMFKDSEEAAE**

R. akari Hartford QRQDEVNRKIVEDEVERKAENAQAERLEDMDAMFKNAKDEAEHKEKA--NRQRQEELKRKQDEVNHKIVEDEVERKAENAQAERLEDMDAMFKNAKDEAE

R. akari Hartford 2 LNMTII------LNALKEYSQVKAKKHINTGNM-------PPPPPPPP-SGLKDAELAYLTTLGITKDWISRIARLNVKTSTSTFKTTPKIYNFSSDIAV

R. australis LNMNII------LKALKEYSQEKAKKHIHTGNM-------PPPPPPPPPSGSKDSELAYLTTLGITKDWISRITRLNVNTSTSTFKITPKIYNFSSDIAV

R. felis URRWXCal2 ITIEGV------KTALKAKWQQENKTVSNNTEKPLIYNNGTPMPPPIPNGNSNFGTNDYLISMGYTQEFIDRMDKVKPNNNFGK-NHNYTATDFKSNVGK

R. canadensis McKiel LNMYVI------LKALQKYEQEESKNHTHTSNM-------PPPPPPP--PGSKILQITYLKNLGITEDWINRITKLNENINTHTSKTVPQVYDFNSDIAL

REIS ----------------------------------------------------------------------------------------------------

R. massiliae MTU5 LNMPII------LKALHECSQEKAKKHIHTGNM-------PPPPPPLP-PDSQDLELAYLTRLGITK----------SNANTSTFKTTPKIYNFSSDIAV

R. rhipicephali LNMPII------LKALHECSQEKAKKHIHTGNM-------PPPPPPLPLPDSQDLELAYLKSLGITK----------SNANTSTFKTTPKIYNFSSDIAV

R. aeschlimannii LNTTKI------LRALQECSQEKVKKHIHTGNMP------PPPPPPPPLPNSQDLESAYLTRLGITR----------FNINTHISKKISEAYKFKSDIGL

R. montanensis LNMPII------LKALKEHSQEKAKKHIHTGSM-------PPPPPPL--PDSQDLELAYLTSLGITK----------FNANTSTFKTTPKIYNFSSDIAV

R. slovaca 13-B LKMPII------LKALKECSQEKAKKHIHTGNM-------PPPPPPL--PDSQDLELAYLTSLGITK----------FNANTSTFKTTPKTYHFSSDIAL

R. peacockii Rustic LKMPII------LKALKECSQEKAKQHIHTGNM--------PPPPPL--PDSQDLELAYLTSLGITK----------FNANTSTFKTTPKTYRFSSDIAL

R. rickettsii Iowa LKMPIILKAIQELKAIQECSQEKAKQHIHTENM-------PPPPPPPPLPNAHDLKLAYLTSLGITK----------FNANTSTFKTTPKTYHFSSDIAL

R. rickettsii Sheila Smith LKMPII------LKAIQECSQEKAKQHIHTENM-------PPPPPPPPLPNAHDLELAYLTSLGITK----------FNANTSTFKTTPKTYHFSSDIAL

R. japonica LKGPII------LKAIKECSQEKAKKPIHTGNM-------PPPPPPL--PDSQDWELAYLKSLGITQ----------FNTNTSTFKTTPKIYHFSSDIAL

R. honei LKMPII------LKALKECSQEKAKKHIHTGNM-------PPPPPPL--PDSQDLELAYLTSLGITK----------FNANTSTFKTTPKTYHFSSDIAL

R. conorii Malish 7 LKMSII------LKALKECSQEKATQYIHTGNMP------PPPPPPPPLPDSQDLELAYLKSLGITK------------ANTSTFKTTPKTYHFSSDIAL

R. sibirica 246 LKMPII------LKALKECSQEKATKYIHTGNM-------PPPPPPL--PDSQDLELAYLTSLGITK----------FNANTSTLKTTPKTYHFSSDIAL

R. africae ESF-5 LKMPII------LKALKECSQEKAPKYIHTGNMP------PPPPPPPPLPDSQDLELAYLTSLGITK----------FNANTSTFKTTPKTYNFSSDIAL

R. parkeri LNLSII------LQALQECSQEKATKYIHTGNM-------PPPPPPPPLPNSQDLESAYLASLGITK-----------STSTFKTKTTPKTYHFRSDIAL

**[1301-1400 RRRRRRRRRRRRRRRRRRRRRRRRRRRRRRRRRRRRRRRRRRRRRRRRRRRRRRRRRRRRRRRRRRRRRRRRRRRRRRRRRRRRRRRRRRRRRRRRRRRR]**

**[ | | | | | | | | | | ]**

R. bellii RML369-C -------------------------------------------------------------------------------------------GENASISFD

R. bellii OSU 85-389 -------------------------------------------------------------------------------------------GENASISFD

R. prowazekii Madrid E ---------------------------------------------------------------------------------------------------L

**R. typhi Wilmington RKEEVTLDYSPNGMDSKTTEDQEQFK**------**TDNIAAESLEDMAVMFKDSEEAAERKEEVTLDYSP**---------**NGMDSKTTEDQEQFKTDNIAAESL**

R. akari Hartford HKEKANRQRQEELKRKQDEVNRKIVEDEVKRKAENVQAERLEDMDAVFKNAKDEAEHKEKANRQRQEELKRKQD---EVNRKIVEDEVERKAENAQAERL

R. akari Hartford 2 RYKEFALSGQKSAGHKAKYSDADLFQKAI---VESVAFEHSKNLSKVHQNNTYFAKIQEAIDTMHSSFIGPRTEIGQEIHNIYTSKLLALTKDKEFIKYV

R. australis RYKEFALSGQKSAGHKAKYSDADLFKKAI---VESVAFEHSKNLSKVHQNNTYFAKIQEAVDTMHSSFIGPRTEIGQKIHNIYTSKLLELTKDKEFIKYV

R. felis URRWXCal2 NYYE---STSKLGGTDILLTDSQKLENAI---KKEVL-------------AKYIEE---------------------PNRDMQDDSLLKQAFEEKFYYAE

R. canadensis McKiel RYNGFALSGQKSAGHKAKYSDADLLQKAI---IESIVLEHSKNVSEVNQNTQYFEKMQEAVSTMKSSFIGPRTEIGQEIHNIYTSKLLELTKDKEFIRYV

REIS ----------------------------------------------------------------------------------------------------

R. massiliae MTU5 RYKEFALSGQKAAGHKAKYSDANLFKKAI---AESVAFEHSKKLPKAHQNNKYFAKIQEAVDTMHSSFIGPRTEIGQEVHNIYTSKLLELAKDKEFIKYV

R. rhipicephali RYKEFALSGQKAAGHKAKYSDANLLKKAI---VESVAFEHSKKLPKAHQNNKYFAKIQEAADTMHSSFIGPRTEIGQEVHNIYTSKLLELAKDKEFIKYV

R. aeschlimannii RYRKFTLSGEKAVGYKAKYSDADLFKKAI---VESVAFEHLKNLPEAHQNKTYFEKIQEAVNTMQPSFIGPRTEIGQEVHNIYTSKVLELTKDKNFIEYI

R. montanensis RYKEFTLSGQKSAGHKAKYSDANLFKKAI---VESVAFEHSKNLSKAHQNNKYFAKIQEAVDTMHSSFIGPRTEIGQKIHNIYTSKLLELTKDKEFIKYV

R. slovaca 13-B RYKEFTLSGQKSAGYKAKYSDADLLKKAI---VESVAFEHSKNLSKAHQNNKYFEQSR-AVDTMHSSYSLELNR--TKIHNIILLNFW-ITKDKEFIKYV

R. peacockii Rustic RYKEFTLSGQKSAGYKAKYSDADLLQKAI---VESVAFEHSKNLSKAHQNNKYFEQIQEAVDTMHPRFIGPRTEIGQKIHNIYTSKLLALTKDKEFIKYV

R. rickettsii Iowa RYKEFTLSGQKSAGYKAKYSDADLLQKAI---VESVAFEHSKNLSKAYQNNKYFEQIQEAVDTMHPRFIGPRTEIGQNIHNIYTSKLLELTKDKEFIKYV

R. rickettsii Sheila Smith RYKEFTLSGQKSAGYKAKYSDADLLQKAI---VESVAFEHSKNLSKAYQNNKYFEQIQEAVDTMHPRFIGPRTEIGQNIHNIYTSKLLELTKDKEFIKYV

R. japonica RYKEFTLSGQKSAGHKAKYSDADLLKKAI---VESVAFEHSKNLSKAHQNNKYFEKIQEAVDTMHSSFIGPRTEIGQKIHNIYTSKLLELTKDKEFIKYV

R. honei RYKEFTLSGQKSAGYKAKYSDADLLKKAI---VESVAFEHSKNLSKAHQNNKYFEQIQEAVDTMQSSFIGPRTEIGQKIHNIYTSKLLELTKDKEFIKYV

R. conorii Malish 7 RYKEFTLSGQKSAGYKAKYSDADLLKKAI---VESVAFEHSKNLSKAHQNNKYFEQIQKAVNTMYSSFIGHRTELEQKIHNIYTSKLLELTKDKEFIKYV

R. sibirica 246 RYKEFTLSGQKSAGYKAKYSDADLLKKAI---VESVAFEHSKNLSKAHQNNKYFEQIQEAVDTMYSSFIGPRTEIGQKIHNIYTSKLLELTKDKEFIKYV

R. africae ESF-5 RYEELALSGPKSAGYKAKYSDADLLKKAI---VESVAFEHSKNLSQAHQNNQYFEQIQEAVDTMHSSFTGHRTEIGQNIHNIYTSKLLELTKDKEFIKYV

R. parkeri KYKEFTLSGQKSAGYKAAYSDADLLKKAI---VESVAFEHSKNLSEAHQNTDYFEQVEEAVNTMHSSFTGPRTEMGQIIHNIYISKLLELTKDKEFIKYV

**[1401-1500 RRRRRRRRRRRRRRRRRRRRRRRRRRRRRRRRRRRRRRRRRRRRRRRRRRRRRRRRRRRRRRRRRRRRRRRRRRRRRRRRRRRRRRRRRRRRRRRRRRRR]**

**[ | | | | | | | | | | ]**

R. bellii RML369-C QDLIDILKILKE------------------------------------------------VPDFVSI---------------------------------

R. bellii OSU 85-389 QDLIDILKILKE------------------------------------------------VPDFVSI---------------------------------

R. prowazekii Madrid E QRQIILNHNQNE---------------------------------------------------------------------------------------V

**R. typhi Wilmington EDMAVMFKDSEE**----------------**AAERKEEVTLNY**-------------------------------------------------------**SPNGM**

R. akari Hartford EDMAAMFKNAKD----------------EAEYKEKANRQRQDEVNRKIVEDEVERKAENAQAERLEDMDAMFKNAKDEAEHKEKANRQRQEELKRKQDEV

R. akari Hartford 2 EDDIILSKKLTE----AFTSADSDFIGPRTEIGQEVHNIYTQQLAKYP-EETVKEAFNTANSDFIGPRTEIGQEVHNIYKSKLLELAKDKELFL-CVEQL

R. australis EDDIILSKKLTE----AFTSADSDFIGPRTEIGQEVHNIYTQQLTKYP-EETVKEAFNTANSDFIGPRTEIGQEVHNIYKSKLLELAKDKKLFL-FVEQL

R. felis URRWXCal2 DKNTKVIPKPSE------VNFDPNFIGPRTEVGQEIYELYEQELLKLA-----------RDPVFI-------EYVKNNNNTQ----KDERELLISFIEQI

R. canadensis McKiel EDNVKPSKKRTDALIAAFTSVNSDFIGP---ITEDIHNIFTQQMTKYT-EEEVREAFNTANSDFIGPRTEIGQEVHNIYNSKLLELTKDKELLL-FVEEL

REIS ----------------------------------------------------------------------------------------------------

R. massiliae MTU5 KDDIILSKKLTE----VFTSADSDFIDPRTEIEQKIHNIYTQQLTKYP-EEAVKEAFTSADSDFIGPRTEIGQEVRNIYTSQLLELAKDKELFL-FVTQL

R. rhipicephali KDDIILSKKLTE----AFTSVDSDFIDPRTKLEQKIHNIYTQQLTKYP-EEAVKEAFNTANSDFIGPRTEIGQEVRNIYTSKLLELTKDKELFL-FVAQL

R. aeschlimannii EGNIIPKQKLTE----AFNTANSDFIGPRTEIGQEIHNIYTQQLTKYP-EEAVKEAFNTANSDFIGPRTEIGQEVHNIYTSKLLESAKDKELFL-SVEQL

R. montanensis EDDIILSKKLTE----AFTSADSDFIGPRTEIGQKIHNIYTQQLTKYP-EEAVKEAFNTANSDFIGPRTEIGQAVHNIYKSQLLELAKDKELFL-FVEQL

R. slovaca 13-B KDNIILSKKLTE----AFTSADSDFIGPRTELGQKIHNIYIQQLTKYP-EEAVKEAFNTANPDFIGPRTEIGQEVHNIYTSKLLELAKDKELFL-FAQQV

R. peacockii Rustic EDNIILSKKLTE----AFTSADSDFIDSRTEIGQKIHNIYIQQLTKYP-EEAVKEAFNTAHPDFIGPRTEIGQEVHNIYKSQLLELAKDKELFL-LVQQV

R. rickettsii Iowa KDNIILSKKLTE----VFTSADSDFIGPRTEIGQKIHDIYIQQLTKYP-EEAVKEAFNTAHPDFIGPRTEIGQEVHNIYKSQLLELAKDKELFL-FVQQV

R. rickettsii Sheila Smith ED-IILSKKLTE----AFTSADSDFIGPRTEIGQKIHDIYIQQLTKYP-EEAVKEAFNTAHPDFIGPRTEIGQEVHNIYKSQLLELAKDKELFL-FVQQV

R. japonica EDTIILSKKLTE----AFTSAHSDFIGPRTEIGQEVHNIYTQQLTKYP-EEAVKEAFNTASLDFIGPRTEIGQEVHNIYTSQLSKLAKDKELFL-FVQQI

R. honei EDNIILNKKLTE----AFTSADSDFIDSRTELGQKIHNIYIQQLTKYP-EEAVKEAFNTASLDFIGPRTEIGQKIHNIYKSQLLELAKDKELFL-FVQQV

R. conorii Malish 7 EDNIILNKKLTK----AFTSADSDFIDSRTELEQKIHNIYIQQLTKYP-EEEVKEAFNTASLDFIGPRTEIGQEVHNIYKSQLLELTKDTELCL-FTQQV

R. sibirica 246 EDNIILSKKLTE----AFTSADSDFIDSRTGLGQKIHDIYIQQLTKYP-EEEVKEAFNTANPDFIGPRTEIGQEVHNIYKSQLLELTKDQELSL-FTQQV

R. africae ESF-5 EDNIILSKKLTE----AFTSADSDFTDSRTGLEQKIHDIYIQQLTKYP-EEAVKEAFNTANPDFIGPRTEIGQEVHNIYKSQLLELTKDQELSL-FTQQV

R. parkeri EDNIILNKKLTK----AFTSAGSGFIDSRTRLEQKIHDIYIQQLTKYPEEEAVKEAFNTANPDFIGPRTEIGQEVHNIYKSQLLELTKDQELFL-FTQQI

**[1501-1600 RRRRRRRRRRRRRRRRRRRRRRRRRRRRRRRRRRRRRRRRRRRRRRRRRRRRRRRRRRRRRRRRRRRRRRRRRRRRRRRRRRRRRRRRRRRRRRRRRRRR]**

**[ | | | | | | | | | | ]**

R. bellii RML369-C ----------------------------------------------------------------------------------------------------

R. bellii OSU 85-389 ----------------------------------------------------------------------------------------------------

R. prowazekii Madrid E NSHTTKNQEKFKTD------NVTEEYLEDM----------------------------------------------------------------------

**R. typhi Wilmington DSKTTEDQEQFKTD**------**NIAAESLEDM**----------------------------------------------------------------------

R. akari Hartford NCKMVEDEVERKAE------NAEDEYLEDM----------------------------------------------------------------------

R. akari Hartford 2 LAESAELEQKYGTDLQSEN-NNQEKKVGRLDPKQ------------------------------------------------------------------

R. australis LAESAELEQKYGSDVQSEN-NNQEKKVGRLDPKQ------------------------------------------------------------------

R. felis URRWXCal2 ESKRPELEQKYGSDVQSED-NNQEKKVGHLNMKQ------------------------------------------------------------------

R. canadensis McKiel LTESTNLEQKYGSNVQPENKGKKESLVDKMKKRLQQENKVKNDKSSIESNTKYEDNDDNNKKLELSDSKTTVSQMLSNSNDS------------------

REIS ---------KYNSK--------------------------------------------------------------------------------------

R. massiliae MTU5 LAESTALEQKYGSDIQSEN-SNNEKKIGRLDLKT------------------------------------------------------------------

R. rhipicephali LVESTELEQKYGSDIQSEN-SNNDETIGRLDLKT------------------------------------------------------------------

R. aeschlimannii LVESTEIDKNMVVIFNQKV-SNNRKKTGHLDPGL------------------------------------------------------------------

R. montanensis LVESTELEQKYGSDIHSGTIVNNEKKIGRLDPKK------------------------------------------------------------------

R. slovaca 13-B LVESTELERKYGSDIQSEN-SNNEKKVGRLDQKK------------------------------------------------------------------

R. peacockii Rustic LVESAELEQKYGSDIQSEN-SNDEKKVGRLDQTK------------------------------------------------------------------

R. rickettsii Iowa LVDSAELEQKYGSDIQSEN-SNDEKKVGRLDKTK------------------------------------------------------------------

R. rickettsii Sheila Smith LVDSAELEQKYGSDIQSEN-SNDEKKVGRLDKTK------------------------------------------------------------------

R. japonica LVESTELEQKYGSDIQSEN-SNNEKKIGRLNSKK------------------------------------------------------------------

R. honei LVESTELEQKYGSDIQSEN-SKNEKKVGRLDKKK------------------------------------------------------------------

R. conorii Malish 7 LAEATELEQKYGSDIQSEN-SNNEKKVERLDQEK------------------------------------------------------------------

R. sibirica 246 LAESTELERKYGSDIQSGN-SNNEKKVGRLDQEK------------------------------------------------------------------

R. africae ESF-5 LVESIELEQKYSSDIQSEN-SNNEKKVGLLDQEKLQSFKQVLNQEKLQSFKQVLDQEQLQSFKQVLDQKQLQSFKQELDQEQLQSFKQELDQEQLQSFKQ

R. parkeri LAESTELEQKYGSDIQSEN-SDNEKTVGRLDNEKTVGRLDNEKTVGRLDNEKTVGRLDQEK---------------------------------------

**[1601-1700 RRRRRRRRRRRRRRRRRRRRRRRRRRRRRRRRRRRRRRRRRRRRRRRRRRRRRRRRRRRRRRRRRRRRRRRRRRRRRRRRRRR ]**

**[ | | | | | | | | | | ]**

R. bellii RML369-C ------------------------------------------------------------------IRAHPD----------------------------

R. bellii OSU 85-389 ------------------------------------------------------------------IRAYPD----------------------------

R. prowazekii Madrid E ------------------------------------------------------------------ALMFKNSEDTAEQKEEVNCQHH------------

**R. typhi Wilmington ------------------------------------------------------------------AVMFKDSEEAAEQKEEVNRQHH------------**

R. akari Hartford ------------------------------------------------------------------AAMFKNAEDEAEYKEKANRQRQ------------

R. akari Hartford 2 ------------------------------------------------------------------LRLFLQANESANDESSTKDNTQ------------

R. australis ------------------------------------------------------------------LRLFQQENEYANDESSTKDNTQ------------

R. felis URRWXCal2 --------------------------------------------------F---------------QSLFQQENESANDESSTKDDPQ------------

R. canadensis McKiel ----------------------------------KNDKSLTESDTKYEDNDDNNKKLELSDSKTTVSQMLSDSNDSKNDKSLTESDTKYED---------

REIS ----------------------------------------------------------------------------------------------------

R. massiliae MTU5 ------------------------------------------------------------------LRLFQQKNEATNDESSTKDDTQ------------

R. rhipicephali ------------------------------------------------------------------LRLFQQKNETTNDESSTKDDTQ------------

R. aeschlimannii ------------------------------------------------------------------LKLFEQKNKATNDESLTKDDTQ------------

R. montanensis ------------------------------------------------------------------LRLFQQKNEATNDESSTKDDTQSENSNKKSEQSE

R. slovaca 13-B ------------------------------------------------------------------LQLFKQENEATNDESSTKDDTQ------------

R. peacockii Rustic ------------------------------------------------------------------LRLFQQ-NEATNDESSTKDDTQ------------

R. rickettsii Iowa ------------------------------------------------------------------LRLFQQENEATNDESSTKDDTQ------------

R. rickettsii Sheila Smith ------------------------------------------------------------------LRLFQQENEATNDESSTKDDTQ------------

R. japonica ------------------------------------------------------------------LRLFPQENKATNDESSTKDDTQ------------

R. honei ------------------------------------------------------------------LRLFPQENKATNDESSTKDDTQ------------

R. conorii Malish 7 ------------------------------------------------------------------LQLFKQENEATNDESSTKDDTQ------------

R. sibirica 246 ------------------------------------------------------------------LQSFKQENEATNDASSTKDDTQ------------

R. africae ESF-5 ELDQEQLQSFKQELDQEQLQSFKQVLDQEQLQSFKQELDQEQLQSFKQELDQEQLQSFKQELDQEQLQSFKQENEATNDESSTKDDTQ------------

R. parkeri ------------------------------------------------------------------LQSFKQENEATNDESSTKDDTQ------------

**[1701-1800 ]**

**[ | | | | | | | | | | ]**

R. bellii RML369-C ----------------------------------------------LFPTVFNDPDTLA----------FLEKCSPED----------------------

R. bellii OSU 85-389 ----------------------------------------------LFPTVFNDPDTLA----------FLEKCSPED----------------------

R. prowazekii Madrid E -----------------EEQNRQKQEH------------------------INTEEEAVHKE-----KIIHITEETET----------------------

**R. typhi Wilmington -----------------EEQNRQKQEHNC----------------------LDTEEEVVHKE-----KIADLTAETET----------------------**

R. akari Hartford -----------------DEVNRKMVEDEVE------------------RKAENAEDEAEHKENENRQRQEEQNCQEQE----------------------

R. akari Hartford 2 ----------------PEDSNKKSEEFDSE--------------TALSPRLLSSNDSKNDKSSDN----IDLRSSDEE----------------------

R. australis ----------------PEDSNKKSEESNSE--------------TAVSPRLLSSNDSKNDKSSDNKKSLLDLRSSDEE----------------------

R. felis URRWXCal2 ----------------PEDSNKKSEKSDSE--------------TALSPRLLSSNDSKNDKSSDDKKSLLVLRSSE------------------------

R. canadensis McKiel ----------------NDDNNKKLELSDSK--------------TTVSQMLSDSNDSKNDKSSDDKKSLLALRSSDEE----------------------

REIS ----------------------------------------------------------------------------------------------------

R. massiliae MTU5 ----------------PEDNNKKSEQSDSK--------------TVLSPRLLCSNDSKNDKSSDDKKSLLALRSSDEN----------------------

R. rhipicephali ----------------PEDSNKKSEQSDSK--------------TVLSPRLFCSNDSKNDKSSDDKKSLLALRSSDENDKGYETDESDDKKSLLVLRSSD

R. aeschlimannii ----------------PEDSNKKSEQSDSK--------------TVLSPRSLYSNDSKNDKSSDDKKSLLALRSSDEDDKGYETDESDDKKSPLALRSSD

R. montanensis ENEATNDESLTKDDTQPEDSNKKSEQSDSK--------------TALSPRLLSSNDSKNDKSSDDKKSLLALRSSDED----------------------

R. slovaca 13-B ----------------PEDSNKKSEQSDSK--------------TALSPRLLSSNDSKNDKSSDDKKSLLALRSSDED----------------------

R. peacockii Rustic ----------------PEDSNKKSEQSDSN--------------TALSPRLLSSNDSKNDKSSDYKKSLLALRSSDED----------------------

R. rickettsii Iowa ----------------PEDSNKKSEQSDSK--------------TALSPRLLSSNDSKNDKSSDYKKSLLALRSSDEDDQGYATGYTTDEEELEESNSTT

R. rickettsii Sheila Smith ----------------PEDSNKKSEQSDSK--------------TALSPRLLSSNDSKNDKSSDYKKSLLELRSSDEDDQGYATGYTTDEEELEESNSTT

R. japonica ----------------PEDSNKKSEQSDSKSEQSDSKSEQSDSTTALSPRLLSSNDSKNDKSSDDKKSLLALRSSDED----------------------

R. honei ----------------PEDSNKKSEQSDSK--------------TALSPRLLSSNDSKNDKSSDDKKSLLALRSSDED----------------------

R. conorii Malish 7 ----------------PEDSNKKSEQSDSK--------------TALSPRLLSSNDSKNDKSSDDKKSLLALRSSDED----------------------

R. sibirica 246 ----------------PEDSNKKSEQSDSK--------------TALSPRLLSSNDSKNDKSSDDKKSLLALRSSDED----------------------

R. africae ESF-5 ----------------PEDSNKKSEQSDSK--------------TALSPRLLSSNDSKNDKSSDDKKSLLALRSSDED----------------------

R. parkeri ----------------PEDSNKKSEQSDSK--------------TALSPRLLSSNDSKNDKSSDDKKSLLALRSSDED----------------------

**[1801-1900 ]**

**[ | | | | | | | | | | ]**

R. bellii RML369-C ----------------------------------------------------------------------------------------YENIIIELSLLD

R. bellii OSU 85-389 ----------------------------------------------------------------------------------------YENIIIELSLLD

R. prowazekii Madrid E ------------------------------------------------------------------------------EAFKKEIDL------MDVSFKT

**R. typhi Wilmington ------------------------------------------------------------------------------KVFKKEIALE-ENEAMDVSFKT**

R. akari Hartford --------------------------RKRLAAEEDEQTEKTTRKQEET----------------------------EEEKLKNDIALEGEDEDIGVSFKT

R. akari Hartford 2 --------------------------DKGYETDE-ELKESNNT---------------------------------TKEESQKDIALESEDEAIDVSFTT

R. australis --------------------------DKGYETDE-ELEESNYT---------------------------------TEGESKKDIALESEDEDIDVSFNT

R. felis URRWXCal2 ------------------------------------------------------------------------------EESKKDIALESEDEAIDMSFKT

R. canadensis McKiel --------------------------DEGYETDEKELEESNNT---------------------------------TEEEFKKDIPLESEDKTINMSLET

REIS ----------------------------------------------------------------------------------------------------

R. massiliae MTU5 --------------------------DKGYETDEEELEESNST---------------------------------TDEELKKDIVLESEDEALDVSFKT

R. rhipicephali EDDKGYETDESDDKKSLLALRSSDEDDKGYETDEEELEESNSI---------------------------------TDEELKKDIVLESKDEAIDVSFKT

R. aeschlimannii EDDKGYETDESDDKKSLLALRSSDEDDKGYETDEEELEESDST---------------------------------TNEELKKDIVLESEDEAIDVSFKT

R. montanensis --------------------------DKGYETNEEELEESNSTTDEKL----------------------------TDEELKKDIVLESEDEAIDVSFKT

R. slovaca 13-B --------------------------DKGYATDEEELEESNST---------------------------------TDEELKKDIVLESEDEAIDVSFKT

R. peacockii Rustic --------------------------DKGYATDEEELEESNST---------------------------------TGEELKKDIVLESEDEAIDVSFKT

R. rickettsii Iowa GEE--LKKDISDYKKSLLALRSSDEDDQGYATDEEELEEGNST---------------------------------TGEELKKDIVLESEDEAIDVSFKT

R. rickettsii Sheila Smith GEE--LKKDISDYKKSLLALRSSDEDDQGYATDEEELEEGNST---------------------------------TGEELKKDIVLESEDEAIDVSFKT

R. japonica --------------------------DEGYATDEGELEESNST---------------------------------TDEELKKDIVLESEDEAIDVSFKT

R. honei --------------------------DKGYATDEEELEESNST---------------------------------TDEELKKDIVLESEDEAIDVSFKT

R. conorii Malish 7 --------------------------DTGYATDEEELEESNST---------------------------------TDEELKKDVVLESEDEAIDVSFKT

R. sibirica 246 --------------------------DTGYATDEEELEESNSTTNEELEESNSTTNEELEESNSTTNEELEESNSTTNEELKKDVVLESEDEAIDVSFKT

R. africae ESF-5 --------------------------DTGYATDEEELEESNSTTDGE-----------------------------LKEELKKDVVLESEDEAIDVSFKT

R. parkeri --------------------------DTGYATDEEELEESNST---------------------------------TDEELKKDVVLESEDEAIDVSFKT

**[1901-2000 * AT Domain ]**

**[ | | | | | | | | | | ]**

R. bellii RML369-C SKSQDLESLIVKKQISEE----VTLHNQVASITNKPIHMGI-HGRLLLPTAAITGGDEE**DAINRGVWISGLYGVSNQKAWRSIPKYQGRTSGVTIGMDTE**

R. bellii OSU 85-389 SKSQDLESLIVKKQISEE----VTLHNQVASITNKPIHMGI-HGRLLLPTAAITGGDEE**DAINRGVWISGLYGVSNQKAWRSIPKYQGRTSGVTIGMDTE**

R. prowazekii Madrid E ETITEQDEATQRQQVSDDTSRKVAILVKATSTLHKPVHHNILSDRLKV--TVIGAGDEK**TNVNRGIWISGLYGVNKQGVWKNIPKYQGRTTGLTIGADAE**

**R. typhi Wilmington ETIVEQDEAIQRQQVSDDTSRKVAILVKATSTLHKPVHHNILSDRLKV--TVIGAGDEKTNINRGLWISGLYGVNKQGSWKNIPKYQGRTTGFTIGADAE**

R. akari Hartford EAIVEQDEAAQRQQVSDDTSRKVAILVKATSTLHKPVHHNILSDRLKV--SAIATGDEE**PTIARGVWISGLYGMNKQGTWKNIPKYQGRTTGITIGADIE**

R. akari Hartford 2 EAIAEQDEATQRQQVSDDTSRKVAILAKATSTLHKPVHYNILSDRLKV--SAIGAGDEE**PIIARGVWISGLYGMNKQGTWKNIPKYQGRTTGITIGADVE**

R. australis EAIVEQDEAAQRQQVSDDTSRKVAILVKATSTLHKPVHCNILSDRLKV--AAIGAGDEE**PTIARGVWISGLYGVNKQGTWKNIPKYQGRTTGVTIGADAE**

R. felis URRWXCal2 EAIAEQDEATQRQQVSDDTNRKVAILVKATSTLHKPVHYNILSDRLKV--AAIGAGDEE**ASINRGVWISGLYGINKQGTWKNIPKYQGRTTGVTIGADAE**

R. canadensis McKiel EEIARQDEAVLKEQISKETNNKVAILVKATSTLNKYIHPSILANRLNI--GIIAAGDEE**ASINRGLWISGLYGINKQSAWKNIPKYQGRTTGITIGTDAE**

REIS -----------------------------------------------------------**---------------QQQNTWKDLLQMK-------------**

R. massiliae MTU5 EAITEQDEATQRQQVSDDTSRKVAILVKATSTLHKPVHYNILSDRLKV--AAIGAGDEE**ASINRGVWISGLYGINTQETWKNIPKYQSRTTGITIGTDAE**

R. rhipicephali EAITEQDEATQRQQVSDDTSRKVAILVKATSTLHKPVHYNILSDRLKV--AAIGAGDEE**ASINRGVWISGLYGINTQETWKNIPKYQSCTTGITIGTDAE**

R. aeschlimannii EAITEQDEATQKQQVSDDTSRKVTILVKATSTLHKPIHYNILSDRLKV--AAIGAGDEE**ASINRGVWISSLYDIRKQKTWKNIPKYQSRTTSITIGTDAE**

R. montanensis EAITEQNEATQRQQVSDDTSRKVAILVKATSTLHKPVHYNILSDRLKV--AAIGAGDEE**ASINRGVWISGLYDINKQGTWKNIPKYQSRTTGITIGTDAE**

R. slovaca 13-B EAITEQDEVTQRQQVSDDTSGKVAILVQATSTLHKPVHYNI-NDRLTV--AAIGAGDEE**TIINRGVWISGLYGINKQRIWKNIPKYQNRTTGITIGTDAE**

R. peacockii Rustic EAITEQDEVTQRQQVSDDTSGKVAILVQATSTLHKPVHYNI-NDRLKV--AAIGAGDEE**TSINRGVWISGLYGISKQRIWKNIPKYQNRTTGITIGTDAE**

R. rickettsii Iowa EAITEQDKVTQRQQVSDDTSGKVAILVQATSTLHKPVHYNI-NDRLTV--AAIGAGDEE**TSINRGVWISGLYGINKQSIWKNIPKYQNRTTGITIGADAE**

R. rickettsii Sheila Smith EAITEQDKVTQRQQVSDDTSGKVAILVQATSTLHKPVHYNI-NDRLTV--AAIGAGDEE**TSINRGVWISGLYGINKQSIWKNIPKYQNRTTGITIGADAE**

R. japonica EAITEQDEATQRQQVSGDTSGKVAILVKATSTLHKPVHYNI-SDRLKV--TAIGAGDEE**TSINRGVWISGLYGINKQSTWKNIPKYQNRTTGITIGTDAE**

R. honei EAITEQDEVTQRQQVSDDTSGKVAILVQETSTLHKPVHYNI-NDRLTV--AAIGAGDEE**TSINRRVWISGLYGINKQRIWKNIPKYQNRTTGITIGTDAE**

R. conorii Malish 7 EAITEQDEVTQRQQVSDDTSGKVAILVQATSTLHKPVHYNI-NDRLTV--AAIGAGDEE**TSINRGVWISGLYGINKQRIWKNIPKYQNRTTGITIGTDAE**

R. sibirica 246 EAITEQDEVTQRQQISDDTSGKVAILVQATSTLHKPVHYNI-NDRLTV--AAIGAGDEE**TSINRGVWISGLYGINKQRIWKNIPKYQNRTTGITIGIDAE**

R. africae ESF-5 EAITEQDEVTQRQQISDNTSGKVAILVQATSTLHKPVHYNI-NDRLTV--AAIGAGDEE**TSINRGVWISGLYGINKQRIWKNIPKYQNRTTGITIGTDAE**

R. parkeri EAITEQDEVTQRQQVSDYTSGKVAILVQATSTLHKPVHYNI-NDRLTI--AAIGAGDEE**TSINRGVWISGLYGINKQRIWKNIPKYQNRTTGITIGTDAE**

**[2001-2100 ]**

**[ | | | | | | | | | | ]**

R. bellii RML369-C **LSNSSDVIGIAYSRIESHFKYNKKFAKTA-LNGHLLSVYGLKELPKNFSLQAIASVGHNYIKNKATTANNIIGKYQNNNFNFEA-LLNYKYRINYNL-YL**

R. bellii OSU 85-389 **LSNSSDVIGIAYSRIESHFKYNKKFAKTA-LNGHLLSVYGLKELPKNFSLQAIASVGHNYIKNKATTANNIIGKYQNNNFNFEA-LLNYKYRINYNL-YL**

R. prowazekii Madrid E **FINNHDVIGIAYSNLESKIKYNKKLGKTA-VHGHLLSVYGLKELVKGFSLQSITSYGHNYIKNKSKNLNKIIGKYQNNN--FQT-LLNYKYRTKYNL-HF**

**R. typhi Wilmington FINNHDVIGIAYSNLESKIKYNKKLGKTA-VYGHLLSIYGLKELIPGFSLHTIVSYGYNYIKNKSKNLDKIIGKYQNNNLSFQT-LLNYKYCTKYNL-HF**

R. akari Hartford **FINSHDVIGIAYSRLESKIKYNEKLGKTA-VNVHLFSIYGLKELNKGFSLQAITSYGHNYIKNKSKSINNIVGKYQNNNFSFQT-LLNYKYRTKDDL-HF**

R. akari Hartford 2 **FINSHDVIGIAYSRLESKIKYNKKLGTIA-VNGHLFSIYGLKELIKDLSLHAITSYGHNYVKNKSKNINNIIGKYQNNNLSFES-LLNYKYHTKYDL-HF**

R. australis **FINSHDVIGIAYSRLESQIKYNKKLGKTAMLTVTLLSIYGLKELIKGFSLQAITSYGHNYIKNKSKSINNIIGKYQNNNLSLRNVLLNYKYHTTYYLKHF**

R. felis URRWXCal2 **FINSHDVIGIAYSRLEFQIKYNKKLEKTA-VNGHLLSIYGLKELIKGFSLQAITSYGHNYIKNKSKSINNIIGKYQNNNLSFQT-LLNYKYRTKYDL-HF**

R. canadensis McKiel **FINSHDVIGIAYSRLNSQVKYNKKLGKTA-INGHLLSIYGQKELIKGFTLQAITTYSHNYIKNKSISTNNIIGKYQNNNVSFES-LLNYKYRTKYDL-YL**

REIS **---------------------NQKMARSR----------------------------------------------------FSS----------------**

R. massiliae MTU5 **FINSHDVIGIAYSRLESLIKYNKKLGKTT-VNGHLLSIYGLKELIKGFSLQAITSYGHNYIKNRSKSINNIIGKYQNNNLSFQT-LLNYKYRTKYDL-YF**

R. rhipicephali **FINRHDVIGIAYSRLESLIKYNKKLGKTT-VNGHLLSIYGLKELIKGFSLQAITSYGHNYIKNRSKSINNIIGKYQNNNLSFQT-LLNYKYRTKYDL-YF**

R. aeschlimannii **FINSHDVIGIAYSRLESLIKYNKKLGKTT-VNGHLLSIYGLKELIKGFSLQAITSYGHNYIKNRSKSIDNIIGKYQNNNLNFQT-LLNYKYRTKYDL-YF**

R. montanensis **FINSHDVIGIAYSRLESQIKYNKKLGKTA-VNGHLLSIYGLKELIKGFSLQAITSYGHNYIKNRSKSINNIIGKYQNNNLSFQT-LLNYKYRTKYDL-HF**

R. slovaca 13-B **FINSHDVIGIAYSRLESQINYNKKLGKTA-VNGHLLSIYSLKELIKGFSLQTITSYGHNYIKNRSKNINNIIGKYQNNNLSFQT-LLNYKYRTKYDL-HF**

R. peacockii Rustic **FINSHDVIGIAYSRLASQIKYNKKLGKTA-VNGHLLSIYSLKELIKGFSLQTITSYGLNYIKNRSKNINNIIGKYQNNNLSFQT-LLTYKYRTKYDL-QF**

R. rickettsii Iowa **FINSHDVIGIAYSRLASQIKYNKKLGKTA-VNGHLLSIYSLKELIKGFSLQTITSYGHNYIKNRSKNINNIIGKYQNNNLSVQT-LLNYKYRTKYDL-HF**

R. rickettsii Sheila Smith **FINSHDVIGIAYSRLASQIKYNKKLGKTA-VNGHLLSIYSLKELIKGFSLQTITSYGHNYIKNRSKNINNIIGKYQNNNLSVQT-LLNYKYRTKYDL-HF**

R. japonica **FINSHDVIGIAYSRLESQIKYNKKLGKTV-VNGHLLSIYGLKELIKGFSLQAITSYGHNYIKNRSKSINNIIGKYQNNNLSFQT-LLNYQYRTKYDL-HF**

R. honei **FINSHDVIGIAYSRLESQIKYNKKLGKTT-VNGHLLSIYSLKELIKGFSLQTITSYGHNYIKNRSKNINNIIGKYQNNNLSFQT-VLNYKYRTKYDL-HF**

R. conorii Malish 7 **FINSHDVIGIAYSRLESQIKYNKKLGKTT-VNGHLLSIYSLKELIKGFSLQTITSYGHNYIKNRSKNINNIIGKYQNNSLSFQT-LLNYKYRTKYDL-HF**

R. sibirica 246 **FINSHDVIGIAYSRLESQIKYNKKLGKTT-VNGHLLSIYSLKELIKGFSLQTITSYGHNYIKNRSKNINNIIGKYQNNNLSFQT-LLNYKYRTKYDL-HF**

R. africae ESF-5 **FINSHDVIGIAYSRLESQIKYNKKLGKTT-VNGHLLSIYSLKELIKGFSLQTITSYGHNYIKNRSKNINNIIGKYQNNNLSFQT-LLNYKYRTKYDL-HF**

R. parkeri **FINSHDVIGIAYSRLESQIKYNKKLGKTT-VNGHLLSIYSLKELINGFSLQTITSYGHNYIKNRSKNINNIIGKYQNNNLSFQT-LLNYKYRTKYDL-HF**

**[2101-2200 ]**

**[ | | | | | | | | | | ]**

R. bellii RML369-C **IPNIGLKYDYSRSSGYKGNNF-VQKLMIQKKSNRLLTTSLG**SKVEFKSIKVLNDITLVPSLYGSIENHFYNKDTKVNAKAVLNNQIIEEK-IIISKQPKF

R. bellii OSU 85-389 **IPNIGLKYDYSRSSGYKGNNF-VQKLMIQKKSNRLLTTSLG**SKVEFKSIKVLNDITLVPSLYGSIENHFYNKDTKVNAKAVLNNQIIEEK-IIISKQPKF

R. prowazekii Madrid E **IPSIGFKYDYSRASNYKEYNVDIENLMIQKKSNQSFESSIG**AKIVSKPIISKNNIILTLSAHGNIERHFNNKNTKVNAKATFKKQTLQET-IIIPKQPKL

**R. typhi Wilmington IPSIGFKYDYSRASNYKEYNIDIENLMIQKKSNQSFESSIGGKIVSKPIIITNNTILTLSVHGNIERHFNNKNTKVNAKATFKKQTLQET-IIIPKQPKF**

R. akari Hartford **IPNIGFKYDYSRASSYKEYNVDIENLVIQKKSNQLFESSIG**GKIVFKPIATISNVVLTPSLYGNIERHFNHKNTKVNAKATFKGQILQET-IIIPKQPKL

R. akari Hartford 2 **IPNIGFKYDYSRASNYKEYNVDIENLMIQKKSNQLFESSIG**GKIVFKPIATISNVVLTPSLYGNIERHFNHKNTKVNAKATFKGQILQET-IIIPKQPKL

R. australis **IPNIGFKYDYSRASNYKEYNVDIENLMIQKKSNQSFESSIG**GKIVFKPIATISNIVLTPSLYGNIERHFNNKNTKVNAKATFKGQTLQETNYYTWKQPKL

R. felis URRWXCal2 **IPNIGFKYDYSRASNYKEYNVDIENLMNQKKSNQSFESSIG**GKIVFKPIATVNNIILTPSLYGNIERHFNNKNTKVNAKATFKGQTLQET-IIIPKQPKL

R. canadensis McKiel **IPNISLKYDYSRASNYKEYNVDIENLMIQKKSNQSFESSLG**GKIVFKPIATISNIVLTPSLYGNIEYHFNNKNTKVNAKATFKGQALQEI-IILPKQPKL

REIS **-----------------------------------------**-----------------------------------------------------------

R. massiliae MTU5 **IPNIGFKYDYSRASNYKEYNVDIENLMIQKKSNQSFESSLG**GKIVFKPIVTTNNIVLTPSLYGNIEHHFNNKNTKVNAKATFKGQTLQEK-IIIPKQPKL

R. rhipicephali **IPNIGFKYDYSRASNYKEYNVDIENLMIQKKSNQSFESSLG**GKIVFKPIVTTNNIVLTPSLYGNIEHHFNNKNTKVNAKATFKGQTLQEK-IIIPKQPKL

R. aeschlimannii **IPNIGFKYDYLRASNYKEYNVDIENLMIQKKSNQSFESSLG**GKIVFKPIVTKTI----------------------------------------------

R. montanensis **IPNIGFKYDYSRASNYKEYNVDIENLLIQKKSNQLFESSIG**SKIVFKPIVTTNNIVLTPSLYGNIEHHFNNKNTKVNAKATFKGQTLHEK-IIIPKQPKL

R. slovaca 13-B **IPNISFQYDYSRASNYKEYNVDIENLMIQKKSNQLFESSLG**GKIVFKPIVTTNNIVLTPSLYGNIEHHFNNKNTKVNAKATFKGQTLQET-IITLKQPKL

R. peacockii Rustic **IPNIGFQYDYSRASNYKEYNVDIENLMIQKKSNQLFESSLG**GKIVFKPIVTTNNIVLTPSLYGNIEHHFNNKNTKVNAKATFKGQTLQET-IITLKQPKL

R. rickettsii Iowa **IPNIGFQYDYSRASNYKEYNVDIENLMIQKKSNQLFESSLG**GKIVFKPIVTTNNIVLTPSLYGNIEHHFNNKNTKVNAKATFKGQTLQET-IITLKQPKL

R. rickettsii Sheila Smith **IPNIGFQYDYSRASNYKEYNVDIENLMIQKKSNQLFESSLG**GKIVFKPIVTTNNIVLTPSLYGNIEHHFNNKNTKVNAKATFKGQTLQET-IITLKQPKL

R. japonica **IPNIGFQYDYSRASNYKEYNVDIENLMIQKKSNQLFESSLG**GKIVFKPIVTKNNIVLTPSLYGNIEYHFNNKNTKVNAKATFKGQTLQET-IITLKQPKL

R. honei **IPNIGFQYDYSRASNYKEYNVDIENLMIQKKSNQLFESSLG**GKIVFKPIVTTNNIVLTPSLYGNIEHHFNNKNTKVNAKATFKGQTLQET-IITLKQPKL

R. conorii Malish 7 **IPNIGFQYDYSRASNYKEYNVDIENLMIQKKSNQLFESSLG**GKIVFKPIVTTNNIVLTPSLYGNIEHHFNNKNTKVNAKATFKGQTLQET-IITLKQPKL

R. sibirica 246 **IPNIGFQYDYSRASNYKEYNVDIENLMIQKKSNQLFESSLG**GKIVFKPIVTTNNIVLTPSLYGNIEHHFNNKNTKVNAKATFKGQTLQET-IITLKQPKL

R. africae ESF-5 **IPNIGFQYDYSRASNYKEYNVDIENLMIQKKSNQLFESSLG**GKIVFKPIVTTNNIVLTPSLYGNIEHHFNNKNTKVNAKATFKGHTLQET-IITLKQPKL

R. parkeri **IPNIGFQYDYSRASNYKEYNVDIENLMIQKKSNQLFESSLG**GKIVFKPIVTTNNIVLTPSLYGNIEHHFNNKNTKVNAKATFKGQTLQET-IITLKQPKL

**[2201-2242 ]**

**[ | | | | | ]**

R. bellii RML369-C GYNIGGNVLLTKKNINVVFEYNHYTHKKYKSHQGLVKLKINL

R. bellii OSU 85-389 GYNIGGNVLLTKKNINVVFEYNHYTHKKYKSHQGLVKLKINL

R. prowazekii Madrid E GYNIGNNILMSIKNINVLLEYNYYTHKKYHSHQGLVKLKVNL

**R. typhi Wilmington GYNIGNNILMSIKNINVLYEYNYYTHKKYHSHQGLIKLKINL**

R. akari Hartford GYNIGSNMLMSRKNINVLLEYNYYTHRKYQSHQGLVKLKVNL

R. akari Hartford 2 GYNIGSNMLMSRKNINVLLEYNYYTHRKYQSHQGLVKLKVNL

R. australis GYNIGSNILMSRKNINVLLEYNYYTHKKYQSHQG--------

R. felis URRWXCal2 GYNIGSNILMSKKNINVLLEYNYYTHRKYQSHQGLIKLKVNL

R. canadensis McKiel GYNIGSNILMSRKNINVLLEYNYYTHKKYQSHQGLVKLKVNL

REIS ------------------------------------------

R. massiliae MTU5 GYNIGSNILMSRKNINVLLEYNYYTHKKYQSHQGLIKLKVNL

R. rhipicephali GYNIGSNILMSRKNINVLLEYNYYTHKKYQSHQGLL------

R. aeschlimannii ------------------------------------------

R. montanensis GYNIGSNILMSRKNINVLLEYNYYTHRKYQSHQGL-------

R. slovaca 13-B GYNIGSNILMSRKNINVLLEYNYYTHRKYQSHQGLY------

R. peacockii Rustic GYNIGSNILMSRKNINVLLEYNYYTHRKYQSHQGLIKLKVNL

R. rickettsii Iowa GYNIGSNILMSRKNINVLLEYNYYTHRKYQSHQGLMKLKVNL

R. rickettsii Sheila Smith GYNIGSNILMSRKNINVLLEYNYYTHRKYQSHQGLMKLKVNL

R. japonica GYNIGSNILMSRKNINVLLEYNYYTHRKYQSHQGL-------

R. honei GYNIGSNILMSRKNINVLLEYNYYTHRKYQSHQGL-------

R. conorii Malish 7 GYNIGSNILMSRKNINVLLEYNYYTHRKYQSHQGLIKLKVNL

R. sibirica 246 GYNIGSNILMSRKNINVLLEYNYYTHRKYQSHQGLIKLKVNL

R. africae ESF-5 GYNIGSNLLMSRKNINVLLEYNYYTHRKYQSHQGLIKLKVNL

R. parkeri GYNIGSNILMSRKNINVLLEYNYYTHRKYQSHQGL-------

**[1-100 RRRRRRRRRRRRRRRRRRRRRRRRRRRRRRRRRRRRRRRRRRRRRRR]**

**[ | | | | | | | | | | ]**

**R. typhi Wilmington MKKSKILRKFLATASLCGTLFTNSNATGAIIPNSGSVSLNTGAGLVGGVFQNGDIIQIVNGGREIKISADKANAVIGGISALRELPDFGGVEVSQNVSIG**

R. prowazekii Madrid E **MKKSKILRKFLATASLCGTLFTNSNA**TGTIIPNNGSVSLNTDAGLVGGVFNNGDIIQIVNGGREIKISADKANAIIGGINTLKELPDFGGVEVSQNVSIG

R. prowazekii Rp22 **MKKSKILRKFLATASLCGTLFTNSNA**TGTIIPNNGSVSLNTGAGLVGGVFNNGDIIQIVNGGREIKISADKANAIIGGINTLKELPDFGGVEVSQNVSIG

R. felis URRWXCal2 **MKKSKILRKFLTTASLCGTLFTSSNA**AGATIPNLGSVSLSTGAGLVGGVFNNGDIIQIAVGGIGIKISADKANAVVGGINTLIALPAFGGVEVSQNVSIG

**[101-200 RRRRR RRRRRRRRRRRRRRRRRRRRRRRRRRRRRRRRRRRRRRRRRRRRRRRRRRRRRRRRRR]**

**[ | | | | | | | | | | ]**

**R. typhi Wilmington PLSAGENLNTNFGPLKFISNNVTSIITGVDTQTFSNIDFAGKNATLQINKDLNITTKIDNTVAGNNGTITFEGSGVISNHIGFTNSLLGINVGNGEAKID**

R. prowazekii Madrid E PLNAGEDLNTNFGPLKFISNNVTSIITGVGTKTFSNIDFAGKNATLQINKDLNITTKIDNTVAGNNGSITFEGSGIISNHIGYTNSLLGINVGNGEAKIY

R. prowazekii Rp22 PLNAGEDLNTNFGPLKFISNNVTSIITGVGTKTFSNIDFAGKNATLQINKDLNITTKIDNTVAGNNGSITFEGSGIISNHIGYTNSLLGINVGNGEAKIY

R. felis URRWXCal2 PLSATVGLTPNFGPLKFISNNVTSIITGVGNQTFNNIDFAGKNSTLQINNGLNITTQIDNTVAGNNGIITFAGTGTISNNIGLTNSLSQINVGNGEAQIY

**[201-300 RRRRRRRRRRRRRRRRRRRRRRRRRRRRRRRRRRRRRRRRRRRRRRRRRRRRRRRRRRRRRRRRRRRRRRRRRRRRRRRRRRRRRRRRRRRRRRRRRRRR]**

**[ | | | | | | | | | | ]**

**R. typhi Wilmington ASRANNITINAKNINLTHNNSIFTLCDCNITTLKGNINNTTGIDGQGILNLAYDPDSSNIITGDICNIGSLDTINVLRGSATLNSTIVKATNINLQHDTA**

R. prowazekii Madrid E APEANNITINAKNINLTHNNSILTLCDGNITTLKGNINNTTEIDGQGILNLAYDLGSSSIITGDIGNIGSLDTINVLLGSATFNSTILKATNINLKHNTS

R. prowazekii Rp22 APEANNITINAKNINLTHNNSILTLCDGNITTLKGNINNTTEIDGQGILNLAYDLGSSSIITGDIGNIGSLDTINVLLGSATFNSTILKATNINLKHNTS

R. felis URRWXCal2 MPGAGNSIINAANINLTNNNSILTLFDGNVTTLTGNINNTTGVDGQGILNLAHDLASDNIITGDIGNIGSLAAINVLLGSATLNSTILKATNINLQSNTS

**[301-400 RRRRRRRRRRRRRRRRRRRRRRRRRRRRRRRRRRRRRRRRRRRRRRRRRRRRRRRRRRRRRRRRRRRRRRRRRRRRRRRRRRRRRRRRRRRRRRRRRRRR]**

**[ | | | | | | | | | | ]**

**R. typhi Wilmington TLNLGDNIIVIGNIKGINNEEERLNLKVND-QIDNEMIIPINKGMSGKLNLQGNATLNGNIDNLRILKFSGGHDKILNLQGNTEVEHIIFEDSILNSGTI**

R. prowazekii Madrid E TLNLDDNIIVIGNIKG-NNNKDILNFKVHGTNLDNEMIIPAPQKTHGTLNFKGNATLNGNINNLNILKFSGGHGKTLNLQGNTKVDNLVFADSVLDSGTI

R. prowazekii Rp22 TLNLDDNIIVIGNIKG-NNNKDILNFKVHGTNLDNEMIIPAPQKTHGTLNFKGNATLNGNINNLNILKFSGGHGKTLNLQGNTKVDNLVFADSVLDSGTI

R. felis URRWXCal2 VLNLDDDIIVTGNINGAN-------------------------GVNGTLNFIGNATLNGNINNLNILQCSGGNGKILDLQGNITVNSIVFADSVLASGTI

**[401-500 RRRRRRRRRRRRRRRRRRRRRRRRRRRRRRRRRRRRRRRRRRRRRRRRRRRRRRRRRRRRRRRRRRRRRRRRRRRRRRRRRRRRRRRRRRRRRRRRRRRR]**

**[ | | | | | | | | | | ]**

**R. typhi Wilmington TVNGLLNTHVVTFNKSNANGGTLIINAKNTISASLLNAIKAKIQINANLTINHPSAGHIGDIRIADNTTYTINADIGHVNLLQNGAKIMFEGADAMLALI**

R. prowazekii Madrid E SVNGLLDTDCVTFNNSNVNGGTLIINAKNTISAKLLNATKAKIQINANLTMNHPSAGDISDIRIADNTIYTIDAKNGNVNLLNNNAKIIFEGADSMLALI

R. prowazekii Rp22 SVNGLLDTDCVTFNNSNVNGGTLIINAKNTISAKLLNATKAKIQINANLTMNHPSAGDISDIRIADNTIYTIDAKNGNVNLLNNNAKIIFEGADSMLALI

R. felis URRWXCal2 SVNGLLNVGGITFNNSNASGGTLIINTENTINVALLNAIKAKIQINANLTINDPSAGDIGDIRIANNTTYTIDAANGNVNLLNNGAKIIFEGADSELDLV

**[501-600 RRRRRRRRRRRRRRRRRRRRRRRRRRRRRRRRRRRRRRRRRRRRRRRRRRRRRRRRRRRRRRRRRRRRRRRRRRRRRRRRRRRRRRRRRRRRRRRRRRRR]**

**[ | | | | | | | | | | ]**

**R. typhi Wilmington NTSFT-DRTFTIYNNLNQSGNDEYGIVKIEAITKGITISNHSEPYTIGQDNTHRLKELIVEGGGNIIVDNTVFTKLLSINSTGQITFNRTLDLGAGGNIA**

R. prowazekii Madrid E NTGVTADRTFTIYNNLNQSGNDEYGIVKIEAIKKVITIANQSGPYTIGQDNTHRLKELIVEGAGDIIIDDTIFTKLLSINSTGQITFNRTLDLGAGGNIA

R. prowazekii Rp22 NTGVTADRTFTIYNNLNQSGNDEYGIVKIEAIKKVITIANQSGPYTIGQDNTHRLKELIVEGAGDIIIDDTIFTKLLSINSTGQITFNRTLDLGAGGNIA

R. felis URRWXCal2 NTSNINDRQFTFYNNLNKSGNDEYGIVRIGATTKDLTIENNGGPYTIGQDNTHRLKEFIVDGAGNIVVDNTVFTKLLSMNSTGQVTFNQALDLGAGGNIA

**[601-700 RRRRRRRRRRRRRRRRRRRRRRRRRRRRRRRRRRRRRRRRRRRRRRRRRRRRRRRRRRRRRRRRRRRRRRRRRRRRRRRRRRRRRRRRRRRRRRRRRRRR]**

**[ | | | | | | | | | | ]**

**R. typhi Wilmington FGKNGTLVVHGVAGSITTSENNQGILTINSGNVTGTIGANGLGLKLVNIGAGSVTCSANVFAPVALTNPSSILILADGVTLTGTVTTHNNTKGILSLGIG**

R. prowazekii Madrid E FGKHGTLVVNGVTGSITTSENNQGILTINSGNITGVIGTNELGLKLVNIGADPVTCSANVFASVALTNPSSVLILADGVTLTGEVTTHNNTKGVLSLGTG

R. prowazekii Rp22 FGKHGTLVVNGVTGSITTSENNQGILTINSGNITGVIGTNELGLKLVNIGADPVTCSANVFASVALTNPSSVLILADGVTLTGEVTTHNNTKGVLSLGTG

R. felis URRWXCal2 FGKDGTLVVNGVTGSVTTSANNQGTLTINSGNINGTIGSNGSSLKLVNIGANLVTFSANVFAPVVLTNAGSALTLADGVTLTGAVTTNNNTKGILSLGVG

**[701-800 RRRRRRRRRRRRRRRRRRRRRRRRRRRRRRRRRRRRRRRRRRRRRRRRRRRRRRRRRRRRRRRRRRRRRRRRRRRRRRRRRRRRRRRRRRRRRRRRRRRR]**

**[ | | | | | | | | | | ]**

**R. typhi Wilmington SNVTGLIGENNATLEKINIGAGASNIDNNIYAGSMVLTDKTSELILNTNVVVNGNITTIAGNNSGKLVFTGNGD--------------------------**

R. prowazekii Madrid E SNITGQIGTNSAALEKINIGAGASNIDSNIYAGSTVLTDQTSELTLNNDVVVNSNIITTAGNNSGKLIFTGNGG--------------------------

R. prowazekii Rp22 SNITGQIGTNSAALEKINIGAGASNIDSNIYAGSTVLTDQTSELTLNNDVVVNSNIITTAGNNSGKLIFTGNGG--------------------------

R. felis URRWXCal2 SNVTGQIGANGAALEKINIGAGASSLGSNIYVGSTVLTNQTSVLTLNNNVVVNGNITTTAGNDSGKLIFAGDGSVISNVGANGAALEEVIFNGVDNIGGT

**[801-900 RRRRRRRRRRRRRRRRRRRRRRRRRRRRRRRRRRRRRRRRRRRRRRRRRRRRRRRRRRRRRRRRRRRRRRRRRRRRRRRRRRRRRRRRRRRRRRRRRRRR]**

**[ | | | | | | | | | | ]**

**R. typhi Wilmington ----------------------------------------------------------------------------------------------------**

R. prowazekii Madrid E ----------------------------------------------------------------------------------------------------

R. prowazekii Rp22 ----------------------------------------------------------------------------------------------------

R. felis URRWXCal2 ANGAVFNVANIAANATVTELMTGDLVYNAAGKVTATGGLTGNIDFQNNAGTFNLGAGSTLTGTVTSTGGVNGTLNVSDAGIITGNIDNLNILEFSGSTGT

**[901-1000 RRRRRRRRRRRRRRRRRRRRRRRRRRRRRRRRRRRRRRRRRRRRRRRRRRRRRRRRRRRRRRRRRRRRRRRRRRRRRRRRRRRRRRRRRRRRRRRRRRRR]**

**[ | | | | | | | | | | ]**

**R. typhi Wilmington ----------------------------------------------------------------------------------------------------**

R. prowazekii Madrid E ----------------------------------------------------------------------------------------------------

R. prowazekii Rp22 ----------------------------------------------------------------------------------------------------

R. felis URRWXCal2 TLDLQGNTRVNSIVFTNNAAASGTINAGGELTLGSLTFNANAVGDVLVINAPSTINGAILNGLKGKIEIKANLTINDPSAGEVNEIDIADNTTYTIDAKN

**[1001-1100 RRRRRRRRRRRRRRRRRRRRRRRRRRRRRRRRRRRRRRRRRRRRRRRRRRRRRRRRRRRRRRRRRRRRRRRRRRRRRRRRRRRRRRRRRRRRRRRRRRRR]**

**[ | | | | | | | | | | ]**

**R. typhi Wilmington ----------------------------------------------------------------------------------------------------**

R. prowazekii Madrid E ----------------------------------------------------------------------------------------------------

R. prowazekii Rp22 ----------------------------------------------------------------------------------------------------

R. felis URRWXCal2 GNIDLLKNGAKIVFEGADSRLDLINTSVTDDRTFTLYNNLNKSGNDEYGIVRIGATTKDLTIANNGGPYTIGQDNTHRLKNFIVDGAGNIVVDNTVFTKL

**[1101-1200 RRRRRRRRRRRRRRRRRRRRRRRRRRRRRRRRRRRRRRRRRRRRRRRRRRRRRRRRRRRRRRRRRRRRRRRRRRRRRRRRRRRRRRRRRRRRRRRRRRRR]**

**[ | | | | | | | | | | ]**

**R. typhi Wilmington ----------------------------------------------------------------------------------------------------**

R. prowazekii Madrid E ----------------------------------------------------------------------------------------------------

R. prowazekii Rp22 ----------------------------------------------------------------------------------------------------

R. felis URRWXCal2 LSMNSTGQVTFNQALDLGAGGNILFGADGELIVKGVTGSVTTSANNQGTLTIQSGNVTGTIGANGSSLKLVNIGANPVTFSDDVFAPVALTNAGSALTLA

**[1201-1300 RRRRRRRRRRRRRRRRRRRRRRRRRRRRRRRRRRRRRRRRRRRRRRRRRRRRRRRRRRRRRRRRRRRRRRRRRRRRRRRRRRRRRRRRRRRRRRRRRRRR]**

**[ | | | | | | | | | | ]**

**R. typhi Wilmington --------------------------------------------------------------------------------------------------IT**

R. prowazekii Madrid E --------------------------------------------------------------------------------------------------IT

R. prowazekii Rp22 --------------------------------------------------------------------------------------------------IT

R. felis URRWXCal2 DGVTLTGAVTTNNNTKGILVLGAGSSVTGGIGGNNAALERVTLGAGASSLGGNIYSGAVALTDQTSILTLQDGAVVNGGITTTAGDGKSKLIFAKDGAVT

**[1301-1400 RRRRRRRRRRRRRRRRRRRRRRRRRRRRRRRRRRRRRRRRRRRRRRRRRRRRRRRRRRRRRRRRRRRRRRRRRRRRRRRRRRRRRRRRRRRRRRRRRRRR]**

**[ | | | | | | | | | | ]**

**R. typhi Wilmington GNIGANGAALQEVVFNGTTNIGGTANSQNFTVAHSEANVVITGLTTGSLKYKDTGTIIANGGLVGDIDFNNKAGKFILG---------------------**

R. prowazekii Madrid E GNIGANGAALQEVVFNGTTNIGGTANSQNFTVAHSAANVVITGLTTGALKYKDTGTIIAHGGLVGDIDFNNKAGKFILG---------------------

R. prowazekii Rp22 GNIGANGAALQEVVFNGTTNIGGTANSQNFTVAHSAANVVITGLTTGALKYKDTGTIIAHGGLVGDIDFNNKAGKFILG---------------------

R. felis URRWXCal2 GNVGAVGSALEEVVFNGADNIGGTANAETFTVANAAANAVITGLATGTLKYTDTGTITANGGWTGDIDFNNKAGTFILGANSTLTGAVSSTGGINGTLKA

**[1401-1500 RRRRRRRRRRRRRRRRRRRRRRRRRRRRRRRRRRRRRRRRRRRRRRRRRRRRRRRRRRRRRRRRRRRRRRRRRRRRRRRRRRRRRRRRRRRRRRRRRRRR]**

**[ | | | | | | | | | | ]**

**R. typhi Wilmington ----------------------------------------------------------------------------------------------------**

R. prowazekii Madrid E ----------------------------------------------------------------------------------------------------

R. prowazekii Rp22 ----------------------------------------------------------------------------------------------------

R. felis URRWXCal2 LGSGSITGVVNGLDIFEFSGSNGTAFDLAGNTTVNNVLFTNSNAASGTININAGLNSGQITFNMGNANGGTLVINAPTTVGDIVNAANGNIIINADFTIT

**[1501-1600 RRRRRRRRRRRRRRRRRRRRRRRRRRRRRRRRRRRRRRRRRRRRRRRRRRRRRRRRRRRRRRRRRRRRRRRRRRRRRRRRRRRRRRRRRRRRRRRRRRRR]**

**[ | | | | | | | | | | ]**

**R. typhi Wilmington ----------------------------------------------------------------------------------------------------**

R. prowazekii Madrid E ----------------------------------------------------------------------------------------------------

R. prowazekii Rp22 ----------------------------------------------------------------------------------------------------

R. felis URRWXCal2 DPDAGGIHNITIKDNTTYTIDSINGNVDLLANGAKIIFEGADSELDLINANDRTFTLHNNLNPSNAQDEFGIVRIGATKKDLTIANNGGPYTIGQDNTHR

**[1601-1700 RRRRRRRRRRRRRRRRRRRRRRRRRRRRRRRRRRRRRRRRRRRRRRRRRRRRRRRRRRRRRRRRRRRRRRRRRRRRRRRRRRRRRRRRRRRRRRRRRRRR]**

**[ | | | | | | | | | | ]**

**R. typhi Wilmington ---------------------------------------------------------------------------DGTTIDGSVLCTGGIAGTLHFIGDG**

R. prowazekii Madrid E ---------------------------------------------------------------------------DGAMIDGSVLCNGGVAGTLDFIGDG

R. prowazekii Rp22 ---------------------------------------------------------------------------DGAMIDGSVLCNGGVAGTLDFIGDG

R. felis URRWXCal2 LKNFIVDGAGNIVVDNTVFTKLLSMNSTGQVTFNQELDLGADGLIVFGDVGTLIANDDVTGDIDFHGKAGTFELDDGATIDGSVLGTGGVAGTLNFIGDG

**[1701-1800 RRRRRRRRR RRRRRRRRRRRRRRRRRRRRRRRRRRRRRRRRRRRRRRRRRRRRRRRRRRRRRRRRRRRRR]**

**[ | | | | | | | | | | ]**

**R. typhi Wilmington NVTQNIGSDTENSISTINIQGDNTKNVTITNDIFVNNIYFTNGGVLQLGGNLTTHNIDFGANGGTLEFNGNNTYNLNAIIVNGQNGILNAFTNLKATDDT**

R. prowazekii Madrid E NVTQNIGADNANSISTINIQGDNTKNVTIANDIFVDNIHFTNGGILQLGGNLTTHNIDFGANGGTLEFNGNNTYNLNAIIVNGQNGILNAFTNLKASDDT

R. prowazekii Rp22 NVTQNIGADNANSISTINIQGDNTKNVTIANDIFVDNIHFTNGGILQLGGNLTTHNIDFGANGGTLEFNGNNTYNLNAIIVNGQNGILNAFTNLKASDDT

R. felis URRWXCal2 NVTGNIGTDAANSPATINIQGDNTKNITIANDIFVGNINFTNGGVLQLSGNLTTPNIDFGAKGGTLEFNGNNTYNLNAVIANGQNGILNVFTTLKSTDAS

**[1801-1900 RRRRRRRRRRRRRRRRRRRRRRRRRRRRRRRRRRRRRRRRRRRRRRRRRRRRRRRRRRRRRRRRRRRRRRRRRRRRRRRRRRRRRRRRRRRRRRRRRRRR]**

**[ | | | | | | | | | | ]**

**R. typhi Wilmington IGTVKIINIGQIGTPQNFTIQVNNKNLTLVSNINSSINFCDSNSQLILSAPVDQTIKFINNLNG--TGGGIITLDGNGNNLTISGNNGIKLGSRDNELSS**

R. prowazekii Madrid E IGTVKIINIGQIGTPQNFTIQVNNKNLTLVSSVNSSINFGDANSQLILSAPVDQTIKFINNLNE--TGGGIITLDSNGNNLTISGNNGIKLGSKGNELSS

R. prowazekii Rp22 IGTVKIINIGQIGTPQNFTIQVNNKNLTLVSSVNSSINFGDANSQLILSAPVDQTIKFINNLNE--TGGGIITLDSNGNNLTISGNNGIKLGSKGNELSS

R. felis URRWXCal2 IGTVKTINIGKVGTPQNFTVQVNNGNLALLSSPNSSINFGDADSQLILSAPIDQTVTFANSLNGIKTGGGIVTLDGNGNNLTVSGNNGATFGTVGNELAS

**[1901-2000 RRRRRRRRRRRRRRRRRRRRRRRRRRRRRRRRRRRRRRRRRRRRRRRRRRRRRRRRRRRRRRRRRRRRRRRRRRRRRRRRRRRRRRRRRRRRRRRRRRRR]**

**[ | | | | | | | | | | ]**

**R. typhi Wilmington LNIKGKVTVTHNLDVQNIHQLNINNGAFFNDHSLTSAKIKNINIGEETGEATYTLDAINCDFDLNTSGMVFKHQDSVLELKNSSNTNDRIITLTSVLDPG**

R. prowazekii Madrid E LNIKGKVTVTNDLDIQNIHQLNINNGALFDDQSLTSAKIKNINIGTVAGGATYTLDAINDNFDLNTSGMVFKHQDSILELKNSSNTNDHTITLTSALDPG

R. prowazekii Rp22 LNIKGKVTVTNDLDIQNIHQLNINNGALFDDQSLTSAKIKNINIGTVAGGATYTLDAINDNFDLNTSGMVFKHQDSILELKNSSNTNDHTITLTSALDPG

R. felis URRWXCal2 LNIKGKVTVTNNLDVQNIQQLNINNGAVFTDQSLTSAKIANISIGEVTGSATYALDAVNSDFDLNTGGIVFEHQDSVLELKNSSNANDRTITLTAALDPG

**[2001-2100 RRRRRRRRRRRRRRRRRRRRRRRRRRRRRRRRRRRRRRRRRRRRR ]**

**[ | | | | | | | | | | ]**

**R. typhi Wilmington NGQSGIIKLITDTNKLTIDNNGNVAYTLGTANHMLKQLTFTSINNGAIALKVGINVENITLNIKDIELNEVNANVLFNKNVTYTATGNINGHVDFQGNAG**

R. prowazekii Madrid E NNQFGIIKLITDTNKLTIDNNGNVAYTLGTANHMLKQLTFASIDNGAIALKVGINVENVTLNIKDIELNEVNANVLFNKNTTYTATGNINGHVDFQGNAG

R. prowazekii Rp22 NNQFGIIKLITDTNKLTIDNNGNVAYTLGTANHMLKQLTFASIDNGAIALKVGINVENVTLNIKDIELNEVNANVLFNKNTTYTATGNINGHVDFQGNAG

R. felis URRWXCal2 NDQFGIVNLTTDTNKLTIDNNGNAAYTLGTVNHRLKQLTFSSTGNGAIALNVGINVENIALNVKTIELGVVNANVLFNKNATYTATGDINGNVDFQGNAG

**[2101-2200 ]**

**[ | | | | | | | | | | ]**

**R. typhi Wilmington VINLDDGIKIDGSVTSTNDVNGTLNLNGSGEVTGLITNIAMLQAGAGDVSLSASGNYSITEIQGNGNNNLTFAANSHLTTDINKTDGQAVNLVFINGGSV**

R. prowazekii Madrid E VINLNDDIEIDGSVTSTGNVNGTLNFNGSGKVTGLINNIVMLQAGAGDVSLSASGNYSITEIQGNGNNNLTFAANSHLTTDINKTGGQDLNLVFINGGSV

R. prowazekii Rp22 VINLNDDIEIDGSVTSTGNVNGTLNFNGSGKVTGLINNIVMLQAGAGDVSLSASGNYSITEIQGNGNNNLTFAANSHLTTDINKTGGQDLNLVFINGGSV

R. felis URRWXCal2 VINLNDGIKIDGIVTSTGNVNGTLNWGGAGEVTGLITNIAMLKAGAGDVLLSAGGNYSITEIQGNGNNDLTFAANSNLTGGINTSGGQAVNLVFQGGGSV

**[2201-2300 ]**

**[ | | | | | | | | | | ]**

**R. typhi Wilmington SGSVGLSKAVGDIIIHAGNVTFNNTVKSSNVVISDGSTMQVNNNITANDISGKNINNGTLKLNNHTPIIITSTLGHNNSIGTIEVANNDVTITGALKAQN**

R. prowazekii Madrid E SGSIGANAAVGDIIINAGSVNFSNTLKSGNIVISDGATMQVNNNVTATDISGKNANNGTLKLNNHTPINITSTLGNNNAIGTIEVANNDVTITGTLQAQN

R. prowazekii Rp22 SGSIGANAAVGDIIINAGSVNFSNTLKSGNIVISDGATMQVNNNVTATDISGKNANNGTLKLNNHTPINITSTLGNNNAIGTIEVANNDVTITGTLQAQN

R. felis URRWXCal2 SGSVGSNAAVGDITIQAGAVNFGSTVKSGNVILSNGATMQVNNNVTATDISGENANDGTLKLNNLAPINITGTLGNNNTIGTIEVANNDATVTGGLKAQN

**[2301-2400 ]**

**[ | | | | | | | | | | ]**

**R. typhi Wilmington IHFSNAAQETTLTLGAASQVTNITTAGNNIHTLEITDLDTGNDGTIGTENNRLKSIELTGNGTITINSKHVYSSITTANNEQGNVKLNIEGGIAYDLGSE**

R. prowazekii Madrid E IHFSNATQAATLTLGAASQVTNITTAGNNIHTLEVTDFDTGNDGIIGDANNRLKSIELTGNGTVTINSPHVYSSITTANNAQGNVKLNIEGGITYDLGSK

R. prowazekii Rp22 IHFSNATQAATLTLGAASQVTNITTAGNNIHTLEVTDFDTGNDGIIGDANNRLKSIELTGNGTVTINSPHVYSSITTANNAQGNVKLNIEGGITYDLGSK

R. felis URRWXCal2 INFSNAAQAATLTLGAAAQVTNITTAGNNIHTLAVTDLDTGN-GVIGAANNRLKAIELTGNGTVTVNSKNFYSGITTANNGQGNVKLNIDGGIVYDLGSK

**[2401-2500 ]**

**[ | | | | | | | | | | ]**

**R. typhi Wilmington IRSLANVQISEDSTVKGDVYSKYLNIDAGKTINFDRGDNNMNPKNLDIPDAIIDLDVLPRSLSLFNYFTDIKADNLNFVDGTATANFKDAVVIDARIDNG**

R. prowazekii Madrid E IKSLANVQISEDTTIRGDVYSKYLNIDAGKTINFDRGDNNMNPKNLDIPDALIDLDVLPRSLSLFNYFTDIKADNLNFADDTATANFKDAVVIDAHIDNG

R. prowazekii Rp22 IKSLANVQISEDTTIRGDVYSKYLNIDAGKTINFDRGDNNMNPKNLDIPDALIDLDVLPRSLSLFNYFTDIKADNLNFADDTATANFKDAVVIDAHIDNG

R. felis URRWXCal2 LGSLASVQVSGNSTVKGDVYSKDINVDAGKTIDFDRGNNNTNPKNLAVPGAIVDLDVLPRSLSLFNYLTDIKADNLNFADATTTANFKDAVLIDAPINNG

**[2501-2600 ]**

**[ | | | | | | | | | | ]**

**R. typhi Wilmington GTLKFNNNVWLTQEIKNANSIEIASNKFMLLQKNIKAATLIADKANLVLLDNVEINTNLNVRDIVLDLANYELKYTGNVTHNGLLTIITYFDTALQKGGH**

R. prowazekii Madrid E GILKFNDNAWLTQEIKNANIIEIASDKFMLLQKNIKAATLIADNANLVLLDNVEVNTNLNVRDIVLDLANYELKYTGNVTHNGLLTIITYFDTALQKGGH

R. prowazekii Rp22 GILKFNDNAWLTQEIKNANIIEIASDKFMLLQKNIKAATLIADNANLVLLDNVEVNTNLNVRDIVLDLANYELKYTGNVTHNGLLTIITYFDTALQKGGH

R. felis URRWXCal2 GTFKFNENVWLKQEITNAKSIEIASNKFMLLEKNIKADTLIADEANLVLLNNLEMNTNLNVRDVVLDVATYELKYTGNVTHNGLLTIITHFDTALQKGGH

**[2601-2700 ]**

**[ | | | | | | | | | | ]**

**R. typhi Wilmington ILVGQGSNVDMSDLDNLIIKIKSRSDITNITSDTKHQVVKLEAGAIYNPVPQTKVIIDASGEQNKFVKWVTDANGFVLLTNT----------DTIGQNNT**

R. prowazekii Madrid E ILVSQGSNVDMSDLDNLIIKIKAHSDITNITSDTKHQIVKLETGAIYTPVPQTKVIIDASEEQNKFVKWVADANGLVLLTDTGGRDDTGGRDDTRGRGNT

R. prowazekii Rp22 ILVSQGSNVDMSDLDNLIIKIKAHSDITNITSDTKHQIVKLETGAIYTPVPQTKVIIDASEEQNKFVKWVADANGLVLLTDTGGRDDTGGRDDTRGRGNT

R. felis URRWXCal2 ILVGNGANVDMSGLDNLIVKIKSRSDITKITSNTKHQVVALEAGGAFTPAPQAKVTIDASGEQNRFVKWVSDANGLVLLANT----------DNEGGDGG

**[2701-2800 ]**

**[ | | | | | | | | | | ]**

**R. typhi Wilmington DNGSLDNGYVGNSSNNNSSNGVVGPSKDKDHGITDIAPIFDPSPILDYTKNNYVASSIANQLINHVKGFGNTTDAGKLLNDLGFMLPNRVTETLDRLSHR**

R. prowazekii Madrid E DNGCRDNCDVGNIS-NNSSNEAGGSSSDKNYGITDVVPIFDPSPILDYTKNNYVASGIANQLINHVKDFGNTTDAGKLLNDLGFMSPNRVTETLDRLSNR

R. prowazekii Rp22 DNGCRDNCDVGNIS-NNSSNEAGGSSSDKNYGITDVVPIFDPSPILDYTKNNYVASGIANQLINHVKDFGNTTDAGKLLNDLGFMSPNRVTETLDRLSNR

R. felis URRWXCal2 NGGDNGGGDGG----NGGGNGGGGSGGGGNSGGGGAVSIFDPSPILDDTKNNQVASGIANQLINNVKGFGNNTDSGKLLNDLGLISPSRVSEALDRLGHR

**[2801-2900 AT Domain ]**

**[ | | | | | | | | | | ]**

**R. typhi Wilmington TNINGLNEGVAGLNGIEIENFLTDIAINMDNFTAEGIGNRLEELIDAGTVNGLNRTNASLNNNNLNLRRLAANNQTVIAAGDEDNTVTGIWGMSFYSKIK**

R. prowazekii Madrid E INVNGLNEGVVGLNGIEVENFLTDIAINMDNFTAKEIGNRLEELSDANTVNGLNKTN-TLLNNKINLKRLNTNNQAIIAAGDED**NIVTGIWGMSFYGKIK**

R. prowazekii Rp22 INVNGLNEGVVGLNGIEVENFLTDIAINMDNFTAKEIGNRLEELSDANTVNGLNKTN-TLLNNKINLKRLNTNNQAIIAAGDED**NIVTGIWGMSFYGKIK**

R. felis URRWXCal2 TNTNGINEGVGEFNGIEIEDLLTDIAINMDNLTSERIGNRLEELGDENTLSGQNEPN---SNQNRRRRTSTTNNQAAVAAGDED**NIGTGIWGIPFYGKAT**

**[2901-3000 ]**

**[ | | | | | | | | | | ]**

**R. typhi Wilmington QNSKNSASGYQSNTGGGIIGFDYNIDNSIVIGAAYTMADSKVKHKNDKSGDKTKAKSNIYSIYGLYHWLNNNFFVEAIGVYGRNKIKNYEKRITAITDQT**

R. prowazekii Madrid E **QNSKNSASGYQSNTGGGIIGFDYNIDNSIVIGAAYTMADSKVKHKNDKNGDRTKAKSNIYSIYGLYNWLTNNFFVEAIGVYGRNKIKNYEKRITTITDQI**

R. prowazekii Rp22 **QNSKNSASGYQSNTGGGIIGFDYNIDNSIVIGAAYTMADSKVKHKNDKNGDRTKAKSNIYSIYGLYNWLTNNFFVEAIGVYGRNKIKNYEKRITTITDQI**

R. felis URRWXCal2 **QKSKNGSSGYKSNTGGGIIGFDYNIDNSIVIGAAYTMADSKVRHKNDKNGDRTKAKSNIYSIYGLYNWLTNNFFVEAIGSYGRNKVKNYEKRLTSTNDQT**

**[3001-3100 ]**

**[ | | | | | | | | | | ]**

**R. typhi Wilmington AIGKFINTFYSYELLGGYNYLVSNRTTITPMFGIRYTTLKNNSYKENNTTFQNLSIKKNYHDKFETILGLNGATHYLAQDIIIKPELHWFINYQYKNKLP**

R. prowazekii Madrid E **AIGKFINTFYSYELLGGYNYLISHRTTITPMFGMRYATFKNNGYKENNTTFQNLSIKKNYYDKFETILGLNSVTHYLSQDIIIKPELHWFINYQCKNKLP**

R. prowazekii Rp22 **AIGKFINTFYSYELLGGYNYLISHRTTITPMFGMRYATFKNNGYKENNTTFQNLSIKKNYYDKFETILGLNSVTHYLSQDIIIKPELHWFINYQCKNKLP**

R. felis URRWXCal2 **AIGKFTNTFYSGELLGGYNYLMSHLTTITPMLGIRYATFKNNSYKENSTTFQNLSIRKNSYNKFETILGLKGVTNYLVQDIIVKSELHGFINYNFKGKLP**

**[3101-3200 ]**

**[ | | | | | | | ]**

**R. typhi Wilmington NIDARLDGIDEPLTTIRFKSAKITYNLGGGISTKNNMIEFGIRYNLSLAKKYTAHQGSLKIKVKL**

R. prowazekii Madrid E **NIDARLDGIDEPLTTIRFKPAKITYNLGGGISTKNNMIEFGIRYNLSLAKKYTAHQG**SLKIKVNL

R. prowazekii Rp22 **NIDARLDGIDEPLTTIRFKPAKITYNLGGGISTKNNMIEFGIRYNLSLAKKYTAHQG**SLKIKVNL

R. felis URRWXCal2 **NIDARLDGIDGPLTTVRFKPAKITYNLGGGISTKYNMMEFGIRYNLSLAKKYMANQG**SLKIKVNL

**[1-100 ]**

**[ | | | | | | | | | | ]**

R. bellii RML369-C ---------------------------------MSKDPNVEEIIKEFDPIANKEFTEAEKQEQTRQEQELF-ESEGDSIFISGADATNASSIPTSSLSAS

R. bellii OSU 85-389 ---------------------------------MSKDPNVEEIIKEFDPIANKEFTEAEKQEQTRQEQELF-ESEGDSIFISGADATNASSIPTSSLSAS

R. prowazekii Madrid E -----------------------------------MSKNGNQDISEFDPL-NREFTEAEKQQQMQQEQEFFSQTILD--IADDGFMV--ASSSQATPSIS

R. prowazekii Rp22 -----------------------------------MSKNGNQDISEFDPL-NREFTEAEKQQQMQQEQEFFSQTILD--IADDGFMV--ASSSQATPSIS

**R. typhi Wilmington -----------------------------------MSKNDNQDISEFDPL-NREFTEAEKQQQMQQEQEFFSQSILD--IVDDGFIV--ASSSQSTPSIS**

R. australis ------------------------------------------MSQDHTGYENDEGYESDIDEKTQEQAAPAQPTLDT---ADDGFSFTPASSTQSTPAIS

R. australis ----------------------------------------------------YENDEEYESGIDEKKQEKAALAQPTLDTADDGFSFTPASSTQSTPSIS

R. felis URRWXCal2 MSKDSDNPGYESGYESDTEEKKQEQAVPAQPISSTANKDGNPDTSEFDPLANKEYTEEQKQKLEQEQKEYFSQTTPQELEADDGFSFTPASSTQSTPSIS

R. canadensis McKiel -----------------------------------MSKDGNPDTSEFDPLANREFTEEEKQQQAKQEQEFFSQTTLD--VADDGLIITAASSTQSTSSIS

R. tamurae ------------------------------------------DTSEFDPLANKEYTEEIGFRDKHLDRPKNFTNYTPELEADDGFIVTSASSAQSTPSMS

R. helvetica ------------------------------------------DTCEFDPL-NREFTEEEKQQQTKQEQELFSQTTPA--TADDGFMV--ASSAQSTPSIS

REIS_1 ----------------------------------------MKMMKDMNPI-----IDEKKQEQAAPAQPTLD-------AADDGFIVTSASSAQSTPSIS

REIS_2 ----------------------------------------------------------------------------------------------------

REIS_3 ----------------------------------------MQNNHNTDEKSAAELLEEFEQKYNYRGSKEVNHTKTDNSDIGMQEQVTEETTAQDSVSFG

R. massiliae MTU5 -------------------------------MRGFMSKDGNLDTSEFDPLANKEYTEEQKQKLEQEQKELLSQTTTPELEADDGFIVTSASSAQSTPSIS

R. rhipicephali ------------------------------------------DTSEFDPLANKEYTEEQKQKLEQEQKELLSQTTTPELEADDGFIVTSASSAQSTPSIS

R. aeschlimannii -------------------------------------------TSEFDPLANKEYTE------EQEQKELLSQTTTPELEADDGFIVTSASSTQSTPSIS

R. montanensis ------------------------------------------DTSEFDPLANKEYTEEQKQTLEQKQKEFLSQTTTPELEADDGFIVTSASSVQSTPSIS

R. slovaca ------------------------------------------DTSEFDPLANKEYTEEQKQTLEQEQKEFLSQTTTPALEADDGFIVTSASSAQSTPSMS

R. peacockii Rustic -----------------------------------MSKDGNLDTSEFDPLANKEYTEEQKQTLEQEQQEFLSQTITPALEADDGFIVTSASSAQSTPSMS

R. peacockii Rustic p19 ----------------------------------------------------------------------------------------------------

R. rickettsii Iowa -------------------------------MRGFMSKDGNLDTSEFDPLANKEYTEEQKQTLEQEQKEFLSQTTTPELEADDGFIVTSESSAQSTPSMS

R. rickettsii Sheila Smith -----------------------------------MSKDGNLDTSEFDPLANKEYTEEQKQTLEQEQKEFLSQTTTPELEADDGFIVTSESSAQSTPSMS

R. japonica -----------------------------------MSKDGNLNTSEFDPLANKEYTEEQKQTLEQEQKEFLSQTTTPELEADDGFIVTSASSAQSTPSTS

R. honei ------------------------------------------DTSEFDPLANKEYTEEQKQTLEQEQKEFLSQTTTPELEADDGFIVTSASSAQSTPSMS

R. conorii str. Malish 7 -------------------------------MRGFMSKDGNLDTSEFDPLANKEYTEEQKQTLEQEQKEFLSQTTTPALEADDGFIVTSASFAQSTPSMS

R. sibirica 246 -----------------------------------MSKDGNLDTSEFDPLANKEYTE------EQEQKEFLSHTTTPALEADDGFIVTSASFAQSTPSMS

R. africae ESF-5 -------------------------------MRGFMSKDGNLDTSEFDTLANKEYTAEQKQTLEQGQKEFLSQTTTPELEADDGFIVTSASFAQSTPSMS

R. parkeri ------------------------------------------DTSEFDPLANKEYTEEQKQTLEQEQKEFLSQTTTPALEADDGFIVTSASFAQSTPSMS

R. heilongjiangensis -----------------------------------MSKDGNLDTSEFDPLANKEYTEEQKQTLEQEQKEFLSQTTTPELEADDGFIVTSASSAQSTPSIS

Israeli tick typhus ------------------------------------------DTSEFDPLANKEYTEEQKQTLEQEQKEFLSQTTTPALEADDGFIVTSASFAQSTPSMS

R. sp. A-167 ------------------------------------------DTSEFDPLANKEYTEEQKQTLEQEQKEFLSQTTTPALEADDGFIVTSASFAQSTPSMS

Candidatus R. tasmanensis ----------------------------------LMSKDGNLDTSEFDPLANKEYTE------EQKQKEFLSQTTTPELEADDGFIVTSASSAQSTPSIS

Rickettsia sp. IG-1 ------------------------------------------DTSEFDPLANKEYTEEQKQTLEQEQKEFLSQTTTPELEADDGFIVTSASSAQSTPSMS

Candidatus R. barbariae --------------------------------------DGNLDTSEFDTLANKEYTAEQKQTLEQEQKEFLSQTITPELEADDGFIVTSASFAQSTPSMS

Rickettsia sp. S ------------------------------------------DTSEFDTLANKEYTAEQKQTLEQEQKEFLSQTITPALEADDGFIVTSASFAQSTPSMS

Candidatus R. goldwasserii ----------------------------------------------------------QKQTLEQEQK------------ADDGFIVTSASSAQSTPAIS

R. mongolotimonae ------------------------------------------DTSEFDPLANKEYTE------EQEQKEFLSQTTTPALEADDGFIVTSASFAQSTPSMS

R. sp. BJ-90 ------------------------------------------DTSEFDPLANKEYTEEQKQTEEQEQKEFLSHTTTPALEADDGFIVTSASFAQSTPSMS

Candidatus R. andeanae ------------------------------------------DTS--DPLANKEYTEEQKQKLEQEKKEFLSQTTTPELEADDGFIVTSASSAQFTPLIS

Rickettsia sp. Bar29 ------------------------------------------DTSEFDPLANKEYTENKEYTEEQKQKELLSQTTTPELEADDGFIV--TSSAQSTPSIS

Rickettsia sp. TwKM01 ------------------------------------------DISEFDPLANKEYTE------EQEQKELLSQTTTPELEADDGFIVTSASSAQSTLSIS

Rickettsia sp. IRS 4 ------------------------------------------DTSEFDPLANKEYTEEQKQKLEQEQKELLSQTTTPELEADDGFIVTSASSAQSTPSIS

R. raoultii ------------------------------------------DTSEFDPLANKEYTE------EQKQKEFLSQTTTPELEADDGFIVTSTSSAQSTPSIS

R. asiatica ------------------------------------------DTCEFDPL-NREFTEEEKQQQTKQEQELFSQTTPA--TADDGFMV--ASSAQSTPSIS

**[101-200 ]**

**[ | | | | | | | | | | ]**

R. bellii RML369-C ALAGGISGDGFI-DPITEAIRKEILEKQRDLIRNQLLKE----------NAENPDLKSNFESDEKFRDFLRTLNEDPNKKELYDKALENPELKKGLENIE

R. bellii OSU 85-389 ALAGGISGDGFI-DPITEAIRKEILEKQRDLIRNQLLKE----------NAENPDLKSNFESDEKFRDFLRTLNEDPNKKELYDKALENPELKKGLENIE

R. prowazekii Madrid E FLSNNRPHGDHKSDPITEAIRKEILEKQRD-----ILREYFVNTNPELAEQIAKE-----EDDRKFRAFL--SN--QDNYALINKAFEDTKTKKNLEKAE

R. prowazekii Rp22 FLSNNRPHGDHKSDPITEAIRKEILEKQRD-----ILREYFVNTNPELAEQIAKE-----EDDRKFRAFL--SN--QDNYALINKAFEDTKTKKNLEKAE

**R. typhi Wilmington VLSNSLPHGDQKSDPITEAIRKEILEKQRD-----ILREYLANTNPELAAQIAKE-----EDDKKFRAFL--SN--QDNYALINKAFEDPETKKNLEEVE**

R. australis TLSGTISTDDQISDPITKAVREIIIQQQKDEIAEQILKDLAALVDRDLAEQKRKEIEEEKEKDKKLSVFF--GN--PANREFIDNALEKPELKKKLESIE

R. australis TLSDTISHDGQTSDPITKAVRETIIQPQKDEIAEQILKDLAALVDRDLAEQKRKEIEEEKEKNKTLSAFF--GN--PANRELIDKALEKPELKKKLEAIE

R. felis URRWXCal2 SLSGGISSDSQTSDPITKAVRETIIQPQKDEIAEQILKDLAALADRDLAEQKRKEIEE--EKDKTLSAFF--GN--PANREFIDKALENPELKKKLESIE

R. canadensis McKiel ALSGSILADGQTLDPITEAIRKDILEKQRD-----ILREYFINTNPELAAQIAKE-----EEDKRLRAFL--SN--PDHRGLIDKAFEDSETKKKLEAAE

R. tamurae TLSGNISPDSQTSDPITKAVRETIIQPQKDNLIEQILKDLAALTDRDLAEQKRKEIEEEKEKDKTLSTFF--GN--PANREFIDKALEKPELKKKLESID

R. helvetica VLSGNISPDGQTSDPITKAVRETIIQPQKDNLIEQILKDLAVLADRNLAEQKRKEIEEEKEKDKTLSAFF--GN--PANREFIDRALENPELKKKLESIE

REIS_1 ALLDNISPDGQTSDPITKAVRETIIQPQKDNLIEQILKDLAAFADRDLAEQKRKEIEE--EKDKTLNAFF--GN--PANRKFIDKALENPELKKKLELIE

REIS_2 ----------------------------------------------------------------------------------------------------

REIS_3 MSGVGISLNSEGVDPITESIRKEILQKQREQL-------------RLYFSTRHHELEEHTLDDERFKKFLMHLN--NERRTLVSSALENEKVKAAMEEIE

R. massiliae MTU5 ALSGNISPDSQTSDPITKAIRETIIQPQKDNLIEQILKDLAALTDRDLAEQKRKEIEEEKEKDKTLSTFF--GN--PANREFIDKALENPELKKKLESIE

R. rhipicephali ALSGNISPDSQTSDPITKAVRETIIQPQKDNLIEQILKDLAALTDRDLAEQKRKEIEEEKEKDKTLSTFF--GN--PANREFIDKALENPELKKKLESIE

R. aeschlimannii ALSGNISPDSQTSDPITKAVRETIIQPQKDNLIEQILKDLAALTDRDLAEQKRKEIEE--EKDKTLSTFF--GN--PANREFIDKALENPELKKKLESIE

R. montanensis ALSGNISPDSQTSDPITKTVREIIIQPQKDNLIEQILKDLAALTDRDLAEQKRKEIEE--EKDKTLSTFF--GN--PANREFIDKALENPELKKKLESIE

R. slovaca ALSGNISPDSQTSDPITKAVRETIIQPQKDNLIEQILKDLAALTDRDLAEQKRKEIEEEKEKDKTLSTFF--GN--PANREFIDKALENPELKKKLESIE

R. peacockii Rustic ALSGNISPDSQTSDPITKAVRETIIQPQKDNLIEQILKDLAALTDRDLAEQKRKEIEE--EKDKTLSTFF--GN--PANREFIDKALENPELKKKLESIE

R. peacockii Rustic p19 ------------------------MQKQREQL-------------RLFLAVRNPELDEYTSDDERFTKFLMHLN--NEQRKFVNDALESEKVKAAMEQIE

R. rickettsii Iowa ALSGNISPDSQTSDPITKAVRETIIQPQKDNLIEQILKDLAALTDRDLAEQKRKEIEEEKEKDKTLSTFF--GN--PANREFIDKALEKPELKKKLESIE

R. rickettsii Sheila Smith ALSGNISPDSQTSDPITKAVRETIIQPQKDNLIEQILKDLAALTDRDLAEQKRKEIEEEKEKDKTLSTFF--GN--PANREFIDKALEKPELKKKLESIE

R. japonica ALSGNISPDSQTSDPITKAVRETIIQPQKDNLIEQILKDLAALTDHDLAEQKRKEIEEEKDKDKTLSTFF--GN--PANREFIDKALENPELKKKLESIE

R. honei ALSGNISPDSQTSDPITKAVRETIIQPQKDNLIEQILKDLAALTDRDLAEQKRKEIEEEKEKDKTLSTFF--GN--PANREFIDKALENPELKKKLESIE

R. conorii str. Malish 7 ALSGNISPDSQTSDPITKAVRETIIQPQKDNLIEQILKDLAALTDRDLAEQKRKEIEEEKEKDKTLSTFF--GN--PANREFIDKALENPELKKKLESIE

R. sibirica 246 ALSGNISPDSQTSDPITKAVRETIIQPQKDNLIEQILKDLAALTDRDLAEQKRKEIEEEKEKDKTLSTFF--GN--PANREFIDKALENPELKKKLESIE

R. africae ESF-5 ALSGNISPDSQTSDPITKAVRETIIQPQKDNLIEQILKDLAALTDRDLAEQKRKEIEEEKEKDKTLSTFF--GN--PANREFIDKALDNPELKKKLESIE

R. parkeri VLSGNISP-SQTSDPITKAVRETIIQPQKDNLIEQILKDLAALTDRDLAEQKRKEIEEEKEKDKTLSTFF--GN--PANREFIDKALENPELKKKLESIE

R. heilongjiangensis ALSGNISPDSQTSDPITKAVRETIIQPQKDNLIEQILKDLAALTDRDLAEQKRKEIEEEKDKDKTLSTFF--GN--PANREFIDKALENPELKKKLESIE

Israeli tick typhus ALSGNISPDSQTSDPITKAVRETIIQPQKDNLIEQILKDLAALTDRNLAEQKRKEIEEEKEKDKTLSTFL--GN--PANREFIDKALENPELKKKLESIE

R. sp. A-167 ALSGNISPDSQTSDPITKAVRKTIIQPQKDNLIEQILKDLAALTDRDLAEQKRKEIEEEKEKDKTLSTFL--GN--PANREFIDKALENPELKKKLESIE

Candidatus R. tasmanensis ALSGNISPASQTSDPITKAVRETIIQPQKDNLIEQILKDLAALTDRDLAEQKRKEIEE--EKDKTLSTFF--GN--PANREFIDKALENPELKQKLESIE

Rickettsia sp. IG-1 ALSGNISPDSQTSDPIIKAVRETIIQPQKDNLIEQILKDLAALTDRDLAEQKRKEIEE--EKDKTLSTFF--SN--PANREFIDKALENPELKKKLESIE

Candidatus R. barbariae TLSGNISPDSQTSDPITKAVRETIIQPQKDNLIEQILKDLAALTDRDLAEQKRKEIEEEKEKDKTLSTFF--GN--PANREFIDKALEKPELKKKLESID

Rickettsia sp. S ALSGNISPDSQTSDPITKAVRKTIIQPQKDNLIEQILKDLAALTDRDLAEQKRKEIEEEKEKDKTLSTFF--GN--PANREFIDKALENPELKKKLESIE

Candidatus R. goldwasserii ALSGNISPDSQTSDPITKAVRETIIQPQKDNLIEQILKDLAALTDRDLAEQKRKEIEE--EKDKTLSTFF--GN--PANRAFIDKALENPELKKKLESIE

R. mongolotimonae ALSGNISPDSQTSDPITKAVRETIIQPQKDNLIEQILKDLAAFTDRDLAEQKRKEIEEEKEKDKTLSTFF--GN--PANRELIDKALENPELKKKLESIE

R. sp. BJ-90 ALSGNISPDSQTSDPITKAVRETIIQPQKDNLIEQILKDLAALTDRDLAEQKRKEIEEEKEKDKTLSTFF--GN--PANREFIDKALENPELKKKLESIE

Candidatus R. andeanae ALSGNISPDSQTSAPITKAVRETIIQPQKDNLIEQILKDLAALTDRDLAEQKRKEIEEEKEKDKTLSTFF--GN--PANRELIDKALENPELKKKLESIE

Rickettsia sp. Bar29 ALSGNISPDSQTSDPITKAVRETIIQPQKDNLIEQILKDLAALTDRDLAEQKRKEIEEEKDKDKTLSTFF--GN--PANREFIDKALENPELKKKLESIE

Rickettsia sp. TwKM01 ALSGNISPDSQTSDPITKAVRETIIQPQKDNLIEQILKDLAALTDRDLAEQKRKEIEEEKEKDKTLSTFF--GN--PANREFIDKALENPELKKKLESIE

Rickettsia sp. IRS 4 ALSGNISPDSQTSDPITKAVRETIIQPQKDNLIEQILKDLAALTDRDLAEQKRKEIEEEKEKDKTLSTFF--GN--PANREFIDKALENPELKKKLESIE

R. raoultii ALSGNISPDIQTSDPITKAVGEIIIQPQKDNLIEQILKDLAALTDRDLAEQKRKEIEEEKEKDKTLSTFF--GN--PANREFIDKALENPELKKKLESIE

R. asiatica VLSGNISPDGQTSDPITKAVRETIIQPQKDNLIEQILKDLAVLADRNLAEQKRKEIEEEKEKDKTLSAFF--GN--PANREFIDRALENPELKKKLESIE

**[201-300 ]**

**[ | | | | | | | | | | ]**

R. bellii RML369-C IAGYKNVHASHSAEVYHENKIKEEQEELLRKYLNNDPAYSEEAKDQEKFRQFLANLNAGERQGLYDKALSDEQFKGQYENIRQEYANKYVGGFRSMQWEN

R. bellii OSU 85-389 IAGYKNVHASHSAEVYHENKIKEEQEELLRKYLNNDPAYSEEAKDQEKFRQFLANLNAGERQGLYDKALSDEQFKGQYENIRQEYANKYVGGFRSMQWEN

R. prowazekii Madrid E IVGYKNVLSTYSV------------------------------------------------------------------------ANGYQGGFQPVQWEN

R. prowazekii Rp22 IVGYKNVLSTYSV------------------------------------------------------------------------ANGYQGGFQPVQWEN

**R. typhi Wilmington IVGYRNILSTYSA------------------------------------------------------------------------ASGYPGGFQPVQWEN**

R. australis ITGYKNILLTYSA------------------------------------------------------------------------ANGYHGGFKPVQWEN

R. australis IAGLKNVFLTYIA------------------------------------------------------------------------ANGYSGGFKPVQWEN

R. felis URRWXCal2 IAGYKNVLSTYSA------------------------------------------------------------------------ANGYQGGFKPVQWEN

R. canadensis McKiel IQGYRNVFSIYS-------------------------------------------------------------------------AENYKGGFKPIQWKN

R. tamurae IAGYKNVHNTFSA------------------------------------------------------------------------ASGYPGGFKPVQWEH

R. helvetica IAGYKNVLSIYSA------------------------------------------------------------------------ANGYQGGFKPVQWEP

REIS_1 IAGYKNVHNTFSA------------------------------------------------------------------------ASGYPGGFKPVQWKN

REIS_2 ----------------------------------------------------------------------------------------------------

REIS_3 IVGYRNIHTSFS-------------------------------------------------------------------------AEHYQGGFKPMDWSG

R. massiliae MTU5 IAGYKNVHNTFSA------------------------------------------------------------------------ASGYPGGFKPVQWEN

R. rhipicephali IAGYKNVHNTFST------------------------------------------------------------------------ASGYPGGFKPVQWEN

R. aeschlimannii IAGYKNVHNTFSA------------------------------------------------------------------------ASGYPGGFKPVQWEN

R. montanensis IAGYKNVHNIFSA------------------------------------------------------------------------ASGYPGGFKPVQWEN

R. slovaca IAGYKNVHNTFSA------------------------------------------------------------------------ASGYPGGFKPVQWEN

R. peacockii Rustic IAGYKNVHNTFSA------------------------------------------------------------------------ASGYPGGFKPVQWEN

R. peacockii Rustic p19 LVGYRNIHTSFA-------------------------------------------------------------------------AENYPGGFKRMDWSG

R. rickettsii Iowa IAGYKNVHNTFSA------------------------------------------------------------------------ASGYPGGFKPVQWEN

R. rickettsii Sheila Smith IAGYKNVHNTFSA------------------------------------------------------------------------ASGYPGGFKPVQWEN

R. japonica IAGYKNVHNTFSA------------------------------------------------------------------------ASGYPGGFKPVQWEN

R. honei IAGYKNVHNTFSA------------------------------------------------------------------------ASGYPGGFKPVQWEN

R. conorii str. Malish 7 IAGYKNVHNTFSA------------------------------------------------------------------------ASGYPGGFKPVQWEN

R. sibirica 246 IAGYKNVHNTFSA------------------------------------------------------------------------ASGYPGGFKPVQWEN

R. africae ESF-5 IAGYKNVHNTFSA------------------------------------------------------------------------ASGYPGGFKPVQWEN

R. parkeri IAGYKNVHNTFSA------------------------------------------------------------------------ASGYPGGFKPVQWEN

R. heilongjiangensis IAGYKNVHNTFSA------------------------------------------------------------------------ASGYPGGFKPVQWEN

Israeli tick typhus IAGYKNVHNTFSA------------------------------------------------------------------------ASGYPGGFKPVQWEN

R. sp. A-167 IAGYKNVHNTFSA------------------------------------------------------------------------ASGYPGGFKPVQWEN

Candidatus R. tasmanensis IAGYKNVHNTFSA------------------------------------------------------------------------ASGYPGGFKPVQWEN

Rickettsia sp. IG-1 IAGYKNVHNTFSA------------------------------------------------------------------------ASGYPGGFKPVQWEN

Candidatus R. barbariae IAGYKNVHNTFSA------------------------------------------------------------------------ASGYPGGFKPVQWEH

Rickettsia sp. S IAGYKNVHNTFSA------------------------------------------------------------------------ASGYPGGFKPVQWEN

Candidatus R. goldwasserii IAGYKNVHNTFSA------------------------------------------------------------------------ASGYPGGFKPVQWEN

R. mongolotimonae IAGYKNVHNTFSA------------------------------------------------------------------------ASGYPGGFKPVQWEN

R. sp. BJ-90 IAGYKNVHNTFSA------------------------------------------------------------------------ASGYPGGFKPVQWEN

Candidatus R. andeanae IAGYKNVHNTFSA------------------------------------------------------------------------ASGYPGGFKPVQWEN

Rickettsia sp. Bar29 IAGYKNVHNTFSA------------------------------------------------------------------------ASGYPGGFKPVQWEN

Rickettsia sp. TwKM01 IAGYKNVHNTFSA------------------------------------------------------------------------TSGYPGGFKPVQWEN

Rickettsia sp. IRS 4 IAGYKNVHNTFSA------------------------------------------------------------------------ASGYPGGFKPVQWKN

R. raoultii IAGYTNVHNTFSA------------------------------------------------------------------------ASGYPGGFKPVQWEN

R. asiatica IAGYKNVLSIYSA------------------------------------------------------------------------ANGYQGGFKPVQWEP

**[301-400 ]**

**[ | | | | | | | | | | ]**

R. bellii RML369-C QVSAGDLRSTVIKNDAGEEICTLAEKTHKTAPMTVYKQDGTAVTVNSYRTIDFPIDLEGKS----------GTMHLSLVAQNKEGKS---NNALRFTAHY

R. bellii OSU 85-389 QVSAGDLRSTVIKNDAGEEICTLAEKTHKTAPMTVYKQDGTAVTVNSYRTIDFPIDLEGKS----------GTMHLSLVAQNKEGKS---NNALRFTAHY

R. prowazekii Madrid E QVSASDLRSTVVKNDEGEELCTLNETTVKTKDLIVAKQDGTQVQINSYREINFPIKLDKAQWVNAFIYGSIKSMHLSMVALKADGTKPAKDKAVYFTAHY

R. prowazekii Rp22 QVSASDLRSTVVKNDEGEELCTLNETTVKTKDLIVAKQDGTQVQINSYREINFPIKLDKAN----------GSMHLSMVALKADGTKPAKDKAVYFTAHY

**R. typhi Wilmington QVSASDLRSTVVKNDAGEELCTLNETTIKTNSLIVAKQDGTQVQINSYREIDFPIKLDKAD----------GSMHLSMVALKADGTKPAKDKAVYFTAHY**

R. australis QISASDLRATVVKNDAGDELCTLNETTVKTKPFTVAKKDGTQVQINSYRAIDFPIKLDKAD----------GSMHLSMVALKADGTKPSKDRAVYFTAHY

R. australis QISASDLRATVVKNDAGDELCTLNETTVKTKPFTVAKQDGTQVQINSYREIDFPIKLDKAD----------GSMHLSMVALKADGTKPSKDKAVYFTAHY

R. felis URRWXCal2 QISASDLRATVVRNDAGDELCTLNETTVKTKPFTVAKQDGTQVQINSYREIDFPIKLDKAD----------GSMHLSMVALKADGTKPSKDKAVYFTAHY

R. canadensis McKiel QVSASSLRTTVVENDAGDVLCTLNETTHNTKPFIVAKQDGTQVQVSSYREIDFPIKLDKAD----------GSMHLSMVALKADGTKPSKNKAVYFTAHY

R. tamurae HVSASDFRATVVKNDAGDELCTLNETTVKTKPFTLAKQDGTQVQISSYREIDFPIKLDKAD----------GSIHLSMVALKADGTKPSKDKAVYFTAHY

R. helvetica QISASGLRATVVKNDVGDELCTLNEITVKTKPFTVAKQDGTQVQVSSYREIDFPIKLDKAD----------GSMHLSMVALKADGTKPSKDKAVYFTAHY

REIS_1 QVSASDLRATVVKNDADDELCTLNETTVKTEPFTVAK-------------IDFPIKLDKAD----------GSMHLSMVALKADGTKPSKDKAVYFTAHY

REIS_2 ----------------------------------------------------------------------------------------------------

REIS_3 -VASINTRSQIIQNGVGDEICTLKEQTHTTSPLTISKQDGSTVIVNSYRTIDFPVELHEPA---------SGTMHLSLVARDKDGKAPSLERAVYFTAHY

R. massiliae MTU5 QVSASDLRATVVKNDAGDELCTLNETTVKTKPFTVGKQDGTQVQISSYREIDFPIKLDKAD----------GSMHLSMVALKADGTKPSKDKAIYFTAHY

R. rhipicephali QVSASDLRATVVKNDAGDELCTLNETTVKTKPFTVAKQDGTQVQISSYREIDFPIKLDKAD----------GSMHLSMVALKADGTKPSKDKAIYFTAHY

R. aeschlimannii QVSASDLRATVVKNDAGDELCTLNETTVKTRPFTVAKQDGTQVQISSYREIDFPIKLDKAD----------GSMHLSMVALKADGTKPSKDKAVYFTAHY

R. montanensis QVSASDLRATVVKNDAGDELCTLHETTVKTKPFTVAKQDGTQVQISSYREIDFPIKLDKAD----------GSMHLSIVALKADGTKPSKDKAVYFTAHY

R. slovaca HVSASDLRATVVKNDAGDELCTLNETTVKTKPFTLAKQDGTQVQISSYREIDFPIKLDKAD----------GSMHLSMVALKADGTKPSKDKAVYFTAHY

R. peacockii Rustic HVSASDLRATVVKNDAGDELCTLNETTVKTKPFTLAKQDGTQVQISSYREIDFPIKLDKAD----------GSMHLSMVALKADGTKPSKDKAVCFTAHY

R. peacockii Rustic p19 E-SELNTRSQIVKNDAGSEICTLKEQTHKTTPLTLSKQDESTTIVSSYRTIDFPVKLEKPA---------SGTMHLSLVARNKDGNAPLLEKAVYFTAHY

R. rickettsii Iowa HVSASDLRATVVKNDAGDELCTLNETTVKTKPFTLAKQDGTQVQISSYREIDFPIKLDQAD----------GSMHLSMVALKADGTKPSKDKAVYFTAHY

R. rickettsii Sheila Smith HVSASDLRATVVKNDAGDELCTLNETTVKTKPFTLAKQDGTQVQISSYREIDFPIKLDQAD----------GSMHLSMVALKADGTKPSKDKAVYFTAHY

R. japonica HVSASDLRATVVKNDAGDELCTLNETTVKTKPFTLAKQDGTQVQISSYREIDFPIKLDKAD----------GSMHLSMVALKADGTKPSKDKAVYFTAHY

R. honei HISASDLRATVVKNDAGDELCTLNETTVKTKPFTLAKQDGTQVQISSYREIDFPIKLDKAD----------GSMHLSMVALKADGTKPSKDKAVYFTAHY

R. conorii str. Malish 7 HVSANDLRATVVKNDAGDELCTLNETTVKTKPFTLAKQDGTQVQISSYREIDFPIKLDKAD----------GSMHLSMVALKADGTKPSKDKAVYFTAHY

R. sibirica 246 HVSASDLRATVVKNDAGDELCTLNETTVKTKPFTLAKQDGTQVQISSYREIDFPIKLDKAD----------GSMHLSMVALKADGTKPSKDKAVYFTAHY

R. africae ESF-5 HVSASDLRATVVKNDAGDELCTLNETTVKTKPFTLAKQDGTQVQISSYREIDFPIKLDKAD----------GSMHLSMVALKADGTKPSKDKAVYFTAHY

R. parkeri HVSASDLRATVVKNDAGDELCTLNETTVKTKPFTLAKQDGTQVQISSYREIDFPIKLDKAD----------GSMHLSMVALKADGTKPSKDKPVYFTAHY

R. heilongjiangensis HVSASDLRATVVKNDAGDELCTLNETTVKTKPFTLAKQDGTQVQISSYREIDFPIKLDKAD----------GSMHLSMVALKADGTKPSKDKAVYFTAHY

Israeli tick typhus HVSASDLRATVVKNDAGDELCTLNETTVKTKPFTLAKQDGTQVQISSYREIDFPIKLDKAD----------GSMHLSMVALKADGTKPSKDKAVYFTAHY

R. sp. A-167 HVSASDLRATVVKNDAGDELCTLNETTVKTKPFTLAKQDGTQVQISSYREIDFPIKLDKAD----------GSMHLSMVALKADGTKPSKDKAVYFTAHY

Candidatus R. tasmanensis QVSASDLRATVVKNDAGDELCTLNETTVKTKPFTLAKQDGTQVQISSYREIDFPIKLDKAD----------GSMHLSMVALKADGTKPSKDKAVYFTAHY

Rickettsia sp. IG-1 HVSASDLRATVVKNDAGDELCTLNETTVKTKPFTLAKQDGTQVQISSYREIDFPIKLDKAD----------GSMHLSMVALKADGTKPSKDKAVYFTAHY

Candidatus R. barbariae HVSASDFRATVVKNDAGDELCTLNETTVKTKPFTLAKQDGTQVQISSYREIDFPIKLDKAD----------GSIHLSMVALKADGTKPSKDKAVYFTAHY

Rickettsia sp. S HVNASDLRATVVKNDAGDELCTLNETTVKTKPFTLAKQDGTQVQISSYREIDFPIKLDKAD----------GSMHLSMVALKAAGTKPSKDKAVYFTAHY

Candidatus R. goldwasserii HVSASDLRATVVKNDAGDELCTLNETTVKTKPFTLTKQDGTQVQISSYREIDFPIKLDKAD----------GSMHLSMVALKADGTKPSKDKAVYFTAHY

R. mongolotimonae HVSASDLRATVVKNDAGDELCTLNETTVKTKPFTLAKQDGTQVQISSYREIDFPIKLDKAD----------GSMHLSMVALKADGTKPSKDKAVYFTAHY

R. sp. BJ-90 HVSASDLRATVVKNDAGDELCTLNETTVKTKPFTLAKQDGTQVQISSYREIDFPIKLDKAD----------GSMHLSMVALKADGTKPSKDKAVYFTAHY

Candidatus R. andeanae QVSASDLRATVVKNDAGDALCTLNETTVKTKPFTVAKQDGTQVQISSYREIDFPIKLDKAD----------GSMHLSMVALKADGTKPSKDKAVYFTAHY

Rickettsia sp. Bar29 QVSASDLRATVVKNDAGDELCTLNETTVKTKPFTVATQDGTQVQISSYREIDFPIKLDKAD----------GSMHLSMVALKADGTKPSKDKAIYFTAHY

Rickettsia sp. TwKM01 QVSASDLRATVVKNDAGDELCTLNETTVKTKPFTVAKQDGTQVQISSYREIDFPIKLDKAD----------GSMHLSMVALKADGTKPSKDKAIYFTAHY

Rickettsia sp. IRS 4 QVSASDLRATVVKNDAGDELCTLNETTVKTQPFTVAKQDGTQVQISSYREIDFPIKLDKAD----------GSMHLSMVALKADGTKPSKDKAVYFTAHY

R. raoultii QVSASDLRATVVKNDAGDELCTLNETTVKTKPFTVAKQDGTQVQISSYREIDFPIKLDKAD----------GSMHLSMVALKADGTKPSKDKAVYFTAHY

R. asiatica QISASGLRATVVKNDVGDELCTLNEITVKTKPFTVAKQDGTQIQVSSYREIDFPIKLDKAD----------GSMHLSMVALKADGTKPSKDKAVYFTTHY

**[401-500 ]**

**[ | | | | | | | | | | ]**

R. bellii RML369-C EADPHPDGTPKLKEVSSPQPIKFMGKDENAVGYIEHGGEIYTLPVTRGKYEAMMKEVAVNKGQGVDVSQTIEQDIYRVQGGEKSVSSQQIDPANEKPIET

R. bellii OSU 85-389 EADPHPDGTPKLKEVSSPQPIKFMGKDENAVGYIEHGGEIYTLPVTRGKYEAMMKEVAVNKGQGVDVSQTIEQDIYRVQGGEKSVSSQQIDPANEKPIET

R. prowazekii Madrid E EEG--PNGKPQLKEISSPQPLKFVGTGDDAVAYIEHGGEIYTLAVTRGKYKEMMKEVALNHGQSVALSQTIAEDLTHVQGPSH--ETHK---PIIIPNQE

R. prowazekii Rp22 EEG--PNGKPQLKEISSPQPLKFVGTGDDAVAYIEHGGEIYTLAVTRGKYKEMMKEVALNHGQSVALSQTIAEDLTHVQGPSH--ETHK---PIIIPNQE

**R. typhi Wilmington EEG--PNGKPQLKEISSPQPLKFVGTGDDAVAYIEHGGEIYTLAVTRGKYKAMMKEVALNNGQSVDLSQTIAEDLTKVQGPSH--VRHT---PIITPNQE**

R. australis EEG--PNGKPQLKEISSPQPLKFAGDGPDAVAYIEHGGEIYTLAVTRGKYKEMMKEVELHQGHSVDLSQIIAEDLTKVQGRSQ--ETLQ---PIITPNQE

R. australis EEG--PNGKPQLKEISSPKPLKFAGDGPDAVAYIEHGGEIYTLAVTRGKYKEMMKEVELHQGQSVDLSQTIAKDLTKVQGRSQ--ETLQ---PIITPNQE

R. felis URRWXCal2 EEG--PNGKPQLKEISSPKPLKFAGDGPDAVAYIEHGGEIYTLAVTRGKYKEMMREVELNQGQSVDLSQTIAEDLTKVQGRSQ--ETPQ---PIITPNQE

R. canadensis McKiel EEG--PNGKPLLKEISSPQPLKFAGDGPDAVAYIEHGGEIYTLAVTRGKYNEMMKEVELNKGQAIDLSQTIAEDLTKVQGRAQ--EIPQ---PIITPNQG

R. tamurae EEG--PNGKPQLKEISSPKPLKFAGTGDDAIAYIEHGGEIYTLAVTRGKYKEMMKEVELNQGQSVDLSQ--AEDLIIGQGQSKALETNE---PTINPNQG

R. helvetica EEG--PNGKPLLKEISSPQPLKFAGTGDDAVAYIEHGGEIYTLAVTRGKYKEMMKEVELNKGQSVDLSQTIAEDLTKVQGRSQ--ETPQ---PIITPNQE

REIS_1 EGG--PNGKPQLKEISSPKPLKFAGTGDDAIAYIEHGGEIYTLAVTRGKYKEMMKEVELNQGQSVDLSQ--AEDLIIGQGQSKALETNE---PTINPNQG

REIS_2 --------------MSSPKPIKFLGSNFDAIGYIEHGGGIYTLLVTRGKYEEMMKEVARNKGEAIDLSL-------------------------------

REIS_3 EATPKPNGVPKLKEVSSPKPIKFLGSNKESIGYIEHGGEIYTLPVTKGKYEEMMKEVAKNQGQAVDLSL-------------------------------

R. massiliae MTU5 EEG--PNGKPQLKEISSPKPLKFAGTGDDAIAYIEHGGEIYTLAVTRGKYKEMMKEVELNQGQSVDLSQ--AEDIIIGQGQSK----EQ---PLITPQQT

R. rhipicephali EEG--PNGKPQLKEISSPKPLKFAGTGDDAIAYIEHGGEIYTLAVTRGKYKEMMKEVELNQGQSVDLSQ--AEDIIIGQGQSK----EQ---PLITPQQT

R. aeschlimannii EEG--PNGKPQLKEISSPKPLKFAGTGDDAIAYIEQGGEIYTLAVTRGKYKEMMKEVELNQGQSVDLSQ--AEDIIIGQGQSK----EQ---PLITPQQT

R. montanensis EEG--PNGKPQLKEISSPKPLKFAGTGDDAIAYIEHGGEIYTLAVTRGKYKEMMKEVELNQGQSVDLSQ--AEDIIIGQGQSK----EE---PLITPQQT

R. slovaca EEG--PNGKPQLKEISSPKPLKFAGTGDDAIAYIEHGGEIYTLAVTRGKYKEMMKEVELNQGQSVDLSQ--AEDIIIGQGQSK----EQ---PLITPQQT

R. peacockii Rustic EEG--PNGKPQLKEISSPKPLKFAGTGDDAIAYIEHGGEIYTLAVTRGKYKEMMKEVELNQGQSVDLSQ--AEDIIIGQGQSK----EQ---PLITPQQT

R. peacockii Rustic p19 EATPKPNGVPKLKEVSSPQPIKFLGSGKEAVGYIEHGGEIYTLPVTRGKYEEMMREVARNRGQAVDLSL-------------------------------

R. rickettsii Iowa EEG--PNGKPQLKEISSPKPLKFAGTGDDAIAYIEHGGEIYTLAVTRGKYKEMMKEVELNQGQSVDLSQ--AEDIIIGQGQSK----EQ---PLITPQQT

R. rickettsii Sheila Smith EEG--PNGKPQLKEISSPKPLKFAGTGDDAIAYIEHGGEIYTLAVTRGKYKEMMKEVELNQGQSVDLSQ--AEDIIIGQGQSK----EQ---PLITPQQT

R. japonica EEG--PNGKPQLKEISSPKPLKFAGTGDDAIAYIEHGGEIYTLAVTRGKYKEMMKEVELNQGQSVDLSQ--AEDIIIGQGQSK----EQ---PLITPQQT

R. honei EEG--PNGKPQLKEISSPKPLKFAGTGDDAIAYIEHGGEIYTLAVTRGKYKEMMKEVELNQGQSVDLSQ--AEDIIIGQGQSK----EK---PLITPQQT

R. conorii str. Malish 7 EEG--PNGKPQLKEISSPKPLKFAGTGDDAIAYIEHGGEIYTLAVTRGKYKEMMKEVELNQGQSVDLSQ--AEDIIIGQGQSK----EQ---PLITPQQT

R. sibirica 246 EEG--PNGKPQLKEISSPKPLKFAGTGDDAIAYIEHGGEIYTLAVTRGKYKEMMKEVELNQGQSVDLSQ--AEDIIIGQGQSK----EQ---PLITPQQT

R. africae ESF-5 EEG--PNGKPQLKEISSPKPLKFAGTGDDAIAYIEHGGEIYTLAVTRGKYKEMMKEVELNQGQSVDLSQ--AEDIIIGQGQSK----EQ---PLITPQQT

R. parkeri EEG--PNGKPQLKEISSPKPLKFAGTGDDAIAYIEHGGEIYTLAVTRGKYKDMMKEVELNQGQSVDLSQ--AEDIIIGQGQSK----EQ---PLITPQQT

R. heilongjiangensis EEG--PNGKPQLKEISSPKPLKFAGTGDDAIAYIEHGGEIYTLAVTRGKYKEMMKEVELNQGQSVDLSQ--AEDIIIGQGQSK----EQ---PLITPQQT

Israeli tick typhus EEG--PNGKPQLKEISSPKPLKFAGTGDDAIAYIEHGGEIYTLAVTRGKYKEMMKEVELNQGQSVDLSQ--AEDIIIGQGQSK----EQ---PLITPQQT

R. sp. A-167 EEG--PNGKPQLKEISSPKPLKFAGTGDDAIAYIEHGGEIYTLAVTRGKYKEMMKEVELNQGQSVDLSQ--AEDIIIGQGQSK----EQ---PLITPQQT

Candidatus R. tasmanensis EEG--PNGKPQLKEISSPKPLKFAGTGDDAIAYIEHGGEIYTLAVTRGKYKEMMKEVELNQGQSVDLSQ--TEDIIIGQGQSK----KQ---PLITPQQT

Rickettsia sp. IG-1 KEG--PNGKPQLKEISSPKPLKFAGTGDDAIAYIEHGGEIYTLAVTRGKYKEMMKEVELNQGQSVDLSQ--AEDIIIGQGQSK----EQ---PLITPQQT

Candidatus R. barbariae EEG--PNGKPQLKEISSPKPLKFAGTGDDAIAYIEHGGEIYTLAVTRGKYKEMMKEVELNQGQSVDLSQ--AEDIIIGQGQSK----EQ---PLITPQQT

Rickettsia sp. S EEG--PNGKPQLKEISSPKPLKFAGTGDDAIAYIEHGGEIYTLAVTRGKYKEMMKEVELNQGQSVDLSQ--AEDIIIGQGQSK----EQ---PLITPQQT

Candidatus R. goldwasserii EEG--PNGKPQLKEISSPKPLKFAGTGDDAIAYIEHGGEIYTLAVTRGKYKEMMKEVELNQGQSVDLSQ--AEDIIIGQGQSK----EQ---PLITPQQT

R. mongolotimonae EEG--PNGKPQLKEISSPKPLKFAGTGDDAIAYIEHGGEIYTLAVTRGKYKEMMKEVELNQGQSVDLSQ--AEDIIIGQGQSK----EQ---PLITPQQT

R. sp. BJ-90 EEG--PNGKPQLKEISSPKPLKFAGTGDDAIAYIEHGGEIYTLAVTRGKYKEMMKEVELNQGQSVDLSQ--AEDIIIGQGQSK----EQ---PLITPQQT

Candidatus R. andeanae EEG--PNGKPQLKEISSPKPLKFAGTGDDAIAYTEHGGEIYTLAVTRGKYKEMMKEVELNQGQSIDLSQ--AEDIIIGQGQSK----EQ---PLITPQQT

Rickettsia sp. Bar29 EEG--PNGKPQLKEISSPKPLKFAGTGDDAIAYIEHGGEIYTLAVTRGKYNEMMKEVELNQGQSVDLSQ--AEDIIIGQGQSK----EQ---PLITPQQT

Rickettsia sp. TwKM01 EEG--PNGKPQLKEISSPKPLKFAGTGDDAIAYIEHGGEIYTLAVTRGKYKEMMKEVELNQGQSVDLSQ--AEDIIIGQGQSK----EQ---PPITPQQT

Rickettsia sp. IRS 4 EEG--PNGKPQLKEISSPKPLKFAGTGDDAIAYIEHGGEIYTLAVTRGKYKEMMKEVELNQGQSVDLSQ--AEDLIIGQRQSKALETNE---PTINPNQG

R. raoultii EEG--PNGKPQLKEISSPKPLKFAGTGDDAIAYIEHGGEIYTLAVTRGKYKEMMKELELNQGQSVDLSQ--AEDIIIGQGQSK----EQ---PLITPQQT

R. asiatica EEG--PNGKPLLKEISSPQPLKFAGTGDDAVAYIEHGGEIYTLAVTRGKYKEMMKEVELNKGQSVDLSQTIAEDLTKVQGRSQ--ETPQ---PIITPNQE

**[501-600 RRRRRRRRRRRRR]**

**[ | | | | | | | | | | ]**

R. bellii RML369-C KNTEIPEPQSGIASNQIPPVTPLSSGQKPVVSQMPQPQQAEILQP-QPQGIVDAAVGLSQAMQNLLDQLNKDLKNE---NEPAGLIGEVAEKILGEKEAN

R. bellii OSU 85-389 KNTEIPEPQSGIASNQIPPVTPLSSGQKPVVSQMPQPQQSEILQP-QPQGIVDAAVGLSQAMQNLLDQLNKDLKNE---NEPAGLIGEVAEKILGEKEAN

R. prowazekii Madrid E LESSIEQHT----SQQVPPITTFNKSLQPKISQIHQLQPQQAQSSGIPNPVLNAANALSTSMQDLLNNINSYLTKNQDINKQSDLIKEAAIAILNNKKSD

R. prowazekii Rp22 LESSIEQHT----SQQVPPITTFNKSLQPKISQIHQLQPQQAQSSGIPNPVLNAANALSTSMQDLLNNINSYLTKNQDINKQSDLIKEAAIAILNNKKSD

**R. typhi Wilmington LELSIETHS----NQQVPPITTFNKPLQPEISQTHQLQPQQAQSSGIPNLVLNAAHALSISMQDLLHNINASLTQKQDINKQSDLIKEAANTILNNKKSD**

R. australis LKSSIETPT----TTQVPPITPASQPLHTETSQM--PQSQQVNPN-----LFNAATALSCSMQDLLNYVNAGLTKEKDGNTQIDLINEAATAILNNEK--

R. australis LKSSIETPT----TTQVPPITPDSQPLQTETAQM--PQSQQVNPN-----LLNAATALSGSMQDLLNYVNAGLTKEKDGNTQIDLINAAATAILNNEK--

R. felis URRWXCal2 LKSSIETPT----TTQVPPITPANQPLQPETSQM--PQPQQVNPN-----LLNAATALSTSMQDLLNYVNAGLTKEKDGNKQIDLINEAATAILNNEKSD

R. canadensis McKiel LKSSIAKPT------------------ATQTTQM--LQPQQEESSGIPNLVLNAVTVLSGSMHDLLNYVNAGLTKEIEGNKQIDLIKEACAAILNNEKSD

R. tamurae LQSSIETPT------------------STQTTQM--PQSQQVNPN-----LLNAATALSGSMQDLLNYVNAGLTKETDGNKQIDLIKEAATAILNNEKSD

R. helvetica LKSSIETPT----ATQMPSITPANQPLQPETSQVQPPQPQQAQSSGIPNPVLNAAIALSGSMQDLLNYVNTGLTKETDGNKQIDLIKEAATAILNNEKSD

REIS_1 LQSSIETP------------TPT------QTTQM--LQSQQVNPN-----LLNTATALSGSMQDLLNYVNAGLTKETDGNKQIDLIKEAATVILNNEKSD

REIS_2 ---------------------------------------------------------------------------------------------------E

REIS_3 ---------------------------------------------------------------------------------------------------E

R. massiliae MTU5 ASSSVESPQY---KQQVPPITPTNQPLQPETSQM--PQSQQVNPN-----LLNAATALSGSMQDLLNYVNAGLTKEIDSNKQIDLIKEAATAILYNEKSD

R. rhipicephali ASSSVESPQY---KQQVPPITPTNQPLQPETSQM--PQSQQVNPN-----LLNAATALSGSMQDLLNYVNAGLTKEIDSNKQIDLIKEAATAILHNAKSD

R. aeschlimannii ASSSVELPQY---KQQVPPITPTNQPLQPETSQM--SQSQQVNPN-----LLNAATTLSGSMQDLLNYVNAGLTKEIDTNKQIDLIKEAATAILNNEKSD

R. montanensis ASSSVEPPQY---KQQVPPITPTNQPLQPGTSQM--PQSQQVNLN-----LLNAATTLSGSMQDLLNYVNAGLTKEIDSNKQIYLIKEAATAILNNEKSD

R. slovaca TSSSVEPPQY---KQQVPPITPTNQPLQAETSQM--PQSQQVNPN-----LLNTATALSGSMQDLLNYVNAGLTKEIDSNKQIDLIKEAAKAILNNEKSD

R. peacockii Rustic TSSSVEPPQY---KQQVPPITPTNQPLQPETSQM--PQSQQVNPN-----LLNAATALSGSMQDLLNYVNAGLTKEIDSNKQIDLIKEAATAILNNEKSD

R. peacockii Rustic p19 ---------------------------------------------------------------------------------------------------E

R. rickettsii Iowa TSSSVEPPQY---KQQVPPITPTNQPLQPETSQM--PQSQHVNPN-----LLNAATALSGSMQYLLNYVNAGLTKEIDSNQQIDLIKEAATVILNNEKSD

R. rickettsii Sheila Smith TSSSVEPPQY---KQQVPPITPTNQPLQPETSQM--PQSQHVNPN-----LLNAATALSGSMQYLLNYVNAGLTKEIDSNQQIDLIKEAATVILNNEKSD

R. japonica TSSSVEPPQY---KQQVPPITPTNQPLQPETSQM--PQSQQVNPN-----LLNAATALSGSMQDLLNYVNAGLTKE------IDLIKEAATAILNDKKSD

R. honei TSSSVEPPQY---KQQVPPITPTNQPLQPETSQM--PQSQQVNPN-----LLNAATALSGNMQDLLNYVNTGLAKEIDSNKQIDLIKEAATAILNNEKSD

R. conorii str. Malish 7 TSSSVEPPQY---KQQVPPITPTNQPLQPETSQM--PQSQQVNPN-----LLNTATALSGSMQDLLNYVNAGLTKAIDSNKQIDLIKEAATAILNNEKSD

R. sibirica 246 TSSSVEPPQY---KQQVPPITPTNQPLQPETSQM--LQSQQVNPN-----LLNTATALSGSMQDLLNYVNAGLTKEIDSNKQIDLIKEAATAILNNEKSD

R. africae ESF-5 TSSSVEPPQY---KQQVPPITPTNQPLQPETSQM--QQSQQVTPN-----LLNTATALSGSMQDLLNYVNAGLTKEIDSNKQIDLIKEAATAILNNEKSD

R. parkeri TSSSVEPPQH---KQQVPPITPTNQPLQPETSQM--PQSQQVNPN-----LLNTATALSGSMQDLLNYVNAGLTKEIDSNKQIDLIKEAATAILNNEKSD

R. heilongjiangensis TSSSVEPPQY---KQQVPPITPTNQPLQPETSQM--PQSQQVNPN-----LLKAATALSGSMQDLLNYVNAGLTKE------IDLIKEAATAILNDKKSD

Israeli tick typhus TSSSVEPPQY---KQQVPPITPTNQPLQPETSQM--PQSQQVNPN-----LLNTATALSGSMQDLLNYVNAGLTKEIDSNKQIDLIKEAATAILNNEKSD

R. sp. A-167 TSSSVEPPQY---KQQVPPITPTNQPLQPETSQM--PQSQQVNPN-----LLNTATALSGSMQDLLNYVNAGLTKEIDSNKQIDLIKEAATAILNNEKSD

Candidatus R. tasmanensis ASSSVEPPQY---KQQVPPITPTNQPLQSETSQM--PQSQQVLPN-----LLNTATALSGSMQDLLNYVNAGLTKE------IDLIKEAATAILNNAKSD

Rickettsia sp. IG-1 TSSSVEPPQY---KQQVPPITPTNQPLQPETSQI--QQSQQVNPN-----LLNAATALSGSMQDLLNYVNAGLTKEIDSNKQIDLIKEAATAILNNEKSD

Candidatus R. barbariae ASSSVEPPQY---KQQVPPITPTNQPLQPETSQM--QQSQQVTPN-----LLNTATALSGSMQDLLNYVNAGLTKEIDSNKQIDLIKETATAILNNEKSD

Rickettsia sp. S TSSSVEPPQY---KQQVPPITPTNQPLQPETSQM--QQSQQVNPN-----LLNTAMALSGSMQDLLNYVNAGLTKEIDSNKQIDLIKEAATAILNNEKSD

Candidatus R. goldwasserii TSSSVEPPQY---KQQVPPITPTNQPLQPETSQM--PQSQQVNPN-----LLNAATALSGSMQDLLNYVNAGLTKEIDSNKPIDLIKEAATAILNNEQSD

R. mongolotimonae TSSSVEPPQY---KQQVPPITPTNQPLQPETSQM--PQSQQVNPN-----LLNTATTLSGSMQDLLNDVNAGLTKEIDSNKQIDLIKEAATAILNNEKSD

R. sp. BJ-90 TSSSVEPPQY---KQQVPPITPTNQPLQPETSQM--LQSQQVNPN-----LLNTATALSGSMQDLLNYVNAGLTKEIDSNKQIDLIKEAATAILNNEKSD

Candidatus R. andeanae ASSSVEPPQY---KQQVPPITPTNQPLQPETSQM--PQSQQVNPN-----LLNAATALSGSMQDLLNY------------KQIDLIKEAATAILNNEKND

Rickettsia sp. Bar29 ASSSVESPQY---KQQVPPITPTNQPLQPETSQM--PQSQQVNPN-----LLNAATVLSGSMQDLLNYVNAGLTKEIDSNKQIDLIKEAATAILHNEKSD

Rickettsia sp. TwKM01 ASSSVESPQY---KQQVPPITPTNQPLQPETSQM--PQSQQVNPN-----LLNAATALSGSMQDLLNYVNAGLTKE------IDLIKEAATAILHNEKSD

Rickettsia sp. IRS 4 LQSSIETP------------TPT------QTTQM--PQSQQVNPN-----LLNAATALSGSMQDLLNYVNAGLTKETDGNKQIDLIKEAATVILNNEKSD

R. raoultii ASSSVEPPQH---KQQVPPITPTNQPLQTKASQM--PQSQQVNPN-----LLNAATVLSGSMQDLLHDVNAGLTKEIDSNKQIDLIKEAATAILNNEKSD

R. asiatica LKSSIETPT----ATQMPSITPANQPLQPETSQVQPPQPQQAQSSGIPNPVLNAAIALSGSMQDLLNYVNTGLTKETDGNKQIDLIKEAATAILNNEKSD

**[601-700 RRRRRRRRRRRRRRRRRRRRRRRRRRRRRRRRRRRRRRRRRRRRRRRRRRRRRRRRRRRRRRRRRRRRRRRRRRRRRRRRRRRRRRRRRRRRRRRRRRRR]**

**[ | | | | | | | | | | ]**

R. bellii RML369-C VDQKQAGINTLAENVLSNKELPEEVRVEGIGKILDTINNDNVLSEPEKAKLLGGITSVALDQDSLSQAAKKQIVGKITDSALELNTSDVRLQAINGITDA

R. bellii OSU 85-389 VDQKQAGINTLAENVLSNKELPEEVRVEGIGKILDTINNDNVLSEPEKAKLLGGITSVALDQDSLSQAAKKQIVGKITDSALELNTSDVRLQAINGITDA

R. prowazekii Madrid E FAEKQYNIIDLAKNIFSNKDIIADAKVNVVNTLLETIQNDQNTLDIKKSKILEDTVAITLNSENIELKQKQQILEKVVDIGLSIKDDISRVVAVDSIMDT

R. prowazekii Rp22 FAEKQYNIIDLAKNIFSNKDIIADAKVNVVNTLLETIQNDQNTLDIKKSKILEDTVAITLNSENIELKQKQQILEKVVDIGLSIKDDISRVVAVDSIMDT

**R. typhi Wilmington FAEKQYNIIALTENTLSNKDIIADAKVNVVSALLETIQNDQNTLDIQKSKILEATVAITLNSENIELKQKQQILEKVVDVSLSIKDDISRAVAIDSITDA**

R. australis --EKQANFITLTKNMVNNNALTPDTKVARVNAVLETIKNNQDTPDIEKSKMLEATVAITLNSENLTPKQKQQMLEKAVDVDLSFKDDTSRAVAIDGITGA

R. australis --EKQANIIVLTENTVNNNALTPDTKVAGVNAVLENIKNNQNTPDLEKSKMLEATVAIALNSENLAPKQKQQMLEKAVDVGLNLKDDTSRAVAIDGITDT

R. felis URRWXCal2 IAEKQANIIALTENTVNNNDLTPDTKVAGVNAVLETIKNDQNTPDLEKSKMLEATVAIALNSENLEPKQKQQMLEKAVDVGLSLKDDASRVTAIDGITDA

R. canadensis McKiel LVAKQAHVITLAENMVNNKDLVPDVKVAGVCSILEIIKSDNALSNPEKSKMLEAIVAIALNSENLESKYKLHIVEKAGDLGLGLNDANARANTIDGITNA

R. tamurae IAEKQANIIALTENTVNNNDLTPDTKVAGVNAVLETIKNDQNTPDLEKSKMLEATVAIALNSENLEPKQKQQMLEKAVDVGLSLKDDASRAAAIDGITDA

R. helvetica IAEKQANIIALAENTVNNKDLTPDAKVAGVNAILETIKNDQNTPDLEKSKMLEATVAIALNSENLEPKQKQQMLEKAVDVGLSLKDDASRAAAIDGITDA

REIS_1 IAEKQANIIALTENTVNNNALTPDTKVAGVNAVLETIKNDQNTPDLEKSKMLEATVAIALNSENLEPKQKQQMLEKAVDVGLSLKDDASRAAAIDGITDA

REIS_2 VEDRAQDLVS------------------------------------------------------------------------------------------

REIS_3 VEPQAQDLI-------------------------------------------------------------------------------------------

R. massiliae MTU5 IAEKQTNIIALAENTVNNQNLTPNAKVAGVHAVLETIKNDQNTPDLEKSKMLEATVAIALNSENLEPKQKQQMLEKTVDVGLSLKDDASRAAAIDGITDA

R. rhipicephali IAEKQTNIIALAENTVNNQNLTPDAKVAGVNAVLETIKNDQNTPDLEKSKMLEATVAITLNSENLEPKQKEQMLEKTVDVGLSLKDDASRAAAIDGITDA

R. aeschlimannii IAEKQANIIALAENTVNNKNLKPDAKVTGVNAVLKTIKNDQNTPDLEKSKMLEATVAIVLNSENLEPKQKQQMLEKALDVGLSLKDDASRAAAIDDITDA

R. montanensis IAEKQANIIALAENTVNNKNLKPDAKVTGVNAVLETIKNDQNTPNLEKSKMLEATVAIVLNSENLEPKQKQQMLEKAVDVGLSLKDDASRAAAIDGIKDV

R. slovaca IAEKQANIIALAENTVNNKNLKPDAKVAGVNAVLETIKNDQNTPDLEKSKMLEATVAIVLNSENLEPKQKQQMLEKAVDVGLSLKDDASRAAAIDGIKDV

R. peacockii Rustic IAEKQANIIALAENTVNNKNLKPDAKVAGVNAVLETIKNDQNTPDLEKSKMLEATVAIVLNSENLEPKQKQQMLEKAVDVGLSLKDDASRAAAIDGIKDV

R. peacockii Rustic p19 VERKAQDLIR------------------------------------------------------------------------------------------

R. rickettsii Iowa IAEKQANIIAFAENTVNNKNLKPDAKVAGVNAVLETIKNDQNTPDLEKSKMLEATVAIVLNSENLEPKQKQQVLDKAVDVGLSLKDDASRAAAIDGIKDV

R. rickettsii Sheila Smith IAEKQANIIAFAENTVNNKNLKPDAKVAGVNAVLETIKNDQNTPDLEKSKMLEATVAIVLNSENLEPKQKQQVLDKAVDVGLSLKDDASRAAAIDGIKDV

R. japonica IAEKQANIIALAENTVNNKNLTPDAKVAGVNAVLETIKNDQNTPDLEKSKMLEATVAIALNSENLEPKQKQQILEKAVDVGLSLKDDASRAAAIDGITDA

R. honei IAEKQANIIALAENTVNNKNLKPDAKVTGVNAVLETIKNDQNTPDLEKSKMLEATVAIVLNSENLEPKQKQQMLEKAVGVGLSLKDDASRAAAIDGIKDV

R. conorii str. Malish 7 IAEKQANIIALAENTVNNKNLKPDAKVTGVNAVLETIKNDQNTPNLEKSKMLEATVAIVLNSENLEPKQKQQMLEKAVDVGLSLKDDASRAAAIDGIKDV

R. sibirica 246 IAEKQANIIALAENTVNNKNLKPDAKVAGVNAVLETIKNDQNTPNLEKSKMLEATVAIILNSENLEPNQKQQMLEKAVDVGLSLKDDASRAVTIDGIKDV

R. africae ESF-5 IAEKQANIIALAENTVNNKNLKPDAKVAGVNAILETIKNDQNTPNLEKSKMLEATVAIVLNSENLESKQKQQMLEKAVDVGLSLKDDASRAATIDGIKDV

R. parkeri IVEKQANIIALAENTVNNKNLKPDAKVAGVNAVLEIIKNDQNTPNLEKSKMLEATVAIVLNSENLEPKQKQQMLEKAVDVGLSLKDDASRAATIDGIKDV

R. heilongjiangensis IAEKQANIIALAENTVNNKNLTPDTKVAGVNAVLETIKNDQNTPDLEKSKMLEATVAIALNSENLEPKQKQQILEKAVDVGLSLKDDASRAAAVDGITDA

Israeli tick typhus IAEKQANIIALAENTVNNKNLKPDAKVAGVNAVLETIKNDQNTPNLEKSKMLEATVAIVLNSENLEPKQKQQMLEKAVDVGLSLKDDASRAAAIDGIKDV

R. sp. A-167 IAEKQANIIALAENTVNNKNLKPDAKVAGVNAVLETIKNDQNTPNLEKSKMLEATVAIVLNSENLEPKQKQQMLEKAVDVGLSLKDDASRAAAIDGIKDV

Candidatus R. tasmanensis IAEKQANIIALAENTVNNKNLTPDAKVAGVNAVLETIKNDQNTPDLEKSKMLEATVAIALNSENLEPKQKQQMLEKAVDVGLSLKDDASRAAAIDGITDA

Rickettsia sp. IG-1 IAEKQANIIALAENTVNNKNLKPDAKVAGIN--LETIKNDQSTLDLEKSKMLEATVAIVLNSENLEPKQKQQMLEKAVDVGLSLKDDASRAAAIDGIKDV

Candidatus R. barbariae IAEKQANISALAENTVNNKNLKPDAKVAGVNAVLETIKNDQNTPNLEKSKMLEATVAIVLNSENLEPKQKQQMLEKAVDVGLSLKDDASRAATIDGIKDV

Rickettsia sp. S IAEKQANIIALAENTVNNKNLKPDAKVAGVNAVLETIKSDQNTPNLEKSKMLEATVAIVLNSENLEPKQKQQMLEKAVDVGLSLKDDASRAATIDGIKDV

Candidatus R. goldwasserii IAEKQANIIALAENTVNNKNLKPDAKVAGVNAVLETIKNDQNTPDLEKSKMLEATVAIVLNSENLEPKQKQQMLEKAVDVGLSIKDDASRAAAIEGIKDV

R. mongolotimonae IAEKQANIIALAENMVNNKNLKPDAKVAGVNAVLETIKNDQNTPNLEKSKMLEATVAIILNSENLEPKQKQQMLEKAVDVGLSLKDDASRAVTIDGIKDV

R. sp. BJ-90 IAEKQANIIALAENTVNNKNLKPDAKVAGVNAVLETIKNDQNTPNLEKSKMLEATVAIILNSENLEPNQKQQMLEKAVDVGLSLKDDASRAVTIDGIKDV

Candidatus R. andeanae IAEKQTNIIALAENTVNNKNLTPDAKVAGVNAVLETIKNDQNTPDLEKSKMLEATVAIALNSENLEPKQKQQMLEKAVDVGLSLKDDASRAAVIDGITDA

Rickettsia sp. Bar29 IAEKQTNIIALAENTVNNQNLTPNAKVAGVHAVLETIKNDQNTPDLEKSKMLEATVAIALNSENLEPKQKQQMLEKTVDVGLSLKDDASRAAAIDGITDA

Rickettsia sp. TwKM01 IAEKQTNIIALAENTVNNQNLTPDAKVAGVNAVLETIKNDQNTPDLEKSKMLEATVAIALNSENLEPKQKQQMLEKTVDVGLSLKDDASRAAEIDGVTDA

Rickettsia sp. IRS 4 IAEKQANIIALTENTVNNNDLTPDTKVAGVNAVLETIKNDQNTPDLEKSKMLEATVAIALNSENLEPKQKQQMLEKAVDVGLSLKDDASRAAAIDGITDA

R. raoultii IAEKQTNIIALAENTVNNKNLTPDAKVAGVNAVLETIKNDQNTPDLEKSKMLEATVAIALNSENLEPQQKQQMLEKAVDVGLSLKDDASRAAAIDGITDA

R. asiatica IAEKQANIIALAENTVNNKDLTPDAKVAGVNAILETIKNDQNTPDLEKSKMLEATVAIALNSENLEPKQKQQMLEKAVDVGLSLKDDASRAAAIDGITDA

**[701-800 RRRRRRRRRRRRRRRRRRRRRRRRRRRRRRRRRRRRRRRRRRRRRRRR RRRRRRR]**

**[ | | | | | | | | | | ]**

R. bellii RML369-C VLDSNLNKDEKGEIFEVISNVI-EASKHDAPEKSQLKSDVL-DKAREAGILSPEQQQLIQQNLDKIKEKQAA--EETIKKVNGIL--YDP-LSDAVKKTD

R. bellii OSU 85-389 VLDSNLNKDEKGEIFEVISNVI-EASKHDAPEKSQLKSDVL-DKAREAGILSPEQQQLIQQNLDKIKEKQAA--EETIKKVNGI---FDP-LSDAVKKTD

R. prowazekii Madrid E VIKSNIANEDKEKIFITVFDQI-NSYEFSNVAKQKLLDSIL-KKTAETQVLSPEQQQLMNQNLDNITTEHTK--RDTIEKVNNIL--LEP-LSNTALKTT

R. prowazekii Rp22 VIKSNIANEDKEKIFITVFDQI-NSYEFSNVAKQKLLDSIL-KKTAETQVLSPEQQQLMNQNLDNITTEHTK--RDTIEKVNNIL--LEP-LSNTALKTT

**R. typhi Wilmington VIKSNIANKDKEQIFMTIFDQV-NSYEFSNITKQKLLGSML-KKAVETKVISPEQQQLIHQNLDKITTEHTK--RDTIEKVNNIL--LDP-FSNTVLKTT**

R. australis VIKSNLSTKDKGTMLIAVGDKV-NASELSNAEKQQLLGSVL-KKGVETKILSPEQQQLMQQNLDKITAEQTK--NDNITEVQGIL--ANP-AFNTIAKTA

R. australis VIKSNLSTKDKGTMLIAVGDKV-NASELSNAEKQQLLGSVL-KKGVETQVLSPEQQQLMQQHLDKITAEQTK--NAKITEVQGIL--ANP-AFNTIAKTE

R. felis URRWXCal2 VIKSNLSTEDKGTMLIAVGDKV-NASELSNAEKQKLLGSVL-KKGVEAQVLSPEQQQLMQQNLDKITAEQTK--NAQITEVQGIL--ANP-AFNTIAKTE

R. canadensis McKiel VIKSNLSTEDKETMLMAVGDKV-NTAQLSNTEKQKLLGSVL-KIGVEAAVLSEEQQQLMRQNLDKITVEQEK--ESKVAAVLSVL--VDP-AFNTIAKTK

R. tamurae AIKSNLSTEDKGTMLIAVGDKI-NASELSNAEKQKLLGSVL-KKGVEVQVLSPEQQQLMQQNLDKITAEQTK--KDTIKKVNDIL--FDP-LSNTELKTT

R. helvetica VIKSNLSTEDKGTMLIAVDDKV-NASELSNAEKQKLLGSVL-KKGVEAQVLSPAQQQLMQQHLDKITAEQIK--KDTIKKVNDIL--FDP-LSNTELKTT

REIS_1 AIKSNLSTEDKGTMLIAVGDKV-NASELSNAEKQKLLGSVL-KKGVEAQVLSPEQQQLMQQNLDKIMAEQTK--KDTIKKVNDIL--FDP-LSNTELKTT

REIS_2 -----------------------HIGKVSGAKIERVTGEEEEKSGLTSKMAR-------INNLDGSRAAQIKYHEDQITKYKNILKELEPALQSRDQERE

REIS_3 -----------------------SSTKQQGAEITRVTGEGLAKSGLAEKLDEEAINNATDSKLEQIKHHEAQ--IARYKKILEEH--IEPALECRDKERE

R. massiliae MTU5 VIKSNLSTEDKGT---AVGDKV-NVSELSNAEKQKLLGSVL-KKGVEAQVLSPAQQQLMQQNLDKITAEQTK--KDTIKKVNDIL--FDP-LSNTELKTT

R. rhipicephali VIKSNLSTEDKGTMLIAVGDKV-NVSELSNAEKQKLLGSVL-KKGVEAQVLSPAQQQLMQQNLDKITAEQTK--KDTIKKVNDIL--FDP-LSSTELKTT

R. aeschlimannii VIKSSLSTEDKGTMLIAVGDKV-NVSELSNAEKQKLLGSVL-KKGVEAQVLSPAQQQLMQQNLDKITAEQTK--KDTIKKVNDIL--FDP-LSNTELKTT

R. montanensis VIKSNLSPEDK--MLIAVGDKV-NVSELSNAEKQKLLGSVL-KKGVEAQVLSPAQQQLMQQHLDKITAEQTK--KDTIKKVNDIL--FDP-LSNTELKTT

R. slovaca VIKSNLSPEDKGTMLIAVGDKV-NVSELSNAEKQKLLGSVLKKKGVEAQVLSPAQQQLMQQHLYKITAEQTK--KDTIKKVNDIL--FDP-LSNTELKTT

R. peacockii Rustic VIKSNLSNA----MLIAVGDKV-NVSELSNAEKQKLLGSVL-KKGVEAQVLSPAQQQLMQQHLDKIMAEQTK--KDTIKKVNDIL--FDP-LSNTELKTT

R. peacockii Rustic p19 ----------------------------------------------------------------------------------------------------

R. rickettsii Iowa VIKSNLSTV----MLIAVGDKV-NVSELSNAEKQKLLGSVL-KKGVEAQVLSPAQQQLMQQHLDKIMAEQTK--KDTIKKVNDIL--FDP-LSNTELKTT

R. rickettsii Sheila Smith VIKSNLSTV----MLIAVGDKV-NVSELSNAEKQKLLGSVL-KKGVEAQVLSPAQQQLMQQHLDKIMAEQTK--KDTIKKVNDIL--FDP-LSNTELKTT

R. japonica VIKSNLSTEDKGTMFIAVGDKV-NVSELSNAEKQKLLGSVL-KKGVEAQVLSPAQQQLMQQNLDKITAEQTK--KDTIKKVNDIL--FDP-LSNTELKTT

R. honei VIKSNLSTEDKWTMLITVGDKV-NVSELSNAEKQKLLGSVL-KKGVEAQVLSPAQQQLIQQNLDKITAEQTK--KDTIKKVNNIL--FDP-LSNTELKTT

R. conorii str. Malish 7 VIKSNLSPEDK--MLIAVGDKV-NVSELSNAEKQKLLGSVL-KKGVEAQVLSPAQQQLMQQHLYKIMAEQTK--KDTIKKVNDIL--FDP-LSNTELKTT

R. sibirica 246 VIKTNL---------------------LSNTEKQKLLGSVL-KKGVEAQVLSPAQQQLMQQHLDKITAEQIK--KDTIKKVNDIL--FDP-LSNTELKTT

R. africae ESF-5 VIKSNLSTEDKGTMLIAVGDKV-NVSELSNAEKQKLLGSVL-KKDVEAQVLSPAQQQLMQQHLDKITAEQTK--KDTIKKVNDIL--FDP-LSNTELKTT

R. parkeri VIKSNLYTEDKGTMLIAVGDKV-NVSELSNAEKQKLLGSVL-KKGVEAQVLSPAQQQLMQQHLDKITAEQTK--KDTIKKVNDIL--FDP-LSNTELKTT

R. heilongjiangensis VIKSNLSTEDKGTMLIAVGDKV-NVSELSNAEKQKLLGSVL-KKGVEAQVLSPAQQQLMQQNLDKITVEQTK--KDTIKKVNDIL--FDP-LSNTELKTT

Israeli tick typhus VIKSNLSTEDK--MLIAVGDKV-NVSELSNAEKQKLLGSVL-KKGVEAQVLSLAQQQLMQQHLDKIMAEQTK--KDIIKKVNDIL--FDP-LSNTELKTT

R. sp. A-167 VIKSNLSTEDK--MLIAVGDKV-NVSELSNAEKQKLLGSVL-KKGVEAQVLSLAQQQLMQQHLDKIMAEQTK--KDIIKKVNDIL--FDP-LSNTELKTT

Candidatus R. tasmanensis VIKSNLSTEDKGTMLIAVGDKV-NVSELSNAEKQKLLGSVL-KKGVEAQVLSPAQQQLIQQNLDKIMAEQTK--KDTIKKVNDIL--FDP-LSNTELKTT

Rickettsia sp. IG-1 VIKSNLSTEDKGTMLIAVGDKV-NVSELSNAEKQKLLGSVL-KKSVETQVLSPAQQQLMQQNLDKITAEQTK--KDTIKKVNDIL--FDP-LSNTELKTT

Candidatus R. barbariae VIKSNLSTEDKGTMLIAVGDKV-NVSELSNAEKQKLLGSVL-KKDVEAQVLSLAQQQLMQQHLDKITAEQTK--KDTIKKVNDIV--FDP-LSNTELKTT

Rickettsia sp. S VIKSNLSTEDKGTMLIAVGDKV-NVSELSNAEKQKLLGSVL-KKDVEAQVLSPAQQQLMQQHLDKITAEQTK--KDTIKKVNDIL--FDP-LSNTELKTT

Candidatus R. goldwasserii VIKSNLSTEDKGTMLIAVGDKV-NVSELSNAEKQKLLGSVL-KK----QVLSPAQQQLMQQNLDKIMAEQTK--K-------------------------

R. mongolotimonae VIKTNLSTEDKGTMLIAVGDKVNNVSELSNAEKQKSLGSVL-KKGVETQVLSPAQQQLMQQHLDKIMAEQTK--KDTIKKVNDIL--FDP-LSNTELKTT

R. sp. BJ-90 VIKTNL---------------------LSNTEKQKLLGSVL-KKGVEAQVLSPAQQQLMQQHLDKITAEQIK--KDTIKKVNDIL--FDP-LSNTELKTT

Candidatus R. andeanae VIKNNLSTEDKGTMLIAVGDKV-NVSELSNVEKQKLLGSVL-KKGVEAQVLSLAQQQLMQQNLDKITAEQTK--KDTIKKANDIL--FDP-LSNTELKTT

Rickettsia sp. Bar29 VIKSNLSTEDKGTMLIAVGDKV-NVSELSNAEKQKLLGSVL-KKGVEAQVLSPAQQQLMQQNLDKIMAEQTK--KDTIKKVNDIL--FDP-LSSTELKTT

Rickettsia sp. TwKM01 VIKSNLSTEDKGTMLIAVGDKV-NVSELSNTEKQKLLGSVL-KKGVEAQVLSPAQQQLMQQNLDKITAEQTK--KDTIKKVNDIL--FDP-LSNTELKTT

Rickettsia sp. IRS 4 AIKSNLSTEDKGTMLIAVGDKI-NASELSNAEKQKLLGSVL-KKGVEVQVLSPEQQQLMQQNLDKITAEQTK--KDTIKKVNDIL--FDP-LSNTELKTT

R. raoultii VIKS-LSTEDKGIMLIAVGDKV-NVSELSNAEKQKLLGSVL-KKGVEVQVLSPAQQQLMQQNLDKITAEQTK--KDTIKKVNDIL--FDP-LSNTELKTT

R. asiatica VIKSNLSTEDKGTMLIAVDDKV-NASELSNAEKQKLLGSVL-KKGVETQVFSPEQQQLMQQNLDKITAEQTK--KDTIKKVNDIL--FDP-LSNTELKTT

**[801-900 RRRRRRRRRRRRRRRRRRRRRRRRRRRRRRRRRRRRRRRRRRRRRRRRRRRRRRRRRRRRRRRRRRRRRRRRRRRRRRRRRRRRRRRRRRRRRRRRRRRR]**

**[ | | | | | | | | | | ]**

R. bellii RML369-C AIKTITTDVLDGPAKAEIKGEIVEGITKEVAQSPL-SIKDKVGIIEVVGEAIASHKD--MSIPEKATIASFAEDGIIKSTAELKDKELMTKGLIDGIHKG

R. bellii OSU 85-389 AIKTITTDVLDGPAKAEIKGEIVEGITKEVAQSPL-SIKDKVGIIEVVGEAIASHKD--MSIPEKATIASFAEDGIIKSTAELKDKELMTKGLIDGIHKG

R. prowazekii Madrid E NIQVMTSNVLDSPVQIEMKSKLIQVVTKTVAESALVEPKDKTEIVKGIGKTIVTHSDTSLPLHDKVVIMGSVAKGIVESKNDLLDRELIIAGLVDGIYEA

R. prowazekii Rp22 NIQVMTSNVLDSPVQIEMKSKLIQVVTKTVAESALVEPKDKTEIVKGIGKTIVTHSDTSLPLHDKVVIMGSVAKGIVESKNDLLDRELIIAGLVDGIYEA

**R. typhi Wilmington NIQVITANVLDSPVPVEIKSELIQVVTQKVAESAL-EPKDKTEIVKGIGKIIATHSDTSLPLYDKGVIMESVAKGIVESKTDLRERELITEGLVNGIYEV**

R. australis AIQKVTTKVLDSPITAEIKGETLESITKIVAESPL-NVQDKTDIVKGMGEAIASHR--TMAPTKKIAAIESVETGVAKSITDLEDKKLMTKGLVDGIYED

R. australis AIQNVTTKVLDSPIKAEIKGETLESITKVVAESPL-NGQDKVDIVKGMGEAIASHK--TMSPTEKISAIESVETGVAESITALEDKKLMTKGLVDGIYED

R. felis URRWXCal2 AIQNVTTKVLDSPIKAEIKGETLESITKVVAESPL-NGQDKADIVKGMGEAIASHK--TMAPTEKISTIESVEKGVAESITDLEDKKLMTKGLVEGIYEG

R. canadensis McKiel EIQDITTKVLDAPVKAEVKGHIIEGITKTIAESSL-DAKDKTDIVQGVGETITSHSNTFLTY--KVTIMDFAEQGIAEGKTDLPVRELMTKGLVEGVYES

R. tamurae NIQAITSNVLDSPAKVEVKSEIIEGITNTVAGSSL-EAKDKAEIVKGVGETIATHSDTSLSLPGKALIIASAEKGIAESKTDLPDRELMTKGLVDGIYEG

R. helvetica NIQAITSNVLDGPATAKVKGEIIQEITNTVAGSSL-EAQDKAAIIKGIGETIATHSDTSLSLPNKALIMASAEKGIAESQTNLPDRELMTKGLVDGIYEG

REIS_1 NIQAITS---------------------------------------------------------------------------------------------

REIS_2 KILLLTKKCLTDAKYKEFKPYIRKTAKQHIETHHE---------------AYLEEADGGKALSELCEIMQNIGDG-----------------WLDGASEK

REIS_3 KILLLTKKCLTDPKYKEFESYIKKTAEQHIELHHE---------------AYLKEADGGKSLKELTEIMKNIG---------------------------

R. massiliae MTU5 NIQAITSNVLDGPATAEVKGEIIQEITNTVAGSSL-EAQDKAEIVKGVGETIATHSDTSLSLPNKALIMASAEKGIVESKTNLPDRELMTKGLVDGIYEG

R. rhipicephali NIQAITSNVLDGPATAEVKGEIIQEITNTVAGSSL-EAQDKAEIVKGVGETIATHSDTSLSLPNKALIMASAEKGIVESKTNLPDRELMTKGLVDGIYEG

R. aeschlimannii NIQAITSNVLDGPATAEVKGGIIQEITNTVAGSSL-EAQDKAEIVKGVGETIATHSDTSLSLPNKALIMASAEKGIAESKTNLPDRELMTKWLVDGIYKG

R. montanensis NIQAIMSNVLDGPATAEVKGEIIQEITNTVAGSSL-EAQDKAAIIKGVGETIATHSDTSLSLPNKALIMASAEKGIAESQTNLPDRELMTKGLVDGIYEG

R. slovaca NIQAITSNVLDGPATAEVKGEIIQEITNTVAGSSL-EAQDKAAIIKGVGETIATHSDTSLSLPNKALIMASAEKGIAESQTNLPDRELMTKGLVDGIYEG

R. peacockii Rustic NIQAIISNVLDGPATAAVKGEIIQEITNTVAGSSL-EAQDKAAIVKGVGETIATHSDTSLSLPNKALIMASTEKGIAESQTNLPDRELMTKGLVDGIYED

R. peacockii Rustic p19 ----------------------------------------------------------------------------------------------------

R. rickettsii Iowa NIQAIISNVLDGPATAVVKGEIIQEITNTVAGSSL-EAQDKATIVKGVGETIATHSDTSLSLPNKALIMASAEKGIAESQTTLPDRELMTKGLVDGIYEG

R. rickettsii Sheila Smith NIQAIISNVLDGPATAVVKGEIIQEITNTVAGSSL-EAQDKATIVKGVGETIATHSDTSLSLPNKALIMASAEKGIAESQTTLPDRELMTKGLVDGIYEG

R. japonica NIQAIISNVLDGPATAEVKGEIIQEITNTVAGSSL-EAHDKAAIIKGISETIATHSDTSLSLPNKALIMASAEKGIAESQANLPDRELMTKGLVDGIYEG

R. honei NIQAITSNVLDGPATAEVKGEIIQEITNIVAGSSL-EAQDKAAIVKGVGETIATHSDNSLSLPNKALIMASAEKGIAESQTNLPDRELMTKGLVDGIYEG

R. conorii str. Malish 7 NIQAITSNVLDGPATAEVKGEIIQAITNTIAGSSL-EAQDKAAIIKGVGETIATHSDTSLSLPNKALIMASAEKGIAESQTNLPDRELMTKGLVDGIYEG

R. sibirica 246 NIQAITSNVLDGPATAEVKGEIIQEITNTVAGSSL-EAQDKAAIIKGIGETIATHSDTSLSLPNKALIMASAEKGIAESQTNLPDRELMTKGLVDGIYEG

R. africae ESF-5 NIQAITSNVLDGPATAEVKGEIIQEITNTVAGSSL-EAQDKAAIIKGVGETIATHSDTSLSLPNKALIMASAEKGIAESQTNLPDRELMTKGLVDGIYEG

R. parkeri NIQAITSNVLDGPATAEVKGEIIQEITNTVAGSSL-EAQDKAEIVKGVGETIATHSDTSLSLPNKALIMASAEKGIAESKTNLPDRELMTKVLVDGIYEG

R. heilongjiangensis NIQAITSNVLDGPATAEVKGEIIQEITNTVAGSSL-EAHDKAAIVKGVSETIATHSDTSLSLPNKALIMASAEKGIAESQANLPDRELMTKGLVDGIYEG

Israeli tick typhus NIQAITSNVLDGPATAEVKGEIIQAITNTVAGSSL-EAQDKAAIIKGVGETIATHSDTSLSLPNKALIMASAEKGIAESQTNLPDRELMTKGLVDGIYEG

R. sp. A-167 NIQAITSNVLDGPATAEVKGEIIQAITNTVAGSSL-EAQDKAAIIKGVGETIATHSDTSLSLPNKALIMASAEKGIAESQTNLPDRELMTKGLVDGIYEG

Candidatus R. tasmanensis NIQAITSNVLDGPATAEVKGEIIQEITNTVAGSSL-EAQDKAAIVKGVGETIATHSDTSLSLPNKALIMASAEKGIAESKTKLP----------DGIYEG

Rickettsia sp. IG-1 NIQAITSNVLDDPATAEVKGKIIQEITNTVAGSSL-EAQDKAAIVKGVGETIATHSDTSLSLPNKALIMASAQKGIAESQTNLPDRELMTKGLVDGIYEG

Candidatus R. barbariae NIQTITSNVLDGPATAEVKGEIIQEITNTVAGSSF-EAQDKAAIIKGVGETIATHSDTSLSLPNKALIMASAEKGIAESQTNLPDRELMTKGLVDGIYEG

Rickettsia sp. S NIQAITSNVLDGPATAEVKGEIIQEITNTIAGSSL-EAQDKAAIIKGVGETIATHSDTSLSLPNKALIMASAEKGIAASQTNLPDRELMTKGLVDGIYEG

Candidatus R. goldwasserii ----------------------------------------------------------------------------------------------------

R. mongolotimonae NIQAITSNVLDGPATAKVKGEIIQEITNTVAGSSL-EAQDKAAIIKGIGETIATHSDTSLSLPNKALIMASAEKGIAESQTNLPDRELMTKGLVDGIYEG

R. sp. BJ-90 NIQAITSNVLDGPATAEVKGEIIQEITNTVAGSSL-EAQDKAAIIKGVGETIATHSDTSLSLPNKALIMASAEKGIAESQTNLPDRELMTKGLVDGIYEG

Candidatus R. andeanae NIQAITSNVLDGPATAEVKGAIIQEITNTVAGSSL-EAQDKAEIVKGVGETIATHSDTSLSLLNKALIMASAEKGIAGSKTNLPDRELMTKGLVDGIYEG

Rickettsia sp. Bar29 NIQAITSNVLDGPATAEVKGEIIQEITNTVAGSSL-EAQDKAEIVKGVGETIATHSDTSLSLPNKALIMASAEKGIVESKTNLPDRELMTKGLVDGIYEG

Rickettsia sp. TwKM01 NIQAITSNVLDGPATAEVKGKIIQEITNTVAGSSL-EAQDKAEIVKGVGETIATHSNTSLSLPNKALTMASAEKGIVESKTNLPDRELMTKGLVDGIYEG

Rickettsia sp. IRS 4 NIQAITSNVLDSPAKVEVKSEIIEGITNTVAGSSL-EAKDKAEIVKGVGETIATHSDTSLSLPGKALIIASAEKGIAESKTDLPDRELMTKGLVDGIYEG

R. raoultii NIQAITSNVLDGPATAEVKGEIIQEITNTVAGSSL-EAQDKAEIVKGVGETVATHSDTSVSLPNKALIMASAEKGIAESKTNLPDRELMTKGLVDGIYEG

R. asiatica NIQAITANVLDSPAKVEVKSEIIEGITNTVAGSSL-EAKDKAEIVKGVGKTIATHSDTSLSLPDKALIMASAEKGIAESKTDLPDRELMTKGLVEGVYES

**[901-1000 RRRRRRRRRRRRRRRRRRRRRRR ]**

**[ | | | | | | | | | | ]**

R. bellii RML369-C IGNE------SPEITQGVVKSIKANAVPEEKSTLENIANEAILEREMQNLTQGLKGQDLEQDKPEKDIVKAARETMEALGNLNKAAEPGFEKGPVDDTSK

R. bellii OSU 85-389 IGNE------SPEITQGVVKSIKANAVPEEKSTLENIANEAILEREMQNLTQGLKGQDLEQDKPEKDIVKAARETMEALGNLNKAAEPGFEKGPVDDTSK

R. prowazekii Madrid E KGDNAV----VHAISSMIANSNINQSEKEALKRSQDVVSEKVLDKEIQNLDRELKAQNINESKLHDDIYNKTQDVANALKNVITTV--------------

R. prowazekii Rp22 KGDNAV----VHAISSMIANSNINQSEKEALKRSQDVVSEKVLDKEIQNLDRELKAQNINESKLHDDIYNKTQDVANALKNVITTV--------------

**R. typhi Wilmington KGDKAV----VHAISSVIANSNINQSEKEVLKKSQDIVSERVLDKEIQNLDGELKEKNIEESKLRDDIYNKTQDVANELN--IKTF--------------**

R. australis KANPEITSEMMKAVSKGVDNSTAIPEDKQAL---KDAASEAALDRATQNFTEELKGQNLDEPKPRDDIYNKAQDIAYALKNVVTTV--------------

R. australis KANPEM----TKAVSRGVDKSTARPEDKQAL---KDAASEVALDRETQNFTKGLKEQNLEKPKPRDDIYNKAQDVAEALKNVITPV--------------

R. felis URRWXCal2 KANPEITSEKTKAVSRGIDKSTAIPEDKQAL---KDAANEAALDRETQNLTEGLKRQNLGEPKPRDDIYNKAQDVADALKNVITPV--------------

R. canadensis McKiel KADPEITSAITKAVSNGIDNSTSVLEDKPAL---KDAALEAVLDRETQNLNKDL-SQTVEQTKPRDDIYNKYAVS-------------------------

R. tamurae KGGPEI----TKAVSSGIDNSNINNSEKEALKKAKDAASEAALDRETQNLTEGLKGQNIEEPKPLDDIYNKAQDVANALKNVITPV--------------

R. helvetica KGGPEI----TKAVSSGIDNSNINDSEKEALKKAKDTVSEAALDRETQNLNKDLQGQNIEEIQPHHDIYNKSQDMTDALKNVIDPV--------------

REIS_1 ----------------------------------------------------------------------------------------------------

REIS_2 AIGSDR---------KGSIAANDNNLEKSKTKQIKDILKAGG---------------------------------KLGFNVVLESV--------------

REIS_3 --------------YKSFEEENSKNNSKGASGKIKDVLKAGG---------------------------------KLGFNIVLESL--------------

R. massiliae MTU5 KGGPEI----TKAVSSGIDNSNINDSEKEALKKVKDAASEATLDIETQNLTEGLKGQNIEEHKPRDDIYNKAQEVINAVNPVIEAL--------------

R. rhipicephali KGGPEI----TKAVSSGIDNSNINDSEKEALKKAKDAASEATLDIETQNLTEGLKGQNIEEHKPRDDIYNKAQEVINAVNPVIEAL--------------

R. aeschlimannii KRGPEI----TKAVSSGIDNSNINDSEKEALKKAKGAASEAALDIETQNLTEGLKGQNIEEHKPRDDIYNKAQEVINAVNPVIEVL--------------

R. montanensis KGGPEI----TKAVSSGIDNSNSNDSEKEALKKAKDTASEAALDRDTQNLTEGLKGQNIEEHKPHDDIYNKAREVINAVNPVIEAL--------------

R. slovaca KGGPEI----TKAVSSGIDNSNINDSEKEALKKAKDAASEAALDRDTQNLTEGFKGQNIEEHKPHDDIYNKAREVINAVNPVIEAL--------------

R. peacockii Rustic KGAPEI----TKAVSSGIDNSNINDSEKEALKKAKDAASETALDRDTQNLTEGLKGQNIEEHKPHDDIYNKAREVINAVNPVIEAL--------------

R. peacockii Rustic p19 ----------------------------------------------------------------------------------------------------

R. rickettsii Iowa KGGPEI----TKAVSSGIDNSNINDSEKEALKKAKDAASETALDRDTQNLTEGLKGQNIEEHKPHDDIYNKAREVINAVNPVIEAL--------------

R. rickettsii Sheila Smith KGGPEI----TKAVSSGIDNSNINDSEKEALKKAKDAASETALDRDTQNLTEGLKGQNIEEHKPHDDIYNKAREVINAVNPVIEAL--------------

R. japonica KGGPEI----TKAVSSGIDNSNINDSEKEALKKAKDAASEAALDRETQNLTEGLKGQNIEEHKPHDDIYNKVREVINAVNPVIEAL--------------

R. honei KGGPEI----TKAVSSGIDNSNINDSEKEALKKAKDAASEAALDRDTQNLTEGLKGQNIEEHKPHDDIYNRAREVINAVNPVIEAL--------------

R. conorii str. Malish 7 KGGPEI----TKAVSSGIDNSNINDSEKEALKKAKDAASEAALDRDTQNLTEGFKGQNIEEHKPHDDIYNKAREVINAVNPVIEAL--------------

R. sibirica 246 KGGPEI----TKAVSSGIDNSNINDSEKEALKKAKDAASEAALDRDTQNLTEGLKGQNIEEHKPHDDIYNKAREVINAVNPVIEAL--------------

R. africae ESF-5 KGGPEI----TKAVSSGIDNSNINDSEKEALKKAKDAASEAALDRDTQNLTEGLKGQNIEEHKPHDDIYNKAREVINAVNPVIEAL--------------

R. parkeri KGGPEI----TKAVSSGIDNSNINDSEKEALKKAKDAASEAALDRDTQNLTEGFKGQNIEEHKPHDDIYNKAREVINAVNPVIEAL--------------

R. heilongjiangensis KGGPEI----TKAVSSGIDNSNINDSEKEALKKAKDAASEAALDRETQNLTEGLKGQNIEEHKPHDDIYNKVREVINAVNPVIEAL--------------

Israeli tick typhus KGGPEI----TKAVSSGIDNSNINDSEKEALKKAKDAASEAALDRDTQNLTEGFKGQNIEEHKPHDDIYNKAREVINAVNPVIEAL--------------

R. sp. A-167 KGGPEI----TKAVSSGIDNSNINDSEKEALKKAKDAASEAALDRDTQNLTEGLKGQNIEEHKPHDDIYNKAREVINAVNPVIEAL--------------

Candidatus R. tasmanensis KGGPGI----TKAVSSGIDNSNINDSEKEALKKAKDAASEAALDRETQNLTEGLKGQNIEEHKPRDDIYNKAQEVINTINPVIEAL--------------

Rickettsia sp. IG-1 KGGPEI----TKAVSSGIDNSNINDSQKEALKKAKDAASEAALDRDTQNLTEGLKGQNIEEHKPHDNIYNKAREVINAVNPIIEAL--------------

Candidatus R. barbariae KGGPEI----TKAVSSGIDNSNINDSEKEALKKAKDAASEAALDRDTQNLTEGLKGQNIEEHKPHDDIYNKAREVINAVNPVIEAL--------------

Rickettsia sp. S KGGPEI----TKAVSSGIDNSNINNSEKEALKKAKDAASEAALDRDTQNLTEGFKGQNIEEHKPHDDIYNKAREVINAVNPVIEAL--------------

Candidatus R. goldwasserii ----------------------------------------------------------------------------------------------------

R. mongolotimonae KGGPEI----TKAVSSGIDNSNINDSEKEALKKAKDAASEAALDRDTQNLTEGLKGQNIEEHKPHDDIYNKAREVINAVNPVIEAL--------------

R. sp. BJ-90 KGGPEI----TKAVSSGIDNSNINDSEKEALKKAKDAASEAALDRDTQNLTEGFKGQNIEEHKPHDDIYNKAREVIHAVNPVMEAL--------------

Candidatus R. andeanae KGGPEI----TKAVSSGIDNSNINDSEKEALKKAKDAASEAALDRETQNLTEGLKGQNIEEHKPRDDIYNKAQEVINAVNPVIEAL--------------

Rickettsia sp. Bar29 KGGPEI----TKAVSSGIDNSNINDSEKEALKKVKDAASEATLDIETQNLTEGLKGQNIEEHKPRDDIYNKAQEVINAVNPVIEAL--------------

Rickettsia sp. TwKM01 KGGPEI----TKAVSSGIDNSNINDSEKEALKKAKDAASEATLDIETQNLTEGLKGQNIEEHKPRDDIYHKAQEVINAVNPVIEAL--------------

Rickettsia sp. IRS 4 KGGPEI----TKAVSSGIDNSNINNSEKEALKKAKDAASEAALDRETQNLTEGLKGQNIEEPKPRDDIYNKAQDVANALKNVITPV--------------

R. raoultii KGGPEI----TKAVSSGIDNSNIKDSEKEALKKAKDAASEAALDRETQNLTEGLKGQNIEEHKPRDDIYNKAQEVINAVNPVIEAL--------------

R. asiatica KTDPEI----TKAVSSGINNSNINGSEKEALKKAKDTVSEAALDRETQNLNKDLQGQNIEEIQPHHDIYNKSQDMTDALKNVIDPV--------------

**[1001-1100 ]**

**[ | | | | | | | | | | ]**

R. bellii RML369-C QSKEQTEKPIVSE--EEKVVQETSSRLNDISQFISKKVNNLRSLLDERRNLKTNEEKKAESEKQAK------DLTEKFNEKSSTKDQLDFIQAN------

R. bellii OSU 85-389 QSKEQTEKPIVSE--EEKVVQETSSRLNDISQFISKKVNNLRSLLDERRNLKTNEEKKAESEKQAESEKQAKDLTEKFNEKSSTKDQLDFIQAN------

R. prowazekii Madrid E ---LDDNSGQRGV--SEEAPKKVSSLLNDISKRTIEKINNLRAMLSQDGNLKTFEEKKDEATKKVD------ELVKAFDNKSSTEEQQNFIKSN------

R. prowazekii Rp22 ---LDDNSGQRGV--SEEAPKKVSSLLNDISKRTIEKVNNLRAMLSQDGNLKTFEEKKDEATKKVD------ELVKAFDNKSSTEEQQNFIKSN------

R. typhi Wilmington ---LDDNHGKREV--SEEVPKNTSSLLNDISQRTIEKVNNLRAMLSQDANLKTFEEKKDESTKKVD------ELVKAFDNKSSTEEQQNFIKSH------

R. australis ---LDANPEKREVS-EEEVMNKTSSILNDISKIAIEKVNNLRAMLSPDSNLKTLEEKKAEATKKVD------ELVKEFGTKSSTEEQQSFIQAN------

R. australis ---LDAHPEKREVS-EEEVMKKTSSILNDISNLTIEKVNNFRAMLSPDSNLKTLEEKKAEATKKVD------ELVKEFGTKSSTEEQQSFIKAN------

R. felis URRWXCal2 ---LDAHPEKREVSEEEEVVKKTSSILNDISKLAIEKVNNFRAMLSPDGNLKTLEEKKAESTKKVD------ELVKEFGTKSSTEEQQSFIKAN------

R. canadensis McKiel ---------------EEEVVQKTSSILSDISKLAVEKVNNFRAMLSPNGNLNTLEAKRAKSEQEISKIVNAYKAIDEKYKVQYEQQQGSYNNTKELEEKK

R. tamurae ---LDAHPEKREVS-KEEVVKKTSNILNDISKLAVEKVNNIRAMLSPYGNLKTLEEKRAESTKKVD------ELVKEFGTKSSTEEQQSFIKAN------

R. helvetica ---LEAHS-------EEQMAKKTSSILNDISSYVERIKSTFR------DPLDIAKEKKKESIKKVD------ELVKEFGTKSSTEEQQSFIKAN------

REIS_1 ----------------------------------------------------------------------------------------------------

REIS_2 ---------------------------------------------KAGGADAYLRKQAIEGGKKVG----------------------SYVACC------

REIS_3 ---------------------------------------------KAGGVENYVKQKAIEESKKVG----------------------SYIAHC------

R. massiliae MTU5 ---AKPKEPVVSA--EERIVHETSSILNNISKLAVEKVNNFRAMLSSNGNLKTLEKKKEESIKKVD------ELVKAFGTKSSTEEQQSFIKAN------

R. rhipicephali ---EKPKAPVVSA--EERIVQETSSILNNISKLAVEKVNNFRAMLSSNGNFKTLEKKKEESIKKVD------ELVKAFGTKSSTEEQQSFIKAN------

R. aeschlimannii ---EKSKEPVVSA--EERIVQETSSILNNISKLAVEKVNNFRAMLSSNGNLKTLEEKKEESIKKVD------ELVKAFGTKSSTEEQQSFIKAN------

R. montanensis ---EKSKEPVVSS--EERIGQETSSILNNISKLAVEKVNNFRAMFSPNGNLKTLEEKKEESIKKVD------KLVKEFGTKSSTAEQQSFIKAN------

R. slovaca ---EKFKEPVVSA--EERIVQETSSILNNISKLAVEKVNNFRAMLSPTGNLKTLEEKKEESIKKVD------ELVKAFGTKSSTEEQQSFIKTN------

R. peacockii Rustic ---EKSKEPVVSA--EERIVQETSSILNNISKLAVEKVNNFRAMLSPNGNLKTLEEKKEESIKKVD------ELVKAFGTKSSTEEQQSFIKTN------

R. peacockii Rustic p19 ----------------------------------------------------------------------------------------------------

R. rickettsii Iowa ---EKSKEPVVSA--EARIVQETSSILNNISKLAVEKVNNFRAMLSPNGNLKTLEEKKEESIKKVD------ELVKAFGTKSSTAEQQSFIKTN------

R. rickettsii Sheila Smith ---EKSKEPVVSA--EARIVQETSSILNNISKLAVEKVNNFRAMLSPNGNLKTLEEKKEESIKKVD------ELVKAFGTKSSTAEQQSFIKTN------

R. japonica ---EKSKEPVVSA--EERIVQETSSILNNISKLAVEKVNNFRAMLSPNGNLKTLEEKKEESIKKVD------ELVKAFGTKSSTEEQQSFIKAN------

R. honei ---EKSKEPVVSA--EERIVQETSSILNNISKLAVEKVNNFRAMLSPNGNLKTLEETKEESIKKVD------ELVKAFGTKSSTEEQQSFIKAN------

R. conorii str. Malish 7 ---EKSKEPVVSA--EERIVQETSSILNNISKLAVEKVNNFRAMLSPNGNLKTLEEKKEEAIKKVD------ELVKAFGTKSSTEEQQSFIKTN------

R. sibirica 246 ---EKSKEPVVSA--EERIVHETSSILNNISKLAVEKVNNFRAMLSPNGNLKTLAEKKEESIKKVD------ELVKAFGTKSSTEEQQSFIKTN------

R. africae ESF-5 ---EKSKEPVVSA--EDRIVQETSSILNNISKLAVEKVNNLRAMLSPNGNLKTLEEKKEESIKKVD------ELVKAFGTKSSTEEQQSFIKTN------

R. parkeri ---EKSKEPVVSA--EERIVQETSSILNNISKLAVEKVNNLRSMLSPNGNLKTLEEKKEESIKKVD------ELVKAFGTKSSTEEQQSFIKTN------

R. heilongjiangensis ---EKSKEPVVSA--EKRIVQETSSILNNISKLAVEKVNNFRAMLSPNGNLKTLEEKKEESIKKVD------ELVKAFGTKSSTEEQQSFIKAN------

Israeli tick typhus ---ETSKEPVVSA--EERIVQETSSILNNISKLAIEKVNNFRAMLSPNGNLKTLEEKKEESIKKVD------ELVKAFGTKSSTEEQQSFIKTN------

R. sp. A-167 ---EKSKEPVVSA--EERIVQETSSILNNISKLAIEKVNNFRAMLSPNGKLKTLEEKKEESIKKVD------ELVKAFGTKSSTEEQQSFIKTN------

Candidatus R. tasmanensis ---EKSKEPVVSA--EEKIVQETSSILNNISKLAVEKVNNFRAMLSPNGNLKILEEKKEESIKKVD------ELVKAFSTKSSTEEQQSFIKAN------

Rickettsia sp. IG-1 ---EKSKEPVVSA--EERIVQETSSILNNISKLAVEKVNNFRAMLSSNGNLKTLEEKKEDSIKKVD------ELVKAFGTKSSTEEQQSFIKAN------

Candidatus R. barbariae ---EKSKEPVVSA--EERIVQETSSILNNISKLAVEKVNNFRAMLSRNGNLKTLEEKKEESIKKVD------ELVKAFGTKSSTEEQQSFIKTN------

Rickettsia sp. S ---EKSKEPVVSA--EERIVQETSSILNNISKLAVEKVNNFRAMLSPNGNLKTLEEKKEESIKKVD------ELVKAFGTKSSTEEQQSFIKTN------

Candidatus R. goldwasserii ----------------------------------------------------------------------------------------------------

R. mongolotimonae ---EKSKEPVVSA--EERIVQETSSILNNISKLAVEKVNNFRAMLSPNGNLKTLEEKKEESIKKVD------ELVKAFGTKSSTEEQQSFIKTN------

R. sp. BJ-90 ---EKSKEPVVSA--EERIVHETSSILNNISKLAVEKVNNFRAMLSPNGNLKTLAEKKEESIKKVD------ELVKAFGTKSSTEEQQSFIKTN------

Candidatus R. andeanae ---EQSKEPVVSAE-EERIVQETSSILNNISKLAVEKVNNLRAMLSPNGNLKTLEEKKEESIKKVD------ELVK------------------------

Rickettsia sp. Bar29 ---AKPKELVVSA--EERIVQETSSILNNISKLAVEKVNNFRAMLSSNGNLKTLEQKKEKPIKKVD------ELVKAFGTKSSTEEQQSFIKAN------

Rickettsia sp. TwKM01 ---EKPKEPVVSA--EERIVQETSSILNNISKLAVEKVNNFRAMLSSNGNLKTLEKKKEESIKKVD------ELVKAFGTKSSTEEQQSFIKAN------

Rickettsia sp. IRS 4 ---LDAHPEKREVS-KEEVVKKTSNILNDISKLAVEKVNNFRAMLSPDGNLKTLEEKRAESTKKVD------ELVKEFGTKSSTEEQQSFIKAN------

R. raoultii ---EKSKEPVVSA--EERIVQETSSILNNISKLAVEKVNNFRAILSSNGNLKTLAEKKEESIKKVD------ELVKEFGTKSSTEEQQSFIKAN------

R. asiatica ---LEAHS-------EEQMAKKTSSILNDISSYVERIKSTFR------DPLDIAKEKKKESIKKVD------ELVKEFGTKSSTEEQQSFIKAN------

**[1101-1200 ]**

**[ | | | | | | | | | | ]**

R. bellii RML369-C -LIDNKDLSKDARLKAIDNLLQEQVEKRGAAVSGQSQDKTEDVRTLSGKSELKPVSRDEPDIE-RAKMVVGKDKVNIKDNVAIMAKLTDAKSA-IQLENP

R. bellii OSU 85-389 -LIDNKDLSKDARLKAIDNLLQEQVEKRGAAVSGQSQDKTEDVRTLSGKSELKPVSRDEPDIE-RAKMVVGKDKVNIKDNVAIMAKLTDAKSA-IQLENP

R. prowazekii Madrid E -LIDNKTLSREIRLQIIDNLLKAQAQKRAETIENLS-AKTEDVRVISGKSELKPISQDEPYIQ-KAKMVVERDRVDIKDNIKIMSALINARDS-IQSENF

R. prowazekii Rp22 -LIDNKTLSREIRLQIIDNLLKAQAQKRAETIENLS-AKTEDVRVISGKSELKPISQDEPYIQ-KAKMVVERDRVDIKDNIKIMSALINARDS-IQSENF

R. typhi Wilmington -LIDNKTLSREVRLQIIDNLLKAQAQKRAETIENLS-AKTEDVRVVSGKSELEPISKDEPYIQ-KAKMVVERDRVGIKDNIKIMGALINARDS-IQSENL

R. australis -LIDDKTLSKEVRLQTIDKLLQEQAQKRAEAIKNPN-VKTEDLRVVSGQSALKPISNDEPDIE-KTKMVVGRDRVNIKDNIKIMGALMNARDSIIQSEKL

R. australis -LTDDKTLSKEVRLQTIDKLLQEQAQKRAKAIENPN-VKTEDVRVVSEQSELKPISNDEPGIE-KTKMVVGRDRVNIKDNIKIIGALMNARDSIIQSENL

R. felis URRWXCal2 -LIDDKTLSKEIRLQTINKLLQEQAQKRAEAIENPN-VKTEDVRVVSG--------------------------VNIKDNIKIMGALMNARDSIIQSENL

R. canadensis McKiel LFIENLKLEDKVKIKLISTLIQNEAQNKKKGTVNNITMLTENRTKLEAKVAIQDKSKLGYDAQLKPRKNTGRVH--------------------------

R. tamurae -LIDDKTLSKEVRLQTIDKLLQEQAQKRADAVENRS-VKTKDVRVVSGQSELKPISKDEPDIE-KAKMVVERDRVNIKDNIKIMGALMNARDS-IQSEIL

R. helvetica -LIDDKTLSKEVRLQTIDKLLQEQEQKQAEAVENPS-IKTEDVRVVSGQSELKPISKDEPDIE-KAKMVVERDRVNIKDNIKIMGALMNAXDS-IQLENL

REIS_1 ----------------------------------------------------------------------------------------------------

REIS_2 -IAD----------RIKGRLCGKELHANDNGVSAKT-FKSCVVSASHSKTKAPEVSR-------------------------------------------

REIS_3 -VAD----------RIKGTLCGKQSYANDNGVSAKT-FKSCVVSASHSKNKSSEVLR-------------------------------------------

R. massiliae MTU5 -LIDDKTLSKEVRLQTIDKLLQEQTQKQAEAIENPS-VKTEDVRVVSGKSELKPISKDTPDIE-KAKMVVERDRVNIKENIKIMGALMNARDS-IQSENV

R. rhipicephali -LIDDKTLSKEVRLQTIDKLLQEQTQKQAEAIENPS-VKTEDVRVVSGKSELKPISKDTPDIE-KAKMVVGRDRVNIKENIKIMGALMNARDS-IQSENV

R. aeschlimannii -LVDDKTLSKEVRLQTIDKLLQEQVQKQAEAIENPS-VKTEDVRVVSGKSELKPISKDKPDIE-KAKMVVGRDRVNIKENIKIMGALMNARDI-IQSENL

R. montanensis -LIDDKTLSKEVRLQTIDKLLQEQTQKRAEAIENPS-VKTEDVRVVSGKSKLKPISKDKPDIE-KAKMVVGRGRVNIEENIKIMGALMNARDS-IQSENL

R. slovaca -LIDDKTLSKEVRLQTIDKLLQEQ--KRAEAIENPS-VKTEDVRVVSGKSKLKPISKDNPDIE-KAKMVVGRDRVNIKGNIKIMGALMNARDI-IQSENL

R. peacockii Rustic -LIDDKTLSKEVRLQTIDKLLQEQ--KRAEAIENPS-VKTEDVRVVSGKSKLKPISKDNPDIE-KAKMVVGRDRVNIKGNIKIMGTLMNARDI-IQSENL

R. peacockii Rustic p19 ----------------------------------------------------------------------------------------------------

R. rickettsii Iowa -LIDDKTLSKEVRLQTIDKLLQEQ--KRAEAIENPS-VKTEDVRVVSGKSQLKPISKDNPDIE-KAKMVVGRDRVNIKGNIKIMGALMNARDI-IQSENL

R. rickettsii Sheila Smith -LIDDKTLSKEVRLQTIDKLLQEQ--KRAEAIENPS-VKTEDVRVVSGKSQLKPISKDNPDIE-KAKMVVGRDRVNIKGNIKIMGALMNARDI-IQSENL

R. japonica -LIDDKTLSKEIRLQTIDKLLQEQ--KRAEAIENPS-VKTEDVRVVSGKSKLKPISKDKPDIE-KAKMVVGRDRVNIKGNIKIMRALMNARDS-IQSENL

R. honei -LIDDKTLSKEVRLQTIDKLLQEQ--KRSEAIENPS-VKTEDVRVVSGKSKLKPISKDNPDIE-KAKMVVGRDRVNIKGNIKIMGALMNARDI-IQSENL

R. conorii str. Malish 7 -LIDDKTLSKEVRLQTIDKLLQEQ--KRSEAIENPS-VKTEDVRVVSGKSKLKPISKDNPDIE-KAKMVVGRDRVNIKGNIKIMGALMNARDI-IQSENL

R. sibirica 246 -LIDDKTLSKEVRLQTIDKLLQEQ--KRAEAIENPS-VKTEDVRVVSGKSKLKPISKDNPDIE-KAKMVVGRDRVNIKGNIKIMGALMNARDI-IQSENL

R. africae ESF-5 -LIDDKTLSKEVRLQTIDKLLQEQ--KRAEAIENPS-VKTEDVRVVSGKSKLKPISKDNPDIE-KAKMVVGRDRVNIKGNIKIMGALMNARDI-IQSENL

R. parkeri -LIDDKTLSKEVRLQTIDKLLQEQ--KRAEAIENPS-FKTEDVRVVSGKSKLKPISKDNPDIE-KAKMVVGRDRVNIKGNIKIMGALMNARDI-IQSENL

R. heilongjiangensis -LIDDKTLSKEIRLQTIDKLLQEQ--KRAEAIENPS-VKTEDVRVVSGKSKLKPISKDKPDIE-KAKMVVGRDRVNIKGNIKIMRALMNARDS-IQSENL

Israeli tick typhus -LIDDKTLSKEVRLQTIDKLLQEQ--KRAEAIENPS-VKTEDVRVVSGKSKLKPISTDNPDIE-KAKMVVGRDRVNIKGNIKIMGALMNARDI-IQSENL

R. sp. A-167 -LIDDKTLSKEVRLQTIDKLLQEQ--KRAEAIENPS-VKTEDVRVVSGKSKLKPISTDNPDIE-KAKMVVGRDRVNIKGNIKIMGALMNARDI-IQSENL

Candidatus R. tasmanensis -LIDDKTLSKEVRLQTIDKLLQEQ--KRAEAIENPS-VKTEDVRVVSGKSKLKPISKDKPDIE-KAKMVVGRDRVNIKDNIKIMGALMNARNS-IQSENL

Rickettsia sp. IG-1 -LIDDKTLSKEVRLQTIDKLLQEQ--KRAEAIENPS-VKTEDVRVVSGQFKLKPISKDNPDIE-KAKMVVGRDRINIKGNIKIMGALMNARDI-IQSENL

Candidatus R. barbariae -LIDDKTLSQEVRLQTIDKLLQEQ--KRAEAIENPS-VKMEDVRVV-----------------------VGRDRVNIKGNIKIMGALMNARDI-IQSKNL

Rickettsia sp. S -LIDDKTLSKEVRLQTIDKLLQEQ--RRAEAIENPS-VKTEDVRVLSGKSKLKPISKDNPDIE-KAKMVVGRDRVNIKGNIKIMGALMNARDI-IQSENL

Candidatus R. goldwasserii ----------------------------------------------------------------------------------------------------

R. mongolotimonae -LIDDKTLSKEVRLQTIDKLLQEQ--KRAEAIENPS-VKTEDVRVVSGKSKLKPISKDNPDIE-KAKMVVGRDRVNIKGNIKIMGALMNARDI-IQSENL

R. sp. BJ-90 -LIDDKPLSKEVRLQTIDKLLQEQ--KRAEAIENPS-VKTEDVRVVSGKSKLKPISKDNPDIE-KAKMVVGRDRVNIKGNIKIMGALMNARDI-IQSENL

Candidatus R. andeanae ----------------------------------------------------------------------------------------------------

Rickettsia sp. Bar29 -LIDDKTLSKEVRLQTIDKLLQEQTQKQAEAIENPS-VKTEDVRVVSGKSELKPISKDTPDIE-KAKMVVGRDRVNIKENIKIMGALMNARNS-IQSENV

Rickettsia sp. TwKM01 -LIDDKTLSKEVRLQTIDKLLQEQTQKQAEAIENPS-VKTEDVRVVSGKSELKPISKDTPDIE-KAKMVVGRDRVNIKENIKIMGALMNARDS-IQSENV

Rickettsia sp. IRS 4 -LIDDRTLSKEVRLQTIDKLLQEQAQKRADAVENRS-VKTKDVRVVSGQSELKPISKDEPDIE-KAKMVVERDRVNIKDNIKIMGALMNARDS-IQSEIL

R. raoultii -LIDAKTLSKEVRLQTIDKLLQEQAQKRAEAIENPS-VKMEDVRVVSGKSELKPISKDKPDTE-KAKMVVGRDRVNIKENIKIMEALMNARDS-IQAENL

R. asiatica -LINDKTLSKEVRLQTIDKLLQEQEQKQAEAVENPS-VKTEDVRVVSGQSELKPISKDEPDIE-KAKMVVERDRVNIKDNIKIMGALMNARDS-IQSENL

**[1201-1219 ]**

**[ | | ]**

R. bellii RML369-C TISNVKAPNNKKGQSFP--

R. bellii OSU 85-389 TISNVKAPNNKKGQSFP--

R. prowazekii Madrid E N----KSIHIKKESSFPQR

R. prowazekii Rp22 N----KSIHIKKESSFPQR

R. typhi Wilmington N----KSTHIKKESSVPQR

R. australis N----KLIPIKKESAFPQR

R. australis P------------------

R. felis URRWXCal2 N----KSTPIKRESSFPPR

R. canadensis McKiel -------------------

R. tamurae N----KSKPIKRES-----

R. helvetica N----KSTPIKRESS----

REIS_1 -------------------

REIS_2 -------------------

REIS_3 -------------------

R. massiliae MTU5 N----KSTPIKRESSPLQR

R. rhipicephali N----KSTPIKRE------

R. aeschlimannii N----KSIPIKRE------

R. montanensis N----KSTPIKRE------

R. slovaca N----KSTPIKRE------

R. peacockii Rustic N----KSTPIKRESSPPQR

R. peacockii Rustic p19 -------------------

R. rickettsii Iowa N----KSTPIKRESSPPQR

R. rickettsii Sheila Smith N----KSTPIKRESSPPQR

R. japonica N----KSTPIKRESSPPQR

R. honei N----KSTPIKRE------

R. conorii str. Malish 7 N----KSTPIKRESSPPQR

R. sibirica 246 N----KSTPIKRESSPPQR

R. africae ESF-5 N----KSIPIKRESSPPQR

R. parkeri N----KSTPIKRE------

R. heilongjiangensis N----KSTPIKRESPPPQR

Israeli tick typhus N----KSTPIKRE------

R. sp. A-167 N----KSTPIKRE------

Candidatus R. tasmanensis N----KLTPIKRESSPPQR

Rickettsia sp. IG-1 N----KSTPIKRE------

Candidatus R. barbariae N----KSTPIKRESSP---

Rickettsia sp. S N----KSKPIKRE------

Candidatus R. goldwasserii -------------------

R. mongolotimonae N----KSTPIKRE------

R. sp. BJ-90 N----KSTPIKRE------

Candidatus R. andeanae -------------------

Rickettsia sp. Bar29 N----KSTPIKRE------

Rickettsia sp. TwKM01 N----KSTPIKRE------

Rickettsia sp. IRS 4 N----KSKPIKRES-----

R. raoultii N----KSTPIKRE------

R. asiatica N----KSTPIKRESS----

**[1-100 ]**

**[ | | | | | | | | | | ]**

R. bellii RML369-C MM--------------------------------------------------------------------------------------------------

R. bellii OSU 85-389 -MDQKPNFLKRLISKRLIKTISTAAFIAAASSSTMGASVQKTVNV----------------------------------------PTASFGTGTDWLPAV

R. prowazekii Madrid E -MAQKPNFLKKIISAGLVTASTATIVAG-FSGVAMGAAMQYNRTT-NAAATTFDGIGFDQ------AAGANIPVAPNSVITANANNPITFNTPNGHLNSL

R. prowazekii Rp22 -MAQKPNFLKKIISAGLVTASTATIVAG-FSGVAMGAAMQYNRTT-NAAATTFDGIGFDQ------AAGANIPVAPNSVITANANNPITFNTPNGHLNSL

**R. typhi Wilmington -MAQKPNFLKKIISAGLVTASTATIVAG-FSGVAMGAVMQYNRTT-NAAATTVDGAGFDQT-----GAGVNLPVATNSVITANSNNAITFNTPNGNLNSL**

R. akari Hartford -MAQKPNFLKKLISAGLVTASTATIVAG-FAGSAMCAATQQNRTT-VGAATTLDGVGFDQD-----AANANLAVVPNAVITANVNHGIDFNAPAGSLNGL

R. australis ----------------------------------MGAATQQNRTT-VGVATTVDGAGFDQT-----AANANLAVAPNAVITANSNNGISFNTPAGNFNGL

R. felis URRWXCal2 -MAQKPNFLKKLISAGLVTASTATIVAG-FAGSAMGAATQQNRTT-VGAATTVDGAGFDQT-----AAPANLAVAPNAVITANANNGINFNTPAGSFNGL

R. canadensis McKiel -MAQKPNFLKKLISAGLVTASTATIVAG-FAGSAMGAANRNINAHVNLNAVGMPGIVTGDSLTYVVQAGGPFIATADGGNDNVGNPTVLFGSMNMLQSGV

R. tamurae ----------MGAANRDINANVNLDVVG-IPGIVTGDSLTYVVQA---------GGPCTAT-----ATGGN---------DNGGNATVLFGSINMLQNGV

R. helvetica ----------MGAANRDINANVNLDVVG-IPGIVTGDSLTYVVQA---------GGPCTAT-----ATGGN---------DNGGNATVLFGSINMLQNGV

REIS -MAQKPNFLKKLISAGLVTASTATIVAS-FAGSAMGALLATNGNT---------ALNAPAN-----WNPAQAPATGDTLRIANNGDRVTLDSVGFNVAAL

R. monacensis -MAQKPNFLKKLISAGLVTASTAAIVAG-FAGSAMGALLATNGNT---------ALNAPAN-----WNPAQAPATGDTLRIANNGDRVTLDSVGFNVAAL

R. massiliae MTU5 MMAQKPNFLKKLISAGLVTASTATIVAS-FAGSAMGAATQQNRTT-NGAATTVDGAGFDQT-----VALANVAVALNAVITANANNGINLNTPAGSFNGL

Candidatus R. amblyommii ---------------------------S-FAGSAMGAAIQQNRTT-NGVATTVDGVGFDQT-----AAAANVAVALNAVITANANNDINFNTPAGSFNGL

R. rhipicephali ----------------------------------MGAATQQNRTT-NRAATTVDGAGFDQT-----VAPANVAVALNAVITANANNGINLNTPAGSFNGL

R. aeschlimannii --------------------STATIVAS-FAGAAMGAATQQNRTT-NGAATTVDGAGFDQT-----AAPANVAVALNAVITANANNGINLNTPDGSFNGL

R. montanensis ----------------------------------MGAATQQNRTT-NGAATTVDGAGFDQT-----AAQVNAAVAPNAVITANANNGINFNTPAGSFNGL

R. slovaca 13-B ----------------------------------MGAAIQQNRTT-NGVATTVDGAGFDQT-----AVPANVAVALNAVITANAHKGINLNTPAGSFNGL

R. peacockii Rustic -MAQKPNFLKKLISAGLVTASTATIVAS-FAGSAMGAAIQQNRTT-NGAATTVDGAGFDQT-----AVLANVGVALNAVITANANNGINLNTPAGSFNGL

R. rickettsii Iowa -MAQKPNFLKKLISAGLVTASTATIVAS-FAGSAMGAAIQQNRTT-NGAATTVDGAGFDQT-----AAPANVGVALNAVITANANNGINFNTPAGSFNGL

R. rickettsii Sheila Smith -MAQKPNFLKKLISAGLVTASTATIVAS-FAGSAMGAAIQQNRTT-NGAATTVDGAGFDQT-----AAPANVGVALNAVITANANNGINFNTPAGSFNGL

R. japonica -MAQKPNFLKKLISAGLVTASTATIVAS-FAGSAMGAAIQQNRTT-NGVATTVDGVGFDQT-----VALANVAVAPNAVITANANNGINLNTPAGSFNGL

R. honei ----------------------------------MGAAIQQNRIT-NRAATTVDGVGFDQN-----ATPADVAVALNAVITANANNGITLNTPAGSFNGL

R. conorii str. Malish 7 -MAQKPNFLKKLISAGLVTASTATIVAS-FAGSAMGAAIQQNRTT-NAVATTVDGVGFDQT-----AVPANVAVPLNAVITAGVNKGITLNTPAGSFNGL

R. sibirica 246 -MAQKPNFLKKLISAGLVTASTATIVAS-FAGSAMGAAIQQNRTT-NANATTVDGAGFDQT-----AVPANVAVPLNAVITANVHKGINLNTPAGSFNGL

R. africae ESF-5 -MAQKPNFLKKLISAGLVTASTATIVAS-FAGSAMGAAIQQNRTT-NADATTVDGAGFDQT-----AVPANVAVPLNAVITASVHKGINLNTPAGSFNGL

R. parkeri ----------------------------------MGAAIQQNRTT-NADATTVDGAGFDQT-----AVPANVAVPLNAVITAAAHKGINLNTPAGSFNGL

R. heilongjiangensis -MAQKPNFLKKLISAGLVTASTATIVAS-FAGSAMGAAIQQNRTTNNGVATTVDGVGFDQT-----AGLVNVAVAPNAVITANANNGINLNTPAGSFNGL

Israeli tick typhus ----------------------------------MGAAIQQNRTT-NAVATTVDGAGFDQT-----AALGNVAVPINAVITANVHQGINLNTPAGSFNGL

Rickettsia sp. A-167 ----------------------------------MGAAIQQNRTT-NAVATTVDGAGFDQT-----AILGNVAVPINAVITANVHQGINLNTPAGSFNGL

Candidatus R. tasmanensis ------------ASAGLVTASTATIVAS-FAGSAMGAAIQQNRTT-NGAATTVDGAGFDQM-----AAPANVAVALNAVITANANNGINLNTPAGSFNGL

Rickettsia sp. IG-1 -------------------------------------AIQQNRTT-NGAATTVDGAGFDQA-----AAPANVAVALNAVITANANNGINLNTPAGSFNGL

Candidatus R. barbariae ---------------------TATIVAS-FAGSAMGAAIQQNRTT-NADATTVDGVGFDQT-----AVPANVAVPLNAVITASVHKGINLNTPAGSFNGL

Rickettsia sp. S ----------------------------------MGAAIQQNRTT-NADATTVDGVGFDQT-----AVLANVAVPLNAVITASVHKGINLNTPAGSFNGL

Candidatus R. goldwasserii -----------------------TMVAS-FAGSAMGAAIQQNRTT-NGVATTVDGAGFDQN-----AVPANVAVALNAVVTANANNVINLNTPAGSFNGL

Rickettsia mongolotimonae --------------------STATIVAS-FAGSAMGAAIQQNRTT-NADATTVDGAGFDQT-----AVPANVAVPLNAVITANVHKGINLNTPAGSFNGL

Rickettsia sp. BJ-90 --------------------STATIVAS-FAGSAMGAAIQQNRTT-NAIATTVDGAGFDQT-----AVPANVAVPLNAVITANVHKGINLNTPAGSFNGL

Candidatus R. andeanae -------------------------------GSAMGAAIQQNRTT-NGAATTVDGAGFDQT-----AAAANVAVALNAVITANANNGINLNTPAGSFNGL

Rickettsia sp. Bar29 ----------------------------------MGAATQQNRTT-NGAATTVDGAGFDQT-----VAPANVAVALNAVITANANNGINLNTPASSFNGL

Rickettsia sp. TwKM01 ----------------------------------MGAATQQNRTT-NGAATTVDGAGFDQT-----VAPANVAVALNAVITANANNGINLNTPAGSFNGL

R. raoultii --------------------STATIVAS-FAGSAMGAATQQNRTT-NGAATTVDGAGFDQT-----AAPANVAVALNAVITANANNGINLNTPAGSFNGL

R. asiatica ----------MGAANRDINANVNLDAAG-IPGIVTGDSLTYIVQA---------GGPYTAT-----ATGGN---------DNGGNATVLFGSINMLQNGV

Rickettsia sp. 110 --------------------NVRLEQAGVGTGIVTGDSLTYVVQA---------GGPFTAT-----ATGGN---------DADDHPTVLFGSISVLQNGV

Rickettsia sp. mmx79 --------------------NVRLEQAGVGTGIVTGDSLTYVVQA---------GGPFTAT-----ATGGN---------DADDHPTVLFGSISVLQNGV

Rickettsia sp. 103 --------------------NVNLEQAG-VPGIVTGDSLTYVVQA---------GGPFTAT-----ATGGN---------DDAANPTVLFGSISVLQNGV

Rickettsia sp. J52 --------------------NVNLEQAV-VPGIVTGDSLTYVVQA---------GGPFTAT-----ATGGN---------DNAANPTVLFGSISVLQNGV

Candidatus R. hoogstraalii -MAQKPNFLKKLISAGLVTASTATIVAG-FAGSAMGAATQQNRTT-AGAATTVDGAGFDQT-----AALANLAVAPNAVITANANDGINFNTPAGSFNGL

Rickettsia sp. California 2 ----------------------------------MGAATQQNRTT-VGAATTVDGAGFDQT-----AAPANLAVAPNAVITANANNGINFNTPAGSFNGL

R. hulinensis ----------------------------------MGAAIQQNRTT-NGVATTVDGVGFDQT-----VALANVAVAPNAVITANANNGINLNTPAGSFNGL

**[101-200 ]**

**[ | | | | | | | | | | ]**

R. bellii RML369-C --MNEASNL-------------------------------------------------------------------------------------------

R. bellii OSU 85-389 A-VGNGDSL-LYKCTGNTIIGNVNN------------------TVTINN-NGI--------------------------NPILQIGTAT-----------

R. prowazekii Madrid E F-LDTANDL-AVTINEDTTLGFITNIAQ-QAKFFNFTVA-AGKILNITG-QGITVQEASNTINAQNALTKVHGGAAINANDLSGLGSITFAAAPSVLEFN

R. prowazekii Rp22 F-LDTANDL-AVTINEDTTLGFITNIAQ-QAKFFNFTVA-AGKILNITG-QGITVQEASNTINAQNALTKVHGGAAINANDLSGLGSITFAAAPSVLEFN

**R. typhi Wilmington F-LDTANTL-AVTINENTTLGFVTNVTK-QGNFFNFTIG-AGKSLTITG-HGITAQQAATTKSAQNVVSKVNAGAAINDNDLSGVGSIDFTAAPSVLEFN**

R. akari Hartford L-LNTANDL-AVTVSENTTLGFVTNAAN-QANFFNLTLA-AGKTLAITG-QGITAAQAGATKNAQNVVAQVNGGNAVANNDLSGLGTIDFGGVPSALVFN

R. australis F-LDTANNL-AVTVNENTTLGFITKAAN-NANFFNLTLA-AGKTLTITG-QGITAAQAGATQNAQNVVTQVNGGNAIANNDLSSLGTTDFGAAPSTLVFN

R. felis URRWXCal2 F-LDTANNL-AATVSEDTTLGFITNAAN-NGNFFNFTLG-AGKTLTITG-QGITAGQAAATKNAQNAVAQVNGGNAIANNDLSGVGTIDFGAAPSTLVFN

R. canadensis McKiel F---------AVNGAADMAIGSVSGT---AGQLLTLNII-AGNTLTLNGAPGV---------------------AALPVNTYTNLGPVNFTDANSVFKVN

R. tamurae F---------AVN-GADIAIGSVSGT---AGQLLTVNI--AGNTLTLNGAPGV---------------------AAFPVNTYTNLGPVNFVDAAAVFKVS

R. helvetica F---------AVN-GADIAIGSVSGT---AGQLLTVNI--AGNTLTLNGAPGV---------------------AAFPVNTYTNLGPVNFVDAAAVFKVS

REIS I-IDTGGNVEAVTLSKSLNIGSIVGVA--GADLANITVNQAGQVLTLTGTQGL-------------------GAAANQVNIYSGLGAIDFTNLAFTLNFN

R. monacensis I-IDTGGNAEAVTLSKSLNIGSIVGVA--GADLANITVNQAGQVLTLTGTQGL-------------------GAAANQVNIYSGLGAIDFANLAFTLNFN

R. massiliae MTU5 F-LDTANNV-AVTVSADTTLGFITNAAN-NGNSFDLTLG-AGKTLTITG-QGITNAQAAATKNAQNVVAPFNGGAAIANNDLSGVGTIDWGAADSTLVFN

Candidatus R. amblyommii S-LDTANNL-AVTVSADTTLGFITNAVN-KGNSFNFTLD-AGKTLTITG-QGITNAQAAVTNNAQNVVAPFNGGAAIDNNDLSGVGTIDFGAAASTLVFN

R. rhipicephali F-LDTANNL-AVTVSADTTLGFITNAAN-QGNSFDLMLG-AGKTLTITG-QGITNAQAAVTKNAQNVVAQFNGGAAIANNDLSGVGTIDWGAADSTLVFN

R. aeschlimannii F-LDTANNL-AVTVSADTTLGFITNAAN-NANSFDLRVG-AGKTLTITG-QGITNAQAAVTKNAQNVVAQFNGGAAIANNDLSGVGTIDFGAAASTLVFN

R. montanensis F-LGTANNL-AVTVSADTTLGFVTNVAN-NGNSFNLTLG-AGKTLTITG-QGITNAQAAVTNNAQNVVAQFNGGVAVANNDLSGVGAIDFGAAASTLVFN

R. slovaca 13-B F-LDTANNL-AVTVSEDTTLGFITNVVN-NANFFNLTLN-AGKTLTITG-QGITNAQAAATKNAQNVVVQCNNGAAIGNNDLKGVGSIDFGAAASTLVFN

R. peacockii Rustic F-LNTANHL-AVTVSEDTTLGFITSAVN-NAHSFNLTLN-AGKTLTITG-QGITNAQAAATKNAQNVVVQFNNGAAIDNNDLKGVGRIDFGAAASTLVFN

R. rickettsii Iowa L-LNTANNL-AVTVSEDTTLGFITNVAH-NAHSFNLTLN-AGKTLTITG-QGVTNAQAAATKNAQNVVVQFNNGAAIDNNDLKGVGRIDFGAPASTLVFN

R. rickettsii Sheila Smith L-LNTANNL-AVTVSEDTTLGFITNVVH-NAHSFNLTLN-AGKTLTITG-QGVTNAQAAATKNAQNVVVQFNNGAAIDNNDLKGVGRIDFGAPASTLVFN

R. japonica F-LSNANNL-AVTVSEDTTLGFINNAAN-NANRFNLTLD-AGKTLTITG-QGITNVQSAATHNAQNIVAKFNGGAAIANNDLSGLGTIDFGAAASTLVFD

R. honei L-LNTANNL-VVTVGEDTTLGFITRAVN-NVNSFNLTLN-AGKTLTITG-QGITNAQAAATQNAKNVVVQFNNGAAIDNNDLKGVGRIDFHAAPSTLVFN

R. conorii str. Malish 7 F-LNTANNL-DVTVREDTTLGFITNVVN-NANHFNLMLN-AGKTLTITG-QGITNVQAAATKNANNVVAQVNNGAAIDNNDLQGVGRIDCGAAASTLVFN

R. sibirica 246 F-LNTANNL-AVTVSEDTTLGFITHVVN-NANFFNLTLN-AGKTLTITG-QGITNVQAAATKNAQNVVVQVNNGAAIENNDLQGVGTIDFGAAASTLVFN

R. africae ESF-5 F-LNTANNL-AVTVSEDTTLGFITRAVN-NANFFNLTLN-AGKTLTITG-QGITNVQAAATKNAQNVVVQVNNGAAIENNDLQGVGTIDFGAAASTLVFN

R. parkeri F-LNTANNL-AVTVSEDTTLGFITRAVN-NANFFNLTLN-AGKTLTITG-QGITNVQAAATKNAQNVVVQVNNGAAIENNDLQGVGEIDFGAAASTLVFN

R. heilongjiangensis F-LNNANNL-AVTVSEDTTLGFINN-AN-NANHFNLTLG-ADKTLTITG-QGITNVQAAATHNAQNIVAQFNGGAAIANNDLSGLGTIDFGAAASTLVFD

Israeli tick typhus F-LNTANNL-DVTVIEDTTLGFITHAVN-NANHFNLTLN-AGKTLTITG-QGITNVQAAATKNAQNVVVQVNNGAAIDNNDLQGVGRIDFGAAASTLVFN

Rickettsia sp. A-167 F-LNTANNL-DVTVSEDTTLGFITHAVNHNANHFSLTLN-AGKTLTITG-QGITNVQAAATKNAQNVVVQVNNGAAIDNNDLQGVGRIDFGAAASTLVFN

Candidatus R. tasmanensis F-LDTANNL-AVTVSADTTLGFITNAAN-KGNSFNLTLG-AGKTLTITG-QGITNAQAAVTHNAKNVVVQFNGGAAIANNDLSGVGTIDFGAAASTLVFN

Rickettsia sp. IG-1 F-LETANHL-AVTVSEDTTLGFITRAVN-NVHSFNLTLN-AGKTLTITG-KGITNAQAAATQNAQNVVVQFNNGAAIDNNDLKGVGRIDFGAAASTLVFN

Candidatus R. barbariae F-LNTENDL-AVTVSEDTTLGFITRAVN-NANFFNLTLN-AGKTLTITG-QGITNVQAAATKNAQNVVVQVNNGAAIENNDLQSVGTIDFGAAASTLVFN

Rickettsia sp. S F-LNTANNL-AVTVSEDTTLGFITRAVN-NVNFFNLTLN-AGKTLTITG-QGITNVQAAATKNAQNVVVQVNNGAAIENNDLQGVETIDFGAAASTLVFN

Candidatus R. goldwasserii F-LDTANHL-AVTVSEDTTLGFITNVVN-NGHFFNLTLN-AGKTLTITG-QGITNAQAVATQNAKNVVVQFNNGGAIDNNDLRGVGRIDFGAAASTLVFN

Rickettsia mongolotimonae F-LNTANNL-AVTVSEDTTLGFITHVVN-NANFFNLTLN-AGKTLTITG-QGITNVQAAATKNAQNVVVQVNNGAAIENNDLQGVGTIDFGAAASTLVFN

Rickettsia sp. BJ-90 F-LNTANNL-AVTVSEDTTLGFITHVVN-NANFFNLTLN-AGKTLTITG-QGITNVQAAATKNAQNVVVQVNNGAAIENNDLQGVGTIDFGAAASTLVFN

Candidatus R. andeanae F-LDTANDL-AVTVSADTTLGFITNAAN-NGNSFNFTLG-AGKTLTITG-QGITNAQAAVTNNAQNVVAQFNGGAAIANNDLSGVGTIDLGAAASTLVFN

Rickettsia sp. Bar29 F-LDTANNV-AVTVSADTTLGFITNAAN-NGNFFDLTLG-AGKTLTITG-QGITNAQAAVTKNAQNVVTPFNGGAAIANNDLSGVGTIDWGAADSTLVFN

Rickettsia sp. TwKM01 F-LDTANNL-AVTVSADTTLGFITNAAN-NGNSFDLTLG-AGKTLTITG-QGITNAQAAVTKNAQNVVAQFNGGAAIANNDLSGVGTIDWGAADSTLVFN

R. raoultii F-LDTAHNL-AVTVSADTTLGFITKAAN-NGNSFDLTLG-AGKTLTITG-QGITNAQAAVTKNAQNVVAQFNGGAAIANNDLSGVGTIDLGAAASTLVFN

R. asiatica F---------AVN-GADIAIGSVSGT---AGQLLTVNIV-SGNTLTLNGAPGV---------------------AAFPVNTYTNLGPVNFVDAAAVFKVS

Rickettsia sp. 110 FAVNTKADI-------DIAIGSVSGT---AGQLLTITLA-SGDTLTLNGAPGV---------------------AAFPVNTYTNVGPVDFANNAAVFKVS

Rickettsia sp. mmx79 FAVNTKADI-DI----DIAIGSVSGTAD---QLLTITLA-SGDTLTLNGAPGV---------------------AAFPVNTYTNVGPVDFANNAAVFKVS

Rickettsia sp. 103 F---------AVD-GADIAIGSVSGT---AGQLLTITLA-SGNTLTLNGAPGV---------------------AAFPVNTYTNVGPVNFADAAAVFKVS

Rickettsia sp. J52 F---------AVD-GADIAIGSVSGT---AGQLLTITLA-SGNTLTLNGAPGV---------------------AAFPVNTYTNVGPVNFENDVAVFKVS

Candidatus R. hoogstraalii F-LDTANNL-AVTISEDTTLGFITNDAN-KANFFDFTLG-AGKTLTITG-QGINAGQATVTKNAQNIVVQLNGGNAIANNDLSGVETIDFGAAASTLVFN

Rickettsia sp. California 2 F-LDTANNL-AATVSEDTTLGFITNAAN-NGNFFNFTLG-AGKTLTITG-QGITAGQAAATKNAQNAVAQVNGGNAIANNDLSGVGTIDFGAAPSTLVFN

R. hulinensis F-LNNANNL-AVTVSEDTTLGFINNVAN-NANHFNLTLG-AGKTLTITG-QGITNVQAAATHNAQNIVAQFNGGAAIANNDLSGLGTIDFGAAASTLVFD

**[201-300 ]**

**[ | | | | | | | | | | ]**

R. bellii RML369-C -----------ILGPN------------GV----------------------------------------------------------------------

R. bellii OSU 85-389 -------------AGNTVGVAG---VV-GS-KQLTI------------------------MMMNEASNLILGPN--------------------------

R. prowazekii Madrid E LINPTTQEAPLTLGANSKIVNG---GN-GT-LNITNG---------FIQVSDNTFAGIKTINIDDCQGLMFNST-PDAANTLNL--QVGGNTINFNGIDG

R. prowazekii Rp22 LINPTTQEAPLTLGANSKIVNG---GN-GT-LNITNG---------FIQVSDNTFAGIKTINIDDCQGLMFNST-PDAANTLNL--QVGGNTINFNGIDG

**R. typhi Wilmington LINPTTQEAPLTLGDNAKIVNG---AN-GI-LNITNG---------FVKVSDKTFAGIKTINIGDNQGLMFNTT-PDAANALNL--QGGGNTINFNGRDG**

R. akari Hartford LVNPTTQNAPLILGGNAKIANG---AN-GT-LDVTKG---------FIQVSDKSFATVKTINIGDGQGFIFNTD-ATNGNALNLQ-QVAGSTINFNGTDG

R. australis LANPTTQKAPLILGGNAAIANG---AN-GI-LDVTNG---------FIQVSDKSFATVKTINIGDGQGFIFNTD-ATNGNALNLQ-QVAGSTINFNGTDG

R. felis URRWXCal2 LTNPTTQRAPLILGDNAVIANG---AN-GT-LNVTNG---------FIQVSDETFATIKTINIGDGQGFIFNTD-ATAGNALNL--QVGGATINFNGTDG

R. canadensis McKiel LAANGADGTKTAVFGNTATFNSIVPGGKGH-INIDDG---------NIAIFNGTIGNTNGI-----QGFILGAN-AQVT--LNA-------NAKLDGTVG

R. tamurae LAANGADGTKTAIFGNTATFNGTAAGK-GI-INIDNG---------NIAIFNGTIGNNNGI-----QGLTLTGN-AQAT--LNA-------NTKFDGTVG

R. helvetica LAANGADGTKTAIFGNTATFNGTAAGK-GI-INIDNG---------NIAIFNGTIGNNNGI-----QGLTLTGN-AQAT--LNA-------NTKFDGTVG

REIS ---PADTTNGIILGANAKIANA---ANAGAVLNVTNAGSGIGNQAKGLEVQDNSFVAVKTLNVNDGSLLRFNSN-SDGAGNVNLTLQNGGNVINLQG---

R. monacensis ---PADTTNGIILGANAKIANA---ANAGAVLNVTNAGSGIGNQAKGLEVQDNSFVAVKTLNVNDGSLLRFNSN-SDGAGNVNLTLQNGGNVINLQG---

R. massiliae MTU5 LANPTTQKAPLVLGNNAVIANG---VN-GT-LNVTNG---------FIQVSDKSFATVKAINIGDGQGFIFNTD-ATVGNALNL--QAGGATINFNGTDG

Candidatus R. amblyommii LANPTTQKAPLTLGQNALIVNG---VN-GT-LNVTNG---------FIQVSDKSFATVKAINIGDGQGFIFNTN-ATVGNALNL--QAGGATINFNGTEG

R. rhipicephali LANPTTQKAPLVLGNNTVIANG---VN-GT-LNVTNG---------FIQVSDKSFATVKAINIDDGQGFIFNTD-ATVGNALNL--QAGGATINFNGTDG

R. aeschlimannii LANPTTQKAPLVLGNNAVIANG---VN-GT-LNVTNG---------FIQVSDKSFATVKTINIGDGQGFIFNTD-ATVGNALNL--QAGGATINFNGTDG

R. montanensis LAEPTTQKAPLILGGNAVIANG---VN-GT-LNVTNG---------FIQVSDKSFATVKAINIGDGQGFIFNTNVAAGGNALNL--QVGGATINFNGTDG

R. slovaca 13-B LANPTTQKAPLILGDNALIVNG---AN-GT-LNVTNG---------FIQVSSKSFATVNAINIGDGQGIMFNTD-ANNANALNL--QAGGTTINFNGTDG

R. peacockii Rustic LANPTTQKAPLILGDNAVIVHG---NN-GT-LNVTNG---------FIQVSNKSFATVKAINIGDGQGIMFNTD-ANNANALNL--QAGGTTINFNGTDG

R. rickettsii Iowa LANPTTQKAPLILGDNAVIANG---VN-GT-LNVTNG---------FIQVSNKSFATVKAINIADGQGIIFNTD-ANNANTLNL--QAGGTTINFTGTDG

R. rickettsii Sheila Smith LANPTTQKAPLILGDNAVIANG---VN-GT-LNVTNG---------FIQVSNKSFATVKAINIADGQGIIFNTD-ANNANTLNL--QAGGTTINFTGTDG

R. japonica LANPTTQKAPLILADNALIVNG---AN-GT-LNVTNG---------FIQVSDKSFATVKAINIGDGQGFMFNTN-ATNANALNL--QAGGTTINFNGTDG

R. honei LANPTTQEAPLILGDNTEIVNG---GN-GT-LNVTKG---------FIQVSNKSFATVKAINIADGQGIMFNTD-ANNANALNL--QAGGTTINFNGTDG

R. conorii str. Malish 7 LANPTTQKAPLILGDNAVIVNG---AN-GT-LNVTNG---------FIKVSSKSFATVNVINIGDGQGIMFNTD-ADNVNTLNL--QANGATITFNGTDG

R. sibirica 246 LANPTTQKAPLILGDNAVIVNG---AN-GT-LNVTNG---------FIKVSSKSFATVNAINIGDGQGIIFNTD-ANNANALNL--QAGGTTINFNGTDG

R. africae ESF-5 LANPTTQKAPLILGDNAVIVNG---AN-GT-LNVTNG---------FIKVSSKSFATVNAINIGDGQGIIFNTD-ADNANALNL--QAGGTTINFNGTDG

R. parkeri LANPTTQKAPLILGDNATILNG---AN-GT-LNVTNG---------FIKVSSKSFATVNAINIGDGQGIIFNTD-ANNANALNL--QAGGTTINFNGTDG

R. heilongjiangensis LANPTTQKAPLILADNALIVNG---AN-GT-LNVTNG---------FIQVSDKSFATVKAINIGDGQGFMFNTN-ATNANTLNL--QA-GTTINFNGTDG

Israeli tick typhus LANPTTQNAPLILGDNAVIANG---AN-GT-LNVTNG---------FIKVSSKSFATVNAINIGDGQGIMFNTD-ANNANALNL--QANGATINFNGTDG

Rickettsia sp. A-167 LANPTTQKAPLILGDNAVIANG---AN-GT-LNVTNG---------FITVSSKSFATVNAINIGDGQGIMFNTD-ANNANALNL--QANGATINFNGTDG

Candidatus R. tasmanensis LANPTTQKAPLILGGNA--VNG---VN-GT-LNVTNG---------FIQVSDKSFATVQAINIGDGQGFMFNTG-ATNGNALNL--QAGGATINFNGTDG

Rickettsia sp. IG-1 LANPTTQKAPLILGDNAVIVNG---VN-GT-LNVTNG---------FIQVSNKSFATVKAINIGDGQGIMFNTD-ANNANALNL--QAGGTTINFNGTDG

Candidatus R. barbariae LANPTTQKVPLILGDNAVIVNG---AN-GT-LNVTNG---------FIKVSSKSFATVNAINIGDGQGIIFNTD-ANNANALNL--QAGGAAINFNGTDG

Rickettsia sp. S LANPTTQKAPLTLGDNAVIVNG---AN-GT-LNVTNG---------FIKVSSKSFATVNAINIGDGQGIIFNTD-ADNANALNL--QAGGTTINFNGTDG

Candidatus R. goldwasserii LANPTTQKAPLVLGGDAVVVNG---VN-GT-LNVTNG---------FIQVSDKSFATVKAINIGDGQGIMFNTD-ANNANALNL--QAGGTTINFNGTDG

Rickettsia mongolotimonae LTNPTTQKAPLILGDNAVIVNG---AN-GT-LNVTNG---------FIKVSSKSFATVNAINIGDGQGIIFNTD-ANNANALNL--QAGGTAINFNGTDG

Rickettsia sp. BJ-90 LANPTTQKAPLILGDNAVIVNG---AN-GT-LNVTNG---------FIKVSSKSFATVNAINIGDGQGIIFNTD-ANNANALNL--QAGGTTINFNGTDG

Candidatus R. andeanae LANPTTQKAPLTLGKNAVIVNG---VN-GT-LNVTNG---------FIQVSDKSFATVKAINIGDGQGFIFNTD-PTGGNALNL--QAGGATINFNGTDG

Rickettsia sp. Bar29 LANPTTQKAPLVLGNNAVIANG---VN-GT-LNVTNG---------FIQVSDKSFATVKAINIGDGQGFIFNTD-ATVGNALNL--QAGGATINFNGTDG

Rickettsia sp. TwKM01 LANPTTQKAPLVLGNNAVIVNG---VN-GT-LNVTNG---------FIQVSDKSFATVKAINIGDGQGFIFNTD-ATVGNALNL--QAGGATINFNGTDG

R. raoultii LANPTTQKAPLILGNNAVIANG---VN-GT-LNVTNG---------FIQVSDKSFATVKAINIDDGQGFIFNTD-ATVGNALNL--QAGGATINFNGTDG

R. asiatica LAANGADGTKTAIFGNTATFNGTAAGK-GI-INIDNG---------NIAIFNGTIGNNNGI-----QGLTLTGN-AQAT--LNA-------NTKFDGTVG

Rickettsia sp. 110 LAANGADGTKTAIFGNTATFNGFAPGH-GT-INIDAN---------NIAIFNGTIGNTSGI-----QAITLGAN-AQAT--LNA-------NTKLDGAVG

Rickettsia sp. mmx79 LAANGADGTKTAIFGNTATFNGFAPGH-GT-INIDAN---------NIAIFNGTIGNTSGI-----QAITLGAN-AQAT--LNA-------NTKLDGAVG

Rickettsia sp. 103 LAANGADGTKTAIFGNTATFNGAAPGQ-GT-INIDAN---------NIAIFNGTIGNTNGI-----QAIMLGAN-AQAT--LNA-------NTKLDGAVG

Rickettsia sp. J52 LAANGADGTKTAIFGNTATFNGVEPWR-GT-INIDAN---------NIAIFNGTIGNTNGI-----QAITLGAN-AQAT--LNA-------NTKLCGDVA

Candidatus R. hoogstraalii LTNPTTQKAPLILGDNAVIANG---AN-GT-LNVTNG---------FIQVSDKTFATVKTINIGDGQGFIFNTD-ATNGNVLNL--QAANVTINFNGTDG

Rickettsia sp. California 2 LTNPTTQRAPLILGDNAVIANG---AN-GT-LNVTNG---------FIQVSDETFATIKTINIGDGQGFIFNTD-ATAGNALNL--QVGGATINFNGTDG

R. hulinensis LANPTTQKAPLILADNALIVNG---AN-GT-LNVTNG---------FIQVSDKSFATVKAINIGDGQGFMFNTN-ATNANALNL--QAGGTTINFNGTDG

**[301-400 ]**

**[ | | | | | | | | | | ]**

R. bellii RML369-C --------------AAGNFAV-AGEV-------GSYV-GLLNIN--------------------------------------------------------

R. bellii OSU 85-389 ------------GVAAGNFAV-AGEV-------GSYV-GLLNIN--------------------------------------------------------

R. prowazekii Madrid E TGKLVLVSK---NGAATEFNV-TGTL-------GGNLKGIIELNTA-AVAGKLISQGGAANAVIGTDNGAGRAAG-FIVSVDNGNAATISGQVYAKNMVI

R. prowazekii Rp22 TGKLVLVSK---NGAATEFNV-TGTL-------GGNLKGIIELNTA-AVAGKLISQGGAANAVIGTDNGAGRAAG-FIVSVDNGNAATISGQVYAKNMVI

**R. typhi Wilmington TGKLVLVSK---NGNATEFNV-TGSL-------GGNLKGVIEFDTT-AAAGKLIANGGAANAVIGTDNGAGRAAG-FIVSVDNGNAATISGQVYAKDIVI**

R. akari Hartford TGRLVLLSK---NG-TTDFNVMTGSL-------GGNLKGIIEFNTT-AVAGQLIANAGPANAVIGTNNGAGRAAG-FVVSVDNGNAATVAGQVYAKDIVI

R. australis TGRLVLLSN---NGAATDFNVMTGSL-------GGNLKGIIEFNTTAAVAGQLMANAGPANAVIGTNNGAGRAAG-FVVSVDNGNAATVAGQVYAKNMVI

R. felis URRWXCal2 TGRLVLLSNA-AGGGATDFNV-TGSL-------GGNLKGIIEFNTT-AVAGQLIANAGPANAVIGTNNGAGRAAG-FVVSVANGNAATVAGQVYAKDMVI

R. canadensis McKiel ADGIVL-------GNDAVLNV-ADRV---------NITGITAANNI-NICG------------VAANNGTVNFLGDSTVSTDIGNKNSVAAVNVAGTLIF

R. tamurae AGSVSL-------DNGSILNV-AD---------GVNITGITAVNNI-SIDGK------------AANDGTVNFLGDSAVSTDIGANNAIAAVNVAGELTF

R. helvetica AGSVSL-------DNGSILNV-AD---------GVNITGITAVNNI-SIDGK------------AANDGTVNFLGDSAVSTDIGANNAIAAVNVAGELTF

REIS SGTLHLLAKAGGNGNAANFFVLNGTTLGGGAGDGGDNNGIIHFDTT-AVAGTFGTTAPGLTAVVGINN-ANRAQQ-FIVTAVGGNNATITNQIFAKNVTV

R. monacensis SGTLHLLAKAGGNGNAANFFVLNGTTLGGGAGDGGDNNGIIHFDTT-AVVGTFGTTAPGLTAVVGTNN-ANRAQQ-FIVTAVGGNNATITNQIFAKNVTV

R. massiliae MTU5 TGRLVLLSK---NGAATDFNV-TGSL-------GGNLKGVIAFNTV-AVAGQLIANAGPANAVIGTNNGAGRAAG-FVVSVDNGNAATIAGQVYAKDMVI

Candidatus R. amblyommii TGRLVLLSK---NGAATDFNV-TGSL-------GDNLKGIIEFNTV-AVAGQLIANAGPANAVIGTNNGAGRAAG-FVVSVDNGNAATIAGQVYAKDMVI

R. rhipicephali TGRLVLLSK---NGAATDFNV-TGSL-------GGNLKGIITFNTV-AVAGQLIANAGPANAVIGTNNGAGRAAG-FVVSVDNGNAATIAGQVYAKDMVI

R. aeschlimannii TGRLVLLSK---HGAANDFNV-TGSL-------GGNLKGVIAFNTV-AVAGQLIANAGPANAVIGTNNGAGRAAG-FVVSVDNGNAATIAGQVYAKDMVI

R. montanensis TGRLVLLSK---NGAATDFNV-TGSL-------GGNLKGIIEFNTV-AVAGQLIANAGPANAVIGTNNDAGRAAG-FVVSVGNGNAATITGQVYAKDMVI

R. slovaca 13-B TGRLVLLSK---NAAATDFNV-TGSL-------GGNLKGIIEFNTV-AVDGQLKANAGPANAVIGTNNGAGRAAG-FVVSVDNGKVATIDGQVYAKDMVI

R. peacockii Rustic TGRLVLLSK---NAAATNFNV-TGSL-------GGNLKGIIEFNTV-AVDGQLIANAGPANAVIGTNNGAGRAAG-FIVSVDNGKVATIDGQVYAKDMVI

R. rickettsii Iowa TGRLVLLSK---HAAATNFNI-TGSL-------GGNLKGVIEFNTV-AVDGQLTANAGAANAVIGTNNGAGRAAG-FVVSVDNGKVATIDGQVYAKDMVI

R. rickettsii Sheila Smith TGRLVLLSK---HAAATNFNI-TGSL-------GGNLKGVIEFNTV-AVDGQLTANAGAANAVIGTNNGAGRAAG-FVVSVDNGKVATIDGQVYAKDMVI

R. japonica TGRLVLLSK---NGAATDFNV-TGSL-------GGNLKGIIELNTV-AINGQLIANAGPANAVIGTNNGAGRAAG-FVVSVDNGKAATIDGQVYAKDMVI

R. honei TGRLVLLSK---NAAATNFNV-TGSL-------GDNLKGIIEFNTV-AVDGQLIANAGPANAVIGTNNGAGRAAG-FVVSVDNGKVATIDGQVYAKDMVI

R. conorii str. Malish 7 TGRLVLLSK---NAAATDFNV-TGSL-------GGNLKGIIEFNTV-AVNGQLKANAGANAAVIGTNNGAGRAAG-FVVSVDNGKVATIDGQVYAKDMVI

R. sibirica 246 TGRLVLVSK---NAAATDFNV-TGSL-------GGNLKGIIEFNTV-AVDGQLKANAGPANAVIGTNNGAGRAAG-FVVSVDNGKVATIDGQVYAKDMVI

R. africae ESF-5 TGRLVLLSK---NAAATDFNV-TGSL-------GGNLKGIIEFNTV-AVDGQLKANAGPANAVIGTNNGAGRAAG-FVVSVDNGKVATIDGQVYAKDMVI

R. parkeri TGRLVLLSK---NAAATDFNV-TGSL-------GGNLKGIIEFNTV-AVDGQLKANAGPANAVIGTNNGAGRAAG-FVVSVDNGKVATIDGQVYAKDMVI

R. heilongjiangensis TGRLVLLSK---NGAATDFNV-TGSL-------GGNLKGIIELNTV-ATNGQLIANAGPANAVIGTNNGAGRAAG-FVVSVDNGKAATINGQVYAKDMVI

Israeli tick typhus TGRLVLLSK---NAAATDFNV-TGSL-------GGNLKGIIEFNTV-AVNGQLKANAGPANAVIGTNNGAGRAAG-FVVSVDNGKVATIDGQVYAKDMVI

Rickettsia sp. A-167 TGRLVLLSK---NAAATDFNV-TGSL-------GGNLKGIIEFNTV-AVNGQLKANAGPANAVIGTNNGAGRAAG-FVVSVDNGKVATIDGQVYAKDMVI

Candidatus R. tasmanensis TGRLVLLSK---NGAATDFNV-TGSL-------GGNLKGIIEFNTV-AVGGRLIANAGPANAVIGTNNGAGRAAG-FVVSVDNGKAATIAGQVYAKDMVI

Rickettsia sp. IG-1 TGRLVLLSK---NAAATDFNV-TGSL-------GGNLKGIIEFNTV-AVGGNLIANAGPANAVIGTNNGAGRAAG-FVVSVDNGKVATIAGQVYAKDMII

Candidatus R. barbariae TGRLVLLSK---NAAATDFNV-TGSL-------GGNLKGIIEFNTV-AVDGQLKANAGPANAVIGTNNGAGRAAG-FVVSVDNGKVATIDGQVYAKDMVI

Rickettsia sp. S TGRLVLLSK---NAAATDFNV-TGSL-------GGNLKGIIEFNTV-AVDGQLKANAGPANAVIGTNNGAGRAAG-FVVSVDNGKVATIDGQVYAKDMVI

Candidatus R. goldwasserii TGRLVLLSK---NAAATDFNV-TGSL-------GGNLKGIIEFNTA-VVNGQLIANAGPANAVIGTNNGAGRAAG-FVVSVDNGKVATIAGQVYAKDMVI

Rickettsia mongolotimonae TGRLVLVSK---NAAATDFNV-TGSL-------GGNLKGIIEFNTV-AVDGQLKANAGPANAVIGTNNGAGRAAG-FVVSVDNGKVATIDGQVYAKDMVI

Rickettsia sp. BJ-90 TGRLVLVSK---NAAATDFNV-TGSL-------GGNLKGIIEFNTV-AVDGQLKANAGPANAVIGTNNGAGRAAG-FVVSVDNGKVATIDGQVYAKDMVI

Candidatus R. andeanae TGRLVLLSK---NGAATDFNV-TGSL-------GGNLKGIIEFNTV-AVAGQLIANAGPANAVIGTNNGAGRAAG-FVVSVDNGNAATIAGQVYAQDMVI

Rickettsia sp. Bar29 TGRLVLLSK---NGAATDFNV-TGSL-------GGNLKGIIAFNTV-AVAGQLIANAGPANAVIGTNNGAGRAAG-FVVSVDNGNAATIAGQVYAKDMVI

Rickettsia sp. TwKM01 TGRLVLLSK---NGAATDFNV-TGSL-------GGNLKGIIAFNTV-AVAGQLIANAGPANAVIGTNNGAGRAAG-FVVSVDNGNAATIAGQVYAKDMVI

R. raoultii TGRLVLLSK---NGAATDFNV-TGSL-------GGNLKGIIAFNTV-AVAGQLIANAGPANAVIGTNNGAGRAAG-FVVSVDNGNAATIAGQVYAKDMVI

R. asiatica AGSVSL-------DNGSILNV-AD---------GVNITGITAVNNI-SIDGK------------AANNGTVNFLGDSAVSTDIGANNAIAAVNVAGELTF

Rickettsia sp. 110 AGGITL-------GNAAILNV-AD---------GVNITGITANHNI-SIDGA------------GVNNGTVNFLGDSTVSTDIGANESVAAVNVASELTF

Rickettsia sp. mmx79 AGGITL-------GNAAILNV-AD---------GVNITGITANHNI-SIDGA------------GVNNGTVNFLGDSTVSTDIGANESVAAVNVASELTF

Rickettsia sp. 103 AGGITL-------GNAAILNV-AD---------GVNITGITANANI-SIDGA------------GVNNGTVNFLGDSTVSTDIGANKSVAAVDVASELTF

Rickettsia sp. J52 DGITL--------GNAAILNV-AD---------GVNITGI----TV-SIDGA------------GVNNGTVNFLGDSTVSTDIGANKSVAAVNVASELTF

Candidatus R. hoogstraalii TGRLVLLSK---NGAATDFNV-TGSL-------GGNPKGIIEFNTT-AVAGQLIANAGPASAVIGTNNGAGRAAG-FVVSVANGNAATVAGQVYAKDMVI

Rickettsia sp. California 2 TGRLVLLSNA-AGGGATDFNV-TGSL-------GGNLKGIIEFNTT-AVAGQLIANAGPANAVIGTNNGAGRAAG-FVVSVANGNAATVAGQVYAKDMVI

R. hulinensis TGRLVLLSK---NGAATDFNV-TGSL-------GGNLKGIIELNTV-AINGQLIANAGPANAVIGTNNGAGRAAG-FVVSVDNGKAATIDGQVYAKDMVI

**[401-500 ]**

**[ | | | | | | | | | | ]**

R. bellii RML369-C --------------------------FSNTNSNVSIE---------------------------TDGFTSD----------VTFISNGGVN---------

R. bellii OSU 85-389 --------------------------FSNTNSNVSIE---------------------------TDGFTSD----------VTFISNGGVN---------

R. prowazekii Madrid E QSAN--AGGQVTFEHIVDVGLGGTTNFKTADSKVIITENSNFGSTNFGNLDTQIVV--PDTKILKGNFIGDVKNNGNTAGVITFNANGALVSASTDPNIA

R. prowazekii Rp22 QSAN--AGGQVTFEHIVDVGLGGTTNFKTADSKVIITENSNFGSTNFGNLDTQIVV--PDTKILKGNFIGDVKNNGNTAGVITFNANGALVSASTDPNIA

**R. typhi Wilmington QSAN--AGGQVTFEHLVDVGLGGKTNFKTADSKVIITENASFGSTDFGNLAVQIVV--PNNKILTGNFIGDAKNNGNTAGVITFNANGTLVSGNTDPNIV**

R. akari Hartford QSAN--AGGQVNFNHIVDVGIDGTTTFKTADSKVIIAENSNLGATDFCNLGVQITV--PDTKTLTGNFTGDASNKGNTAGVITFAANGTLASGNSDANVA

R. australis QSAN--AGGQVNFNHIVDVGVDGTTAFKTAASKVAITQNSNFGATDFCNLAVQITV--PDNKTLTGNFTGDASNKGNTAGVITFAANGTLASGNADANVA

R. felis URRWXCal2 QSTN--AGGQVNFGHIVDVGTDGTTAFKTAATTVAITQNSNFGAVDFGNTASQITV--PDTKVLTGNFTGDASNNGNTAGVITFAANGTLASGNADANVA

R. canadensis McKiel QGGNNGNGSNFAAQAINLTSADSFIKFTTQDHQVAGNILNTSGTNNQGGFSVN-----GGDVTVAGNVGAN----GNSLATINFETDNTLVVHKADTNAI

R. tamurae QGGNNGGGGTFTAQAINLTSAGSLIKFTTQDHAVTGNILNTSGTNNQGGFSVN-----GGDVTVTGNIGAD----GNSLATINFETDDSLIVHKAAINAT

R. helvetica QGGNNGGGGTFTAQAINLTSAGSLIKFTTQDHAVTGNILNTSGTNNQGGFSVN-----GGDVTVTGNIGAD----GNSLATINFETDDSLIVHKAAINAT

REIS N------GGTSTFQQQVDVGNGGSTTFK-AASTTTMTISSTLGAVDFGNTASQIIVGAANAPTLTGNFTGDASNAGNTAGVITFLGAGTLNS--ADANTP

R. monacensis ------NGGTSTFQQQVDVGNGGSTTFK-AASTTTMTISSTLGAVDFGNTASQIIVAAANAPTLTGNFTGDASNAGNTAGVITFLGAGTLNS--ADANTP

R. massiliae MTU5 QSAN--AGGQVNFGHIVNVGTDGTTAFKTAASKVAITQNSNFGTTDFGNLAAQITV--PNTMTLTGNFTGDASNPGNTAGVITFAANGTLASASADANVA

Candidatus R. amblyommii QSAN--AGGQVNFGHIVDVGTDGTTAFKTAASKVAITQNSNFGTTDFGNLAAQITV--PDTMTLTGNFTGDASNPGNTAGVITFAANGTLASASADANVA

R. rhipicephali QSAN--AGGQVNFGHIVDVGTDGTTAFKTAASKVAITQNSNFGTTDFGNLAAQITV--PDTMTLTGNFTGDASNPGNTAGVITFAANGTLASASADANVA

R. aeschlimannii QSAN--AGGEVNFGHIVDVGTDGTTAFKTEASKVAITQNSNFGTTDFGNLAAQITV--PDTMTLTGNFTGDASNHGNTAGVITFAANGTLASASADANVA

R. montanensis QSAN--AGGQVNFGHIVDVGTDGTTAFKTAASKVAITQNSNFGTTDFGNLAAQITV--PDTMTLTGNFTGNASNPENTAGVITFAANGTLASASADANVA

R. slovaca 13-B QSAN--AAGQVNFRHIVDVGTDGTTAFKTAASKVAITQNSNFGNTDFGNLAAQITV--PNTMTLTGNFTGDASNPGNTAGVITFDANGTLASASADANVA

R. peacockii Rustic QSAN--AAGQVNFRHIVDVGTDGTTAFKTAASKVTITQNSNFGNTDFGNLAAQIKV--PNAMTLTGNFTGDASNPGNTAGVITFDANGTLESASADANVA

R. rickettsii Iowa QSAN--ATGQVNFRHIVDVGADGTTAFKTAASKVTITQDSNFGNTDFGNLAAQIKV--PNAITLTGNFTGDASNPGNTAGVITFDANGTLESASADANVA

R. rickettsii Sheila Smith QSAN--ATGQVNFRHIVDVGADGTTAFKTAASKVTITQDSNFGNTDFGNLAAQIKV--PNAITLTGNFTGDASNPGNTAGVITFDANGTLESASADANVA

R. japonica QSAN--ANGQVNFRHIVDVGIDGTTAFKTAASIVAITQNSNFGTTDFGNLAAQVTV--PDTMTLTGNFTGDANNPGNTAGVITFAANGTLASASADANVA

R. honei QSVN--AAGQVNFRHIVDVGTDGTTAFKTAASKVTITENSNFGTTDFGNLAAQIEV--PNNMTLTGNFTGDARNPGNTAGVITFNANGILASASADANVA

R. conorii str. Malish 7 QSAN--AVGQVNFRHIVDVGTDGTTAFKTAASKVAITQNSNFGTTDFGNLAAQIIV--PNTMTLNGNFTGDASNPGNTAGVITFDANGTLASASADANVA

R. sibirica 246 QSAN--AVGQVNFRHIVDVGTDGTTAFKTAASKVAITQNSNFGTTDFGNLAAQITV--PNTMTLKGNFTGDASNPGNTAGVITFDANGTLASASADANVA

R. africae ESF-5 QSAN--AVGQVNFRHIVDVGTDGTTAFKTAASKVAITQNSNFGTTDFGNLAAQITV--PNTMTLKGNFTGDASNPGNTAGVITFDANGTLASASADANVA

R. parkeri QSAN--AVGQVNFRHIVDVGTDGTTAFKTAASKVAITQNSNFGTTDFGNLAAQITV--PDAMTLKGNFTGDASNPGNTAGVITFDANGTLASASADANVA

R. heilongjiangensis QSAN--ANGPVNFRHIVDVGIDGTTAFKTAASKVAITQNSNFGTTDFGNLAAQITV--PDTMTLTGNFTGDANNPGNTAGVITFDANGTLASASADANVA

Israeli tick typhus QSAN--AVGQVNFRHIVDVGTDGTTAFKTAASKVAITQNSNFGTTDFGNLAAQITV--PNTMTLKGNFTGDASNPGNTAGVITFDANGTLASASADANVA

Rickettsia sp. A-167 QSAN--AVGQVNFRHIVDVGTDGTTAFKTAASKVAITQNSNFGTTDFGNLAAQITV--PNTMTLKGNFTGDASNPGNTAGVITFDANGTLASASADANVA

Candidatus R. tasmanensis QSAN--AGGQVNFGHIVDVGTDGTTAFKTAASKVAITQNSNFGATDFGNLAAQITV--PDTMTLTGNFTGDASNPGNTAGVITFDANGTLASASADANVA

Rickettsia sp. IG-1 QSAN--AAGQVNFGHIVDVGIDGTTAFKTAASKVAITQNSNFGTTDFGNLAAQINV--PNTMTLTGNFTGDASNPGNTAGVITFDANGTLASASADANVA

Candidatus R. barbariae QSAN--AVGQVNFRHIVDVGIDGTTAFKTAASKVAITQNSNFGTTDFGNLAAQITI--PNTMTLKGNFTGDANNPGNTAGVITFDANGTLASASADANVA

Rickettsia sp. S QSAN--AVGQVNFRHIVDVGTDGTTAFKTAASKVAITQNSNFGTTDFGNLAAQITV--PNTMTLKGNFTGDASNPDNTAGVITFDANGTLASASADANVA

Candidatus R. goldwasserii QSAN--ADGQVNFGHIVDVGTDGTTAFKTAASKVAITQNSNFGNTDFGNLAVQIKV--PDTMTLTGNFTGDASNPGNTAGVITFNANGTLASASADANVA

Rickettsia mongolotimonae QSAN--AVGQVNFRHIVDVGTDGTTAFKTAASKVAITQNSNFGTTDFGNLAAQITV--PNTMTLKGNFTGDASNPGNTAGVITFDANGTLASASADANVA

Rickettsia sp. BJ-90 QSAN--AVGQVNFRHIVDVGTDGTTAFKTAASKVAITQNSNFGTTDFGNLAAQITV--PNTMTLKGNFTGDASNPGNTAGVITFDANGTLASASADANVA

Candidatus R. andeanae QSAN--VGGQVTFGHIVDVGTDGTTAFKTAASKVAITQNSNFGTTDFGNLAAQITV--PDTMILTGNFTGDARNPGNTAGVITFAANGTLASASADANVA

Rickettsia sp. Bar29 QSAN--AGGQVNFGHIVDVGTDGTTAFKTAASKVAITQNSNFGTTDFGNLAAQITV--PNTMTLTGNFTGDASNPGNTAGVITFAANGTLASASADANVA

Rickettsia sp. TwKM01 QSAN--AGGQVNFRHIVDVGTDGTTAFKTAASKVAITQNSNFGTTDFGNLAAQITV--PDTMTLTGNFTGDASNPGNTAGVITFAANGTLASASADANVA

R. raoultii QSAN--AGGQVNFGHIVDVGTDGTTAFKTAASKVAITQNSNFGTTDFGNLAAQITV--PDTMTLTGNFTGDANNPGNTAGVITFAANGTLASASADANVA

R. asiatica QGGNNGGGGTFTAQAINLTSAGSLIKFTTQDHAVTGNILNTSGTNNQGGFSVN-----GGDVTVTGNIGAD----GNSLATINFETDDSLIVHKAAINAT

Rickettsia sp. 110 QGGNDDNGSTFTAQAINLTGADSLIKFTTQDHTVTGNILNTSGTNNQGGFSVN-----GADVTVTGNIGAN----GNSLATINFETDYVLTVHKAAANAM

Rickettsia sp. mmx79 QGGHDDNGSTFTAQAINLTGADSLIKFTTQDHTVTGNILNTSGTNNQGGFSVN-----GADVTVTGNIGAN----GNSLATINFETDYVLTVHKAAANAT

Rickettsia sp. 103 QGGN---GGTFTAQAINLTGADSLIKFTTRAH--------------------------------------------------------------------

Rickettsia sp. J52 QGGNNVNGSTFSAQAINLTGADSLIKFIRQDHKVTGNILNTSGTNNQGGFSVN-----GADLTVTGNIGAN----GNSLATINFETDNVLVVHKAAANAT

Candidatus R. hoogstraalii QSTN--AGGQVNFDHIVDVGTDGTTAFKTAASKVAITQNSNFGATDFGNLAVQITV--PNTKTLTGNFTGDASNNGNTAGVITFAANGTLASGNADANVA

Rickettsia sp. California 2 QSTN--AGGQVNFGHIVDVGTDGTTAFKTAATTVAITQNSNFGAVDFGNTASQITV--PDTKVLTGNFTGDASNNGNTAGVITFAANGTLASGNADANVA

R. hulinensis QSAN--ANGQVNFRHIVDVGIDGTTAFKTAASKVAITQNSNFGTTDFGNLAAQITV--PDTMTLTGNFTGDANNPGNTAGVITFDANGTLASASADANVA

**[501-600 ]**

**[ | | | | | | | | | | ]**

R. bellii RML369-C ------------------------------------------------------------------------GAITLNAKGITLNGNITN----------

R. bellii OSU 85-389 ------------------------------------------------------------------------GAITLNAKGITLNGNITN----------

R. prowazekii Madrid E VTN-INAIE--AEGAGVVELSGIHIA---------------------------ELRLGNG------------GSIFKLADGTVINGPVNQNALMNNNALA

R. prowazekii Rp22 VTN-INAIE--AEGAGVVELSGIHIA---------------------------ELRLGNG------------GSIFKLADGTVINGPVNQNALMNNNALA

**R. typhi Wilmington VTN-IKAIE--VEGAGIVQLSGIHGA---------------------------ELRLGNA------------GSIFKLADGTVINGPVNQNPLVNNNALA**

R. akari Hartford VTNNIKAIE--VAGAGVVQLSGTHTA---------------------------ELSLGNA------------GSVFKLADDTVINGKVNENALIG-GAVA

R. australis VTNDIKAIE--AAGVGVVQLSGIHTA---------------------------ELRLGNA------------GSVFKLADGTVISGQVNQTALIG-GALA

R. felis URRWXCal2 VTNKITAIE--AAGVGVVQLSGTHTA---------------------------ELRLGNA------------GSQFKLADGTIINGNVNQTVLVGNAALA

R. canadensis McKiel FVQNVDTA---TANTGILKLQGANFAINGNIGGNNALKIVNLEDNDNGAATNFTLKQGSKINAQNIYLSSAFDNSLTLEEGTTVTGNIINKLAAG-----

R. tamurae NALFIQNVNTATANTGILKLHGTNYAINGNIGANNALKLVDLADDDNGAATNFTLKQGSSIKAQNISLADIQDNTLTLEEGTTITGDIINTKGNG-----

R. helvetica NALFIQNVNTATANTGILKLHGTNYAINGNIGANNALKLVDLADDDNGAATNFTLKQGSSIKAQNISLADIQDNTLTLEEGTTITGDIINTKGNG-----

REIS VTNKITAIE--ASAAGIVNLSGTHTA---------------------------ELRLGNA------------GSIFKLADGTVITGNVNQTVLIG-DALA

R. monacensis VTNKITAIE--ASAAGIVNLSGTHTA---------------------------ELRLGNA------------GSIFKLADGTVITGNVNQTALIG-GALA

R. massiliae MTU5 VTNNITAIE--ASGVGVVQLSGTHTA---------------------------ELRLGNA------------GSVFQLADGTVINGKVNQTAVVG-GALA

Candidatus R. amblyommii VTNNITAIE--ALGVGVVQLSGTHTA---------------------------ELRLGNA------------GSVFKLADGTVINGKVNQTAVVG-GAFA

R. rhipicephali VTNNITAIE--ASGIGVVQLSGTHTA---------------------------ELRLGNA------------GSVFKLADGTVINGKVNQTAVVG-GALA

R. aeschlimannii VTNNITAIE--ASGIGVVQLSGTHAA---------------------------ELRLGNA------------GSVFKLADGTVINGKVNQTAVVG-GALA

R. montanensis VTNNITAIE--ASGVGVVQLSGTHTA---------------------------ELRLGNA------------GSVFKLADGTVINGKVNQTAVVG-GALA

R. slovaca 13-B VTNNITAIE--ASGAGVVQLSGTHAA---------------------------ELRLGNA------------GSIFKLADGTVINGKVNQTALVA-GVLA

R. peacockii Rustic VTNNITAIE--ASGAGVVQLSGTHAA---------------------------ELRLGNA------------GSIFKLADGTVINGKVNQTALVG-GVLA

R. rickettsii Iowa VTNNITAIE--ASGAGVVQLSGTHAA---------------------------ELRLGNA------------GSIFKLADGTVINGKVNQTALVG-GALA

R. rickettsii Sheila Smith VTNNITAIE--ASGAGVVQLSGTHAA---------------------------ELRLGNA------------GSIFKLADGTVINGKVNQTALVG-GALA

R. japonica VTNNITAIE--ASGVGVVQLSGTHTA---------------------------ELRLGNA------------GSVFKLADGTVINGKVNQTVLVG-GVLA

R. honei VTNNITAIE--ASGAGVVELSGTHAA---------------------------ELRLGNA------------GSVFKLADGTVINGKVNQTAPVG-GALA

R. conorii str. Malish 7 VTNNITAIE--ASGAGVVQLSGTHAA---------------------------ELRLGNA------------GSVFKLADGTVINGKVNQTALVG-GALA

R. sibirica 246 VTNNITAIE--ASGAGVVQLSGTHAA---------------------------ELRLGNA------------GSVFKLADGTVINGKVNQTALVA-GALA

R. africae ESF-5 VTNNITAIE--ASGAGVVQLSGTHAA---------------------------ELRLGNA------------GSVFKLADGTVINGKVNQTALVA-GALA

R. parkeri VTNNITAIE--ASGAGVVQLSGTHAA---------------------------ELRLGNA------------GSVFKLADGTVINGKVNQTALVA-GALA

R. heilongjiangensis VTNNITAIE--ASGVGVVQLSGTHTA---------------------------ELRLGNA------------GSVFKLADGTVINGKVNQTPLVG-GVLA

Israeli tick typhus VTNNITAIE--ASGAGVVQLSGTHAA---------------------------ELRLGNV------------GSVFKLADGTVINGKVNQTVLVA-GALA

Rickettsia sp. A-167 VTNNITAIE--ASGAGVVQLSGTHAA---------------------------ELRLGNV------------GSVFKLADGTVINGKVNQTALVA-GALA

Candidatus R. tasmanensis VTNSITAIE--ASGVGVVQLSGTHTA---------------------------ELRLGNA------------GSVFKLADGTVINGKVNQTAVVG-GALA

Rickettsia sp. IG-1 VTNNITAIE--ASGAGVVQLSGTHAA---------------------------ELRLGNA------------GSVFKLADGTVIKGQVNQTALVG-GVLA

Candidatus R. barbariae VTNNITAIE--ASGAGVVQLSGTHAA---------------------------ELRLGNA------------GSVFKLADGTVINGKVNQTALVA-GALA

Rickettsia sp. S VTNNITAIE--ASGAGVVELSGTHAA---------------------------ELRLGNA------------GSVFKLADGTVINGKVNQTALVA-GALA

Candidatus R. goldwasserii VTNNITAIE--AAGVGVVQLSGTHAA---------------------------ELRLGNV------------GSVFKLADGTVINGKVNQTALVG-GALA

Rickettsia mongolotimonae VTNNITAIE--ASGAGVVQLSGTHAA---------------------------ELRLGNA------------GSVFKLADGTVINGKVNQTALVA-GALA

Rickettsia sp. BJ-90 VTNNITAIE--ASGAGVVQLSGTHAA---------------------------ELRLGNA------------GSVFKLADGTVINGKVNQTALVA-GALA

Candidatus R. andeanae VTNNITAIE--ASGIGIVQLSGTHTA---------------------------ELRLGNA------------GSIFKLADGTVINGKVNQTAVVG-GAFA

Rickettsia sp. Bar29 VTNNITAIE--ASGVGVVQLSGTHTA---------------------------ELRLGNA------------GSVFKLADGTVINGKVNQTAVVG-GALA

Rickettsia sp. TwKM01 VTNNITAIE--ASGIGVVQLLGTHTA---------------------------ELRLGNA------------GSVFKLADGTVINGKVNQTAVVG-GALA

R. raoultii VTNNITAIE--ASGIGVVQLSGTHTA---------------------------ELRLGNA------------GSVFKLADGTVINGTVNQTAVVG-GAFA

R. asiatica NALFIQNVNTATANTGILKLHGTNYAINGNIGANNALKLVDLADDDNGAATNFTLKQGSSIKAQNISLADIQDNTLTLEEGTTITGDIINTKGNG-----

Rickettsia sp. 110 NALFVQNVNTAAPNTGTLKLHGTAFAINGNIGGNNALKLVALENDDNDGATNFTLKQGNSIKAQNIRLADVQDNTLTLEEGTTITGDIVNIKGAG-----

Rickettsia sp. mmx79 NALFVQNVNTAAPNTGTLKLHGTAFAINGNIGGNNALKLVALENDDNDGATNFTLKQGNSIKAQNIRLADVQDNTLTLEEGTTITGDIVNIKGAG-----

Rickettsia sp. 103 ---------------------------------------------------------------------------------STVTG--------------

Rickettsia sp. J52 NALFVQNVNTAAPNTGILKLHGTAFAINGNIGGNNALKSVFLENDYNDGATNFTLKQGNSIKAQNIYLASVQDNTLTLEEGTTITGDIVNTKRAG-----

Candidatus R. hoogstraalii VTNNIKAIE--AAGVGVVQLSGTHTA---------------------------ELRLGNA------------GSVFKLADGTVINGKVNQTALIG-GALA

Rickettsia sp. California 2 VTNKITAIE--AAGVGVVQLSGTHTA---------------------------ELRLGNA------------GSQFKLADGTIINGNVNQTVLVGNAALA

R. hulinensis VTNNITAIE--ASGVGVVQLSGTHTA---------------------------ELRLGNA------------GSVFKLADGTVINGKVNQTVLVG-GVLA

**[601-700 ]**

**[ | | | | | | | | | | ]**

R. bellii RML369-C -----------------------------LKAIDL---NSNNLTFGGNNIQLLAN-GSAITFRAN-EQLTLTSASD--IEINSNIAVTNDQQGSIDASGL

R. bellii OSU 85-389 -----------------------------LKAIDL---NSNNLTFGGNNIQLLAN-GSAITFRAN-EQLTLTSASD--IEINSNIAVTNDQQGSIDASGL

R. prowazekii Madrid E AGSIQLDGSAIITGDIG----NGGV-NAALQHITLANDASKILALDGANI-IGANVGGAIHFQANGGTIKLTNTQNNIV-VNFDLDITTDKTGVVDASSL

R. prowazekii Rp22 AGSIQLDGSAIITGDIG----NGGV-NAALQHITLANDASKILALDGANI-IGANVGGAIHFQANGGTIKLTNTQNNIV-VNFDLDITTDKTGVVDASSL

**R. typhi Wilmington AGSIQLDGSAIITGDIG----NGAV-NAALQDITLANDASKILTLSGANI-IGANAGGAIHFQANGGTIQLTSTQNNIL-VDFDLDVTTDQTGVVDASSL**

R. akari Hartford GGTIQLDGSATITGDIG----NGAG-NAALHGITLADDASKTLTLGGAKI-IGVNAGGTINFQANGGTIKLTSTQNDIL-VNFNLAIAADKTGVVDASGL

R. australis GGAIQLDGSATITGDIG----NGGG-NAALQGITLANDASKTLTLGGANI-IGANAGRTIDFQANGGTIKLTSTQNDIL-VNFDLAITTDKTGVVDASSL

R. felis URRWXCal2 NGAIQLDGSATITGDIG----NGAGNAAPIQGITLANDASKTLTLGGANI-IGANAGGTIDFQANGGTVKLTSTQNNIL-VDFDLAITTDKTGVVDASSL

R. canadensis McKiel NGTIALTGNAAINGNIGVDAAIGGNVGNAIKNINL---AGNNLSYGGSSILLVNNPANAITFGAN-ETLTLTNTTDAALQIKANLAITTDKQGILDASSL

R. tamurae NGTIVLTGNATINGGIGVDIAAGGNLGKTIKNINL---GGKDLSYGGNTVKVQSAPNNGINFAAN-ETLSLTNTTDPIL-IASDI-ITTDKQGIIDGSAL

R. helvetica NGTIVLTGNATINGGIGVDIAAGGNLGKTIKNINL---GGKDLSYGGNTVKVQSAPNNGINFAAN-ETLSLTNTTDPIL-IASDI-ITTDKQGIIDGSAL

REIS GGAIQLDGSATITGDIG----NGGG-NAVLQGITLADDASKTLTLGGANI-IGANVGGMIDFQANGGTIKLTSTQNNIL-VGFNLAINTDQTGVVDASSL

R. monacensis GGAIQLDGSATITGDIG----NGGG-NAVLQGITLADDASKTLTLGGANI-IGANVGGMIDFQANGGTIKLTSTQNNIL-VGFNLAINTDQTGVVDASSL

R. massiliae MTU5 AGAITLDGSATITGDIG----NGGG-AAALQGITLANDATKTLTLGGANI-IGSNVGGTIDFQANGGTIKLTSTQNNIL-VDFDLDITTDQTGVVDASSL

Candidatus R. amblyommii AGAITLDGSATITGDIG----NGGG-AAALRGITLANDAKKTLTLDGANI-IGANVGGTIDFQANGGTLKLTSTQNNIL-VDFDLAITTDQTGVVDANSL

R. rhipicephali AGAITLDGSATITGDIG----NGGG-AAALQGITLANDATKTLTLGGANI-IGAV-GGTIDFQANGGTIKLTSTQNNIL-VDFDLDITTDQTGVVDASSL

R. aeschlimannii AGAITLDGSATITGDIG----NGGG-VAALQGITLANDATKTLTLGGANI-IGANVGGTIDFQANGGTIKLTNTQNNIL-VDFDLAIATDQTGVVDASSL

R. montanensis AGAITLDGSATITGDIG----NGGG-AAALQGITLANDATKTLTLGGANI-IGAN-GGTINFQANGGTIKLTSTQNNIL-VDFDLAINTDQTGVVDASSL

R. slovaca 13-B AGAITLDGSATITGDIG----NAGG-AAALQGITLANDATKTLTLGGANI-IGAN-GGTIDFQANGGTIKLTSTQNNIV-VDFDLAITTDQTGVVDASSL

R. peacockii Rustic AGTITLDGSATITGDIG----NAGG-AAALQGITLANDATKTLTLGGANI-IGAN-GGTIDFQANGGTIKLTSTQNNIV-VDVDLAIATDQTGVVDASSL

R. rickettsii Iowa AGTITLDGSATITGDIG----NAGG-AAALQRITLANDAKKTLTLGGANI-IGAG-GGTIDLQANGGTIKLTSTQNNIV-VDFDLAIATDQTGVVDASSL

R. rickettsii Sheila Smith AGTITLDGSATITGDIG----NAGG-AAALQRITLANDAKKTLTLGGANI-IGAG-GGTIDLQANGGTIKLTSTQNNIV-VDFDLAIATDQTGVVDASSL

R. japonica AGAITLDGSATITGDIG----NGGG-GAALQSITLANDATKTLTLGGANI-ISAN-GGTINFQANGGTIKLTSTQNNIV-VDCDLAIATDQTGVVDASSL

R. honei AGAITLDGSATITGDIG----NAGG-AAALQGITLANDATKTLTLGGANI-IGAN-GGTIAFQANGGTIKLTSTQNNIV-VAFDLAITTDQTGVVDASSL

R. conorii str. Malish 7 AGTITLDGSATITGDIG----NAGG-AAALQGITLANDATKTLTLGGANI-IGAN-GGTINFQANGGTIKLTSTQNNIV-VDFDLAIATDQTGVVDASSL

R. sibirica 246 AGTITLDGSATITGDIG----NAGG-AAALQGITLANDATKTLTLGGANI-IGAN-GGTIDFQANGGTIKFTSTQDNIV-VHFDLDIITDQTGVVDASSL

R. africae ESF-5 AGTITLDGSATITGDIG----NAGG-AAALQGITLANDATKTLTLGGANI-IGAN-GGMIDFQANGGTIKFTSTQNNII-VDFDLAITTDQTGVVDASSL

R. parkeri AGTITLDGSATITGDIG----NAGG-AAALQGITLANDATKTLTLGGANI-IGAN-GGTIDFQANGGTIKFTSTQNNIV-VDFDLAIITDQTGVVDASSL

R. heilongjiangensis AGAITLDGSATITGDIG----NGGG-GAALQGITLANDATKTLTLGGANI-IGAN-GGTINFQANGGTIKLTSTQNNIV-VDCDLAIATDQTGVVDASSL

Israeli tick typhus AGTITLDGSATITGDIG----NAGG-AAALQRITLANDATKTLTLGGANI-IGAN-GGTIDFQANGGTIKLTSTQNNIV-VDFDLAITTDQTGVVDASSL

Rickettsia sp. A-167 AGTITLDGSATITGDIG----NAGG-AAALQGITLANDATKTLTLGGANI-IGAN-GGTIDFQANGGTIKLTSTQNNIV-VDFDLAITTDQTGVVDASSL

Candidatus R. tasmanensis AGAITLDGSATITGDIG----NGGG-AAALQGITLANDATKTLTLGGANI-IGANVGGAIDFQANGGTIKLTSTQNNIV-VDFDLAITTDQTGVVDASSL

Rickettsia sp. IG-1 AGAITLDGSATITGDIG----NAGG-AAALQGITLANDATKTLTLGGANI-IGAN-GGTIDFQANGGTIKLTSTQNNIV-VDFDLAITTDQTGVVDASSL

Candidatus R. barbariae AGTITLDGSATITGDIG----NAGG-AAALQGITLANDAAKTLTLGGANI-IGAN-GGTIDFQANGGTIKFTSTQNNIV-VDFDLAITTDQTGVVDASSL

Rickettsia sp. S AGTITLDGSATITGDIG----NAGG--AALQGITLANDATKTLTLGGANI-IGAN-GGTIDFQANGGTIKFTSTQNNIV-VDFDLAITTDQTGVVDASSL

Candidatus R. goldwasserii AGAITLDGSATITGDIG----KVGG-AAALQGITLANDATKKLTLGGANI-IGAN-GGMIGFQANGGTIKLTSTQNNIV-VNFDLAITTDQTGVVDASSL

Rickettsia mongolotimonae AGTITLDGSATITGDIG----NAGG-AAALQGITLANDATKTLTLGGANI-IGAN-GGTIDFQANGGTIKFTSTQDNIV-VHFDLAIITDQTGVVDASSL

Rickettsia sp. BJ-90 AGTITLDGSATITGDIG----NAGG-AAALQGITLANDATKTLTLGGANI-IGAN-GGTIDFQANGGTIKFTSTQDNIV-VHFDLDIITDQTGVVDASSL

Candidatus R. andeanae AGAITLDGSATITGDIG----NGGG-AAALQGITLANDAKKTLTLGGANI-IGADVGGTIGFQANGGTIKLTSTQNNIL-VDFDLAITTDQTGVVDANSL

Rickettsia sp. Bar29 AGAITLDGSATITGDIG----NGGG-AAALQGITLANDATKTLTLGGANI-IGANVGGTIDFQANGGTIKLTSTQNNIL-VDFDLDITTDQTGVVDASSL

Rickettsia sp. TwKM01 AGAITLDGSATITGDIG----NGGG-XAALQDITLANDATKTLTLGGANI-IGANVGGTIGFQANGGTIKLTSTQNNIL-VDFDLDITTDQTGVVDASSL

R. raoultii AGAITLDGSATMTGDIG----NGGG-AAALQSITLANDATKTLTLGGANI-IGANVGGTIDFQANGGTIKLTSTQNNIL-VDFDLAITTDQTGVVDASSL

R. asiatica NGTIVLTGNATINGGIGVDIAAGGNLGKTIKNINL---GGKDLSYGGNTVKVQSAPNNGINFAAN-ETLSLTNTTDPIL-IASDI-ITTDKQGIIDGSAL

Rickettsia sp. 110 NGTIALTGNATINGGIGVDVAAGGNRGTVIKNINL---GGKNLSYGGKSIQIVNVPANGITFAAN-ETLTLTNATDPIA-INSNILITTDKQGIIDASAV

Rickettsia sp. mmx79 NGTIALTGNATINGGIGVDVAAGGNRGTVIKNINL---GGKNLSYGGKSIQIVNVPANGITFAAN-ETLTLTNATDPIA-INSNILITTDKQGIIDASAV

Rickettsia sp. 103 -----------------------------------------------------------------------------------NILITTDKQGIIDASAV

Rickettsia sp. J52 NGTIALTGNATINGGIG-DVAAGVNIGKVIKNINL---GGKNLSYGGKSIQIVNDPANGITFADN-ETLTLTNATDPIA-INSNILITTDKQGIIDASAV

Candidatus R. hoogstraalii GGAIQLDGSATITGDIG----NGGG-NAALQGITLANDASKTLTLGGANI-IGANAGRMIDFQANGGTIKLTSTQNNIL-VDFDLAIATDKTGVVDASSL

Rickettsia sp. California 2 NGAIQLDGSATITGDIG----NGAGNAAPIQGITLANDASKTLTLGGANI-IGANAGGTIDFQANGGTVKLTSTQNNIL-VDFDLAITTDKTGVVDASSL

R. hulinensis AGAITLDGSATITGDIG----NGGG-GAALQSITLANDATKTLTLGGANI-IGAN-GGTINFQANGGTIKLTSTQNNIV-VDCDLAIATDQTGVVDASSL

**[701-800 ]**

**[ | | | | | | | | | | ]**

R. bellii RML369-C ASGQGLVIKGDIGNY-------S-TSVRLGQFNIGSSATDFNAGNAAIGNLIFGNDGIALFAHNNYLITKTTNASGQGTIIFRP-VVADN--------TN

R. bellii OSU 85-389 ASGQGLVIKGDIGNY-------S-TSVRLGQFNIGSSATDFNAGNAAIGNLIFGNDGIALFAHNNYLITKTTNASGQGTIIFRP-VVADN--------TN

R. prowazekii Madrid E TNNQTLTINGSIGTV------VA-NTKTLAQLNIGSSKTILNAGDVAINELVIENNGSVQLNHNTYLITKTINAANQGQIIVAADPLNTN--------TT

R. prowazekii Rp22 TNNQTLTINGSIGTV------VA-NTKTLAQLNIGSSKTILNAGDVAINELVIENNGSVQLNHNTYLITKTINAANQGQIIVAADPLNTN--------TT

**R. typhi Wilmington TNNQTLTINGSIGTI------GA-NTKTLGRFNVGSSKTILNAGDVAINELVMENDGSVHLTHNTYLITKTINAANQGKIIVAADPINTD--------TA**

R. akari Hartford TNTQTLTIAGSIGTI------AA-NNKTLDQLKIGSSATTLNIGDVAINELVIGNNGSVNLGHVNYLIAKTTNASGQGKIIANS-TIVGNIGGNNCNVTR

R. australis TNTQTLTIAGRIGTI-------A-NNKTLGQLKIGSSATTLSIGDVAINELVIGNNGSVKLGHVNYLIAKTTNASGQGKIIADSNIVINV-GGHNLNITN

R. felis URRWXCal2 INAQTLTISGNIGTI------AA-NNKTLGQFNIGSSKTALNSGDVAINELVIGNNGSVQLAHNTYLITKTTNAANQGKIIFNP-VVNDN--------TT

R. canadensis McKiel TNAQTLTISGNIGTI----VPG--ANKTLGQLKVGSSKTILNAGDVAINELDIGNNGSVQLGHNTYLITKTINAAGQGKIIVAGDPANPT--------TT

R. tamurae TNDQTLTIAGKIGIVDIINNPA--QNVALGQLKIGSSKTALNSGNVAINELVIGNNGSVQFAHNTYLITKTTNAAGQGKITFNP-VVANN--------TT

R. helvetica TNDQTLTIAGKIGIVDIINNPA--QNVALGQLKIGSSKTALNSGNVAINELVIGNNGSVQFAHNTYLITKTTNAAGQGKITFNP-VVANN--------TT

REIS TNTQTLTIRGGIGTI------GA-NNKTLGQFNIGSSKTVLNGGDVAINELVIGNNGSVQFAHNTYLITKTTNAANQGKIIFNP-VVANS--------TT

R. monacensis TNAQTLTIRGGIGTI------GA-NNKTLGQFNIGSSKTVLNGGDVAINELVIGNNGSVQFAHNTYLITKTTNAANQGKIIFNP-LVANS--------TT

R. massiliae MTU5 TNAQTLTISGTIGTI------GA-NNKTLGQFNIGSSKTALNGGNVAINELVIGNNGSVQFAHNTYLITSTTNAADQGKIIFNP-VVNNN--------TT

Candidatus R. amblyommii TNAQTLTISGKIGTI------GA-NNKTLGQFNIGSSKTALNGGNVAINELVIGNNGSVQFAHDTYLITRTTNAAGQGKIIFNP-VVNNN--------TT

R. rhipicephali TNAQTLTISGTIGTI------GA-NNKTLGQFNIGSSKTALDGGNVAINELVIGNNGSVQFAHNNYLITSTTNAADQGKIIFNP-VVNNN--------TT

R. aeschlimannii TNAQTLTISGTIGAI------GA-NNKTLGQFNIGSSKTALNGGNVAINELVIGNNGSVQFAHNTYLITSTTNAVGQGKIIFNP-VVNNN--------TT

R. montanensis TNAQTLTISGTIGTV------GA-NNKTLGQFNVGSSKTALNGGNVAINELVIGNNGSVQFAHDTYLITKTTNAAGQGKIIFNP-VVNNN--------TT

R. slovaca 13-B TNAQTLTINGKIGTI------GA-NNKTLGQFNIGSSKTALSNGNVAINELVIGNNGAVQFAHNTYLITRTTNAAGQGKIIFNP-VVNNN--------TT

R. peacockii Rustic TNAQTLTINGKIGTI------GANNNKTLGQFNIGSSKTALSDGNVAINELFIGNDGAVQFAHNTYLITRTTNAAGQGKIIFNP-VVNNN--------TT

R. rickettsii Iowa TNAQTLTINGKIGTI------GD-NNKTLGQFNIGSSKTVLSNGNVAINELVIGNDGAVQFAHDTYLITRTTNAAGQGKIIFNP-VVNNG--------TT

R. rickettsii Sheila Smith TNAQTLTINGKIGTI------GA-NNKTLGQFNIGSSKTVLSNGNVAINELVIGNDGAVQFAHDTYLITRTTNAAGQGKIIFNP-VVNNG--------TT

R. japonica TNAQTLTISGTIGII------GA-NNTTLGQFNIGSSKTTLNGGNVAINELVIGNNGSVQFAHNTYLITRTTNAAGQGKIIFNP-VVNNN--------TT

R. honei TNAQTLTINGKIGTI------GV-NNKTLGQFNIGSSKTVLSDGNVAINELVIGNNGAVQFAHNTYLITRTTNAAGQGKIIFNP-IVNNS--------TI

R. conorii str. Malish 7 TNAQTLTINGKIGTV------GA-NNKTLGQFNIGSSKTVLSDGDVAINELVIGNNGAVQFAHNTYLITRTTNAAGQGKIIFNP-VVNNN--------TT

R. sibirica 246 TNAQTLTMNGKIGTI------GA-NNKTLGQFNIGSSKTALSDGDVAINELVIGNNGAVQFAHNTYLITRITNAAGQGKIIFNP-VVNNN--------TT

R. africae ESF-5 TNAQTLTINGKIGTI------GA-NNKTLGQFNIGSSKTALSGGDVAINELVIGNNGAVQFAHNTYLITRTTNAAGQGKIIFNP-VVNNN--------TT

R. parkeri TNAQTLTINGKIGTI------GA-NNKTLGQFNIGSSKTALRDGDVAINELVIGNNGAVQFAHNTYLITRTTNAAGQGKIIFNP-VVNNN--------TT

R. heilongjiangensis TNAQTLTISGTIGTI------GA-NNKTLEQFNIGSSKTVLNGGNVAINELVIGNNGSVQFAHNSYLITRTTNAAGQGKIIFNP-VVNNN--------TT

Israeli tick typhus TNAQTLTINGKIGTI------GA-NNKTLGQFNIGSSKTVLSDGDVAINELVIGNNGAVQFAHNTYLITRTTNAAGQGKIIFNP-VVNNN--------TT

Rickettsia sp. A-167 TNAQTLTINGKIGTI------GA-NNKTLGQFNIGSSKTVLSDGDVAINELVIGNNGAVQFAHNTYLITRTTNAAGQGKIIFNP-VVNNN--------TT

Candidatus R. tasmanensis TNAQTLTISGTIGTI------GA-NNKTLGQFNIGSSKTALNRGNVAINELVIGNNGSVQFAHNAYLITRTTNAAGQGKIIFNP-VVNNN--------TT

Rickettsia sp. IG-1 TNAQTLTINGKIGTI------GA-NNKTLGQFNIGSSKTALSDGDVAINELVIGNNGAVQFAHNTYLITRTTNAAGQGKIIFNP-VVNNN--------TT

Candidatus R. barbariae TNAQTLTINGKIGTI------GA-NNKTLGQFNIGSSKTALSGGDVAINELVIGNNGAVQFAHNTYLITRTTNAAGQGKIIFNP-VVNNN--------TT

Rickettsia sp. S TNAQTLTINGKIGTI------GA-NNKTLGQFNIGSSKTALSNGDVAINELVIGNNGAVQFAHNTYLITRTTNAAGQGKIIFNP-VANNN--------TT

Candidatus R. goldwasserii TNAQTLTINGKIGTV------GA-NNKTLGQFNIGSSKTILSDGNVAINELVIGNNGAVQLAHNTYLITRTTNAAGQGKIIFNP-VVNNN--------TT

Rickettsia mongolotimonae TNAQTLTINGKIGTI------GA-NNKTLGQFNIGSSKTALSDGDVAINELVIGNNGAVQFAHNTYLITSTTNAAGQGKIIFNP-VVNNN--------TT

Rickettsia sp. BJ-90 TNAQTLTMNGKIGTI------GA-NNKTLGQFNIGSSKTALSDGDVAINELVIGNNGAVQFAHNTYLITRITNAAGQGKIIFNP-VVNNN--------TT

Candidatus R. andeanae TNAQTLTISGKIGTI------GA-NNKTLGQFNIGSSKTALNGGNVAINELVIGNNGSVQFAHNTYLITRTTNAAGQGKIIFNP-VVNNN--------TT

Rickettsia sp. Bar29 TNAQTLTISGTIGTI------GA-NNKTLGQFNIGSSKTALNGGNVAINELVIGNNGSVQFAHNTYLITSTTNAADQGKIIFNP-VVNNN--------TT

Rickettsia sp. TwKM01 TNAQTLTISGTIGTI------GA-NNKTLGQFNIGSSKTALNGGNVAINELVIGNNGSVQFAHNTYLITSTTNAADQGKIIFNP-VVNNN--------TT

R. raoultii TNAQTLTISGTIGTI------GA-NNKTLGQFNIGSSKTALNGGNVAINELVIGNNSSVQFAHNTYLITRTTNAAGQGKIIFNP-VVNNN--------TT

R. asiatica TNDQTLTIAGKIGIVDIINNPA--QNVALGQLKIGSSKTALNSGNVAINELVIGNNGSVQFAHNTYLITKTTNAAGQGKITFNP-IVANN--------TT

Rickettsia sp. 110 TNNQTLTISGTIGVVDIVNNPAP-NNKSLGQLNVGSSKTILNAGDAAIAELVIGNNGSVQLGDDNYLIAKTTNAAGQGKIIV-----AGE--------TI

Rickettsia sp. mmx79 TNNQTLTISGTIGVVDIVNNPAP-NNKSLGQLNVGSSKTILNAGDAAIAELVIGNNGSVQLGDDNYLIAKTTNAAGQGKIIV-----AGE--------TI

Rickettsia sp. 103 TNNQTLTISGKIGVVDIVNNPAT-NNKSLGQLNVGSSKTILNAGDAAIAELVIGNNGSVQLGHDHYLIAKTTNAAGQGKIIVAANTGAGD--------TT

Rickettsia sp. J52 TNNQTLTISGKIGVVDIVNNPAT-NNKSLGQLNVGSSKTILNAGDAAIAELVIGNNGSVQLGHDHYLIAKTTNAAGQGKIIVAANTGAGN--------TT

Candidatus R. hoogstraalii TNAQTLTIKGNIGII------AA-NNKTLGQFNIGSSKTVLNAGDVAINELVIGNNGSVQFAHNTYLITKTTNAAGQGKIIFNP-IVNNN--------TT

Rickettsia sp. California 2 INAQTLTISGNIGTI------AA-NNKTLGQFNIGSSKTALNSGDVAINELVIGNNGSVQLAHNTYLITKTTNAANQGKIIFNP-VVNDN--------TT

R. hulinensis TNAQTLTISGTIGTI------GA-NNKTLEQFNIGSSKTALNGGNVAINELVIGNNGSVQFAHNSYLITRTTNAAGQGKIIFHP-VVHNN--------TT

**[801-900 ]**

**[ | | | | | | | | | | ]**

R. bellii RML369-C LVAGTNLGSADNPLAEINFAAPAGTNTDTILNVGQGVNLYATEITTAGVNTGSFNFNGGRTSIVSGTVGTA-ANKFNNITLTNNTTVKFTDDVISNGATT

R. bellii OSU 85-389 LVAGTNLGSADNPLAEINFAAPAGTNTDTILNVGQGVNLYATEITTAGVNTGSFNFNGGGTSIVSGTVGIA-ANKFNNITLTNNTTVKFTDDVISNGATT

R. prowazekii Madrid E LADGTNLGSAENPLSTIHFATKA-ANADSILNVGKGVNLYANNITTNDANVGSLHFRSGGTSIVSGTVGGQQGHKLNNLILDNGTTVKFLGDTTFNGGTK

R. prowazekii Rp22 LADGTNLGSAENPLSTIHFATKA-ANADSILNVGKGVNLYANNITTNDANVGSLHFRSGGTSIVSGTVGGQQGHKLNNLILDNGTTVKFLGDTTFNGGTK

**R. typhi Wilmington LADGTNLGSAESPLSNIHFATKA-ANGDSILHIGKGVNLYANNITTTDANVGSLHFRSGGTSIVSGTVGGQQGLKLNNLILDNGTTVKFLGDITFNGGTK**

R. akari Hartford LMAGTNLGSAEAPLSEFNFAAPTGGAIGTVLEVLGGASIHANNITTIAPNNGFFRFVGGGTSIISGTVGTP-ANKIGGVYLDGNSTVQFAGNSVFDGHTF

R. australis LMAGTNLGSAEAPLSEFNFAAPTGGAISTVLTMIGGSNIYANNITTTTPNTGGFGFIGGGTSIISGTVGTP-ANKIGHVYLDGNSTVQFAGNAIFNDHTT

R. felis URRWXCal2 LAAGTNLGSEANPLAEINFGSKG-VNGDTILNVGQGVNLYATNITTTDANVGSFSFTVGGTNIVSGTVGGQQGNKFNTVELDNGTTAKFLGNAIFNGETT

R. canadensis McKiel LSAGTNLGSAANALAEINFAVPV-AQNDSILEFGKGVNLYATNITTAVADTGSFSFTAGGINIVSGIVGGQQGNKFNTVELDNGTTAQFLGSAIFNGKST

R. tamurae LADGTNLGSAANPLSEINFGSKG-ANADTLLNVGKGVNLYATNITTTDANTGSFGFNAGGTYLVNGTVGGQQGNKFNTVELDNGTTAYFLGNAIFNGETT

R. helvetica LADGTNLGSAANPLSEINFGSKG-ANADTLLNVGKGVNLYATNITTTDANTGSFVFNAGGTNIVSGTVGGQQGNKFNTVELDNGTTAYFLGNAIFNGETT

REIS LAAGTKLGSTAKPLAEINFA----PAIDTILNVGQGVNLYATNITTAAANMGSFSFKAGGTNIVRGTVGGQQGNKLNTVTLDNGTTAKFLGNATFNGETK

R. monacensis LAAGTKLGSAAKPLAEINFA----PAINTILNVGQGVNLYATNITTAVADMGSFSFKAGGTNIVRGTVGGQQGNKLNTVTLDNGTTAKFLGNATFSGGTT

R. massiliae MTU5 LAAGTNLGSAANPLAEINFGSKG-AHADTILNVGEGVNLYATNITTTDANVGSFVFNAGGTNIVSGTVGGQQGNKFNTVALDNGTTVKFLGNATFNGETT

Candidatus R. amblyommii LAAGTNLGSAANPLAEINFGSKG-AHADTILNVGEGVNLYATNITTTDANVGSFVFNAGGTNIVSGTVGGQQGNKFNTVALDNGTTVKFLGNATFNGETT

R. rhipicephali LAAGTNLGSAANPLAEINFGSKG-AHADTILNVGEGVNLYATNITTTDANVGSFVFNAGGTNIVSGTVGGQQGNKFNIVALDNGTTVKFLGNATFNGETT

R. aeschlimannii LAAGTNLGSAANPLAEINFGSKG-AHADTILNVGKGVNLYATNITTTDANVGSFVFNAGGTNIVSGTVGGQQGNKFNTVALDNGTTVKFLDNATFNGETI

R. montanensis LAAGTNLGSAANPLAEINFGSKG-AHADTILNVGKGVNLYATNITTTDANVGSFVFNAGGTNIVSGTVGGQQGSKFNTVELDNGTTVKFLGNAMFNGGTK

R. slovaca 13-B LAAGTNLGSATNPLAEINFGSKG-VNVDTVLNVGEGVNLYATNITTTDANVGSFIFNAGGTNIVSGTVGGQQGNKFNTVALDNGTTVKFLGNATFNGNTT

R. peacockii Rustic LAAGTNLGSATNPLAEINFGSKG-VNVDTVLNVGEGVNLYATNITTTDANVGSFVFNAGGTNIVSGTVGGQQGNKFNTVALDNGTTVKFLGNATFNGNTT

R. rickettsii Iowa LAAGTNLGSATNPLAEINFGSKG-VNVDTVLNVGEGVNLYATNITTTDANVGSFVFNAGGTNIVSGTVGGQQGNKFNTVALENGTTVKFLGNATFNGNTT

R. rickettsii Sheila Smith LAAGTNLGSATNPLAEINFGSKG-VNVDTVLNVGEGVNLYATNITTTDANVGSFVFNAGGTNIVSGTVGGQQGNKFNTVALENGTTVKFLGNATFNGNTT

R. japonica LAAGTNLGSAANPLAEINFGSKG-ARADTVLNVGEGVNLYATNITTTDANVGSFVFNAGGKNIVSGTVGGQQGNKFNTVALDNGTTVKFLGNATFNGNTT

R. honei LAAGTNLGSATNPLAEINFGSKG-ANVDTVLNVGKGVNLYATNITTTDANVGSFVFNAGGTNIVSGTVGGQQGNKFNTVELGNGTTVKFLGNATFNGNTT

R. conorii str. Malish 7 LATGTNLGSATNPLAEINFGSKGAANVDTVLNVGKGVNLYATNITTTDANVGSFIFNAGGTNIVSGTVGGQQGNKFNTVALDNGTTVKFLGNATFNGNTT

R. sibirica 246 LAAGTNLGSATNPLAEINFGSKG-VNVDTVLNVGEGVNLYATNITTTDANVGSFIFNAGGTNIVSGTVGGQQGNKFNTVALDNGTTVKFLGNATFNGNTT

R. africae ESF-5 LAAGTNLGSATNPLAEINFGSKG-VNVDTVLNVGEGVNLYATNITTTDANVGSFIFNAGGINIVSGTVGGQQGNKFNTVALDNGTTVKFLGNATFNGNTT

R. parkeri LAAGTNLGSATNPLAEINFGSKG-VNVDTVLNVGEGVNLYATNITTTDANVGSFIFNAGGINIVSGTVGGQQGNKFNTVALDNGTTVKFLGNATFNGNTT

R. heilongjiangensis LAAGTNLGSAANPLAEINFGSKG-VHADTVLNVGEGVNLYATNITTTDANVGSFVFNAGGKNIVSGTVGGQQGNKFNTVALDNGTTVKFLGNATFNGNTT

Israeli tick typhus LATGTNLGSATNPLAEINFGSKG-VNVDTVLNVGEGVNLYATNITTTDANVGSFIFNAGGTNIVSGTVGGQQGNKFNNVALNNGTTVKFLGNATFNGNTT

Rickettsia sp. A-167 LATGTNLGSATNPLAEINFGSKG-VNVDTVLNVGKGVNLYATNITTTDANVGSFIFNAGGTNIVSGTVGGQQGNKFNNVALNNGTTVKFLGNATFNGNTT

Candidatus R. tasmanensis LAAGTNLGSAANPLAEINFGSKG-AHADTILNVSEGVNLYATNITTIDANVGSFVFNAGGTNIVSGTVGGQQGNKFNTVALDNGTTVKFLGNATFNGDTT

Rickettsia sp. IG-1 LAAGTNLGSATNPLAEINFGSKG-VNVDTVLNVGEGVNLYATNITTTDANVGSFVFNAGGTNIVSGTVGGQQGNKFNTVALDNGTTVKFLGNATFNGNTT

Candidatus R. barbariae LAAGTNLGSATNPLAEINFGSKG-VNVDTVLNVGEGVNLYATNITTTDANVGSFIFNAGRTNIVSGTVGGQQGNKFNTVALDNGTTVKFLGNATFNGNTT

Rickettsia sp. S LAAGTNLGSATNPLAEINFGSKG-VNVDTVLNVGEGVNLYATNITTTDANVGSFIFNAGGTNIVSGTVGGQQGNKFNTVALDNGTTVKFLGNATFNGNTT

Candidatus R. goldwasserii LAAGTNLGSAANPLAEINFGSKG-ANADTVLNVGEGVNLYATNITTTDANVGSFVFNAGGTNIVSGTVGGQQGNKFNTVALDNGTTVKFLDNATFNGNTT

Rickettsia mongolotimonae LAAGTNLGSATNPLAEINFGSKG-VNVDTVLNVGEGVNLYATNITTTDANVGSFIFNAGGTNIVSGTVGGQQGNKFNTVALDNGTTVKFLGNATFNGNTT

Rickettsia sp. BJ-90 LAAGTNLGSATNPLAEINFGSKG-VNVDTVLNVGEGVNLYATNITTTDANVGSFIFNAGGTNIVSGTVGGQQGNKFNTVALDNGTTVKFLGNATFNGNTT

Candidatus R. andeanae LAAGTNLGSAANPLAEINFGSKG-AHADTILNVGKGVNLYATNITTTDANVGSFVFNAGGTNIVSGTVGGQQGHKFNTVALDNGTTVKFLGNATFNGETT

Rickettsia sp. Bar29 LAAGTNLGSAANPLAEINFGSKG-AHADTILNVGEGVNLYATNITTTDANVGSFVFNAGGTNIVSGTVGGQQGNKFNTVALDNGTTVKFLGNATFNGETT

Rickettsia sp. TwKM01 LAAGTNLGSAANPLAEINFGSKG-AYADTILNVGEGVNLYATNITTTDANVGSFVFNAGGANIVSGTVGGQQGNKFNTVALDNGTTVKFLDNATFNGETT

R. raoultii LAAGTNLGSAANPLAEINFGSKG-AHADTILNVGEGVNLYATNITTTDANVGSFVFNAGGTNIVSGTVGGQQGNKLNTVALDNGTTVKFLGNATFNGETT

R. asiatica LADGTNLGSAANPLSEINFGSKG-ANADTLLNVGKGVNLYATNITTTDANTGSFVFNAGGTNIVSGTVGGQQGNKFNTVELDNGTTAYFLGNAIFNGETT

Rickettsia sp. 110 LMAGTNLGSVAAPLSEVNFAEPA-VNGGMPSMCARNINLYANNITTTHPNTGSXVFDAGGTNIVSGTVGGQQGKKLNTVTLSHGTTAQFLGNAIFNGATM

Rickettsia sp. mmx79 LMAGTNLGSVAAPLSEVNFAEPA-VNGGMPSMCARNINLYANNITTTHPNTGSXVFDAGGTNIVSGTVGGQQGKKLNTVTLSHGTTAQFLGNAIFNGATM

Rickettsia sp. 103 LMAGTNLGSVAAPLSEVNFAAPV-VNKGIQLIFGKNVNLYANNITTTNPNTGSFVFGAGGTNIVSGTVGGQQGKKLNAVTLGNGTTAQFLGNAIFNGATT

Rickettsia sp. J52 LMAGTNLGSVAAPLSEVNFAAPV-VNVDIQLIFGKNVNLYANNITTTNPNTGSXVFDAGGTNIVSGTVGGQQGKKLNAVTLDNGTTAQFLGNAIFNGATT

Candidatus R. hoogstraalii LAAGTNLGSATNPLAEINFEAP--AGGATTLNVGKGVNLYATNITTATPNVGTFSFTAGGTNIVSGTVGGQQGNKFNTVELDNGSTASFLGNATFNGETT

Rickettsia sp. California 2 LAAGTNLGSEANPLAEINFGSKG-VNGDTILNVGQGVNLYATNITTTDANVGSFSFTVGGTNIVSGTVGGQQGNKFNTVELDNGTTAKFLGNAIFNGETT

R. hulinensis LAAGTNLGSAANPLAEINFGSKG-GHADTVLNVGEGVNLYATNITTTDANVGSFVFNAGGKNIVSGTVGGQQGNKFNTVALDNGTTVKFLGNATFNGNTT

**[901-1000 ]**

**[ | | | | | | | | | | ]**

R. bellii RML369-C IGGNSTLQIANDYITDSIQGNAAGGTGTLQFVNTKGILVTLKGNPNPDNALAALQVSGSADVAID-----------------------------------

R. bellii OSU 85-389 IGGNSTLQIANDYITDSIQGNAAGDTGTLQFVNTKGILVTLKGNPNPDNALAALQVSGSADVAID-----------------------------------

R. prowazekii Madrid E IEGKSILQISNNYTTDHVE--SADNTGTLEFVNTDPITVTLNKQGAYFGVLKQVIISGPGNIVFNEIGNVGIVHGIAANSISFEN----ASLGTSLFLPS

R. prowazekii Rp22 IEGKSILQISNNYTTDHVE--SADNTGTLEFVNTDPITVTLNKQGAYFGVLKQVIISGPGNIVFNEIGNVGIVHGIAANSISFEN----ASLGTSLFLPS

**R. typhi Wilmington IEGKSILQISSNYITDHIE--SADNTGTLEFVNTDPITVTLNKQGAYFGVLKQVMVSGPGNIAFNEIGN-GVAHAIAVDSISFEN----ASLGASLFLLS**

R. akari Hartford ISGNSTLQIGGDYTADFIA--SDDNTGIVEFVNTSPIAVTLNKQAGLVDDLKQITVSGRGNVVINEIGNAGNNHAIKTNTISFEN----ASLGAGLFLPS

R. australis IISNSTLQIGGDYTASFIA--SDDNTGIVEFVNTAPITVTLNKQAGHVNDLKQITVSGRGNVVINEIGNAGNNHAIETDTISFEN----ASLGAGLFLAS

R. felis URRWXCal2 IEANSILQIGGNYTADKVE--SADGTGIVEFVNTTPITVTLNKQAGPVDDLKQITVSGRGNVVINEIGNAGNDHGAATDTISFEN----VSLGAALFLPN

R. canadensis McKiel IDGNSTLQIGSNYTASFIK--SADNTGIVEFVNADNITVTLQTQAAAVDALKQITVSGPGNVVFNETGNLA-EHGINTTKIAFEN----ASLGTRLFLPS

R. tamurae IKANSTLQIGGNYTTDFVT--SADNTGIVKFVNADLITVTLNKQAAPVNAVEQITVSGPGNIVINEIGNAGNEHGMATDTISFEN----ASLGAALFLPS

R. helvetica IEANSTLQIGGNYTTDFVT--SADNTGIVKFVNADLITVTVQKQAGVVDALKQITVSGSGNVVINQTGNAAN-PGVVTDTIAFAD----ASLGTSLFLPS

REIS IENNSTLQIGGNYTADFVA--SADGTGIVEFVNTGPITVTLNKQAVPVNALKQITFSGPGNIVINEIGNAGNEHGVVTDTISFEN----ASLGAVLFLPS

R. monacensis IENNSTLQIGGNYTADFVA--SADGTGIVEFVNTGPITVTLNKQAVPVNALKQITFSGPGNIVINEIGNAGNEHGAVTDTISFEN----ASLGAVLFLPS

R. massiliae MTU5 IEANSTLQIGSNYTADFVA--SADGTGIVEFVNTGPITVTLNKQAAPVGYLKQITVSGTGNVVVNGIGNAGN-PGAATDTIAFEN----SSLGAALFLPS

Candidatus R. amblyommii IAANSTLQIGGNYTADCVA--SADGTGIVEFVNTGPITVTLNKQAVPVNNLKHITVSGPGNVVVNEIGNAVNYHGVATDTIAFEN----SSLGAALFLPS

R. rhipicephali IEANSTLQIGGNYTADFVA--SADGTGIVEFVNTGPITVTLNKQAAPVGYLKQITVSGPGNVVVNGVGNAGNNPGAATDTIAFEN----SSLGAALFLPS

R. aeschlimannii IEANSTLQIGGNYTADFVA--SADGTGIVEFVNTGPITVTLNKQAAPVDYLKQITVSGPGNVVINGIGNAGNNHGVATDTIAFEN----SSLGATLFLPS

R. montanensis IEAKSTLQIGGNYTADFVA--SADGTGIVEFVNTGPITVTLNKQAAPVGVLKQITVSGPGNVVVNEIGNAGNAHGAVTGTIAFEN----SSLGATLFLPS

R. slovaca 13-B IAANSTLQIGGNYTADFVA--SADGTGIVEFVNTGPITVTLNKQAAPVNALKQITVSGPGNVVINEIGNAGNYHGAVTDTIAFEN----SSVGAVVFLPR

R. peacockii Rustic IAANSTLQIGGNYTADFVA--SADGTGIVEFFNTGPITVTLNKQAAPVNALKQITVSGPGNVVINEIGNAGNYHGAVTDTIAFEN----SSLGAVVFLPR

R. rickettsii Iowa IAANSTLQIGGNYTADCVA--SADGTGIVEFVNTGPITVTLNKQAAPVNALKQITVSGPGNVVINEIGNAGNHHGAVTDTIAFEN----SSLGAVVFLPR

R. rickettsii Sheila Smith IAANSTLQIGGNYTADCVA--SADGTGIVEFVNTGPITVTLNKQAAPVNALKQITVSGPGNVVINEIGNAGNHHGAVTDTIAFEN----SSLGAVVFLPR

R. japonica IAANSTLQISGNYTADFIA--SADGTGIVEFVNTGPINVTLNKQAVPVNALKQITVSGPGNVVVNEIGNAGNYHGAMTDTIAFEN----SSLGAVLFLPS

R. honei IAANSTLQIGGNYTADFVA--SADGTGIVEFVNTSPINVTLNKQVAPVNALKQITVSGPGNVVINEIGNAGNYHGAVTDTIAFEN----SSLDAVVFLPR

R. conorii str. Malish 7 IAANSTLQIGGNYTADFVA--SADGTGIVEFVNTGPITVTLNKQAAPVNALKQITVSGPGNVVINEIGNAGNYHGAVTDTIAFEN----SSLGAVVFLPR

R. sibirica 246 IAANSTLQIGGNYTADFVA--SADGTGIVEFVNTDPITVTLNKQAAPVNALKQITVSGPGNVVINEIGNAGNYHGAVTDTIAFEN----SSLGTVVFLPR

R. africae ESF-5 IAANSTLQIGGNYTADFVA--SADGTGIVEFVNTDPITVTLNKQAAPVNALKHITVSGPGNVVINEIGNAGNYHGAVTDTIAFEN----SSLGAVVFLPR

R. parkeri IAANSTLQIGGNYTADFVA--SADGTGILEFVNTGPITVTLNKQAAPVNALKQITVSGPGNVVINEIGNAGNYHGAVTDTVAFEN----SSLGAVVFLPR

R. heilongjiangensis IAANSTLQISGNYTADVIA--SADGTGIVEFVNTGPINVTLNKQAVPVNALKQITVSGPGNVVVNEIGNAGNYHGAMTDTIAFEN----SSLGAVLFLPS

Israeli tick typhus IAANSTLQIGGNYTTDFVA--SADGTGIVEFVNTGPITVTLNKQAAPVNALKQITVSGPGNVVINEIGNAGNYHGAVTDTIAFEN----SSLGAVVFLPR

Rickettsia sp. A-167 ITANSTLQIGGNYTADFVA--SADGTGIVEFVNTGPITVTLNKQAAPVNALKQITVSGPGNVVINEIGNAGNYHGAVTDTIAFEN----SSLGAVVFLPR

Candidatus R. tasmanensis IGANSTLQIGGNYTADFVA--SADGTGIVEFVNTGPITVTLNKQAAPVDALKQITVSGPGNVVINEIGNAGNYHGAATDTIAFEN----SSLGAALFLPS

Rickettsia sp. IG-1 IAANSTLQIGGNYTADFVA--SADGTGIVEFVNTGPITVTLNKQAAPVNALKQITVSGPGNVVINEIGNAGNSHGVVTDTIAFEN----SSLGAVVFLPR

Candidatus R. barbariae IAANSTLQIGGNYTADFVA--SADGTG-------------------------------------------------------------------------

Rickettsia sp. S IVANSTLQIGGNYTADFVA--SADGTGIVEFVNTDPITVTLNKQAAPVNALKQITVSGPSNVVINEIGNAGNYHGAVTDTIAFEN----SSLGAVVFLPR

Candidatus R. goldwasserii IAVNSTLQIGGNYTADCVA--SADGTGIVEFVNTAPITVTLNKQAVLVDALKQITVSGPGNVVINEIGNAGNYHGAVTDTIVFEN----SSLGAAVFLPR

Rickettsia mongolotimonae IAANSTLQIGGNYTADFVA--SADGTGIVEFVNTDPITVTLNKQAAPINALKQITVSGPGNVVINEIGNAGNYHGAVTDTIAFEN----SSLGTVVFLPR

Rickettsia sp. BJ-90 IAANSTLQIGGNYTADFVA--SADGTGIVEFVNTDPITVTLNKQAAPVNALKQITVSGPGNVVINEIGNAVNYHGAVTDTIAFEN----SSLGTVVFLPR

Candidatus R. andeanae IAANSTLQIGGNYTADFVA--SADGSGIVEFVNTGPITVTLNKQAAPVNNLKQITVSGPGNVVVNEIGNAVNYHGVVTDTITFEN----SSLGAALFLPS

Rickettsia sp. Bar29 IEANSTLQIGSNYTADFVA--SADGTGIVEFVNTGPITVTLNKQAAPVGYLKQVTVSGTGNVVVNGIGNAGNNPGAATDTIAFEN----SSLGAALFLPS

Rickettsia sp. TwKM01 IEANSTLQIGGNYTADFVA--SADGTGIVEFVNTGPITVTLNKQAAPVGYLKQITVSGPGNVVVNGIGNAGNNPGAATDTIAFEN----SSLGAALFLPS

R. raoultii IEANSTLQIGGNYTADFVA--SADGTGIVEFVNTGPITVTLNKQAAPVNNLKQITVSGPGNVVINEIGNAVNDHGATTGTIAFEN----SSLGAVLFLPS

R. asiatica IKANSTLQIGGNYTTDFVT--SADNTGIVKFVNADLITVTVQKQARVVDVLKQITVSGSGNVVINQTGNAAN-PGAVTDTIAFAN----ASLGASLFLPS

Rickettsia sp. 110 IENASTLQIGGNYTADKVE--SADHTGIVQFVNADDITVTLNKQAGAVHYLKQITVSGPGNVVINEIGNAGNDYGVSTNTIAFTE----ASLGAAIFLPS

Rickettsia sp. mmx79 IENASTLQIGGNYTADKVE--SADHTGIVQFVNADDITVTLNKQAGAVHYLKQITVSGPGNVVINEIGNAGNDYGVSTNTIAFTE----ASLGAAIFLPS

Rickettsia sp. 103 IEAASTLQIGGNYTAGKVE--SADNTGIVQFVNADDITVTLNKQAGAVDALKQITVSGPGNVVINEIGNAGNDHGVETDTIAFTE----ASLGAAIFLPS

Rickettsia sp. J52 IKAASTLQIGGNYTADKVE--SADNTGIVQFVNADDITVTLNKQAGAVDDLKQITVSGPGNVVINEIGNAGNDYGVKTDTIAFTE----ASLGADIFLPS

Candidatus R. hoogstraalii IEGNSTLQIGGNYTTNLFT--SVDNTGIIEFVNADPITVTVNKQARVVNELKQVTISGPGNVVINQIGNAAN-PGAVTDTVVFANASLGASLGASLSLAS

Rickettsia sp. California 2 IEANSILQIGGNYTADKVE--SADGTGIVEFVNTTPITVTLNKQAGPVDDLKQITVSGRGNVVINEIGNAGNDHGAATDTISFEN----VSLGAALFLPN

R. hulinensis IAANSTLQISGNYTADFIA--SADGTGIVEFVNTGPINVTLNKQAVPVNALKQITVSGPGNVVVNEIGNAGNYHGAMTDTIAFEN----SSLGAVLFLPS

**[1001-1100 ]**

**[ | | | | | | | | | | ]**

R. bellii RML369-C ----------------------------------------------------------------------------------------------------

R. bellii OSU 85-389 ----------------------------------------------------------------------------------------------------

R. prowazekii Madrid E GTPLDV------LTIKSTVG-NGTVDN-FNAPIVVVSGIDSMINNGQIIGDKKNIIALSLGSDNSITVNANTLYSGIRTTKNNQGTVTLSGGMPNNPGTI

R. prowazekii Rp22 GTPLDV------LTIKSTVG-NGTVDN-FNAPIVVVSGIDSMINNGQIIGDKKNIIALSLGSDNSITVNANTLYSGIRTTKNNQGTVTLSGGMPNNPGTI

**R. typhi Wilmington GTPLDV------LTIKSTVG-NGTVDN-FNAPILVVSGIDSMINNGQVIGDQKNIIALSLGSDNSITVNSNTLYAGIRTTKTNQGTVTLSGGIPNNPGTI**

R. akari Hartford DTPLDG------LTIKSTVG-NGTPGN-LNAPIVVVSGIDSVIADGQAIGDQNNIVGLCLGSDNGVTVDATTLYAGIGTTKNNQGTVTLNGGIPNTPGTI

R. australis GTSLDG------LTIKSKVG-NGTPGN-FNVPAVIVSGIDSVIADGQAIGDQKNIVGLGLGSDNGIIVNATTLYAGIGTTKNNQGTVTLSGGIPNTPGTI

R. felis URRWXCal2 GIPLDG------LTIKSTVG-NETATGNFDVPRLIVSGVDSVIADGQAIGDQDNIVGLGLGSDNSITVNATKLYAGIGSVNNNQGTVTLSGGIPNTPGTI

R. canadensis McKiel KIPFDG------LTITSTVG-NGLVGD-FSVPAVIVSDIDSKIVPGQVIGDQNNIVGLGLASDDTITVDDITLNAGLFTVHDGQGTIELNGGIPNTPGTI

R. tamurae GIPFNDAGNAVPLTIKSTVGNNGPEGD-FNVPVVIVSDVDSVIADDQVIGDQNNIVGLSLGSDNGIAVNTTTLYAGIGTIKNNQGTVILSGGIPNTPGTI

R. helvetica GLPFNDAGNTIPLTIKSTVG-NGPEGD-FNVPRVIVSGIDSVIADGQVIGDQDNIIGLGLGSDNGIIVNATTLYAGIGTVNDNQGTVTLSGGVPNTPGTI

REIS GIPFNDAGNTVPLTIKSTVG-NGPAGD-FNVPVVFVSGVDSVIADGQVIGDQNNIVGLGLGSDNGIAVNATTLYAVIRTTKNNQGTIILSGGIPNTPGTI

R. monacensis GIPFNNAVNQVPLTIKSTVG-NGPAGN-FNVPVVFVSGVDSVIADGQVIGDQNNIVGLGLGSDNGIAVNATTLYAVIRTTKNNQGTVILSGGIPNTPGTI

R. massiliae MTU5 GIPFNDAGNTMPLTIKSTVG-NRTATG-FDVPKVIVLGVDSVIADGQVIGDQNNIVGLGLGSDNGIIVNATTLYAGIGTINNNQGTVTLSGGVPNTPGTV

Candidatus R. amblyommii GVPFNDAGNAIPLTIKSTVG-NETATG-FDVPKVIVLGVDSVIADGQVIGDQNNIVGLGLGSDNGIIVNATTLYAGIGTINNNQGTVTLSGGIPNTPGTV

R. rhipicephali GIPFNDAGNTMPLTIKSTVG-NKTATG-FDVPKVIVLGVDSVIADGQVIGDQNNIVGLGLGSDNGIIVNATTLYAGIGTINNNQGTVTLSGGVPNTPGTV

R. aeschlimannii GIPFNDAGNAIPLTIKSTVG-NETAKG-FDVPRVIVLGVDSVIADGQVIGDQNNIVGLGLGSDNGIIVNATTLYAGIGTINNNQGTVTLSGGIPNTPGTV

R. montanensis GIPFND------VTIKSTVG-NETAKG-FDAPKVIVSGVDSVIADGQVIGDQNNIVGLGLGSDNGIIVNATKLYAGIGTTNNNQGTVILSGGVPNTPGTV

R. slovaca 13-B GIPFNDAGNIIPLTIKSTVG-NKTATG-FDVPSVIVLGVDSVIADGQVIGDQNNIVGLGLGSDNDIIVNATTLYAGIGTINNNQGTVTLSGGIPNTPGTV

R. peacockii Rustic GIPFNDAGNTIPLTIKSTVG-NKTATG-FDVPSVIVLGVDSVITDGQVISDQNNIVGLGLGSDNGIIVNATTLYARIGTINNNQGTVTLSGGVPNTPGTV

R. rickettsii Iowa GIPFNDAGNTMPLTIKSTVG-NKTAKG-FDVPSVVVLGVDSVIADGQVIGDQNNIVDLGLGSDNGIIVNATTLYAGISTLNNNQGTVTLSGGVPNTPGTV

R. rickettsii Sheila Smith GIPFNDAGNTMPLTIKSTVG-NKTAKG-FDVPSVVVLGVDSVIADGQVIGDQNNIVGLGLGSDNGIIVNATTLYAGISTLNNNQGTVTLSGGVPNTPGTV

R. japonica GIPFNDAGNTIPLTIKSTVG-NETAEG-FSVPSVIVSGVDSVIADGQVIGDQNNIVGLGLGSDNGIIVNATTLYAGIGTINNNQGTVTLSGGVPNTPGTV

R. honei GIPFNDAGNTMPLTIKSTVG-NKTAKG-FAVPSVVVLGVDSVIADGQVIGDQDNIVGLGLGSDNGIIVNATTLYAGIGTVNNNQGTVTLSGGVPNTPGTV

R. conorii str. Malish 7 GIPFNDAGNRIPLTIKSTVG-NKTATG-FDVPSVIVLGVDSVIADGQVIGDQNNIVGLGLGSDNDIIVNATTLYAGIGTINNNQGTVTLSGGIPNTPGTV

R. sibirica 246 GIPFNDAGNIIPLTIKSTVG-NKTATG-FDVPSVIVLGVDSVIADGQVIGDQNNIVGLGLGSDNDIIVNATTLYAGIGTINNNQGTVTLSGGIPNTPGTV

R. africae ESF-5 GIPFNDAGNIIPLTIKSTVG-NKTAAG-FDVPSVIVSGVDSVIADGQVIGDQNNIVGLGLGSDNDIIVNATTLYAGIGTINNNQGTVTLSGGIPNTPGTV

R. parkeri GIPFNDAGNIMPLTIKSTVG-NKTATG-FNVPSVIVSGVDSVIADGQVIGDQNNIVGLGLGSDNDIIVNATTLYAGIGTINNNQGTVTLSGGIPNIPGTV

R. heilongjiangensis GIPFNDAGNIIPLTIKSTVG-NETAKG-FNVPSVIVSGVDSVIADGQVIGDQNNIVGLGLGSDNGIIVNATTLYAGIGTINNNQGTVTLSGGIPNTPGTV

Israeli tick typhus GIPFNDAGNIIPLTIKSTVG-NKTATG-FDVPSVIVLGVDSVIADGQVIGDQNNIVGLGLGSDNDIIVNATTLYAGIGTINDNQGTVTLSGGIPNTPGTV

Rickettsia sp. A-167 GIPFNDAGNIIPLTIKSTVG-NKTATG-FDVPSVIVLGVDSVIADGQVIGDQNNIVGLGLGSDNDIIVNATTLYAGIGTINDNQGTVTLSGGIPNTPGTV

Candidatus R. tasmanensis GIPFNDAGNTIPLTIKRSVG-NETATG-FDVPSVIVLGVDSVIADGQVIGDQNNIVGLGLGSDNGIIVNATTLYAGIGTINNNQGTVTLSGGVPNTPGAV

Rickettsia sp. IG-1 GIPFNDAGNTIPLTIKSTVG-NKTATG-FAVPSVIVLGVDSVIADGQVIGDQNNIVGLGLGSDNGIIVNATTLYAGIGTINNNQGTVTLSGGVPNIPGTV

Candidatus R. barbariae ----------------------------------------------------------------------------------------------------

Rickettsia sp. S GIPFNDAGNIMPLTIKSTVG-NKTATG-FDVPSVVVLGVDSVIADGQVIGDQNNIVGLGLGSDNDIIVNATTLYAGIGTVNNNQGTVTLSGGIPNTPGTV

Candidatus R. goldwasserii GIPFNDAGNTIPLTIKSTVG-NKTATG-FNVPSVIVLGVDSFIADGQVIGDQDNIVGLGLGSDNGIIVNATTLYAGIGTINNNQGTVTLSGGVPNTPGTV

Rickettsia mongolotimonae GIPFNDAGNIMPLTIKSTVG-NKTATG-FDVPSVIVLGVDSVIADGQVIGDQNNIVGLGLGSDNDIIVNATTLYAGIGTINNNQGTVTLSGGIPNTPGTV

Rickettsia sp. BJ-90 GIPFNDAANIIPLT-KSTVG-NKTATG-FDVPSVIVLGVDSVIADGQVIGDQNNIVGLGLGSDNDIIVNATTLYAGIGTINNNQGTVTLSGGIPNTPGTV

Candidatus R. andeanae GMPFNDAGNIIPLTIKSTVG-NETATG-FDVPRVIVLGVDSVIADGQVIGDQNNIVGLGLGSDNGIIVNATTLYAGIGTINNNQGTVTLSGGIPNTPGTV

Rickettsia sp. Bar29 GIPFNDAGNTMPLTIKSTVG-NRTATG-FDVPKVIVLGVDSVIADGQVIGDQNNIVGLGLGSDNGIIVNATTLYAGIGTINNNQGTVTLSGGVPNAPGTV

Rickettsia sp. TwKM01 GIPFND------VTIKSTVG-NKTATG-FDVPKVIVLGVDSVIADGQVIGDQNNIVGLGLGSDNGIIVNATTLYAGIGTINNNQGTVTLSGGIPNTPGTV

R. raoultii GIPFNDAGNTMPLTIKSTVG-NKTATG-FDVPGVIVLGVDSVIADGQVIGDQNNIVSLGLGSDNGIIVNATTLYAGIGTINNNQGTVTLSGGVPNTPGTV

R. asiatica GLPFNDAGNTIPLTIKSTVG-NGPEGD-FNVPRVIVSGIDSVIADGQVIGDQDNIIGLGLGSDNGIIVNATTLYAGIGTVNDNQGTVTLSGGVPNTPGTI

Rickettsia sp. 110 SLAFDG------LTITSTVK-NGPAGD-FDAPFVVVSGVDSTIANGQAIGDQNNIVGLDLASDNSITVNATTLYAGIATTEDNQGTVTLSGGVPNTPGAI

Rickettsia sp. mmx79 SLAFDG------LTITSTVK-NGPAGD-FDAPFVVVSGVDSTIANGQAIGDQNNIVGLDLASDNSITVNATTLYAGIATTEDNQGTVTLSGGVPNTPGAI

Rickettsia sp. 103 GIAFDG------LTITSTVK-NGPAGD-FDAPFVVVSGVDSTIANGQAIGDQNNIVGLGLASDNSITVNATTLYA-IATTEDNQGTVTLSGGVPNTPGAI

Rickettsia sp. J52 SIAFDV------LTITSTVK-NGPAGD-FDAPFVVVSGVDSTIANGQAIGDQNNIVGLGLASDNSITVNATTLYACIATTEDNQGTVTLSVGVPNTPGAI

Candidatus R. hoogstraalii GILLDG------LTIKSTVG-NKVAGGGFNAPRLIVTNVDSTIAPGQVIGDQNNIIGLGLASDNTITVNDATVYATLFAVNNDEGTIELNGGVPNTPGTI

Rickettsia sp. California 2 GIPLDG------LTIKSTVG-NETATGNFDVPRLIVSGVDSVIADGQAIGDQDNIVGLGLGSDNSITVNATKLYAGIGSVNNNQGTVTLSGGIPNTPGTI

R. hulinensis GIPFNDAGNIIPLTIKSTVG-NETAEG-FNVPSVIVSGVDSVIADGQVIGDQNNIVGLGLGSDNGIIVNATTLYAGIGTINNNQGTVTLSGGVPNTPGTV

**[1101-1200 ]**

**[ | | | | | | | | | | ]**

R. bellii RML369-C ----------------------------------------------------------------------------------------------------

R. bellii OSU 85-389 ----------------------------------------------------------------------------------------------------

R. prowazekii Madrid E YGLGL---ENG--SPKLKQVTFTTDYNNLGSIIANNVTINDYVTLTT-----GGIAGTDFDAKITLGSVNGNANVRFVDSTFSDPRSMIVATQANKGTVT

R. prowazekii Rp22 YGLGL---ENG--SPKLKQVTFTTDYNNLGSIIANNVTINDDVTLTT-----GGIAGTDFDAKITLGSVNGNANVRFVDSTFSDPRSMIVATQANKGTVT

**R. typhi Wilmington YGLGL---ENG--DPKLKQVTFTTDYNNLGSIIATNVTINDDVTLTT-----GGIAGTDFDGKITLGSINGNANVKFVDRTFSHPTSMIVSTKANQGTVT**

R. akari Hartford YGLGV---GSD--SPKLKQVTFATDYNNLGSIVATNVTINDGVTVTT-----GGVVGTDFDGTITLGSVNGNANVRFVDSTFSSPASMIVTTEANNGTVT

R. australis YGLGV---EHG--YPKLKQVTFTTDYNNLGSIIATNVTINDGVTVTT-----GGVVGTDFDGTITLGSVNGNANVRFVDRTFSDPTSMIVTTKANVGTVT

R. felis URRWXCal2 YGLGI---ENG--SPKLKQVTFTTDYNNLGSIIATNATINDGVTVTT-----GGVAGTDFDGKITLGSVNGNANVRFVDGTFSDSTSMIVTTKANNGTVT

R. canadensis McKiel YGLGIKPEGEA--ALKLKLVTVATDYKNLGSTFVNNLIVNDGLTYQA-----GGVVGKDFDGTITLGSANGNSNVIFADGTNSTSTSTVATVKANNGVVT

R. tamurae YGLGI---ENG--TPKLKQVTFTTDYNNLGSIIATNATINDGVTVTT-----GGIAGTDFDGKITLGSANGNANVRFADGTFSDPTSMIVTTKANNGTVT

R. helvetica YSLGI---GNG--TPKLKQVTFTTNYNNLGSIIATNATINDGLTVTT-----GGIAGKDFDGKITLGSANGNSNVIFVDGTNSTATSMVATAKANNGTVT

REIS YGLGI---ENG--TLKLKQVTFTTNYNNLGSIIATNATINDGVTVTT-----GGIAGTDFDGKITLGSINGNANVRFADGTFSDSTSMIVTTKANSGTVT

R. monacensis YGLGI---ENG--TLKLKQVTFTTNYNNLGSIIATNAIINDGVTVTT-----GGIAGTDFDGKITLGSVNGNANVRFADGTFSDSTSMIVTTKANSGTVT

R. massiliae MTU5 YGLGT---GIG--ASQLKQVTFTTDYNNLGNIIAANATINDGVTVTT-----GGIAGTDFDGKITLGSVNGNANVRFADGIFSNSTSMIVTTKANNGTVT

Candidatus R. amblyommii YGLGT---GIG--ASQLKQVTFTTDYNNLGNIIATNATINDGVTVTT-----GGIAGTDFDGKITLGSVNGNANVRFADGILSNSTSMIVTTKANNGTVT

R. rhipicephali YGLGT---GIG--ASQLKQVTFTTDYNNLGNIIATNATINDGVTVTT-----GGIAGTDFDGKITLGSVNGNANVRFADGIFSNSTSMIVTTKANNGTVT

R. aeschlimannii YGLGT---GIG--SSQLKQVTFTTDYNNLGNIIATNATINDGVTVTT-----GGIAGTDFDGKITLGSVNGNANVRFADGIFSNSTSMIVTTKANNGTVT

R. montanensis YGLGK---DAS--ASQLKQVTFTTDYNNLGNIIATNTTINDGVTVTT-----GGIAGTVFNGKITLGSVNGNANVRFADGILSNSTSMIVTTKANNGTVT

R. slovaca 13-B YGLGT---GIS--PSKFKQVTFTTDYNNLGNIIATNATINDGVTVTT-----GGIAGTSFNGKITLGSVNGNGNVRFADGILSNSTSMIGTTKANNGTVT

R. peacockii Rustic YGLGT---GIG--ASKFKQVTFTTDYNNLGNIIATNATINDGVTVTT-----GGIAGTGFDGKITLGSVNGNGNVRFADGILSNSTSMIGTTKTNNGTVT

R. rickettsii Iowa YGLGT---GIG--ASKFKQVTFTTDYNNLGNIIATNATINDGVTVTT-----GGIAGIGFDGKITLGSVNGNGNVRFADGILSNSTSMIGTTKANNGTVT

R. rickettsii Sheila Smith YGLGT---GIG--ASKFKQVTFTTDYNNLGNIIATNATINDGVTVTT-----GGIAGIGFDGKITLGSVNGNGNVRFADGILSNSTSMIGTTKANNGTVT

R. japonica YGLGT---GIG--ASKFKQVTFTTDYNNLGNIIATNTTINDGVTVTTGGIAAGGIAGTDFDGKITLGSVNGNANVRFADGIFSNSTSMIVTTKANNGTVT

R. honei YGLGT---GIG--HSKFKQVTFTTDYNNLGNIIATNATINDGVTVTT-----GGIAGTGFDGKITLGSVNGNGNVRFVDGMLSNSTSMIGTTKANNGTVT

R. conorii str. Malish 7 YGLGT---GIG--ASKFKQVTFTTDYNNLGNIIATNATINDGVTVTT-----GGIAGIGFDGKITLGSVNGNGNVRFVDGILSHSTSMIGTTKANNGTVT

R. sibirica 246 YGLGT---GIG--ASKFKQVTFTTDYNNLGNIIATNATINDGVTVTT-----GGIAGIGFDGKITLGSVNGNGNVRFVDGILSNSTSMIGTTKANNGTVT

R. africae ESF-5 YGLGT---GIG--ASKFKQVTFTTDYNNLGNIIATNATINDGVTVTT-----GGIAGIGFDGKITLGSVNGNGNVRFVDGILSNSTSMIGTTKANNGTVT

R. parkeri YGLGT---GIG--ASKFKQVTFTTDYNNLGNIIATNATINDGVTVTT-----GGIAGIGFDGKITLGSVNGNGNVRFVDGILSNSTSMIGTTKANNGTVT

R. heilongjiangensis YGLGT---GIR--ASQFKQVTFTTDYNNLGNIIATNTTINDGVTVTT-----GGIAGTDFDGKITLGSVNGNANVIFADGIFSNSTSMIVTTKANNGTVT

Israeli tick typhus YGLGT---GIG--ASKFKQVTFTTDYNNLGNIIATNATINDGVTVTT-----GGIAGIGFDGKITLGSVNGNGNVRFVDGILSNSTSMIGTTKANNGTVT

Rickettsia sp. A-167 YGLGA---GIG--ASKFKQVTFTTDYNNLGNIIATNATINDGVTVTT-----GGIAGIGFDGKITLGSVNGNGNVRFVDGILSNSTSMIGTTKANNGTVT

Candidatus R. tasmanensis YGLGT---GIG--ASQLKQVTFTTDYNNLGNIIATNATINDGVTVTT-----GGIAGTDFDDKITLGSVNGNANVRFADGIFSNSTSMIVTTKANNGTVT

Rickettsia sp. IG-1 YGLGT---GIG--DSKFKQVTFTTDYNNLGNIIATNATINDGVTVTT-----GGIAGTGFDGKITLGSVNGNGNVRFADGILSNSTSMIGTTKANNGTVT

Candidatus R. barbariae ----------------------------------------------------------------------------------------------------

Rickettsia sp. S YGLGT---GIG--ASKFKQVTFTTDYNNLGNIIATNATINDGVTVTT-----GGIAGIGFDGKITLGSVNGNGNVRFVDGILSNSTSMIGTTKANNGTVT

Candidatus R. goldwasserii YGLGT---GIG--TSQFKQVTFTTDYNNLGNIIATNAIINDGVTVTT-----GGIAGTDFDGKITLGSVNGNANVIFADGIFSNSTSMIGTTKANNGTVT

Rickettsia mongolotimonae YGLGT---GIG--ASKFKQVTFTTDYNNLGNIIATNATINDGVTVTT-----GGIAGIGFDGKITLGSVNGNGNVRFVDGILSNSTSMIGTTKANNGTVT

Rickettsia sp. BJ-90 YGLGT---GIG--ASKFKQVTFTTDYNNLGNIIATNATINDGVTVTT-----GGIAGIGFDGKITLGSVNGNGNVRFVDGILSNSTSMIGTTKANNGTVT

Candidatus R. andeanae YGLGT---CIGASASQLKQVTFTTDYNNLGHIIATNATINDGVTVTT-----GGIAGTGFDGKITLGSVNGNANVRFADGIFSNSTSMIVTTKANNGTVT

Rickettsia sp. Bar29 YGLGT---GIG--ASQLKQVTFTTDYNNLGNIIAANATINDGVTVTT-----GGIAGTDFDGKITLGSVNGNANVRFADGIFSNSTSMIVTTKANNGTVT

Rickettsia sp. TwKM01 YGLGT---GIG--ASQLKQVTFTTDYNNLGNIIATNATINDGVTVTT-----GGIAGTDFDGKITLGSVNGNANVRFADGIFSNSTSMIVTTKANNGTVT

R. raoultii YGLGT---GIG--TVQLKQVTFTTDYNNLGNIIATNATINDGVTVTT-----GGIAGTDFDGKITLGSVNGNANVRFADGILSNSTSMIVTTKANNGTVT

R. asiatica YGLGI---GNG--TPKLKQVTFTTDYNNLGSIIATNATINDGVTVTT-----GGIAGTDFDGKITLGSVNGNANVRFADGTFSDPTSMIVTTKANNGTVT

Rickettsia sp. 110 YGLGL---ENG--TPKLKQVTFTTDYNNLGSIIATNAIINDGLTVTT-----GGIAGKDFDGTITLGSANGNSNVRFVDGTNSTSTSTVATVKANNGTVT

Rickettsia sp. mmx79 YGLGL---ENG--TPKLKQVTFTTGYNNLGSIIATNAIINDGLTVTT-----GGIAGKDFDGTITLGSANGNSNVRFVDGTNSTSTSTVATVKANNGTVT

Rickettsia sp. 103 YGLGL---ENG--TPKLKQVTFTTDYNNLGSIIATNAIINDGLTVTT-----GGIAGKDFDGTITLGSANGNSNVRFVDGTNSTSTSTVATVKANNGTVT

Rickettsia sp. J52 YGLGF-----G--TPKLKQVTFTTDYNNLGSIIATNAIINDGLTVTT-----GGIAGKNFDGTITLGSANGNSNVRFVDGTNSTSTSTVATVKANNGTVT

Candidatus R. hoogstraalii YGLGF---KNG--DPKLEEVNFTTNYNNLGSIIATNAIIEDGVTVTT-----GGTAGKDFDGKITLGSVNGNSNVIFVDGTNSTATSMVSTVKANNGTVT

Rickettsia sp. California 2 YGLGI---ENG--SPKLKQVTFTTDYNNLGSIIATNATINDGVTVTT-----GGVAGTDFDGKITLGSVNGNANVRFVDGTFSDSTSMIVTTKANNGTVT

R. hulinensis YGLGT---GIG--ASQFKQVTFTTDYNNLGNIIATNTTINDGVTVTT-----GGIAGTDFDGKITLGSVNGNANVRFADGIFSNSTSMIVTTKANNGTVT

**[1201-1300 ]**

**[ | | | | | | | | | | ]**

R. bellii RML369-C ---------------------------------------------------------------GSVNI-NGEINLGANVLGFTDGISTWGNDTTISTTLT

R. bellii OSU 85-389 ---------------------------------------------------------------GSVNI-NGEINLGANVLGFTDGISTWGNDTTISTTLT

R. prowazekii Madrid E YLGNALVSNIGSLDTPVASVRF----TGNDSG-AGLQGNIYSQNIDFGTYNLTILNSNVILGGGTTAI-NGEIDLLTNNLIFANGTSTWGDNTSISTTLN

R. prowazekii Rp22 YLGNALVSNIGSLDTPVASVRF----TGNDSG-AGLQGNIYSQNIDFGTYNLTILNSNVILGGGTTAI-NGEIDLLTNNLIFANGTSTWGDNTSISTTLN

**R. typhi Wilmington YLGNALVGNIGSSDIPVASVRF----TGNDSG-VGLQGNIHSQNIDFGTYNLTILNSDVILGGGTTAI-NGEIDLLTNNLIFANGTSTWGNNTSLSTTLN**

R. akari Hartford YLGDAYVGNIGASDANIASVKF----TGNHNG-AGLQGNIYSQVTDFGTYTLTVLNSNVILGGGTTAI-NGAIDLLTNTLTFASGTSTWGSNTSIETTLT

R. australis YLGNAHVGNIGAADTNVASVKF----TGNDNG-AGLQGNIYSQVTDFGAYDLTVLNSNVILGGGTTAI-NGEIDLLTNTLTFASGTSTWGSNTSIETTLT

R. felis URRWXCal2 YLGSALVGNIGSSDTPVASVKF----IGSDDG-AGLQGNIYSQVTDFGTYDLSVLNSNVILGGGTTAI-NGEIDLLTNTLTFASGTSTWGSNTSIETTLT

R. canadensis McKiel YLGSAFVGNIGCSNTPVASVQF----TGPVGSLEKLQGNIYSTATNFGNVNLNVAASNVILGGGTTAI-NGNIDLATNILTFASGTSTWGDNTSLSTTLT

R. tamurae YLGDAYVGNIGASGTPVASVKF----TGNDNG-AGLQGNIYSQVTDFGTYGLDVLNSNVILGGGTIAI-NGEIDLDTNILTFASGTSTWGSNTSIETTLT

R. helvetica YLGSAAVGNIGSSNALVASVKF----TGPAGSLEKLQGNIYSTATNFGNVNLNVAGSNIILGGDTTAI-NGNINLVTNTLTFESGTSTWGSNTSLSTTLT

REIS YLGDAYVGSIGASDTPVASVKF----TGNDNG-AGLQGNIYSQVTDFGTYNLGVLNSNVILGGGTTAI-NGEIDLDTNILTFASGTSTWGSNTSIETTLT

R. monacensis YLGDAYVGNIGASDTPVASVKF----TGNDNG-AGLQGNIYSQVTDFGTYNLGVLNSNVILGGGTTAI-NGEIDLDTNILTFASGTSTWGSNTSIETTLT

R. massiliae MTU5 YLGNACVGNIGDSDTPVASVRF----TGSDDS-AGLQGNIYSQVIDFGTYDLNILNSNVILGGGTTAI-NGAINLLTNTLTFASGTSTWGNNTSIKTTLT

Candidatus R. amblyommii YLGNACVGNIGDSDTPVASVRF----TGSDDG-AGLQGNIYSQVIDFGTYDLSILNSNVILGGGTTAI-NGAIDLLTNTLTFASGTSTWGNNTSIETTLT

R. rhipicephali YLGNACVGNIGDSNTPVASVRF----TGSDDG-AGLQGNIYSQVIDFGTYDLNILNSNVILGGGTTAI-NGAINLLTNTLTFASGTSTWGNNTSIKTTLT

R. aeschlimannii YLGNAFVGNIGDLGTPVASVRF----TGSDDG-AGLQGNIYSQVIDFGTYDLGILNSNVILGGGTTAI-NGAVNLLTNTLTFASGTSTWGNNTSIKTTLT

R. montanensis YLGGAYVGSIGASNTPVASVRFTGSGTGSDNG-AVLQGNIYSQVIDFGTYDLDIVNSNVILGGGTTAI-NGAIDLCTNTLTFASGTSTLGNNTSIETTLT

R. slovaca 13-B YLGNALVGNIGDSDTPVASVRF----TGSDGN-ARLQGNIYSQVIDFGTYNLDILNSNVILGGGTTAI-NGKINLLTNTLTFASGTSTWGNNTSIETTLT

R. peacockii Rustic YLGNAFVGNIGDSDTPVASVRF----TGSDGG-AGLQGNIYSQVIDFGTYNLGILNTNIILGGGTTAI-NGKINLVTNTLTFASGTSTWGNNTSIETTLT

R. rickettsii Iowa YLGNAFVGNIGDSDTPVASVRF----TGSDSG-AGLQGNIYSQVIDFGTYNLGIVNSNIILGGGATAI-NGKIDLVTNTLTFASGTSTWGNNTSIETTLT

R. rickettsii Sheila Smith YLGNAFVGNIGDSDTPVASVRF----TGSDSG-AGLQGNIYSQVIDFGTYNLGIVNSNIILGGGTTAI-NGKIDLVTNTLTFASGTSTWGNNTSIETTLT

R. japonica YLGNAFVGNIGDSDTPVASVRF----TGSNNG-AGLKGNIYSQVIDFGTYNLGIVNSNVILGGSTTAI-NGKIDLLTNTLTFAGGTSTWGNNTSIETTLT

R. honei YLGNAFVGNIGDSDTPVASVRF----TGSDSG-AGLQGNIYSQVIDFGTYSLGILNSNVILGGGTTAI-NGKISLLTNTLTFASGTSTWGNNTSIETTLT

R. conorii str. Malish 7 YLGNAFVGNIGDSDTPVASVRF----TGSDGG-AGLQGNIYSQVIDFGTYNLGISNSNVILGGGTTAI-NGKINLRTNTLTFASGTSTWGNNTSIETTLT

R. sibirica 246 YLGNAFVGNIGDSDTPVASVRF----TGSDGG-AGLQGNIYSQVIDFGTYNLGILNSNVILGGGTTAI-NGKINLLTNTLTFASGTSTWGNNTSIETTLT

R. africae ESF-5 YLGNAFVGNIGGSDTPVASVRF----TGSDGG-AGLQGNIYSQVIDFGTYNLGILNSNVILGGGTTAI-NGKINLLTNTLTFASGTSTWGNNTSIETTLT

R. parkeri YLGNAFVGNIGDSGTPVASVRF----TGSDGG-AGLQGNIYSQVIDFGTYNLGILNSNVILGGGTTAI-NGKINLLTNTLTFASGTSTWGNNTSIETTLT

R. heilongjiangensis YLGNAFVGNIGDSDTPVASVRF----TGSNNG-AGLQGNIYSQVIEFGTYNLGIVNSNVILGGSTTAI-NGKIDLLTNTLTFAGGTSTWGNNTSIETTLT

Israeli tick typhus YLGNAFVGNIGDSNTPVASVSF----TGSDGG-AGLQGNIYSQVIDFGTYNLGILNSNVILGGGTTAI-NGKINLRTNTLTFASGTSTWGNNTSIETTLT

Rickettsia sp. A-167 YLGNAFVGNIGDSNTPVASVRF----TGSDGG-AGLQGNIYSQVIDFGTYNLGILNSNVILGGGTTAI-NGKINLRTNTLTFASGTSTWGNNTSIETTLT

Candidatus R. tasmanensis YLGNALVGNIGDSDTPVASVRF----TGSDDG-AGLQGNIYSQVIDFGTYDLGILNSNVILGGGTTAI-NGAIDLLTNTLTFASGTSTWGNNTSIETTLT

Rickettsia sp. IG-1 YLGNAFVGNIGDSDTPVASVRF----TGSDGD-ARLQGNIYSQVIDFGTYSLGILNSNVILGGGTTAI-NGKINLLTNTLTFASGTSTWGNNTSIETTLT

Candidatus R. barbariae ----------------------------------------------------------------------------------------------------

Rickettsia sp. S YLGSAFVGNIGDSNTPVASVRF----TGSDGS-AGLQGNIYSQVIDFGTYNLGILNSNVILGGGTTAI-NGKINLSTNTLTFASGTSTWGNNTSIETTLT

Candidatus R. goldwasserii YLGNALVGSIGDSDTPVASVRF----TGSNGG-TGLQGNIYSQVIDFGTYNLGILNSNVILGGGTTAI-NGKIGLLTNTLTFASGTSTWGNNTSIETTLT

Rickettsia mongolotimonae YLGNAFVGNIGDSDTPVASVRF----TGSDGG-AGLQGNIYSQVIDFGTYNLGILNSNVILGGGTTAI-NGKINLLTNTLTFASGTSTWGNNTSIETTLT

Rickettsia sp. BJ-90 YLGNAFVGNIGDSDTPVASVRF----TGSDGG-AGLQGNIYSQVIDFGTYNLGILNSNVILGGGTTAI-NGKINLLTNTLTFASGTSTWGNNTSIETTLT

Candidatus R. andeanae YLGNALVGNIGDSGTPVASVRF----TGSDDG-AGLQGNIYSQVIDFGTYDLGILNSNVILGGGTTAI-NGAINLLTNTLTFASGTSTWGNNTSINTTLT

Rickettsia sp. Bar29 YLGNACVGNIGDSGTPVASVRF----TGSDDSAAGLQGNIYSQVIDFGTYDLNILNSNVILGGGTTAI-NGAINLLTNTLTFASGTSTWGNNTSIKTTLT

Rickettsia sp. TwKM01 YLGNACVGNIGDSDAPVASVRF----TGSDDG-AGLQGNIYSQVIDFGTYDLNILNSNVILGGGTTAINNGAINLLTNTLTFASGTSTWGNNTSIKTTLT

R. raoultii YLGNAFVGNIGDSDTPVASVRF----TGSDKG-AGLQGNIYSQVIDFGTYDLDILNSNVILGGGTTAI-NGAINLLTNTLTFASGTSTWGNNTSIETTLT

R. asiatica YLGSAAVGNIGSSNALVASVKF----TGPAGSLEKLQGNIYSTATNFGNVNLNVAGSNIILGGGTIAI-NGEIDLDTNILTFASGTSTWGSNTSIETTLT

Rickettsia sp. 110 YLGSASVGNIGAANTPVASVQF----TGPAGSLEKLLGNIYSTATNFGNVNLNVAGSNVILGGGTTAI-NGNINLVTNTLTFASGTSTWGGNTSLTTDLT

Rickettsia sp. mmx79 YLGSASVGNIGAANTPVASVQF----TGPAGSLEKLLGNIYSTATNFGNVNLNVAGSNVILGGGTTAI-NGNINLVTNTLTFASGTSTWGGNTSLTTDLT

Rickettsia sp. 103 YLGSASVGNIGAANTPVASVQF----TGPAGSLEKLLGNIYSTATNFGNVNLNVAGSNVILGGGTTAI-NGNINLVTNTLTFASGTSTWGGNTSLTTDLT

Rickettsia sp. J52 YLGSASVGNIGAANTPVASVQF----TGPAGSLEKLLGNIYSTATNFGNVNLNVAGSNVILGGGTTAI-NGNINLVTNTLTFASGTSTWGGNTSLTTDLT

Candidatus R. hoogstraalii YLGRASVGNIGSSDTPVASVKF----TGPAGSLEKLLGNIYSTATNFGNVNLNVAGSNVTLGGGTTAI-NGNIDLVTNTLTFASGTSTWGSNTSLSTTLN

Rickettsia sp. California 2 YLGSALVGNIGSSDTPVASVKF----IGSDDG-AGLQGNIYSQVTDFGTYDLSVLNSNVILGGGTTAI-NGEIDLLTNTLTFASGTSTWGSNTSIETTLT

R. hulinensis YLGNAFVGNIGDSDTPVASVRF----TGSNNG-AGLKGNIYSQVIDFGTYNLGIVNSNVILGGSTTAI-NGKIDLLTNTLTFAGGTSTWGNNTAIETTLT

**[1301-1400 ]**

**[ | | | | | | | | | | ]**

R. bellii RML369-C S----DEVMGNIVISGAAEVK--IAGLKTIKVQDNASIDFIGTKTYTLIQGDISADNGI-DPN--VVGTNRYVIYGL--SGNTEALVTRTIDINNIENIL

R. bellii OSU 85-389 S----DEVMGNIVISGAAEVK--IAGLKTIKVQDNASIDFIGTKTYTLIQGDISADNGI-DPN--VVGTNRYVIYGL--SGNTEALVTRTIDINNIENIL

R. prowazekii Madrid E VS---SGNIGQVVIAEDAQVNATTTGTTTIKIQDNANANFSGTQAYTLIQGGARFNGTLGAPNFAVTGSNIFVKYELIRDSNQDYVLTRT---NDVLNVV

R. prowazekii Rp22 VS---SGNIGQVVIAEDAQVNATTTGTTTIKIQDNANANFSGTQAYTLIQGGARFNGTLGAPNFAVTGSNIFVKYELIRDSNQDYVLTRT---NDVLNVV

**R. typhi Wilmington VS---NGNVGQIVIAEGAQVNATTTGTTTIKIQDNANANFSGTQTYTLIQGGARFNGTLGAPNFDVTGNNIFVKYELIRDANQDYVLTRT---NDVLNVV**

R. akari Hartford VA---NGNMGHIVIAENAQVNATDTGATTINVQDNANANFSGTKTYILIQGGARFNGTLGGPNFAVTGSNRFVNYALIRDANQDYVITRT---NNAANVV

R. australis VA---NGKIGHIVIAENAQVNATTTGTTTINVQDNVNANFSGTKTYILIQGGARFNGTLGGPNFAVTGSNRFVNYGLMRDANQDYVITRT---NDAANVV

R. felis URRWXCal2 VA---NGNIGHIVIAENAQVNATTTGTTTINVQDNANANFSDTQTYTLIQGGARFNGTLGGPNFAVTGSNRFVNYGLIRAANQDYVITRT---NNAANVV

R. canadensis McKiel LA---SGNIGHIVIAEGVQVNATTTGTTTINVQDSANANFSGTQTYTLIQGGARFSGTLGDPKFNVTGSNIFVRYGLIRADNKDYVLTRT---NDVANVV

R. tamurae VA---NGNIGHIVIAEGAQVNATTTGTTTINVQDNANTNFSGTKYYTLIEGGARFNGTLRDPNFAVTGDNRFIKYGLIRDANQDYVITRT---NNAANVV

R. helvetica VS---NGNIGHIVIAEGAQVNVTTIGTTTINVQDNASANFSGTKYYTLIEGGARFNGTLRDPNFAVTGSNRFVNYGLIRAANQDYVVTRT---NDAANVV

REIS VA---NGNIGHIVIAEDAQVNTTTTGTTTINVQDNANANFSGTQTYTLIQGGARFSGTLGGPNFAVTGNNRFVKYGLIRDANQDYVITRT---NNAANVV

R. monacensis VA---NGNIGHIVIAEGAQVNATTTGTTTINVQDNTNANFSGTQTYTLIQGGARFNGTLGGPNFAVTGNNRFVKYGLIRDANQDYVITRT---NNAANVV

R. massiliae MTU5 VA---NGNIGHIVIAEGAQVNATTTGTTTINVQDNANANFSGTQTYTLIQGGARFNGTLGGPNFAVTGSNRFVNYGLIRAANQDYVITRT---NNAENVV

Candidatus R. amblyommii LA---NGNIGHIVITEGAQVNATTTGTTTIKVQDNANANFSGTQTYTLIQGGARFNGTLGGPNFAVTGSNRFVNYGLIRAANQDYVITRT---NNVENVV

R. rhipicephali VA---NGNIGHIVIAEGAQVNATTTGTTTINVQDNTNANFSGTQTYTLIQGGARFNGTLGGPNFAVTGSNRFVNYGLIRAANQDYVITRT---NNAENVV

R. aeschlimannii VA---NGNIGHIVIAEGAQVNATTTGTTTINVQDNANANFSGTQTYTLIEGGARFNGTLGVPNFAVTGSNRFVNYGLIRAANQDYIITRT---NNAENVV

R. montanensis LA---DGNIGHIVIAEGARVNATTTGTTTINVQDKANANFSGTQTYTLIQGGARFNGTLGDPDFAVTGSNRFVNYGLIRAANQDYVITRT---NNVENVV

R. slovaca 13-B LA---NGNIGHIVILEGAQVNATTTGTTTIKVQDNANANFSGTQTYTLIQGGARFNGTLGGPNFAVTGNNRFVNYGLIRAANQDYVITRT---NNAENVV

R. peacockii Rustic LA---NGNIGHIVILEGAQVNATTTGTTTIKVQDNANANFSGTQTYTLIQGGARFNGTLGGPNFAVTGSNRFVNYGLIRAANQDYVITRT---NNAENVV

R. rickettsii Iowa LA---NGNIGHIVILEGAQVNTTTTGTTTIKVQDNANANFSGTQTYTLIQGGARFNGTLGSPNFAVTGSNRFVNYSLIRAANQDYVITRT---NNAENVV

R. rickettsii Sheila Smith LA---NGNIGHIVILEGAQVNTTTTGTTTIKVQDNANANFSGTQTYTLIQGGARFNGTLGSPNFAVTGSNRFVNYSLIRAANQDYVITRT---NNAENVV

R. japonica LA---NGNIGHIVIAEGAQVNATTTGTTTINVQDNANANFSGTQTYTLIQGGARFNGTLGGPNFTVTGSNRFVNYGLIRAANQDYVITRT---NNAENIV

R. honei LA---NDNIGHIVILEGAQVNATTTGTTTIKVQDNANANFSGTQTYTLIQGGARFNGTLGGPNFAVTGSNRFVNYGLIRAANQDYVITRT---NNAENVV

R. conorii str. Malish 7 LA---NGNIGNIVILEGAQVNATTTGTTTIKVQDNANANFSGTQTYTLIQGGARFNGTLGGPNFVVTGSNRFVNYGLIRAANQDYVITRT---NNAENVV

R. sibirica 246 LA---NGNIGNIVILEGAQVNATTTGTTTIKVQDNANANFSGTQTYTLIQGGARFNGTLGGPNFVVTGSNRFVNYGLIRAANQDYVITRT---NNAENVV

R. africae ESF-5 LA---NVNIGNIVILEGAQVNATTTGTTTIKVQDNANANFSGTQTYTLIQGGARFNGTLGGPNFVVTGSNRFVNYGLIRAANQDYVITRT---NNAENVV

R. parkeri LA---NGNIGNIVILEGAQVNATTTGTTTIKVQDNANANFSGTQTYTLIQGGARFNGTLGGPNFVVTGSNRFVNYGLIRAANQDYVITRT---NNAENVV

R. heilongjiangensis LA---NGNIGHIVIAEGAQVNATTTGTTTINVQDNANANFSGTETYTLIQGGARFNGTLGGPNFTVTGSNRFVNYGLIRVANQDYVITRT---NNAENVV

Israeli tick typhus LA---NGNIGNIVILEGAQVNATTTGTTTIKVQDNANANFSGTQTYTLIQGGARFNGTLGGPNFVVTGSNRFVNYGLIRAANQDYVITRT---NNAENVV

Rickettsia sp. A-167 LA---NGNIGNIVILEGAQVNATTTGTTTIKVQDNANANFSGTQTYTLIQGGARFNGTLGGPNFVVTGSNRFVNYGLIRAANQDYVITRT---NNAENVV

Candidatus R. tasmanensis LA---NGNIGHIVIAEGAQVNATTTGTTTINVQDNANANFSGTQTYTLIQGGARFNGTLGGPNFAVTGSNRFVNYGLIRAANQDYVITRT---NNAENVV

Rickettsia sp. IG-1 LA---NGNIGHIVILEGAQVNATTTGTTTIKVQDNANANFSGTQTYTLIQGGARFNGTLGGPNFAVTGSNRFVNYSLIRAANQDYIITRT---NNAENVV

Candidatus R. barbariae ----------------------------------------------------------------------------------------------------

Rickettsia sp. S LA---NGNIGNIVILEGAQVNATTTGTTTIAVQDNANANFSGTQTYTLIQGGARFNGTLGGPNFVVTGSNRFVNYGLIRAANQDYVITRT---NNAENVV

Candidatus R. goldwasserii LA---NGNIGHIVIVEGAQVNATTTGTTTINIQDNANANFSGTQTYTLIQGGARFNGTLGGPNFAVTGSNRFVNYGLIRAANQDYVITRT---NNAENVV

Rickettsia mongolotimonae LA---NGNIGNIVILEGAQVNATTTGTTTIKVQDNANANFSGTQTYTLIQGGARFNGTLGGPNFVVTGSNRFVNYGLIRAANQDYVITRT---NNAENVV

Rickettsia sp. BJ-90 LA---NGNIGNIVILEGAQVNATTTGTTTIKVQDNANANFSGTQTYTLIQGGARFNGTLGGPNFVVTGSNRFVNYGLIRAANQDYVITRT---NNAENVV

Candidatus R. andeanae LA---NGNIGHIVIAEGAQVNATTTGTTTITVQDNANANFSGTQTYTLIQGGARFNGTLGGPNFVVTGSNRFVNYGLIRAANQDYVITRT---NNAENVV

Rickettsia sp. Bar29 VA---NGNIGHIVIAEGAQVNATTTGTTTINVQDNANANFSGIQTYTLIQGGARFNGTLGGPNFAVTGSNRFVNYGLIRAANQDYVITRT---NNAENVV

Rickettsia sp. TwKM01 VA---NGNIGHIVIAEGAQVNATTTGTTTINVQDNANANFSGTQTYTLIQGGARFNGTLGGPNFAVTGSNRFVNYGLIRAANQDYVITRT---NNAENVV

R. raoultii VA---NGNIGHIVIAEGAQVNATTTGTTTINVQDNANANFSGTQTYTLIQGGARFNGTLGGPNFAVTGSNRFVNYGLIRAANQDYVITRT---NNAENVV

R. asiatica VA---NGNIGHIVIAEGAQVNATTTGTTTINVQDNASANFSGTKYYTLIEGGARFNGTLRGPNFAVTGSNRFINYGLIRAANQDYVVTRT---NDAANVV

Rickettsia sp. 110 VS---NGNIGHIVIAEGAQVNATTTGTTTINVQDSANANFSGTQTYTLIQGGARFNGTLGDPKFNVTGSNIFVKYGLIRAANQDYVLTRT---NDATNVV

Rickettsia sp. mmx79 VS---NGNIGHIVIAEGAQVNATTTGTTTINVQDSANANFSGTQTYTLIQGGARFNGTLGDPKSNVTGSNIFVKYGLIRAANQDYVLTRT---DDATNVV

Rickettsia sp. 103 VS---NGNIGHIVIAEGAQVNATATGTTTINVQDSANANFSGTQTYTLIQGGARFNGTLGDPKFNVTGSNIFVKYGLIRAANQDYVLTRT---NDATNVV

Rickettsia sp. J52 VS---NGNIGHIVIAEGAQVNATTTGTTTINVQDSANANFSGTQTYTLIQGGARFNGTLGDPKFNVTGSNIFVKYGLIRAANQDYVLTRT---NDATNVV

Candidatus R. hoogstraalii IASDSSGVIGHIVIAENAKVNATTTRTTTINVQDNANANFSGTKTYILIQGGARFNGTLGGPNFAVTGSNRFVNYDLIRAANQDYVITRT---NNAANVV

Rickettsia sp. California 2 VA---NGNIGHIVIAENAQVNATTTGTTTINVQDNANANFSDTQTYTLIQGGARFNGTLGGPNFAVTGSNRFVNYGLIRAANQDYVITRT---NNAANVV

R. hulinensis LA---NGNIGHIVIAEGAQVNATTTGTTTINVQDNANANFSGTQTYTLIQGGARFNGTLGGPNFTVTGSNRFVNYGLIRAANQDYVITRT---NNAENVV

**[1401-1500 ]**

**[ | | | | | | | | | | ]**

R. bellii RML369-C NNDISGN---GNPVIRHNLVTFLNPNNTGNAEQAYNNIL-LAQNTNDAATSFAATLTDTSTSVNNININVAKEEQGLIGYRLDELRYLCNPESDKMASSE

R. bellii OSU 85-389 NNDISGN---GNPVIRHNLVTFLNPNNTGNAEQAYNNIL-LAQNTNDAATSFAATLTDTSTSVNNININVAKEEQGLIGYRLDELRYLCTPESDKMASSE

R. prowazekii Madrid E TTAVGNSAIANAPGVSQNISRCLESTNTA----AYNNML-LAKDPSDVATFVGAIATDTSAAVTTVNLNDTQKTQDLLSNRLGTLRYLSNAETSDVAGSA

R. prowazekii Rp22 TTAVGNSAIANAPGVSQNISRCLESTNTA----AYNNML-LAKDPSDVATFVGAIATDTSAAVTTVNLNDTQKTQDLLSNRLGTLRYLSNAETSDVAGSA

**R. typhi Wilmington TTAVGNSAIANAPGVHQNIAICLESTDTA----AYNNML-LAKDSSDVATFIGAIATDTGAAVATVNLNDTQKTQDLLGNRLGALRYLSNSETADVGGSE**

R. akari Hartford TNDIANSPFASASGVGQNVTTFVNATNTA----AYNNLL-LAKNSADSADFVGAITTDTSAAVTNAQLDVAKDIQAQLGNRLGTLRYLGTHETAEMAGPE

R. australis TNDIANSPFAGAPGVGQNVTTFVNATNTV----AYNNLL-LAKNSADSANFVGAITTDSSAAVTNAQLDVAKDIQAQLGNRLGALRYLGTPETAEMAGPE

R. felis URRWXCal2 TNDIANSPFASAPGVGQNVTTFVNSTNTA----AYNNLL-LAKNSADSANFVGAITTDTSAAITNAQLDVAKDIQAQLGNRLGALRYLGTPETAEMAGPE

R. canadensis McKiel TKAIGNSQFA---GIGQNVLTFVN-SNTA----AYNNLL-LAKNTTDAANFVGAITTDTSAVVTNAQLDVAKDIQAQLSNRLGALRYLGTYDSSKMAGAE

R. tamurae TNDIAGSQFGGAPGIGQNITTFVNATNTA----AYNNLL-LAKNSADSANFVGAIVTDTSAAVTNVQLNVVKDIQAQLSNRLGALRYLGTPETAEMAGPE

R. helvetica TNDIANGPFGGAPGVGQNVTTFVNATNTA----AYNNLL-LAKNSADSANFVGTITTDTSAAVTNAQLDIAKDIQAQLGNRLSALRYLGTPETAEMTGPE

REIS INDIAGSQFAGAPGVGQNVTTFVNATNTA----AYNNLL-LAKNSADSANFVGAIVTGTSAAVTNVQLDVAKDIQAQLGNRLGALRYLG---TAEMAGPE

R. monacensis TNDIAGSQFAGAPGVGQNVTTFVNATNTA----AYNNLL-LAKNSADSANFVGAIVTGTSAAVTNVQLDVSKDIQAQLGNRLGALRYLGTPETAEMAGPE

R. massiliae MTU5 TNDIANSSFGGAPGVGQNVTTFVNATNTA----AYNNLL-LAKNSADSANFVGAIVTDTSAAVTNAQLDVAKDIQAQLGNRLGTLRYLGTPETAEMAGPE

Candidatus R. amblyommii TNDIANSPFGGAPGVGQNVTTFVNATNTV----AYNNLL-LAKNSADSANFVGAIVTDTSAAVTNAQFDVAKDIQAQLGNRLGALRYLGTPETAEMAGPE

R. rhipicephali TNDIANSSFGGAPGVGQNVTTFVNATNTA----AYNNLL-LAKNSADSANFVGAIVTDTSAAVTNAQLDVAKDIQAQLGNRLGALRYLGTPETAEMAGPE

R. aeschlimannii SNDIANSSFGGAPGVGQNVTTFVNATNTA----AYNNLL-LAKNSADSANFVGAIVTDTSAVVTNAQLDVAKDIQAQLGNRLGALRYLGTPETAEMAGPE

R. montanensis TNDIASSTFGGALGVGQNVTTFVNATNTA----AYNNLL-LAKNSADSANFVGSIVTDTSAAVTNVQLDVAKNIQAQLGNRLGALRYLGTPETAEMAGPE

R. slovaca 13-B TNDIANSPFGGAPGVGQNVTTFVNATNTA----AYNNLL-LAKNSANSANFVGAIVTDTSAAITNAQLEVAKDIQAQLGNRLGALRYLGTPETAEMAGPE

R. peacockii Rustic TNDIANSPFGGAPGVGQNVTTFVNATNTA----AYNNLL-LAKNSANSANFVGAIVTDTSAAITNAQLDVAKDIQAQLGNRLGALRYLGTPETAEMAGPE

R. rickettsii Iowa TNDIANSPFGGAPGVDQNVTTFVNATNTA----AYNNLL-LAKNSANSANFVGAIVTDTSAAITNVQLDLAKDIQAQLGNRLGALRYLGTPETAEMAGPE

R. rickettsii Sheila Smith TNDIANSPFGGAPGVDQNVTTFVNATNTA----AYNNLL-LAKNSANSANFVGAIVTDTSAAITNVQLDLAKDIQAQLGNRLGALRYLGTPETAEMAGPE

R. japonica TNDITNSPFGGAPGVGQNVTTFVNATNTA----AYNNLL-LAKNSADSANFVGTIVTDTSAAITNAQLDVAKDIQAQLGNRLGALRYLGTP---EMVGSE

R. honei TNDIANSPFGVAPGVGQNVTTFVNATNTA----AYNNLL-LAKNSANAANFVGAIVTDTSAAITNAQLDVAKDIQAQLGNRLGALRYLGTPETAEMAGPE

R. conorii str. Malish 7 TNDIANSSFGGAPGVGQNVTTFVNATNTA----AYNNLL-LAKNSANSANFVGAIVTDTSAAITNAQLDVAKDIQAQLGNRLGALRYLGTPETAEMAGPE

R. sibirica 246 TNDIANSPFGGAPGVGQNVTTFVNATNTA----AYNNLL-LAKNSANAANFVGAIVTDTSAAITNAQLDVAKGIQDQLGNRLGALRYLGTPETAEMAGPE

R. africae ESF-5 TNDIANSPFGGAPGVGQNVTTFVNATNTA----AYNNLL-LAKNSANAANFVGAIVTDTSAAITNAQLDVAKDIQAHLGNRLGALRYLGTPETAEMAGPE

R. parkeri TNDIANSPFGGAPGVGQNVTTFVNATNTA----AYNNLL-LAKNSANAANFVGAIVTDTSAAITNAQLDVAKDIQAQLGNRLGALRYLGTPETAEMAGPE

R. heilongjiangensis TNDIANSPFGGAPGIGQNVTTFVNATNTA----AYNNLL-LAKNSADSANFVGTIVTDTSAAITNAQLDVAKDIQAQLGNRLGALRYLGTP---EMVGSE

Israeli tick typhus TNDIANSPFGGAPGVGQNVTTFVNATNTA----AYNNLL-LAKNSANSAHFVGAIVTDTNAAITNAQLDVAKDIQAQLGNRLGALRYLG---TAEMAGPE

Rickettsia sp. A-167 TNDIANSPFGGAPGVGQNVTTFVNATNTA----AYNNLL-LAKNSANAANFVGAIVTDTSAAITNAQLDVAKDIQAQLGNRLGALRYLG---TAEMAGPE

Candidatus R. tasmanensis TNDIANSPFGGAPGVGQNVTTFVNATNTA----AYNNLL-LAKNSADSANFVGAIVTDTSAAITNAQLDIAKDIQAQLGNRLGALRYLG---TAEMAGPE

Rickettsia sp. IG-1 TNDIANSPFGGAPGVGQNVTTFVNATNTA----AYNNLL-LAKNSANSANFVGAIVTDTSAAITNAQLDVVKDIQAQLGNRLGALRYLGTPETAEMAGPE

Candidatus R. barbariae ----------------------------------------------------------------------------------------------------

Rickettsia sp. S TNDIANSPFGGAPGVGQNVTTFVNATNTA----AYNNLL-LAKNSANAANFVGAIVTDTSAAITNAQLDVAKDIQAQLGNRLGALRYLGTPETAEMAGPE

Candidatus R. goldwasserii TNDIANSLFGGAPGVGQNVTTFVNATNTA----AYNNLL-LAKNSANSADFVGAIVTDTSAAITNAQLDVAKDIQAQLGNRLGALRYLGTPETTEMAGPE

Rickettsia mongolotimonae TNDIANSPFGGAPGVGQNVTTFVNATNTA----AYNNLL-LAKNSANAANFVGAIVTDTSAAITNAQLDVAKDIQAQLGNRLGALRYLGTPETAEMAGPE

Rickettsia sp. BJ-90 TNDIANSPFGGAPGVGQNVTTFVNATNTA----AYNNLL-LAKNSAYAANFVGAIVTDTCAAITNAQLDVAKGIQDQLGNRLGALRYLGTPETAEMAGPE

Candidatus R. andeanae TNDITNSPFGGAPGVGQNVTTFVNATNTA----AYNNLL-LAKNSADSANFVGAIVTDTSAAVTNAQLDVAKDIQAQLGNRLGALRYLGTPETAEMAGPE

Rickettsia sp. Bar29 TNDVANSSFGGTPGVGQNVTTFVNATNTA----AYNNLL-LAKNSADSANFVGAIVTDTSAAVTNAQLNVAKDIQAQLGNRLGTLRYLGTPETAEMAGPE

Rickettsia sp. TwKM01 TNDIANSSFGGAPGVGQNVTTFVNATNTA----AYNNLL-LAKNSADSANFVGAIVTDTSAAVTNAQLDVAKDIQSQLGNRLGTLRYLGTPETAEMAGPE

R. raoultii TNDIANSLFGGAPGVGQNVTTFVNATNTA----AYNNLL-LAQNSAAAANFVGAIVTDTSAAVTNAQLDVAKDIQAQLGNRLGALRYLGTPETAEMAGPE

R. asiatica TNDIANSPFGGAPGVGQNVTTFVNATNTT----AYNNLL-LAKNSADSANFVGAITTDTSAAVTNAQLDVAKDIQAQLGNRLSALRYLGTPETAEMAGPE

Rickettsia sp. 110 TTAIGNSPFAGAAGIGQNVATFLGATNTA----AYNNML-LAKNTKDAATFVRAITTNTSAAVTNAQLDVAKDIQAHLGNRVNAVRYLG---TAEMAGPE

Rickettsia sp. mmx79 TTAIGNSPFAGAAGIGQNVATFLGATNTA----AYNNML-LAKNTKDAVTFVRAITTNTSAAVTNAQLDVAKDIQAHLGNRVNAVRYLG---TAEMAGPE

Rickettsia sp. 103 TTAIGNSPFAGAAGIGQNVATFLGATNTA----AYNNML-LAKNTKDAATFVRAITTNTSAAVTNAQLDVAKDIQAHLGNRVNAVRYLG---TAEMAGPE

Rickettsia sp. J52 TTAIGNSPFAGAAGIGQNVATFLGATNTA----AYNNML-LAKNTKDAATFVRAITTNTSAAVTNAQLDVAKDIQAHLGNRVNAVRYLG---TAEMDGPE

Candidatus R. hoogstraalii TNDITNSPFAGASGVGQNVATFLAETNTA----AYSNLVNNAKNSANSANFVGAITTDTSAAVTNAQLDVAKDIQAQLGNRLGALRYLGTPETAEMAGPE

Rickettsia sp. California 2 TNDIANSPFASAPGVGQNVTTFVNSTNTA----AYNNLL-LAKNSADSANFVGAITTDTSAAITNAQLDVAKDIQAQLGNRLGALRYLGTPETAEMAGPE

R. hulinensis TNDIANSPFGGAPGIGQNVTTFVNATNTA----AYNNLL-LAKNSADSANFVGTIVTDTSAAITNAQLDVAKDIQAQLGNRLGALRYLGTP---EMVGSE

**[1501-1600 AT Domain ]**

**[ | | | | | | | | | | ]**

R. bellii RML369-C GG--------VAAGDEPIDNVAYGVWFKPLY**VDTHQSEKGGVAGYKAKTVGTVIGLDTLANDNLMIGAAIGLAKTDVKHQNYKRGDKTTIDSLSFSLYGA**

R. bellii OSU 85-389 GG--------VAAGDEPIDNVAYGVWFKPLY**VDTHQSEKGGVAGYKAKTVGTVIGLDTLANDNLMIGAAIGLAKTDVKHQNYKRGDKTTIDSLSFSLYGA**

R. prowazekii Madrid E TG-------AVSSGDEA--EVSYGVWAKPFY**NIAEQDKKGGIAGYKAKTTGVVVGLDTLASDNLMIGAAIGITKTDIKHQDYKKGDKTDINGLSFSLYGS**

R. prowazekii Rp22 TG-------AVSSGDEA--EVSYGVWAKPFY**NIAEQDKKGGIAGYKAKTTGVVVGLDTLASDNLMIGAAIGITKTDIKHQDYKKGDKTDINGLSFSLYGS**

**R. typhi Wilmington TG-------AVSSGDEAIDQVSYGVWAKPFYNIAEQDKKGGLAGYKAKTAGVVVGLDTLANDNLMIGAAIGITKTDIKHQDYKKGDKTDIKGLSFSLYGA**

R. akari Hartford AGAIP---AAVAAGDEAVDNVAYGVWAKPFY**TDAHQSKKGGLAGYKAKTTGVVIGLDTLAHDNLMIGAAIGITKTDIKHQDYKKGDKTDINGFSFSLYGA**

R. australis GGAIP---AAVAAGDAAVDNVAYGIWAKPFY**TDAHQSKKGGLAGYKAKTTGVVIGLDTLASDNLMIGAAIGITKTDIKHQDYKKGDKTDIKGFSFSLYGA**

R. felis URRWXCal2 AGAIP---AAVAAGDEAVDNVAYGIWAKPFY**TDAHQSKKGGLAGYKAKTTGVVIGLDTLANDNLMIGAAIGITKTDIKHQDYKKGDKTDVNGFTFSLYGA**

R. canadensis McKiel CGLVSTPQSPVTAGEEAIDNVAYGIWAKPFY**TDAHQSKKGGLAGYKAKTTGVVIGLDTLANDNLMIGAALGITKTDIKHQDYKKGDKTDINGFSFSLYSA**

R. tamurae AGAVP---AAVAAGDGAVDNVAYGIWAKPFY**TDAHQSKKGGLAGYKAKTTAVLIGLDTLANDNLMIGAAIGITKTDIKHQDYKKGDKTNVNGFSFSLYGA**

R. helvetica AGAVP---AAVAAGDEAVDNVAYGIWAKPFY**TDAHQSKKGGLAGYKAKTTGVVIGLDMLANDNLMIGAAVGITKTDIKHQDYKKGDKTDVNGFSFSLYGA**

REIS AE--P---AAVAAGDKAVDNVAYGIWTKSFY**TDVHQSKKGGLAGYKAKTTGVVIGLDTLANDNLMIGAAIGITKTDIKHQDYKKGDKTNVNGFSFSLYGA**

R. monacensis AGAVP---AAVAAGDEAVDNVAYGIWAKSFY**TDAHQSKKGGLAGYKAKTTGVVIGLDTLANDNLMIGAAIGITKTDIKHQDYKKGDKTNVNGFSFSLYGA**

R. massiliae MTU5 AGAIP---AAVAAGDEAVDNVAYGIWTKPFY**TDAHQSKKGGLAGYKAKTTGVVIGLDTLANDNLMIGAAIGITKTDIKHQDYKKGDKTNVNGFSFSLYGA**

Candidatus R. amblyommii AGTIP---AAVAAGDEAVDNVAYGIWAKPFY**TDAHQGKKGSLAGYKAKTTGVVIGLDTLANDNLMIGAAIGITKTDIKHQDYKKGDKTDVNGFSFSLYGA**

R. rhipicephali AGAIP---AAVAAGDEAVDNVAYGIWTKPFY**TDAHQSKKGGLAGYKAKTTGVVIGLDTLANDNLMIGAAIGITKTDIKHQDYKKGDKTNVNGFSFSLYGA**

R. aeschlimannii AGAIP---AVVAAGDEAVDNVAYGIWTKPFY**TDVHQSKKGGLAGYKAKTTGVVIGLDTLANDNLMIGAAIGITKTDIKHQDYKKGDKTDVNGFSFSLYGA**

R. montanensis AGAIP---AAVAAGDEAVDNVAYGIWAKPFY**TDAHQSKKGGLAGYKAKTTGVVIGLDTLANDNLMIGAAIGITKTDIKHQDYKKGDKTDVNGFSFSLYGA**

R. slovaca 13-B AGAIP---AAVAAGDEAVDNVAYGIWAKPFY**TDAHQSKKGGLAGYKAKTTGVVIGLDTLANDNLMIGAAIGITKTDIKHQDYKKGDKTDVNGFSFSLYGA**

R. peacockii Rustic AGAIP---AAVAAGDEAVDNVAYGIWAKPFY**TDAHQSKKGGLAGYKAKTTGVVIGLDTLANDNLMIGAAIGITKTDIKHQDYKKGDKTDVNGFSFSLYGA**

R. rickettsii Iowa AGAIS---AAVAAGDEAIDNVAYGIWAKPFY**TDAHQSKKGGLAGYKAKTTGVVIGLDTLANDNLMIGAAIGITKTDIKHQDYKKGDKTDVNGFSFSLYGA**

R. rickettsii Sheila Smith AGAIS---AAVAAGDEAIDNVAYGIWAKPFY**TDAHQSKKGGLAGYKAKTTGVVIGLDTLANDNLMIGAAIGITKTDIKHQDYKKGDKTDVNGFSFSLYGA**

R. japonica AGAIP---AAVAAGDEAVDNVAYGIWAKPFY**TDAHQSKKGGLAGYKAKTTGIVIGLDTLANNNLMIGAAIGITKTDIKHQDYKKGDKTDVNGFSFSLYGA**

R. honei AGAIP---AAVAAGDEAVDNVAYGIWAKPFY**TDAHQSKKGGLAGYKAKTTGVVIGLDTLANDNLMIGAAIGITKTDIKHQDYKKGDKTDVNGFSFSLYGA**

R. conorii str. Malish 7 AGAIP---AAVAAGDEAVDNVAYGIWAKPFY**TDAHQSKKGGLAGYKAKTTGVVIGLDTLANDNLMIGAAIGITKTDIKHQDYKKGDKTDVNGFSFSLYGA**

R. sibirica 246 AGAIP---AAVAAGDEAVDNVAYGIWAKPFY**TDAHQSKQGGLAGYKAKTTGVVIGLDTLANDNLMIGAAIGITKTDIKHQDYKKGDKTDVNGFSFSLYGA**

R. africae ESF-5 AGAIP---AAVAAGDEAVDNVAYGIWAKPFY**TDTHQSKKGGLAGYKAKTTGVVIGLDTLANDNLMIGAAIGITKTDIKHQDYKKGDKTDVNGFSFSLYGA**

R. parkeri AGAIP---AAVAAGDEAVDNVAYGIWAKPFY**TDTHQSKKGGLAGYKAKTTGVVIGLDTLANDNLMIGAAIGITKTDIKHQDYKKGDKTDVNGFSFSLYGA**

R. heilongjiangensis AGAIP---AAVAAGDEAVDNVAYGIWAKPFY**TDAHQSKKGGLAGYKAKTTGIVIGLDTLANNNLMIGAAIGITKTDIKHQDYKKGDKTDVNGFSFSLYGA**

Israeli tick typhus AGAIP---AAVAAGDEAVDNVAYGIWAKPFY**TDAHQSKKGGLAGYKAKTTGVVIGLDTLANDNLMIGAAIGITKTDIKHQDYKKGDKTDVNGFSFSLYGA**

Rickettsia sp. A-167 AGAIP---AAVAAGDEAVDNVAYGIWAKPFY**TDAHQSKKGGLAGYKAKTTGVVIGLDTLANDNLMIGAAIGITKTDIKHQDYKKGDKTDVNGFSFSLYGA**

Candidatus R. tasmanensis AGAIP---AAVAAGDEAVDNVAYGIWAKPFY**TDAHQSKKGGLAGYKAKTTGVVIGLDTLASDNLMIGAAIGITKTDIKHQDYKKGDKTDVNGFSFSLYGA**

Rickettsia sp. IG-1 AGAIP---DAVAAGDEAVDNVAYGIWAKPFY**TDAHQSKKGGLAGYKAKTTGVVIGLDTLANDNLMIGAAIGITKTDIKHQDYKKGDKTDVNGFSFSLYGA**

Candidatus R. barbariae -------------------------------**---------------------------------------------------------------------**

Rickettsia sp. S AGAIP---AAVAAGDEAVDNVAYGIWAKPFY**TDTHQSKKGGLAGYKAKTTGVVIGLDTLANDNLMIGAAIGITKTDIKHQDYKKGDKTDVNGFSFSLYGA**

Candidatus R. goldwasserii AGAIP---AAVAAGDEAVDNVAYGIWAKPFY**TDAHQSKKGGLAGYKAKTTGVVIGLDTLANDNLMIGASIFIIKTDIKHQDYKKGDKTDVNGFSISLYGA**

Rickettsia mongolotimonae AGAIP---AAVAAGDEAVDNVAYGIWAKPFY**TDAHQSKKGGLAGYKAKTTGVVIGLDTLANDNLMIGAAIGITKTDIKHQDYKKGDKTDVNGFSFSLYGA**

Rickettsia sp. BJ-90 AGAIP---AAIAAGDEAVDNVAYGLWAKPFY**TDAHQSKKGGLAGYKAKTTGVVIGLDTLANENLIIGAAIGITKTDIEHQDYKKGAKTDVNGFSFSLYGA**

Candidatus R. andeanae AGTIP---AAVAAGDEAVDNVAYGIWAKPFY**TDAHQSKKGSLAGYKAKTTGVVIGLDTLANDNLMIGAAIGITKTDIKHQDYKKGDKTDVNGFSFSLYGA**

Rickettsia sp. Bar29 AGAIP---AAVAAGDEAVDNVAYGIWTKPFY**TDAHQSKKGGLAGYKAKTTGVVIGLDTLANDNLMIGAAIGITKTDIKHQDYKKGDKTDVNGFSFSLYGA**

Rickettsia sp. TwKM01 AGAIP---AAVAAGDEAVDNVAYGIWTKPFY**TDAHQSKKGGLAGYKAKTTGVVIGLDTLANDNLMIGAAIGITKTDIKHQDYKKGDKTNVNGFSFSLYGA**

R. raoultii VGAIP---AAVAAGDEAVDNVAYGIWAKPFY**TDAHQSKKGGLAGYKAKTTGVVIGLDTLANDNLMIGAAIGITKTDIKHQDYKKGDKTDVNGFSFSLYGA**

R. asiatica VGAVP---AAVAAGDEAVDNVAYGIWAKPFY**TDAHQSKKGGLAGYKAKTTGVVIGLDMLANDNLMIGAAIGITKTDIKHQDYKKGDKTDVNGFSFSLYGA**

Rickettsia sp. 110 AGAV----APVAAGDEAVDNVAYGIWAKPFY**TDAHQSKKGGLAGYKAKTTGVVIGLDTLANDNLMIGAAIGITKTDIKHQDYKKGDKTDVNGFSFSLYGA**

Rickettsia sp. mmx79 AGAV----APVAAGDEAVDNVAYGIWAKPFY**TDAHQSKKGGLAGYKAKTTGVVIGLDTLANDNLMIGAAIGITKTDIKHQDYKKGDKTDVNGFSFSLYGA**

Rickettsia sp. 103 AGAV----APVAAGDEAVDNVAYGIWAKPFY**TDAHQSKKGGLAGYKAKTTGVVIGLDTLANDNLMIGAAIGITKTDIKHQDYKKGDKTDVNGFSFSLYGA**

Rickettsia sp. J52 AGAV----APVAAGDEAVDNVAYGIWAKPFY**TDAHQSKKGGLAGYKAKTTGVVIGLDTLANDNLMIGAAIGITKTDIKHQDYKKGDKTDVNGFSFSLYGA**

Candidatus R. hoogstraalii AGAIP---AAVAAGDEAVDNVAYGIWAKPFY**TDAHQSKKGGLAGYKAKTTGVVIGLDTLANDNLMIGAAIGITKTDIKHQDYKKGDKTDVNGFSFSLYGA**

Rickettsia sp. California 2 AGAIP---AAVAAGDEAVDNVAYGIWAKPFY**TDAHQSKKGGLAGYKAKTTGVVIGLDTLANDNLMIGAAIGITKTDIKHQDYKKGDKTDVNGFTFSLYGA**

R. hulinensis AGAIP---AAVAAGDEAVDNVAYGIWAKPFY**TDAHQSKKGGLAGYKAKTTGIVIGLDTLANNNLMIGAAIGITKTDIKHQDYKKGDKTDVNGFSFSLYGA**

**[1601-1700 ]**

**[ | | | | | | | | | | ]**

R. bellii RML369-C **QQLVANFFVHGNAILSVNQVKNRSQRYVFDKNGNRSTQIASANYSNMTFGGNFMVGYDASLMDGLLVTPMAGLSYLKSSDESYKESGTTFANRQVYSKFS**

R. bellii OSU 85-389 **QQLVANFFVHGNAILSVNQVKNRSQRYVFDKNGNRSTQIASANYSNMTFGGNFMVGYDASLMDGLLVTPMAGLSYLKSSDESYKESGTTFANRQVYSKFS**

R. prowazekii Madrid E **QQLVKNFFAQGNAIFTLNKVKSKSQRYFFESNGKMSKQIAAGNYDNMTFGGNLIFGYDYNAMPNVLVTPMAGLSYLKSSNENYKETGTTVANKRINSKFS**

R. prowazekii Rp22 **QQLVKNFFAQGNAIFTLNKVKSKSQRYFFESNGKMSKQIAAGNYDNMTFGGNLIFGYDYNAMPNVLVTPMAGLSYLKSSNENYKETGTTVANKRINSKFS**

**R. typhi Wilmington QQLVKNFFAQGSAIFTLNKVKSKSQRYFFDANGKMNKQIAAGNYDNITFGGNLMFGYDYNALQGVLVTPMAGLSYLKSSNENYKETGTTVANKRIHSKFS**

R. akari Hartford **QQLVKNFFAQGSAIFSLNHVKNKSQRYFFDANGKMNKQVAAGNYGNITFGGNLMVGYDYNALQGVLVTPMAGLSYLKSSDENYKESGTTVANKQVNSKFS**

R. australis **QQLVNNFFAQGSAIFSLNQVKNKSQRYFFDPNGKMNKQIAAGNYDNMTFGGNLMVGYDYNAVQGVLVTPMAGLSYLKSSDENYKETGTTVANKQVNSKFS**

R. felis URRWXCal2 **QQLVENFFAQGSAIFSLNQVKNKSQRYFFDANGKMNKQIAAGNYDNMTFGGNLMVGYDYNAMQGVLVTPMAGLSYLKSSDENYKETGTTVANKQVNSKFS**

R. canadensis McKiel **QQLVKNFFAQGSAIFSLNHVKNKSQRYFFDANGNMSKQIAAGHYDNMTFGGNLIVGYDYNAMEGVLVTPMAGLSYLKSSDENYKETGTTVANKQVNSKFS**

R. tamurae **QQLVENFFAQGSAIFNLNQVKNKSQRYFFDANGNMSKQIAAGNYDNMTFGGNLTVGYDYNAMQGVLVNPMAGLSYLKSSDENYKETGTTVANKQVNSKFS**

R. helvetica **QQLVKNFFAQGSAIFSLNQVKNKSQRYFFDANGNMSKQIAAGNYDNMTFGGNLMVGYDYNAMQGVLVTPMAGLSYLKSSDENYKETGTTVANKQVNSKFS**

REIS **QQLVENFFAQGSAIFSLNQVKNKSQRYFFDANGNMSKQIAAGNYDNMTFGGNLTVGYDYNAMQGVLVTPMAGLSYLKSSDENYKETSTTVANKQVNSKFS**

R. monacensis **QQLVENFFAQGSAIFSLNQVKNKSQRYFFDANGNMSKQIAAGNYDNMTFGGNLTVGYDYNAMQGVLVTPMAGLSYLKSSDENYKETGTTVANKQVNSKFS**

R. massiliae MTU5 **QQLVENFFAQGSAIFSLNQVKNKSQRYCFDANGNMSKQIAAGNYDNMTFGGNLTVGYDYNAMQGVLVTPMAGLSYLKSSDENYKETGTTVANKQVHSKFS**

Candidatus R. amblyommii **QQLVENFFAQGSAIFNLNQVKNKSQRYFFDANGNMSKQIAAGNYNNMTFGGNLTVGYDYNAMQGVLITPMAGLSYLKSSDENYKETGTTVANKQVNSKFS**

R. rhipicephali **QQLVENFFAQGSAIFSLNQVKNKSQRYFFDANGNMSKQIAAGNYDNMTFGGNLTVGYDYNAMQGVLVTPMAGLSYLKSSDENYKETGTTVANKQVNSKFS**

R. aeschlimannii **QQLVENFFAQGSAIFSLNQVKNKSQRYFFDANGNMSKQIAAGNYDNMTFGGNLTVGYDYNAMQGVLVTPMAGLSYLKSSDENYKETGTTVANKQVNSKFS**

R. montanensis **QQLVENFFAQGSAIFSLNQVKNKSQRYFFDANGNMSKQIAAGNYDNMTFGGNLTVGYDYNAMQGVLVTPMAGLSYLKSSDENYKETGTTVANKQVNSKFS**

R. slovaca 13-B **QQLVKNFFAQGSAIFSLNQVKNKSQRYFFDANGNMSKQIAAGHYDNMTFGGNLTVGYDYNAMQGVLVTPMAGLSYLKSSDENYKETGTTVANKQVNSKFS**

R. peacockii Rustic **QQLVKNFFAQGSAIFSLNQVKNKSQRYFFYANGNMSKQIAAGHYDNMTFGGNLTVGYDYNAMQGVLVTPMAGLRYLKSSDENYKETGTTVANKQVNSKFS**

R. rickettsii Iowa **QQLVKNFFAQGSAIFSLNQVKNKSQRYFFDANGNMSKQIAAGHYDNMTFGGNLTVGYDYNAMQGVLVTPMAGLSYLKSSDENYKETGTTVANKQVNSKFS**

R. rickettsii Sheila Smith **QQLVKNFFAQGSAIFSLNQVKNKSQRYFFDANGNMSKQIAAGHYDNMTFGGNLTVGYDYNAMQGVLVTPMAGLSYLKSSDENYKETGTTVANKQVNSKFS**

R. japonica **QQFVENFFAQGSAIFSLNQVKNKSQRYFFDANGNMSKQIAAGNYDNMTFGGNLTVGYDYNAMQGVLVTPMAGLSYLKSSDENYKETGTTVANKQVNSKFS**

R. honei **QQLVKNFFAQGSAIFSLNQVKNKSQRYFFDANGNMSKQIAAGHYDNMTFGGNLTVGYDYNAMQGVLVTPMAGLSYLKSSDENYKETGTTVANKQVNSKFS**

R. conorii str. Malish 7 **QQLVKNFFAQGSAIFSLNQVKNKSQRYFFDANGNMSKQIAAGHYDNMTFGGNLTVGYDYNAMQGVLVTPMAGLSYLKSSDENYKETGTTVANKQVNSKFS**

R. sibirica 246 **QQLVKNFFAQGSAIFSLNQVKNKSQRYFFDANGNMSKQIAAGHYDNMTFGGNLTVGYDYNAMQGVLVTPMAGLSYLKSSDENYKETGTTVANKQVNSKFS**

R. africae ESF-5 **QQLVKNFFAQGSAIFSLNQVKNKSQRYFFDANGNMSKQIAAGHYDNMTFGGNLTVGYDYNAMQGVLVTPMAGLSYLKSSDENYKETGTTVANKQVNSKFS**

R. parkeri **QQLVKNFFAQGSAIFSLNQVKNKSQRYFFDANGNMSKQIAAGHYDNMTFGGNLTVGYDYNAMQGVLVTPMAGLSYLKSSDENYKETGTTVANKQVNSKFS**

R. heilongjiangensis **QQLVENFFAQGSAIFSLNQVKNKSQRYFFDANGNMSKQIAAGNYDNMTFGGNLTVGYDYNAMQGVLVTPMAGLSYLKSSDENYKETGTTVANKQVNSKFS**

Israeli tick typhus **QQLVKNFFAQGSAIFSLNQVKNKSQRYFFDANGNMSKQIAAGHYDNMTFGGNLTVGYDYNAMQGVLVTPMAGLSYLKSSDENYKETGTTVANKQVNSKFS**

Rickettsia sp. A-167 **QQLVKNFFAQGSAIFSLNQVKNKSQRYFFDANGNMSKQIAAGHYDNMTFGGNLTVGYDYNAMQGVLVTPMAGLSYLKSSDENYKETGTTVANKQVNSKFS**

Candidatus R. tasmanensis **QQLVENFFAQGSAIFSLNQVKNKSQRYFFDANGNMSKQIAAGNYDNMTFGGNLTVGYDYNAMQGVLVTPMAGLSYLKSSDENYKETGTTVANKQVNSKFS**

Rickettsia sp. IG-1 **QQLVKNFFAQGSAIFSLNQVKNKSQRYFFDTNGNMSKQIAAGHYDNMTFGGNLTVGYDYNAMQGVLVTPMAGLSYLKSSDENYKETGTTVANKQVNSKFS**

Candidatus R. barbariae **----------------------------------------------------------------------------------------------------**

Rickettsia sp. S **QQLVKNFFAQGSAIFSLNQVKNKSQRYFFDANGNMSKQIAAGHYDNMTFGGNLTVGYDYNAMQGVLVTPMAGLSYLKSSDENYKETGTTVANKQVNSKFS**

Candidatus R. goldwasserii **QQLVKNFFAQGSAIFSLNQVKNKSQRYFFDANGNMSKQIAAGHYDNMTFGGNLTVGYDYNAMQGVLVTPMAGLSYLKSSDENYKETGTTVANKQVNSKFS**

Rickettsia mongolotimonae **QQLVKNFFAQGSAIFSLNQVKNKSQRYFFDANGNMSKQIASGHYDNMTFGGNLTVGYDYNAMQGVLVTPMAGLSYLKSSDENYKETGTTVANKQVNSKFS**

Rickettsia sp. BJ-90 **QQLVKNFFAQGSAIFSLNQVKNKSQRYFFDANGNMSKQIAAGHYDNMTFGGNLTVGYDYNAMQGVLVTPMAGLSYLKSSDENYKETGTTVANKQVNSKFS**

Candidatus R. andeanae **QQLVENFFAQGSAIFSLNQVKNKSQRYFFEANGNMSKQIAAGNYNNMTFGGNLTVGYDYNAMQGVLVTPMAGLSYLKSSDENYKETGTTVANKQVNSKFS**

Rickettsia sp. Bar29 **QQLVENFFAQGSAIFSLNQVKNKSQRYFFDANGNMSKQIAAGNYDNMTFGGNLTVGYDYNAMQGVLVTPMAGLSYLKSSDENYKETGTTVANKQVHSKFS**

Rickettsia sp. TwKM01 **QQLVENFFAQGSAIFSLNQVKNKSQRYFFDANGNMSKQIAAGNYDNMTFGGNLTVGYDYNAMQGVLVTPMVGLSYLKSSDENYKETGTTVANKQVNNKFS**

R. raoultii **QQLVENFFAQGSAIFSLNQVKNKSQRYFFDANGNMSKQIAAGKYDNMTFGGNLTVGYDYNAMQGVLVTPMVGLSYLKSSDENYKETGTTVANKQVNSKFS**

R. asiatica **QQLVKNFFAQGSAIFSLNQVKNKSQRYFFDANGNMSKQIAAGNYDNMTFGGNLMVGYDYNAMQGVLVTPMAGLSYLKSSDENYKETGTTVANKQVNSKFS**

Rickettsia sp. 110 **QQLVENFFAQGSAIFSLNQVKNKSQCYFFDANGNMSKQIAAGNYNNMTFGGNLTVGYDYNAMEGLLVTPMAGLSYLKSSDENYKETGTTVANKQVNSKFS**

Rickettsia sp. mmx79 **QQLVENFFAQGSAIFSLNQVKNKSQCYFFDANGNMSKQIAAGNYNNMTFGGNLTVGYDYNAMEGLLVTPMAGLSYLKSSDENYKETGTTVANKQVNSKFS**

Rickettsia sp. 103 **QQLVENFFAQGSAIFSLNQVKNKSQRYFFDANGNMSKQIAAGNYNNMTFGGNLTVGYDYNAMEGLLVTPMAGLSYLKSSDENYKETGTTVANKQVNSKFS**

Rickettsia sp. J52 **QQLVENFFAQGSAIFSLNQVKNKSQCYFFDANGNMSKQIAAGNYNNMTFGGNLTVGYDYNAMEGLLVTPMAGLSYLKSSDENYKETGTTVANKQVNSKFS**

Candidatus R. hoogstraalii **QQLVENFFAQGSAIFSLNQVKNKSQRYFFDANGKMNKQIAAGNYDNMTFGGNLMVGYDYNAMQSVLVTPMAGLSYLKSSDENYKETGTTVANKQVNSKFS**

Rickettsia sp. California 2 **QQLVENFFAQGSAIFSLNQVKNKSQRYFFDANGKMNKQIAAGNYDNMTFGGNLMVGYDYNAMQGVLVTPMAGLSYLKSSDENYKETGTTVANKQVNSKFS**

R. hulinensis **QQLVENFFAQGSAIFSLNQVKNKSQRYFFDANGNMSKQIAAGNYDNMTFGGNLTVGYDYNAMQGVLVTPMAGLSYLKSSDENYKETGTTVANKQVNSKFS**

**[1701-1800 ]**

**[ | | | | | | | | | | ]**

R. bellii RML369-C **DRTDFIGGAKLMGYTINLADLIVYPEAHAFVIQKIGGRLSKTQYQLEGQVNPYITPSDKTARTTYNLGLSGTIRPDTKMEYGIGYDLNIAKKFI**SHQGTV

R. bellii OSU 85-389 **DRTDFIGGAKLMGYTINLADLIVYPEAHAFVIQKIGGRLSKTQYQLEGQVNPYITPSDKTARTTYNLGLSGTIRPDTKMEYGIGYDLNIAKKFI**SHQGTV

R. prowazekii Madrid E **DRVDLIVGAKVAGSTVNITDIVIYPEIHSFVVHKVNGKLSNSQSMLDGQTAPFISQPDRTAKTSYNIGLSANIKSDAKMEYGIGYDFNSASKYT**AHQGTL

R. prowazekii Rp22 **DRVDLIVGAKVAGSTVNITDIVIYPEIHSFVVHKVNGKLSNSQSMLDGQTAPFISQPDRTAKTSYNIGLSANIKSDAKMEYGIGYDFNSASKYT**AHQGTL

**R. typhi Wilmington DRIDLIVGAKVTGSAMNINDIVIYPEIHSFVVHKVNGKLSKAQSMLDGQTAPFISQPDRTAKTSYNIGLSANIRSDAKMEYGIGYDFNAASKYTAHQGTL**

R. akari Hartford **DRTDLIVGAKVAGGTMNITDLAVYPEAHVFVVHKVNGRLSKTQSQLDGQVTPFISQPDRTAKTSYNLGLSASIRPDAKMEYGVGYDAQIASKYV**AHQGTL

R. australis **DRTDLIVGAKVAGGTMNITDLAVYPEAHAFVVHKVNGRLSKTQSQLDGQVTPFISQPDKTAKTSYNLGLSASIRPDAKMEYGVGYDAQIASKYV**AHQGT-

R. felis URRWXCal2 **DRTDLIVGVKVAGGTMNITDLAVYPEAHAFVVHKVNGRLSKTQSQLDGQVTPFISQPDRTAKTSYNIGLSASIRPDAKMEYGIGYDFNAASKYT**AHQGTL

R. canadensis McKiel **DRTDLILGVRVMGNTMNFSDLAVYPEAHAFVVHKVNGRLSKTQSQLDGQVTPFISQSDSSAKTSYNLGLSASIRPDSKMEYGIGYDAQIASKYT**AHQGTL

R. tamurae **DRTDLIVGAKVAGGTMNITDLAVYPEAHAFVVHKVNGKLSKTRSVLDGQVTPFISQPDRTAKTSYNLGLSASIRPDAKMEYGIGYDAQIASKYT**AYQGTL

R. helvetica **DRTDLIVGAKVAGGTMNITDLAVYPEAHAFVVHKVNGRLSKTQSVLDGQVTPFISQPDRTAKTSYNLGLSASIRPDAKMEYGIGYDAQIASKYT**AHQGTL

REIS **DRTDLIVGAKVAGGTMNITDLAVYPEAHAFVVHKVNGRLSKTQSVLDGQVTPFISQPDRTAKTSYNLGLSASIRPDAKMEYGIGYDAQIASKYT**AHQGAL

R. monacensis **DRTDLIVGAKVVGGTMNITDLAVYPEAHAFVVHKVNGRLSKTQSVLDGQVTPFISQPDRTAKTSYNLGLSASIRSDAKMEYGIGYDAQIASKYT**AHQGTL

R. massiliae MTU5 **DRTDLIVGAKVVGGTINITDLAVYPEAHAFVVHKVNGRLSKTQSVLDGQVTPCISQPDRTAKTSYNLGLSASIRPDAKMEYGIGYDAQIASKYT**AHQGTL

Candidatus R. amblyommii **DRTDLIVGAKVAGGTMNITDLAVYPEAHAFVVHKVNGRLSKTQSVLDGQVTPCISQPDRTAKTSYNLGLSASIRPDAKMEYGIGYNARIAS---**------

R. rhipicephali **DRTDLIVGAKVAGGTMNITDLAVYPEAHAFVVHKVKGRLSKTQSVLDGQVTPCISQPDRTAKTSYNLGLSASIRPDAKMEYGIGYDAQIASKYT**AHQGTL

R. aeschlimannii **DRTDLIVGAKVVGGTMNITDLAVYPEAHAFVVHKVNGRLSKTQSVLDGQVTPCISQPDRTAKTSYNLGLSASIRPDAKMEYGIGYDAQIASKYT**AHQGTL

R. montanensis **DRTDLIVGAKVAGGTMNITDLVVYPEAHAFVVHKVNGRLSKTQSVLDGQVTPCISQSDRTAKTSYNLGLSASIRPDAKMEYGIGYDAQIASKYT**AHQGTL

R. slovaca 13-B **DRTDLIVGAKVAGSTMNITDLAVYPEVHAFVVHKVTGRLSKTQSVLDGQVTPCISQPDRTAKTSYNLGLSASIRSDAKMEYGIGYDAQISSKYT**AHQGTL

R. peacockii Rustic **DRTDLIVGAKVAGSTMNITDLAVYPEVHAFVVHKVTGRLSKTQSVLDGQVTPCISQPDRTAKTSYNLGLSASIRSDAKMEYGIGYDAQISSKYT**AHQGTL

R. rickettsii Iowa **DRTDLIVGAKVAGSTMNITDLAVYPEVHAFVVHKVTGRLSKTQSVLDGQVTPCINQPDRTTKTSYNLGLSASIRSDAKMEYGIGYDAQISSKYT**AHQGTL

R. rickettsii Sheila Smith **DRTDLIVGAKVAGSTMNITDLAVYPEVHAFVVHKVTGRLSKTQSVLDGQVTPCINQPDRTTKTSYNLGLSASIRSDAKMEYGIGYDAQISSKYT**AHQGTL

R. japonica **DRTDLIVGAKVAGGTMNITDFAVYPEVHAFVVHKVTGRLSKTQSVLDGQVTPCISQPDRTAKTSYNLGLSASIRSDAKMEYGIGYDAQIASKYT**AHQGTL

R. honei **DRTDLIVGAKVAGSTMNITDLAVYPEVHAFVVHKVTGRLSKTQSVLDGQVTPCISQPDRTAKTSYNLGLSASIRSDAKMEYGIGYDAQIASKYT**AHQGTL

R. conorii str. Malish 7 **DRTDLIVGAKVAGSTMNITDLAVYPEVHAFVVHKVTGRLSKTQSVLDGQVTPCISQPDRTAKTSYNLGLSASIRSDAKMEYGIGYDAQISSKYT**AHQGTL

R. sibirica 246 **DRTDLIVGAKVAGSTMNITDLAVYPEVHAFVVHKVTGRLSKTQSVLDGQVTPCISQPDRTAKTSYNLGLSASIRSDAKMEYGIGYDAQISSKYT**AHQGTL

R. africae ESF-5 **DRTDLIVGAKVAGSTMNITDLAVYPEVHAFVVHKVTGRLSKTQSVLDGQVTPCISQPDRTAKTSYNLGLSASIRSDAKMEYGIGYDAQISSKYT**AHQGTL

R. parkeri **DRTDLIVGAKVAGSTMNITDLAVYPEVHAFVVHKVTGRLSKTQSVLDGQVTPCISQPDRTAKTSYNLGLSASIRSDAKMEYGIGYDAQISSKYT**AHQGTL

R. heilongjiangensis **DRTDLIVGAKVAGGTMNITDFAVYPEVHAFVVHKVTGRLSKTQSVLDGQVTPCISQPDRTAKTSYNLGLSASIRSDAKMEYGIGYDAQIASKYT**AHQGTL

Israeli tick typhus **DRTDLIVGAKVAGSTMNITDLAVYPEVHAFVVHKVTGRLSKTQSVLDGQVTPCISQPDRTAKTSYNLGLSASIRSDAKMEYGIGYDAQISSKYT**AHQGTL

Rickettsia sp. A-167 **DRTDLIVGAKVAGSTMNITDLAVYPEVHAFVVHKVTGRLSKTQSVLDGQVTPCISQPDRTAKTSYNLGLSASIRSDAKMEYGIGYDAQISSKYT**AHQGTL

Candidatus R. tasmanensis **NRTDLIVGAKVAGGTMNITDLAVYPEVHAFVVHKVTGRLSKTQSVLDGQVTPCISQPDRTAKTSYNLGLSASIRSDAKMEYGIGYDAQIASKYT**AHQGTL

Rickettsia sp. IG-1 **DRTDLIVGAKVAGSTMNITDLAVYPEVHAFVVHKVTGRLSKTQSVLDGQVTPCISQPDRTAKTSYNLGLSASIRSDAKMEYEIGYDAQISSKYT**AHQGTL

Candidatus R. barbariae **----------------------------------------------------------------------------------------------**------

Rickettsia sp. S **DRTDLIVGAKVAGSTMNITDLAVYPEVHAFVVHKVTGRLSKTQSVLDGQVTPCISQPDRTAKTSYNLGLSASIRSDAKMEYGIGYDAQISSKYT**AHQGTL

Candidatus R. goldwasserii **DRTDLIVGAKVAG-TMNITDLAVYPEVHAFVVHKVTGRLSKTQSVLDGQVTPCISQPDRTAKTSYNLGLSASIRSDAKMEYGIGYDAQISSKYT**AHQGTL

Rickettsia mongolotimonae **DRTDLIVGAKVAGSTMNITDLAVYPEVHAFVVHKVTGRLSKTQSVLDGQVTPCISQPDRTAKTSYNLGLSASIRSDAKMEYGIGYDAQISSKYT**AHQGTL

Rickettsia sp. BJ-90 **DRTDLIVGAKVAGSTMNITDLAVYPEVHAFVVHKVTGRLSKTQSVLDGQVTPCISQPDRTAKTSYNLGLSASIRSDAKMEYGIGYDAQISSKLT**AHQGTL

Candidatus R. andeanae **DRTDLIVGVKVAGGTVNITDLAVYPEAHAFVVHKVNGRLSKTQSVLDGQVTPCISQPDRTAKTSYNLGLSASIRPDAKMEYGIGYDAQIAS---**------

Rickettsia sp. Bar29 **DRTDLIVGAKVAGGTINITDLAVYPEAHAFVVHKVNGRLSKTQSVLDGQVTPCISQPDRTAKTSYNLGLSASIRPDAKMEYGIGYDAQIASKYT**AHQGTL

Rickettsia sp. TwKM01 **DRTDLIVGAKVAGGTMNITNLAVYPEAHAFVVHKVNGRLSKTQSVLDGQVTPCISQPDRTAKTSYNLGLSASIRPDAKMEYGIGYDAQIASKYT**AHQGT-

R. raoultii **DRTDLIVGAKVAGGTMNITNLAVYPEAHAFVVHKVNGRLSKTQSVLDGQVTPCISQPDRTAKTSYNLGLSASIRPDAKMEYGIGYDAQIASKYT**AHQGTL

R. asiatica **DRTDLIVGAKVAGGTMNITDLAVYPEAHAFVVHKVNGRLSKTQSVLDGQVTPFISQPDRTAKTSYNLGLSASIRPDAKMEYGIGYDAQIASKYT**AYQGTL

Rickettsia sp. 110 **DRTDLIVGARVMGGTMNFSDLAVYPEAHAFVVHKVNGRLSKTQSQLDGQVTPFISQPDRTAKTSYNLGLSASIRPDAKMEYGIGYDAQXASKYT**AHQ---

Rickettsia sp. mmx79 **DRTDLIVGARVMGGTMNFSDLAVYPEAHAFVVHKVNGRLSKTQSQLDGQVTPFISQPDRTAKTSYNLGLSASIRPDAKMEYGIGYDAQXASKYT**AHQ---

Rickettsia sp. 103 **DRTDLIVGARVMGGTMNFSDLAVYPEAHAFVVHKVNGRLSKTQSQLDGQVTPFISQPDRTAKTSYNLGLSASIRPDAKMEYGXGYDAQIASKYT**AHQ---

Rickettsia sp. J52 **DRTDLIVGARVMGGTMNFSDLAVYPEAHAFVVHKVNGRLSKTQSQLDGQVTPFISQPDRTAKTSYNLGLSASIRPDAKMEYGIGYDAQXASKYT**AHQ---

Candidatus R. hoogstraalii **DRTDLIVGAKVAGGTMNITDLAVYPEAHAFVVHKVNGRLSKTQSQLDGQVTPFISQPDRTAKTSYNVGLSASIRPDAKMEYGIGYDFNAASKYT**AHQGTL

Rickettsia sp. California 2 **DRTDLIVGVKVAGGTMNITDLAVYPEAHAFVVHKVNGRLSKTQSQLDGQVTPFISQPDRTAKTSYNIGLSASIRPDAKMEYGIGYDFNAASKYT**AHQGT-

R. hulinensis **DRTDLIVGAKVAGGTMNITDFAVYPEVHAFVVHKVTGRLSKTQSVLDGQVTPCISQPDRTAKTSYNLGLSASIRSDAKMEYGIGYDAQIASKYT**AHQGTL

**[1801-1807 ]**

**[ | ]**

R. bellii RML369-C KVRLNF-

R. bellii OSU 85-389 KVRLNF-

R. prowazekii Madrid E KVRVNF-

R. prowazekii Rp22 KVRVNF-

**R. typhi Wilmington KVRINF-**

R. akari Hartford KVRVNF-

R. australis -------

R. felis URRWXCal2 KVRVNF-

R. canadensis McKiel KVRVNF-

R. tamurae -------

R. helvetica -------

REIS K------

R. monacensis K------

R. massiliae MTU5 KVRVNF-

Candidatus R. amblyommii -------

R. rhipicephali -------

R. aeschlimannii -------

R. montanensis -------

R. slovaca 13-B -------

R. peacockii Rustic KVRVNF-

R. rickettsii Iowa KVRVNF-

R. rickettsii Sheila Smith KVRVNF-

R. japonica KVRVNF-

R. honei -------

R. conorii str. Malish 7 KVRVNF-

R. sibirica 246 KVRVNF-

R. africae ESF-5 KVRVNF-

R. parkeri -------

R. heilongjiangensis KVRVNF-

Israeli tick typhus -------

Rickettsia sp. A-167 -------

Candidatus R. tasmanensis KVRVNF-

Rickettsia sp. IG-1 -------

Candidatus R. barbariae -------

Rickettsia sp. S -------

Candidatus R. goldwasserii KVRVKLL

Rickettsia mongolotimonae -------

Rickettsia sp. BJ-90 -------

Candidatus R. andeanae -------

Rickettsia sp. Bar29 -------

Rickettsia sp. TwKM01 -------

R. raoultii -------

R. asiatica -------

Rickettsia sp. 110 -------

Rickettsia sp. mmx79 -------

Rickettsia sp. 103 -------

Rickettsia sp. J52 -------

Candidatus R. hoogstraalii K------

Rickettsia sp. California 2 -------

R. hulinensis -------
